# Nickel-catalyzed enantioselective arylation of pyridine†

**DOI:** 10.1039/c6sc00702c

**Published:** 2016-03-08

**Authors:** J. Patrick Lutz, Stephen T. Chau, Abigail G. Doyle

**Affiliations:** a Department of Chemistry, Princeton University Princeton New Jersey 08544 USA agdoyle@princeton.edu

## Abstract

We report an enantioselective Ni-catalyzed cross coupling of arylzinc reagents with pyridinium ions formed *in situ* from pyridine and a chloroformate. This reaction provides enantioenriched 2-aryl-1,2-dihydropyridine products that can be elaborated to numerous piperidine derivatives with little or no loss in ee. This method is notable for its use of pyridine, a feedstock chemical, to build a versatile, chiral heterocycle in a single synthetic step.

## Abstract 2

A Ni-catalyzed cross coupling of arylzinc reagents with pyridinium ions provides enantioenriched dihydropyridines, which are precursors to diverse piperidine derivatives.

## Graphical abstract

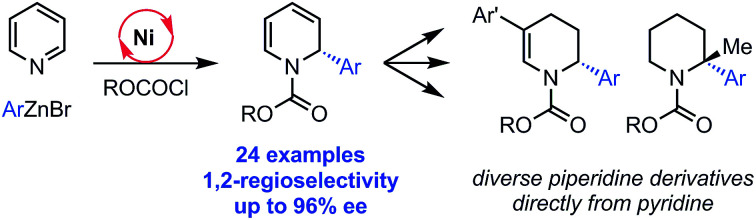

## Introduction

Pyridine and its derivatives comprise one of the most important classes of heterocycles in the field of chemistry. For example, a recent analysis showed that piperidine and pyridine are the two most common nitrogen heterocycles in US FDA-approved pharmaceuticals, together found in approximately 12% of these drugs.^1^ Dihydropyridines are particularly valuable precursors to these motifs and exhibit biological activities in their own right.^2,3^ While the synthetic and biological utility of 1,4-dihydropyridines is well recognized, a recent review has singled out 1,2-dihydropyridines as a class of compounds that is understudied due to limitations in synthetic methodology.^2*e*^ In particular, there is a dearth of methods for the enantioselective formation of 1,2-dihydropyridines.

The most common methods for the synthesis of 1,2-dihydropyridines involve cyclizations or cycloadditions of linear precursors.^4^ Regioselective reductions of substituted pyridines to 1,2-dihydropyridines have also been reported.^5^ However, these strategies have rarely been rendered stereoselective and require the use of prefunctionalized substrates that are accessible only by multi-step synthesis.^6–8^ A third approach involves the addition of a carbon nucleophile to an activated pyridinium salt, which is typically formed *in situ* from the corresponding pyridine and an acylating agent. This approach is particularly attractive, as it employs readily available starting materials and provides 2-substituted 1,2-dihydropyridines in a single step. Furthermore, stereoselective variants have been reported. Comins,^9^ Wanner,^10^ and Charette^11^ have pioneered the use of chiral auxiliaries to control the facial selectivity of Grignard additions to pyridinium salts, though a blocking group at the C3 position is often required for high regio- and diastereoselectivity.

**Scheme 1:**
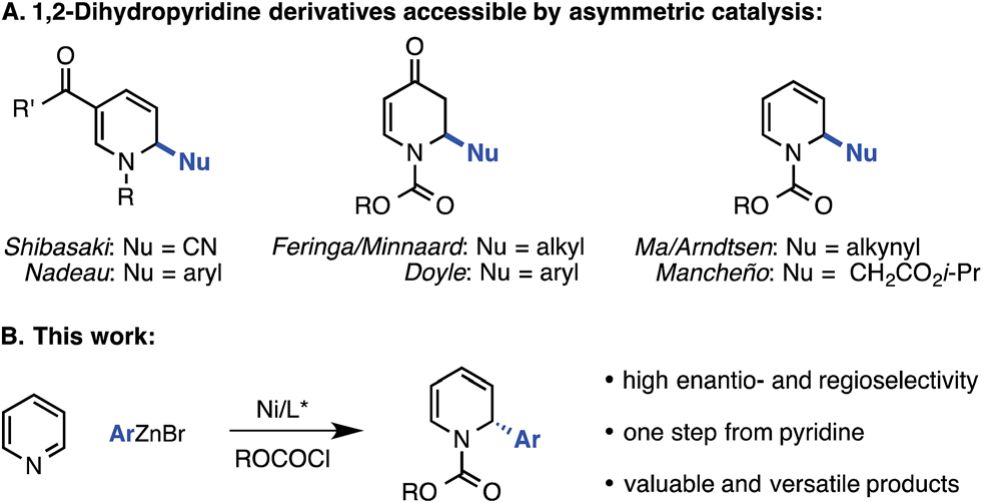
(A) Classes of 1,2-dihydropyridine and piperidinone accessible by asymmetric addition of a nucleophile to a pyridinium salt. (B) Asymmetric arylation of pyridine.

**Scheme 2:**
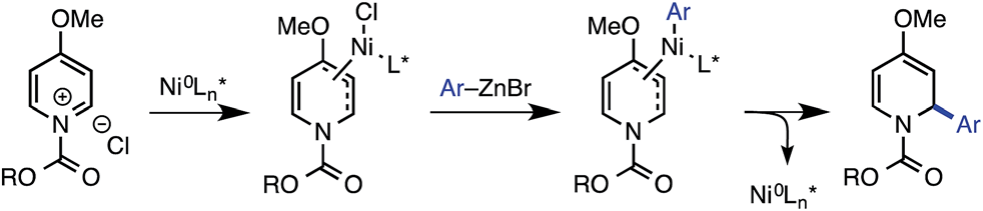
Proposed mechanism for the Ni-catalyzed arylation of 4-methoxypyridinium ions.

By contrast, access to chiral 1,2-dihydropyridines from pyridinium ions in an asymmetric catalytic manner remains largely an unsolved, albeit highly important, problem. Only a few such methods have been disclosed. The first reported example, from Shibasaki, featured the asymmetric addition of cyanide to the 6-position of pyridinium salts derived from nicotinate amides (Scheme 1A).^12^ Another 1,6-addition was achieved by Nadeau and the process group of Merck Frosst in their rhodium-catalyzed coupling of aryl boronic acids with *N*-benzyl nicotinate esters.^13^ These two methods rely on the presence of a carbonyl group conjugated to the pyridinium ring to achieve reactivity, but other reports have demonstrated enantioselective carbon–carbon bond formation in the absence of this structural feature. For example, Feringa and Minnaard reported a copper-catalyzed enantioselective synthesis of 2-alkyl piperidinones from 4-methoxypyridinium salts.^14^ In 2013, we disclosed a catalytic protocol for enantioselective arylation of this same electrophile class. In this process, a Ni(0) catalyst ligated by a chiral phosphoramidite ligand undergoes oxidative addition to the π system of the pyridinium ion, followed by transmetalation with arylzinc halides and reductive elimination to yield 2-aryl-4-methoxy-1,2-dihydropyridines, which are hydrolyzed to the corresponding piperidinones upon workup with aq. HCl (Scheme 2).^15^ Numerous arylzinc nucleophiles were successful coupling partners; nevertheless, 4-methoxypyridinium salt was the only competent electrophile under the reported reaction conditions.

In light of the potential utility of simple 2-aryl-1,2-dihydropyridines, we sought to expand this activation strategy to achieve the enantioselective arylation of unsubstituted pyridinium ions formed *in situ* directly from pyridine and an acylating agent.^16,17^ Pyridine is a stable, abundant and inexpensive feedstock chemical, rendering it an ideal prochiral starting material for organic synthesis. Despite this, pyridine has seen very limited use as a substrate for metal-catalyzed reactions, and a number of potential challenges were evident for the development of the proposed methodology. First, because pyridine is an excellent ligand, it might simply poison the nickel catalyst. While this obstacle was surmountable with 4-methoxypyridine, pyridine is acylated less efficiently than 4-methoxypyridine, so a greater amount of free pyridine would be expected to be present in the reaction mixture.^18^ Another principal concern was whether the catalyst could enforce high regioselectivity for 1,2-addition in the absence of a blocking group installed at the C4 position of the ring. Indeed, only two nucleophile classes have succumbed to coupling with unsubstituted pyridinium ions in an asymmetric fashion: copper acetylides,^19^ and most recently, silyl ketene acetals, though poor regioselectivity for the 1,2-dihydropyridine over the 1,4-dihydropyridine was observed in the latter case.^20^ Here we report the successful translation of our Ni-catalyzed iminium activation mode to the development of a highly regio- and enantioselective catalytic arylation of pyridinium salts derived from pyridine itself (Scheme 1B). Furthermore, we show that the enantioenriched dihydropyridine products of this new reaction can serve as highly valuable precursors to numerous complex piperidine derivatives.

## Results and discussion

### Optimization studies

The previously described reaction conditions, which used PhOCOCl as an acylating agent, provided only trace product when using pyridine in place of 4-methoxypyridine. However, we found that EtOCOCl gave measurable amounts of the desired dihydropyridine product 1, albeit initially in only 14% NMR yield and 42% ee.^21^ Evaluation of precatalysts showed that both Ni(0) and Ni(ii) sources were effective (Table 1, entries 2 and 3), with Ni(acac)_2_ providing the best balance of yield and enantioselectivity (entry 1). The enantioselectivity was not strongly affected by the Ni loading, though below 10 mol% Ni, the reaction yield diminished (entry 4). In the absence of Ni, there is a substantial racemic background reaction, which proceeds to a greater extent than that for 4-methoxypyridine under the original conditions (entry 5).^15^ We also found that concentration has a significant effect on reaction efficiency and selectivity, with higher yields and ee's observed at higher concentration (entry 6). Since equilibrium favors the pyridinium salt at higher reaction concentrations, these results may be explained by diminished catalyst poisoning by free pyridine in solution.

**Table 1:** Evaluation of reaction conditions

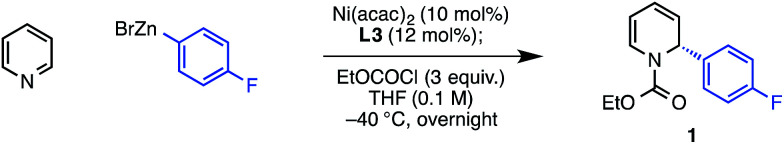			
Entry	Conditions^a^	Yield^b^ (%)	ee^c^ (%)
1	Standard conditions	64	86
2	Ni(cod)_2_ instead of Ni(acac)_2_	71	76
3	NiBr_2_·diglyme instead of Ni(acac)_2_	58	80
4	5 mol% Ni(acac)_2_, 6 mol% L3	38	87
5	No Ni or ligand	72	0
6	0.02 M instead of 0.1 M	56	74
7	Ligand L1 instead of L3	64	74
8	Ligand L2 instead of L3	18	7
9	Ligand L4 instead of L3	24	0
10	−78 °C → rt instead of −40 °C	63	85
11	ArZnBr derived from ArMgBr	43	20
12	+3 equiv. MgBr_2_	13	0
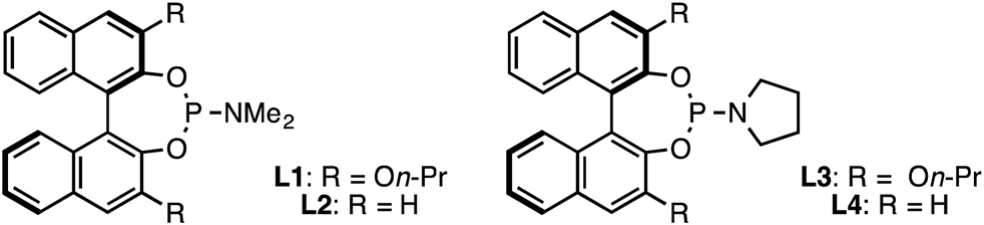			

a Reactions run on a 0.5 mmol scale with 3 equiv. 4-F-PhZnBr.

b Isolated yield.

c Determined using chiral HPLC analysis after Pd/C-catalyzed hydrogenation to the piperidine.

With the modifications described above, the original BINOL-derived phosphoramidite ligand L1 provided 1 with reasonable yield and moderate ee (entry 7). When we investigated the use of (*R*)-Monophos, L2, as a ligand, we found that 3,3′-substitution was critical for high enantioselectivity, showing a dramatic decrease from 74% ee to only 7% ee (entry 8). In an attempt to identify a more selective ligand, we evaluated several Monophos derivatives possessing differing 3,3′-substitution, but we were unable to identify a 3,3′-substituent that gave higher ee than O*n*-Pr. Consequently, we prepared a number of phosphoramidites that retained the 3,3′-O*n*-Pr substitution but that differed in the identity of the *N*-substituent.^22^ Pyrrolidine-derived ligand L3 was optimal, providing 1 in 64% yield and 86% ee (entry 1).

In the course of our optimization studies, we also found that the method of preparation of the arylzinc reagent had a strong influence on the outcome of the reaction. When 4-fluorophenylzinc bromide was prepared by transmetalation of the corresponding aryllithium reagent with ZnBr_2_, the reaction proceeded smoothly and with high ee (entry 1). However, if the zinc reagent was instead prepared by transmetalation between ZnBr_2_ and a Grignard reagent, the yield and enantioselectivity fell off dramatically (entry 11). We believe that this effect is due to the presence of Mg(ii) salts, as the reaction also fails when exogenous MgBr_2_ is added to a reaction with an organolithium-derived zinc reagent (entry 12).

Our early observations regarding the difference in reactivity between EtOCOCl and PhOCOCl led us to investigate the identity of the acylating agent more thoroughly in an attempt to further increase the enantioselectivity of the reaction (Table 2). In doing so, we found that more sterically demanding chloroformates tended to give higher enantioselectivity: methyl chloroformate provided compound 3 in 83% ee, while neopentyl chloroformate provided 6 in 92% ee. Noting that there was only a slight difference in enantioselectivity between isobutyl and neopentyl chloroformate, we elected to use the less expensive reagent, *i*-BuOCOCl, as the acylating agent in our investigation of the reaction scope. Notably, we found that these optimized conditions provided 1,2-adduct 5a with high regioselectivity; none of the 1,4-adduct was observed by NMR.^22^

**Table 2:** Evaluation of acylating agents^a^

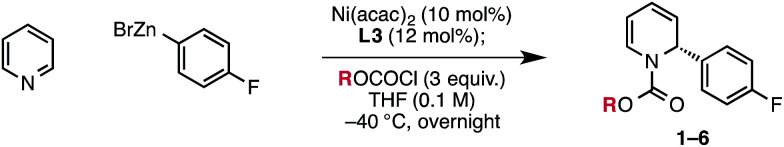
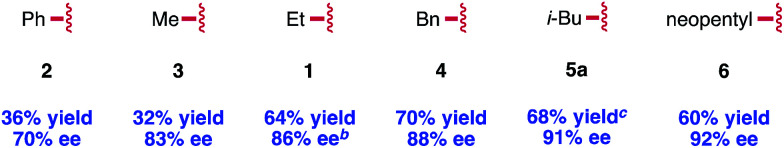

a Isolated yields on a 0.5 mmol scale with 3 equiv. 4-F-PhZnBr.

b Determined after Pd/C-catalyzed hydrogenation to the piperidine.

c Average of two runs.

### Scope elucidation

The scope of the reaction with respect to the arylzinc nucleophile was explored (Table 3). π-Electron-withdrawing groups are well tolerated (5c, 5d), though strong σ-withdrawing substituents lead to depressed enantioselectivity (5e, 5f). Neutral arylzinc bromides give acceptable levels of selectivity (5g–j), as do electron-rich nucleophiles (5k, 5l); the latter is notable as electron-rich nucleophiles deliver low enantioselectivity due to a facile background reaction in our previously described arylation of 4-methoxypyridinium ions. Importantly, many functional groups are compatible with the nickel chemistry: nucleophiles containing nitriles (5c), esters (5d), silyl-protected alcohols (5o), and tertiary amines (5p) all afford >90% ee in the arylation. Furthermore, Cl-substituted nucleophiles perform well, providing a handle for subsequent functionalization at these positions, though the *meta*-Cl nucleophile (5n) gave higher enantioselectivity than the *para*-Cl nucleophile (5m). This trend was observed in a few other cases as well (5i*vs.*5j, 5k*vs.*5l).

**Table 3:** Evaluation of nucleophile scope^a^

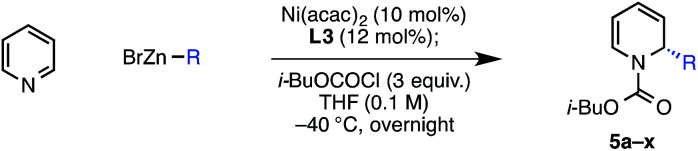
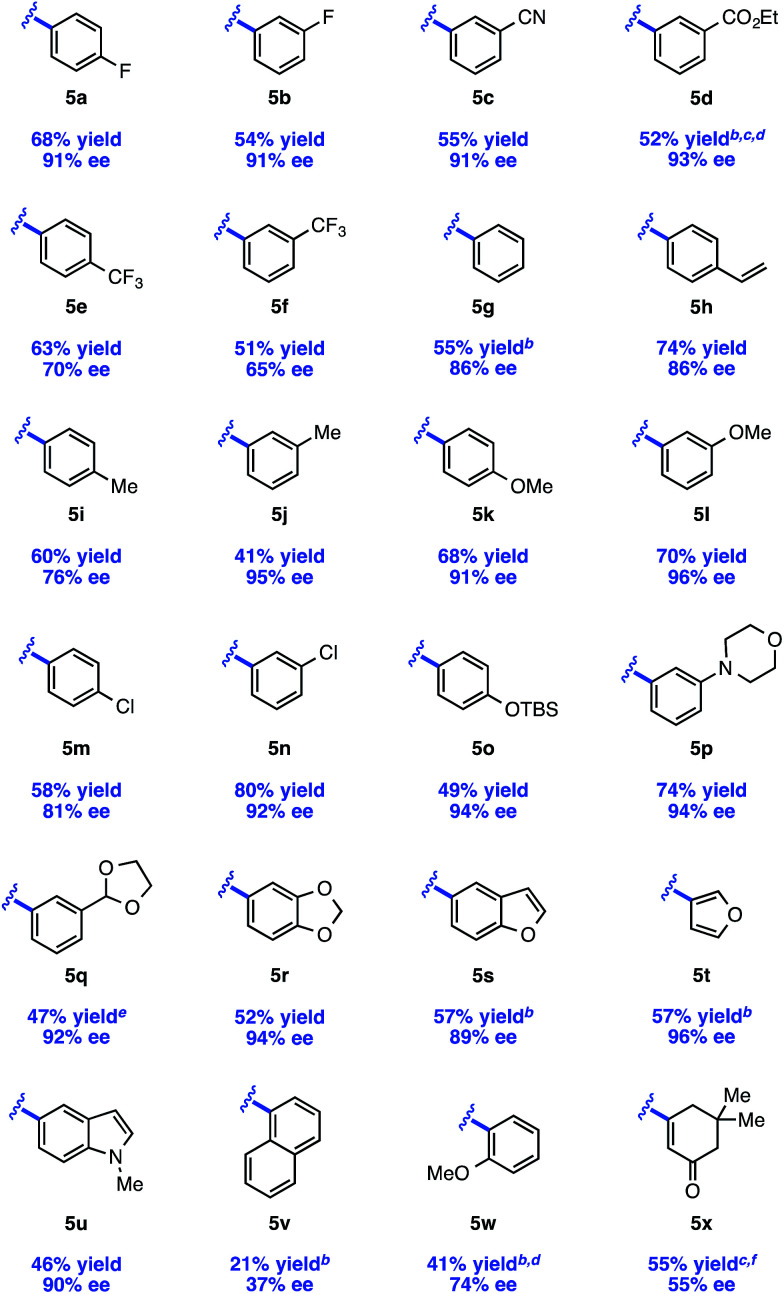

a Isolated yields and ee's are the average of two runs on a 0.5 mmol scale with 3 equiv. RZnBr.

b Reaction was run with 20 mol% Ni(acac)_2_ and 24 mol% L3.

c Reaction was run with RZnI.

d Yield and ee determined after Pd/C-catalyzed hydrogenation to the piperidine.

e Reaction was run with 1.25 equiv. *i*-BuOCOCl.

f Reaction was run from −78 °C → rt.

Several heteroaromatic nucleophiles are amenable to the reaction, providing access to compounds derived from benzofuran (5s), furan (5t), and *N*-methylindole (5u) in ≥89% ee. Nucleophiles substituted at the *ortho* position generally perform poorly in terms of reaction efficiency and selectivity (5v). However, we found that a nucleophile containing coordinating functionality at the *ortho* position, 2-methoxyphenylzinc bromide, delivers a moderate 74% ee (5w). Finally, an alkenylzinc iodide provides 5x in 55% ee. In this case, no reaction was observed at the typical reaction temperature of −40 °C, possibly suggesting a slower transmetalation for this class of alkenyl nucleophile.^23^

While the isolated yields are generally moderate, we believe that the use of pyridine as limiting reagent renders this method practical. Furthermore, upon scaling up the reaction, we observed an increase in reaction efficiency with no influence on selectivity. Under the conditions shown in Table 3, a gram-scale reaction with 4-fluorophenylzinc bromide provided 5a in 80% yield and 90% ee (average of two runs).^24^ Notably, these gram-scale reactions were set up on the benchtop using Schlenk technique, obviating the need for a glovebox.

### Product derivatization

**Scheme 3:**
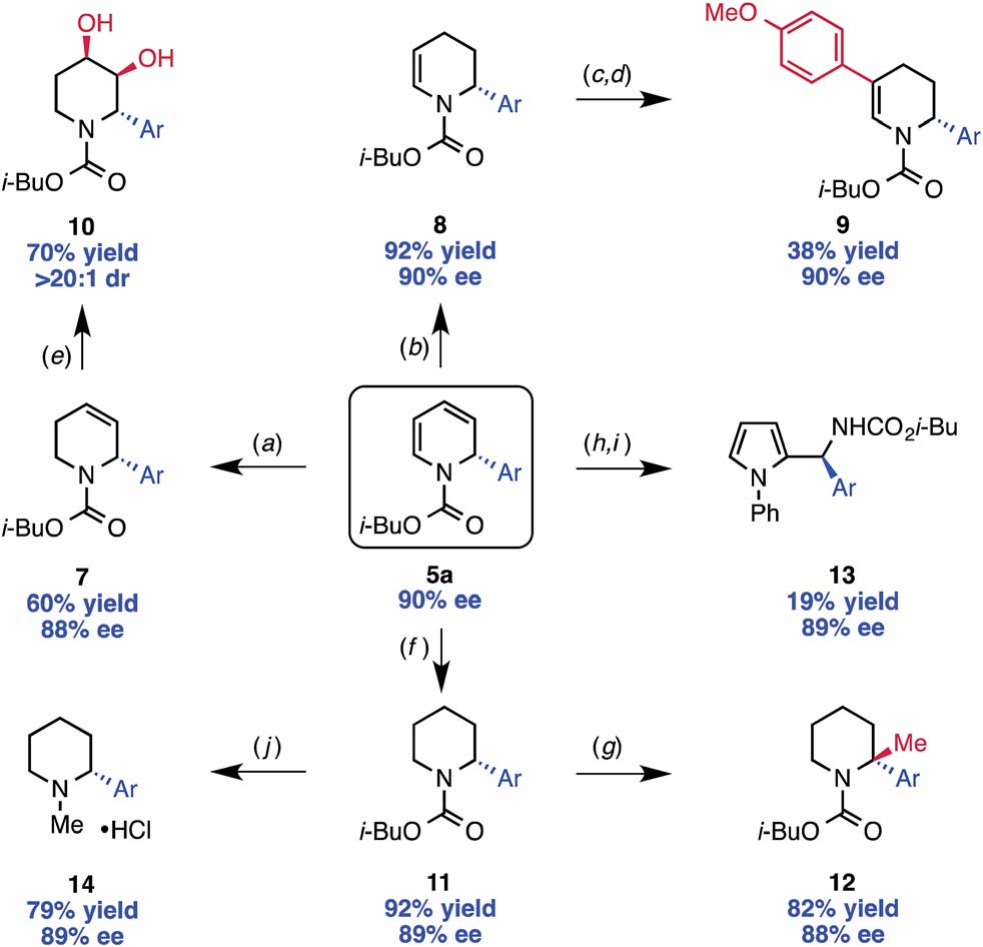
Derivatization of dihydropyridine 5a. Ar = 4-fluorophenyl. Reagents and conditions: (a) Et_3_SiH (1.05 equiv.), trifluoroacetic acid (20 equiv.), CH_2_Cl_2_, −10 °C, 45 s; (b) H_2_ (1 atm), Lindlar catalyst (3 mol%), MeOH, rt, overnight; (c) *N*-iodosuccinimide (3 equiv.), DMF, rt, 2 h; (d) Buchwald precatalyst (2 mol%), XPhos (4 mol%), K_3_PO_4_ (2 equiv.), 4-MeO-PhB(OH)_2_ (1.5 equiv.), THF, 80 °C, overnight; (e) OsO_4_ (4 mol%), *N*-methylmorpholine-*N*-oxide (1.2 equiv.), THF : H_2_O (2 : 1), rt, 3 h; (f) H_2_ (100 psi), Pd/C (5 mol%), MeOH, rt, overnight; (g) *n*-BuLi (1.2 equiv.), THF, −50 °C, 30 min, then MeI (3 equiv.), THF, −78 °C, 2 h; (h) nitrosobenzene (2 equiv.), CH_2_Cl_2_, rt, 2 h; (i) CuCl (20 mol%), MeOH, rt, overnight; (j) LiAlH_4_ (4 equiv.), THF, 50 °C, overnight, then HCl in Et_2_O.

With the scope of the reaction established, we sought to illustrate the utility of the 1,2-dihydropyridine products as valuable building blocks for further organic synthesis. To do so, we explored a number of derivatization reactions, each of which was performed with only minimal optimization (Scheme 3). Importantly, these reactions proceeded with essentially no erosion of ee. The 1,2-dihydropyridine 5a can be selectively reduced with triethylsilane to give tetrahydropyridine 7,^20^ or with Lindlar's catalyst and H_2_ to give the regioisomeric tetrahydropyridine 8.^4*d*^ These compounds are valuable synthetic building blocks in their own right. For example, tetrahydropyridine 8 was further functionalized by iodination with NIS followed by Suzuki cross coupling to provide disubstituted tetrahydropyridine 9. Compound 7 was dihydroxylated with OsO_4_ with >20 : 1 dr (10), demonstrating that the stereocenter set in the pyridinium arylation can provide high substrate control for a diastereoselective reaction.

Full hydrogenation of 5a yields the piperidine product 11, which can be lithiated at low temperature and trapped with an appropriate electrophile with almost no loss in ee, allowing for the formation of a fully substituted, stereodefined carbon (12).^25^ The diene system of 5a is also amenable to Diels–Alder cycloaddition. An interesting example is the Diels–Alder reaction with nitrosobenzene, which provides an unusual bicycle that can be opened with CuCl to afford the chiral pyrrole derivative 13.^26^

**Scheme 4:**
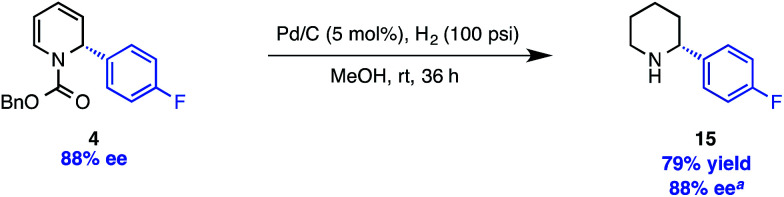
Hydrogenation/deprotection of Cbz-protected dihydropyridine. ^*a*^Determined after Boc protection.

The carbamate protecting group can be removed from 11 with LiAlH_4_ to provide *N*-methyl piperidine derivative 14. For access to the free N–H piperidine 15, we found that the most convenient route was the hydrogenation of the Cbz-protected dihydropyridine 4, which was prepared in only slightly lower ee than 5a (88% *vs.* 91%, Scheme 4) under our standard reaction conditions.

## Conclusions

In summary, we have developed an enantioselective synthesis of 2-aryl-1,2-dihydropyridines, previously only accessible in a diastereoselective or racemic fashion. The reaction proceeds through a pyridinium salt derived from pyridine and an inexpensive chloroformate, and the products are formed by Ni-catalyzed cross coupling with arylzinc halide nucleophiles. The dihydropyridine products are valuable chiral building blocks, as demonstrated by a number of transformations that proceed with little or no loss in ee, providing a variety of chiral piperidine derivatives. More generally, this methodology is noteworthy for its use of pyridine, a feedstock chemical, as a prochiral substrate in a transition metal-catalyzed asymmetric reaction.

## Supplementary Material

SC-007-C6SC00702C-s001
