# Supplementary material for: Nickel-catalyzed enantioselective arylation of pyridine
Source: Chem Sci. 2016 Mar 8;7(7):4105–9. doi: 10.1039/c6sc00702c (PMC5207490; doi:10.1039/c6sc00702c)
Supplement: SC-007-C6SC00702C-s001 [file SC-007-C6SC00702C-s001.pdf]

Electronic Supplementary Material (ESI) for Chemical Science.
This journal is © The Royal Society of Chemistry 2016

Supporting Information

Nickel-Catalyzed Enantioselective Arylation of Pyridine

J. Patrick Lutz, Stephen T. Chau, and Abigail G. Doyle*

Email: agdoyle@princeton.edu

Department of Chemistry, Frick Chemical Laboratory, Princeton University

Princeton, New Jersey 08544, USA

I. General Information.....S-2
II. General Procedures.....S-4
III. Ligand Synthesis/Characterization.....S-6
IV. Additional Optimization Details.....S-9
V. Characterization of Compounds **1**–**6**.....S-11
VI. Derivatization Procedures/Characterization of Compounds **7**–**15**.....S-55
VII. VT-NMR Studies of **5a**.....S-72
VIII. Crude NMR of **5a**.....S-74
IX. NMR Spectra.....S-76

I. General Information

General Procedures. Unless otherwise noted, reactions were performed with rigorous exclusion of air and moisture. Plastic syringes were used to transfer air- and moisture-sensitive reagents. Solvent was freshly distilled/degassed prior to use unless otherwise noted. Reactions were monitored by thin-layer chromatography (TLC) on EMD Silica Gel 60 F_{254} plates, visualizing with UV-light (254 nm) fluorescence quenching and $KMnO_4$ stain. Organic solutions were concentrated under reduced pressure using a rotary evaporator (25 °C, <50 torr). Automated column chromatography was performed using silica gel cartridges on a Biotage SP4 (40–53 µm, 60 Å). SiliaFlash® Irregular silica gel P60 (40–63 µm, 230–400 mesh) was used as received.

Materials. Commercial reagents were purchased from Sigma Aldrich, VWR, Fisher, Oakwood, or Strem, and used as received with the following exceptions. Dichloromethane, tetrahydrofuran (THF), dimethylformamide (DMF) and toluene were dried by passing through activated alumina columns. Nickel(II) acetylacetonate and zinc(II) bromide were purchased from Strem and stored at room temperature in a N_2-filled glovebox. *n*-Butyllithium was purchased from Sigma Aldrich and titrated with diphenylacetic acid prior to use.[1] Anhydrous pyridine was purchased from Sigma Aldrich in a Sure/Seal bottle. *N*-Iodosuccinimide was purchsed from Oakwood and recrystallized from Et_2O:dioxane (1:1) prior to use.

Instrumentation. Proton nuclear magnetic resonance (^1H NMR) spectra were recorded on a Bruker 500 AVANCE spectrometer or a Bruker NB 300 spectrometer (500 and 300 MHz, respectively). Proton chemical shifts are reported in parts per million downfield from tetramethylsilane and referenced to residual protium in the NMR solvent ($CDCl_3$ = δ 7.26 ppm, C_6D_6 = δ 7.16 ppm). Carbon nuclear magnetic resonance (^{13}C NMR) spectra were recorded on a Bruker 500 AVANCE spectrometer (125 MHz). Chemical shifts for carbon are reported in parts per million downfield from tetramethylsilane and are referenced to the carbon resonances of the solvent residual peak ($CDCl_3$ = δ 77.00 ppm, C_6D_6 = δ 128.06 ppm). ^{19}F spectra (282 MHz) and ^{31}P spectra (121 MHz) were recorded on a Bruker NB 300 spectrometer; chemical shifts are reported in parts per million. VT-NMR experiments were performed on a Varian Inova 400 spectrometer. ^{13}C,^{19}F-HMQC experiments were performed on a Bruker 800 AVANCE spectrometer. NMR data are represented as follows: chemical shift (δ ppm), multiplicity (s = singlet, d = doublet, t = triplet, q = quartet, m = multiplet), coupling constant in Hertz (Hz), integration. Peaks attributed to rotamers have been denoted in square brackets, with the major rotamer marked with an asterisk, where applicable.

High-resolution mass spectra were obtained on an Agilent 6220 using electrospray ionization time-of-flight (ESI-TOF). FT-IR spectra were recorded on a Perkin-Elmer Spectrum 100 and are reported in terms of frequency of absorption (cm^{-1}). Reversed-phase liquid chromatography/mass spectrometry (LC/MS) was performed on an Agilent 1260 Infinity analytical LC and Agilent 6120 Quadrupole LC/MS system, using electrospray ionization/atmospheric-pressure chemical ionization (ESI/APCI), and UV detection at 254 and 280 nm. High-performance liquid chromatography (HPLC) was performed on an

[1] Kofron, W. G.; Baclawski, L. M. *J. Org. Chem.* **1976**, *41*, 1879–1880.

Agilent 1200 series instrument with a binary pump and a diode array detector, using Chiralcel OD-H (25 cm x 0.46 cm), Chiralcel OJ-H (25 cm x 0.46 cm), Chiralpak AS-H (25 cm x 0.46 cm), Chiralpak AD-H (25 cm x 0.46 cm), and Regis (*S*,*S*) Whelk-O 1 (25 cm x 0.46 cm) columns. Gas chromatography (GC) was performed on an Agilent 7890A series instrument equipped with a split-mode capillary injection system and flame ionization detectors using a Cyclodex-B (30 m x 0.25 mm) column. Optical rotations were taken with a Jasco P-1010 polarimeter Na/Hal lamp with a 0.5 dm/1 mL cell in spectral grade $CHCl_3$. Where noted, low-temperature reactions were performed in a Thermo Scientific Neslab CB 80 Cryocool.

Absolute Configuration. The absolute configurations of compounds **1**–**14** were assigned by analogy to compound **15**. Compound **15**'s absolute configuration was determined by comparison of the optical rotation to that reported for *ent*-**15** in the literature.[2]

[2] Hussain, S.; Leipold, F.; Man, H.; Wells, E.; France, S. P.; Mulholland, K. R.; Grogan, G.; Turner, N. J. *ChemCatChem* **2015**, *7*, 579–583.

II. General Procedures

Pyridinium Arylation on 0.5-mmol Scale:

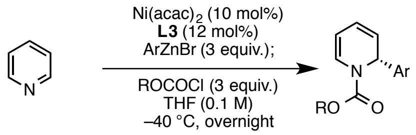

In a nitrogen-filled glovebox, **L3** (30.1 mg, 0.0600 mmol, 0.12 equiv.) was added to a threaded 16 mm x 100 mm glass vial and dissolved in THF (0.5 mL). To this solution was added Ni(acac)$_2$ (12.9 mg, 0.0500 mmol, 0.10 equiv.), followed by THF (0.5 mL). Arylzinc halide (prepared as a 0.60 M solution in THF/hexanes, 2.5 mL, 1.5 mmol, 3.0 equiv.) was added to the Ni/ligand solution. Upon addition of the zinc halide, the solution immediately turned from green and translucent to dark brown and opaque. The glass vial was sealed with a plastic cap lined with a Teflon septum, wrapped with electrical tape, and removed from the glovebox.

Separately, a threaded 16 mm x 100 mm glass vial equipped with a stir bar was put under a nitrogen atmosphere. To this vial was added anhydrous pyridine (39.5 mg, 0.500 mmol, 1.0 equiv.) by weight. The pyridine was dissolved in THF (1 mL), and the Ni/ligand/nucleophile solution was added to the pyridine solution at rt *via* syringe. The vial containing this solution was transferred to a –40 °C Cryocool, and chloroformate (1.5 mmol, 3.0 equiv.) was added at this temperature. The reaction was left to stir at –40 °C overnight (approximately 14 h).

The reaction was quenched with ~5 mL of water and stirred for 10 min at rt. The resulting mixture was diluted with diethyl ether, and the aqueous layer was extracted with diethyl ether (3 x 30 mL). The combined organic layers were dried over MgSO$_4$, and the filtrate was concentrated *in vacuo*. The crude mixture was purified by automated column chromatography (typically 1→10% EtOAc in hexanes) to give the product dihydropyridine.

Pyridinium Arylation on 5-mmol Scale:

Under air, **L3** (301 mg, 0.600 mmol, 0.12 equiv.) and Ni(acac)$_2$ (128 mg, 0.500 mmol, 0.10 equiv.) were added to a flame-dried, 50-mL pear-shaped flask. This flask was sealed with a septum and evacuated/refilled with N$_2$ (x3). The Ni and ligand were dissolved in dry, degassed THF (10 mL). Arylzinc bromide solution (0.60 M in THF/hexanes, 25 mL, 15 mmol, 3.0 equiv.) was added to the Ni/ligand solution, resulting in an immediate color change from green to dark brown.

Pyridine (0.40 mL, 5.0 mmol, 1.0 equiv.) was added under N$_2$ to a flame-dried, 100-mL Schlenk flask equipped with a stir bar, and dissolved in THF (10 mL). To the pyridine solution was added the Ni/ligand/ArZnBr solution *via* syringe. The reaction mixture was cooled to –78 °C, and *i*-BuOCOCl (1.9 mL, 15 mmol, 3.0 equiv.) was added at this temperature. Then, the reaction mixture was transferred to a –40 °C Cryocool, where it was allowed to stir overnight (approximately 14 h).

The reaction was quenched with water (40 mL) and allowed to stir at rt for 10 min. The solution was extracted with Et_2O (3 x 50 mL), and the combined organic fractions were washed with brine (1 x 100 mL). The organic layer was dried over $MgSO_4$ and the filtrate was concentrated *in vacuo*. The crude material was purified by automated column chromatography (1→10% EtOAc in hexanes) to give the product dihydropyridine as an oil.

Formation of Arylzinc Bromide Solutions[3]:

The following procedure was used to prepare the arylzinc bromide solutions, except where noted in Section V.

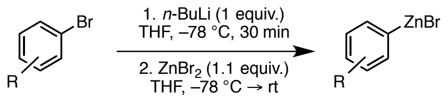

A 50-mL recovery flask equipped with a stir bar was flame dried under vacuum and put under N_2. Bromoarene (7.5 mmol, 1.0 equiv.) was added and dissolved in THF (4 mL). The bromoarene solution was cooled to –78 °C and *n*-BuLi (titrated as 2.2 M in hexanes, 3.4 mL, 7.5 mmol, 1.0 equiv.) was added dropwise. The reaction mixture was allowed to stir at –78 °C for 30 min.

Meanwhile, $ZnBr_2$ (1.86 g, 8.25 mmol, 1.1 equiv.) was weighed into a 50-mL pear-shaped flask under N_2. To the solid was added THF (5 mL), and the mixture was placed in a sonicator until the $ZnBr_2$ had completely dissolved.

After 30 min had elapsed, the $ZnBr_2$ solution was added in one portion to the aryllithium solution at –78 °C. After stirring for 5 min at this temperature, the solution was allowed to warm to rt. If, after warming to rt, the ArZnBr solution was biphasic, more THF was added in 0.5-mL increments with vigorous stirring until a monophasic solution resulted. The ArZnBr solution (nominally 0.6 M) was stored in a N_2-filled glovebox.

Racemic Standards:

Racemic standards for **1**–**6** were prepared in one of two ways: (1) following the "pyridinium arylation on 0.5-mmol scale" procedure, except with PPh_3 (16 mg, 0.060 mmol, 0.12 equiv.) in place of **L3**, or (2) reaction of the appropriate pyridinium salt (prepared *in situ* from pyridine and chloroformate) with the appropriate Grignard reagent in THF.

Racemic standards for **7**–**14** were prepared from **(±)-5a** according to the procedures outlined in Section VI. Similarly, the racemic standard for **15** was prepared from **(±)-4**.

[3] Jensen, A.E.; Kneisel, F.; Knochel, P. *Org. Synth*. **2004**, *Coll. Vol 10*, 391.

III. Ligand Synthesis/Characterization

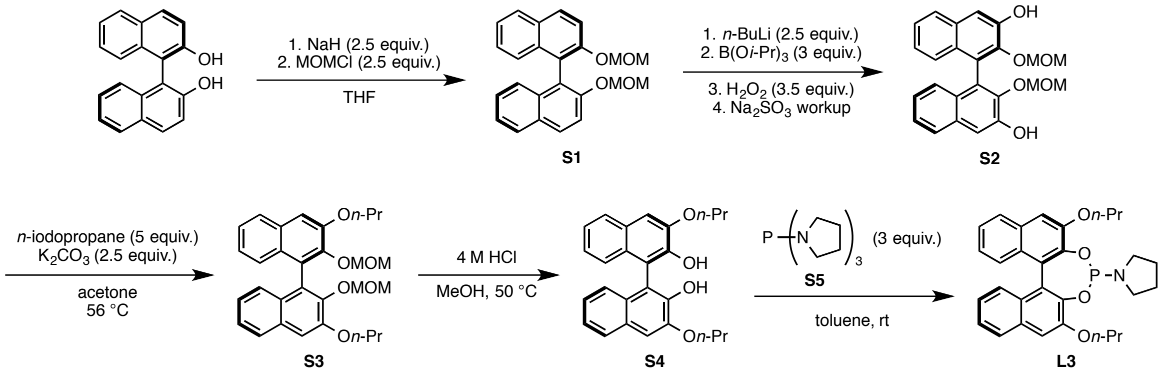

(*R*)-2,2'-bis(methoxymethoxy)-1,1'-binaphthalene (S1)[4]: A flame-dried, 1-L round-bottomed flask equipped with a stir bar was charged with (*R*)-BINOL (30.3 g, 106 mmol, 1.0 equiv.) in dry THF (120 mL). The reaction mixture was cooled to 0 °C and sodium hydride (10.6 g, 264 mmol, 2.5 equiv.) was added portionwise to give a yellow solution. The reaction mixture was warmed to rt and allowed to stir 1 h. The reaction mixture was cooled to 0 °C and MOMCl (20 mL, 260 mmol, 2.5 equiv.) was added *via* syringe. The reaction mixture was allowed to stir at 0 °C for 10 min, then warmed to rt and allowed to stir overnight. The reaction was quenched with sat. NH_4Cl (100 mL) and diluted with water (100 mL). The resulting mixture was extracted with Et_2O (3 x 100 mL) and the combined organic layers were dried with $MgSO_4$ and filtered. The filtrate was concentrated *in vacuo* and the crude product was recrystallized from hexanes:EtOAc (1:1) to give **S1** as a beige solid (34 g, 91 mmol, 86%). All characterization data matched literature values.

^{1}H NMR (500 MHz, $CDCl_3$): δ 7.95 (d, *J* = 9.0 Hz, 2H), 7.87 (d, *J* = 8.1 Hz, 2H), 7.57 (d, *J* = 9.0 Hz, 2H), 7.34 (t, *J* = 8.0 Hz, 2H), 7.22 (m, 2H), 7.15 (d, *J* = 8.5 Hz, 2H), 5.03 (dd, *J* = 53.5, 6.8 Hz, 4H), 3.14 (s, 6H) ppm.

(*R*)-2,2'-bis(methoxymethoxy)-[1,1'-binaphthalene]-3,3'-diol (S2)[5]: A flame-dried, 500-mL round-bottomed flask equipped with a stir bar was charged with **S1** (10 g, 27 mmol, 1.0 equiv.) in dry THF (75 mL). The reaction mixture was cooled to –78°C and *n*-BuLi (2.5 M in hexanes, 27 mL, 67 mmol, 2.5 equiv.) was added to the stirring solution. The solution was allowed to stir at 0 °C for 1 h. The solution was cooled to –78 °C and triisopropyl borate (19 mL, 82 mmol, 3.1 equiv.) was added dropwise. The reaction mixture was warmed to rt and stirred overnight, then concentrated *in vacuo* to give an orange solid. Benzene (100 mL) was added and the solution was cooled to 0 °C. Aqueous hydrogen peroxide (30 wt%, 11 mL, 97 mmol, 3.6 equiv.) was added, and the solution was allowed to stir at 0 °C for 2 h. The reaction mixture was quenched with sat. aq. Na_2SO_3 (150 mL) and extracted with Et_2O (3 x 100 mL). The combined organic portions were dried with $MgSO_4$ and filtered, and the filtrate was concentrated *in vacuo* to give an orange oil. Hexanes were added to the oil and a solid precipitated from solution, and the hexanes were then removed *in vacuo*. The addition of hexanes to precipitate product followed by removing the hexanes *in vacuo* was carried out a total of three times to give crude **S2** as an orange solid (11.3 g, 27.7 mmol, 104%) that was carried directly on to the next step of the synthesis without purification.

[4] Wu, T. R; Shen, L.; Chong, J. M. *Org. Lett.* **2004**, *6*, 2701–2704.
[5] Ooi, T.; Kameda, M.; Maruoka, K.; *J. Am. Chem. Soc.* **2003**, *125*, 5139–5151.

^{1}H NMR (300 MHz, CDCl$_3$): δ 7.77 (d, *J* = 8.2 Hz, 2H), 7.50 (s, 2H), 7.42 (s, 2H), 7.37 (t, *J* = 7.7 Hz, 2H), 7.12 (t, *J* = 7.6 Hz, 2H), 7.04 (d, *J* = 8.1 Hz, 2H), 4.74–4.57 (m, 4H), 3.39 (s, 6H) ppm.

(*R*)-2,2'-bis(methoxymethoxy)-[1,1'-binaphthalene]-3,3'-diol (S3)[5]: A 200-mL recovery flask equipped with a stir bar was charged with **S2** (11.3 g, 27.7 mmol, 1.0 equiv.) dissolved in acetone (20 mL). K_2CO_3 (9.6 g, 69 mmol, 2.5 equiv.) and 1-iodopropane (13.5 mL, 139 mmol, 5.0 equiv.) were added to the solution. The flask was capped with a reflux condenser and the solution was stirred at reflux overnight. The reaction was quenched with water (80 mL) and diluted with Et_2O (40 mL), then extracted with Et_2O (4 x 30 mL), dried with $MgSO_4$, and filtered. The filtrate was concentrated *in vacuo* to give an orange solid (10.3 g, 21.0 mmol, 76%), which was used directly in the next step without further purification.

^{1}H NMR (300 MHz, CDCl$_3$): δ 7.73 (d, *J* = 8.2 Hz, 2H), 7.34 (t, *J* = 8.0 Hz, 2H), 7.28 (s, 2H), 7.19–7.05 (m, 4H), 4.95 (dd, *J* = 49.2, 5.7 Hz, 4H), 4.13 (t, *J* = 6.4 Hz, 4H), 2.52 (s, 6H), 2.04–1.85 (m, 4H), 1.11 (t, *J* = 7.4 Hz, 6H) ppm.

(*R*)-3,3'-dipropoxy-[1,1'-binaphthalene]-2,2'-diol (S4): A 200-mL recovery flask equipped with a stir bar was charged with **S3** (10.3 g, 21.0 mmol) dissolved in MeOH (30 mL). Aqueous HCl (4 M, 21 mL) was added, and the reaction mixture was allowed to stir at 50 °C for 36 h. The reaction mixture was diluted with water (40 mL) and Et_2O (30 mL), then extracted with Et_2O (3 x 30 mL). The combined organic portions were dried with $MgSO_4$ and filtered, and the filtrate was concentrated *in vacuo* to give a yellow solid. The crude product mixture was purified using automated column chromatography (5 → 30% EtOAc in hexanes) to give the product as a crystalline, beige solid (4.02 g, 10.0 mmol, 48%).

^{1}H NMR (500 MHz, CDCl$_3$): δ 7.75 (d, *J* = 8.2 Hz, 2H), 7.33–7.28 (m, 2H), 7.27 (s, 2H), 7.17–7.14 (m, 2H), 7.14–7.11 (m, 2H), 6.00 (s, 2H), 4.28–4.18 (m, 4H), 1.96 (sext., *J* = 7.3 Hz, 4H), 1.11 (t, *J* = 7.4 Hz, 6H) ppm.

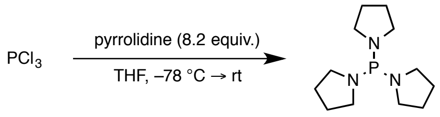

S5

Tri(pyrrolidin-1-yl)phosphane (S5)[6]: In a flame-dried 1-L round-bottomed flask under N_2, pyrrolidine (40 mL, 490 mmol, 8.2 equiv.) was dissolved in dry THF (250 mL). The solution was cooled to –78 °C and PCl_3 (5.2 mL, 59 mmol, 1.0 equiv.) was added dropwise. The reaction was allowed to warm to rt, and it was allowed to stir at this temperature overnight. At this time, the solid was removed by means of a Schlenk filter, and the filtrate was collected in a flame-dried 500-mL Schlenk flask. The solvent was removed *in vacuo* to give a pale yellow oil (12.7 g, 52.5 mmol, 88%), which was used directly in the next step with no further purification. (The NMR spectra were taken in C_6D_6 because **S5** was found to oxidize rapidly in $CDCl_3$).

^{1}H NMR (500 MHz, C$_6$D$_6$): δ 3.12 (td, *J* = 6.6, 4.2 Hz, 12H), 1.60 (m, 12H) ppm.

^{31}P NMR (121 MHz, C$_6$D$_6$): δ 104.18 (s) ppm.

[6] Boltukhina, E. V.; Sheshenev, A. E.; Lyapkalo, I. M. *Tetrahedron*, **2011**, *67*, 5382–5388.

(*R*)-1-(2,6-dipropoxydinaphtho[2,1-d:1',2'-f][1,3,2]dioxaphosphepin-4-yl)pyrrolidine (L3)[7]: A 50-mL round-bottomed flask under N_2 equipped with a stir bar was charged with **S4** (4.57 g, 11.4 mmol, 1.0 equiv.) in toluene (20 mL). **S5** (7.9 mL, 34 mmol, 3.0 equiv.) was added, and the reaction mixture was allowed to stir for 3 h at rt. The toluene was removed *in vacuo* to give a yellow oil. Diethyl ether was added to precipitate the ligand, which was collected in a fritted funnel as a white, crystalline solid (3.2 g, 6.3 mmol, 56%). **L3** was stored in a N_2-filled glovebox.

^{1}H NMR (500 MHz, $CDCl_3$): δ 7.77 (d, *J* = 8.2 Hz, 2H), 7.40–7.31 (m, 2H), 7.31 (d, *J* = 9.3 Hz, 2H), 7.26–7.19 (m, 2H), 7.17–7.02 (m, 2H), 4.29–4.01 (m, 4H), 3.20 (dq, *J* = 11.1, 6.6, 5.9 Hz, 2H), 2.96–2.78 (m, 2H), 1.96 (tt, *J* = 14.2, 6.8 Hz, 4H), 1.73–1.59 (m, 4H), 1.12 (q, *J* = 7.5 Hz, 6H) ppm.

^{13}C NMR (125 MHz, $CDCl_3$): δ 150.83 (d, *J* = 7.7 Hz), 142.76 (d, *J* = 13.2 Hz), 131.49 (d, *J* = 72.4 Hz), 127.67 (d, *J* = 9.5 Hz), 127.10 (d, *J* = 4.4 Hz), 126.98 (d, *J* = 9.4 Hz), 125.80 (d, *J* = 5.1 Hz), 125.11 (d, *J* = 24.6 Hz), 123.61 (d, *J* = 7.3 Hz), 108.40 (d, *J* = 81.5 Hz), 70.38 (d, *J* = 24.1 Hz), 45.45 (d, *J* = 15.3 Hz), 25.93 (d, *J* = 4.6 Hz), 22.71 (d, *J* = 36.9 Hz), 10.81 (d, *J* = 11.1 Hz) ppm.

^{31}P NMR (121 MHz, $CDCl_3$): δ 151.03 ppm.

HRMS: The title compound appears to fragment completely on ionization; a mass of 403.1895, corresponding to [diol **S4** + H]$^+$, was observed ($C_{26}H_{27}O_4^+$, calc'd: 403.1904).

FTIR (thin film, cm^{-1}): 2965, 2936, 2874, 1595, 1440, 1247, 1109.

Optical rotation: $[\alpha]_D^{26}$ −484.7 (*c* 1.0, $CHCl_3$).

[7] Kangying, L.; Zhenghong, Z.; Guofeng, Z.; Chuchi, T.; *Heteroatom. Chem.* **2003**, *14*, 546–550.

IV. Additional Optimization

Table S1. Ligand evaluation. Reactions were run on a 0.05-mmol scale, and ee's were determined after Pd/C-catalyzed hydrogenation of **1** to the piperidine.

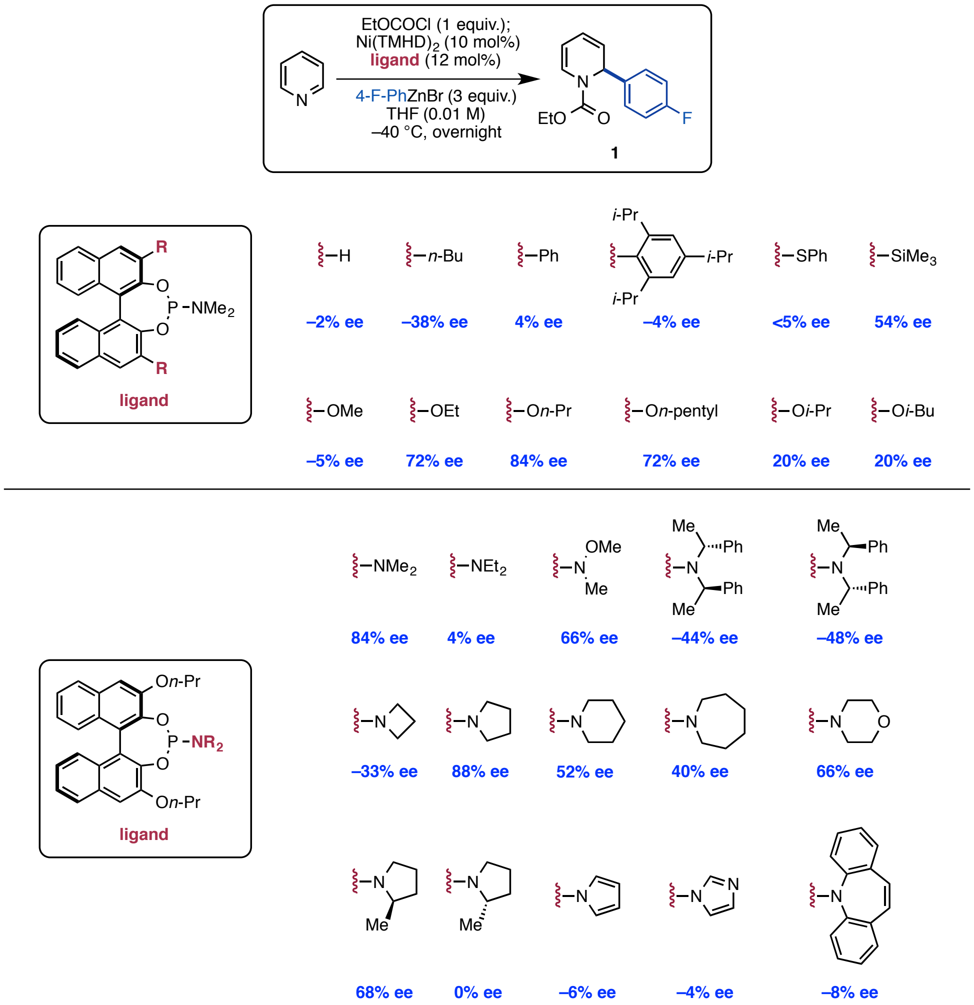

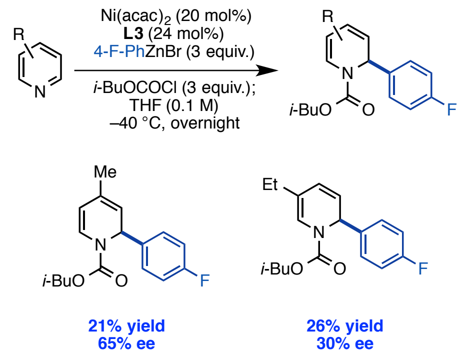

Figure S1. Preliminary results with alkyl-substituted electrophiles in the Ni-catalyzed arylation.

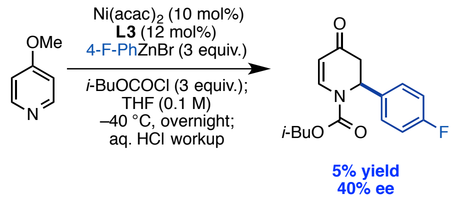

Figure S2. 4-Methoxypyridine electrophile in the Ni-catalyzed arylation.

V. Characterization of Compounds 1–6

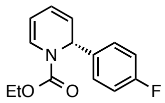

1

(*R*)-Ethyl 2-(4-fluorophenyl)pyridine-1(2H)-carboxylate (1): General procedure was followed with pyridine (42 mg, 0.53 mmol), providing **1** (84 mg, 0.34 mmol, 64% yield, 86% ee) as a clear, colorless oil. Note that the ee was determined after Pd/C-catalyzed hydrogenation to the piperidine.

^{1}H NMR (300 MHz, $CDCl_3$): δ 7.50–7.32 (m, 2H), 7.05–6.93 (m, 2H), 6.82 (dd, *J* = 54.1, 7.3 Hz, 1H), 6.19–5.93 (m, 1H), 5.94–5.69 (m, 1H), 5.70–5.53 (m, 1H), 5.39–5.14 (m, 1H), 4.19 (q, *J* = 7.1 Hz, 2H), 1.36–1.18 (m, 3H) ppm.

^{13}C NMR (125 MHz, $CDCl_3$): δ 162.56 (d, *J* = 245.4 Hz), [154.33, 153.79*], [138.06, 137.00*], [129.28* (d, *J* = 8.0 Hz), 128.52 (d, *J* = 7.5 Hz)], [126.05, 125.26*], 122.42, [121.21*, 120.97], 115.41 (d, *J* = 21.4 Hz), [104.88*, 104.75], [62.52*, 62.44], [55.90, 54.87*], [14.59*, 14.53] ppm.

^{19}F NMR (282 MHz, $CDCl_3$): δ –114.51 (tt, *J* = 8.7, 5.4 Hz) ppm.

HRMS: (ESI-TOF) calculated for $C_{14}H_{15}FNO_2$ ($[M+H]^+$): 248.1081, found: 248.1079.

FTIR (thin film, cm^{-1}): 2985, 1706, 1509, 1324.

Optical rotation (for the dihydropyridine): $[\alpha]_D^{26}$ +230.7 (*c* 1.1, $CHCl_3$).

HPLC (for the piperidine): Chiralpak AS-H, 5% IPA in hexanes, 15 min run, 1 mL/min.

Racemic Standard:

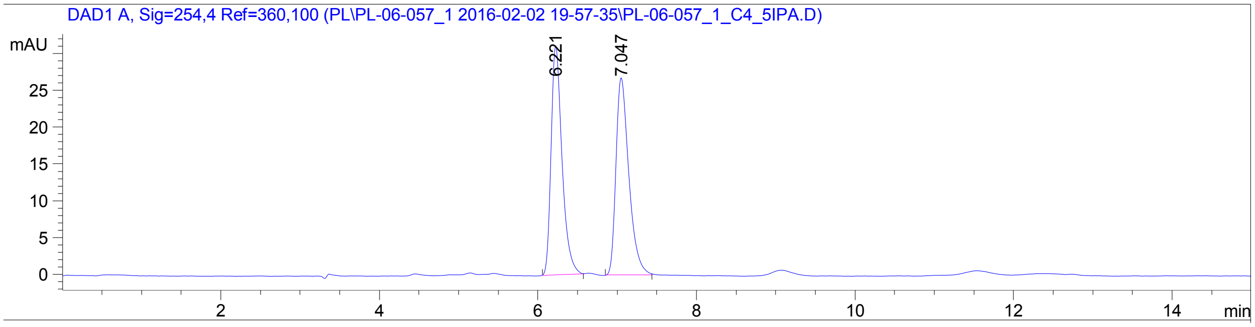

Peak #	RetTime [min]	Type	Width [min]	Area [mAU*s]	Height [mAU]	Area %
1	6.221	BB	0.1460	299.02402	31.16124	49.6400
2	7.047	BB	0.1720	303.36081	26.81054	50.3600
Totals :				602.38483	57.97178	

Enantioenriched:

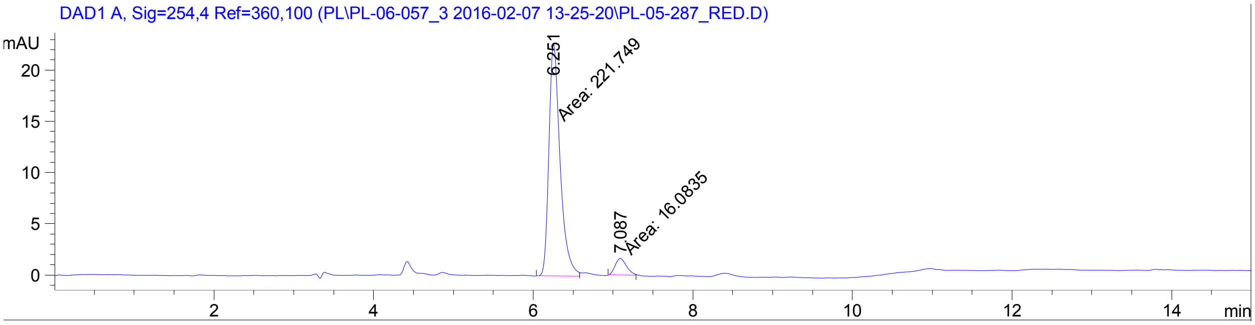

Peak #	RetTime [min]	Type	Width [min]	Area [mAU*s]	Height [mAU]	Area %
1	6.251	MM	0.1631	221.74915	22.66359	93.2375
2	7.087	MM	0.1655	16.08355	1.61962	6.7625
Totals :				237.83270	24.28320	

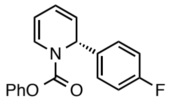

2

(*R*)-Phenyl 2-(4-fluorophenyl)pyridine-1(2H)-carboxylate (2): General procedure was followed with pyridine (40 mg, 0.50 mmol), providing **2** (53 mg, 0.18 mmol, 36% yield, 70% ee) as a clear, colorless oil.

^{1}H NMR (500 MHz, $CDCl_3$): δ 7.54–7.40 (m, 2H), 7.35 (q, *J* = 7.4 Hz, 2H), 7.31–7.26 (m, 1H), 7.22 (t, *J* = 7.4 Hz, 1H), 7.11 (d, *J* = 7.9 Hz, 1H), 7.06–6.99 (m, 2H), 6.98–6.84 (m, 1H), 6.11 (ddd, *J* = 44.6, 9.4, 5.6 Hz, 1H), 5.94 (d, *J* = 5.7 Hz, 1H), 5.78–5.63 (m, 1H), 5.42 (q, *J* = 6.0, 5.4 Hz, 1H) ppm.

^{13}C NMR (125 MHz, CDCl$_3$): δ [162.72* (d, *J* = 246.5 Hz), 162.69 (d, *J* = 247.4 Hz)], [152.29, 150.96*], [137.67, 136.10* (d, *J* = 3.2 Hz)], [129.72* (d, *J* = 8.2 Hz), 128.68 (d, *J* = 8.1 Hz)], [129.71*, 129.64], [129.59, 129.52*], [126.08, 125.92*], [125.69, 124.81*], [123.00, 122.81*], [121.69*, 121.64], [121.27*, 120.84], 115.59 (app. t, *J* = 20.6 Hz), [106.15*, 105.83], [56.62, 55.27*] ppm.

^{19}F NMR (282 MHz, CDCl$_3$): δ [–113.93 (tt, *J* = 9.6, 5.8 Hz), –114.04* (ddd, *J* = 14.1, 8.7, 5.4 Hz)] ppm.

HRMS: (ESI-TOF) calculated for $C_{18}H_{15}FNO_2$ ([M+H]$^+$): 296.1081, found: 296.1083.

FTIR (thin film, cm^{-1}): 3020, 1722, 1336, 1214.

Optical rotation: $[\alpha]_D^{26}$ +449.3 (*c* 0.97, $CHCl_3$).

HPLC: Chiralcel OD-H, 5% IPA in hexanes, 10 min run, 1 mL/min.

Racemic Standard:

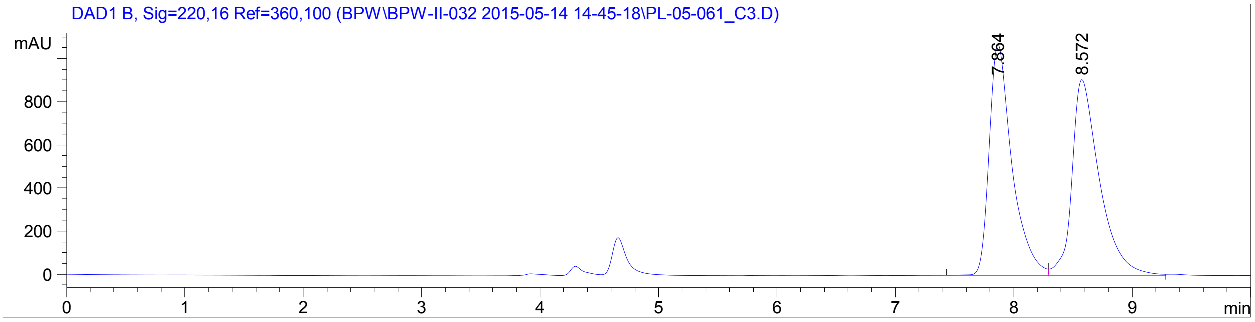

Peak #	RetTime [min]	Type	Width [min]	Area [mAU*s]	Height [mAU]	Area %
1	7.864	BV	0.2006	1.43755e4	1071.23108	49.4559
2	8.572	VV	0.2387	1.46918e4	908.39020	50.5441
Totals :				2.90673e4	1979.62128	

Enantioenriched:

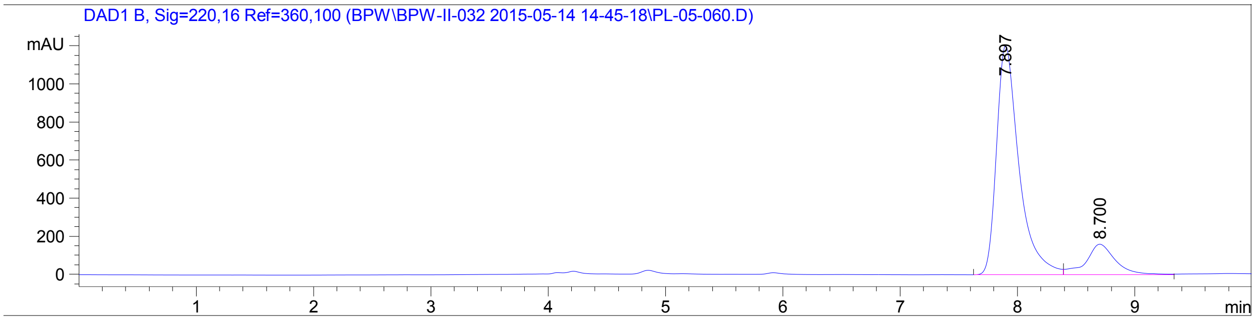

Peak #	RetTime [min]	Type	Width [min]	Area [mAU*s]	Height [mAU]	Area %
1	7.897	BV	0.1995	1.60348e4	1203.39563	85.1188
2	8.700	VB	0.2521	2803.33765	160.24187	14.8812
Totals :				1.88381e4	1363.63750	

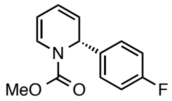

3

(*R*)-Methyl 2-(4-fluorophenyl)pyridine-1(2H)-carboxylate (3): General procedure was followed with pyridine (40 mg, 0.50 mmol), providing **3** (38 mg, 0.16 mmol, 32% yield, 83% ee) as a clear, colorless oil.

^{1}H NMR (300 MHz, $CDCl_3$): δ 7.58–7.29 (m, 2H), 7.08–6.94 (m, 2H), 6.80 (dd, *J* = 61.4, 7.7 Hz, 1H), 6.00–5.98 (m, 1H), 5.92–5.53 (m, 2H), 5.39–5.17 (m, 1H), 3.77 (s, 3H) ppm.

^{13}C NMR (125 MHz, $CDCl_3$): δ [162.58* (d, *J* = 246.3 Hz), 162.52 (d, *J* = 246.8 Hz)], [154.86, 154.25*], [137.81, 136.88*], [129.30* (d, *J* = 8.1 Hz), 128.36 (d, *J* = 7.7 Hz)], [125.99, 125.09*], 121.49, [121.15*, 121.03], [115.49 (d, *J* = 21.9 Hz), 115.42* (d, *J* = 21.3 Hz)], 105.10, [55.73, 55.02*], 53.43 ppm.

^{19}F NMR (282 MHz, $CDCl_3$): δ –114.42 ppm.

HRMS: (ESI-TOF) calculated for $C_{13}H_{13}FNO_2$ ($[M+H]^+$): 234.0925, found: 234.0922.

FTIR (thin film, cm^{-1}): 3019, 2956, 1709, 1508, 1442, 1338.

Optical rotation: $[\alpha]_D^{26}$ +682.6 (*c* 1.1, $CHCl_3$).

HPLC: Chiralcel OJ-H, 5% IPA in hexanes, 20 min run, 1 mL/min.

Racemic Standard:

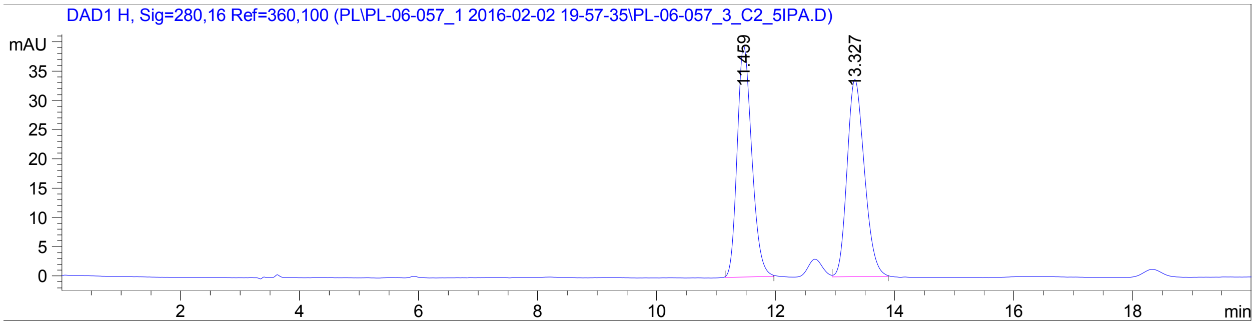

Peak #	RetTime [min]	Type	Width [min]	Area [mAU*s]	Height [mAU]	Area %
1	11.459	BB	0.2616	675.51605	39.49268	49.6359
2	13.327	VB	0.3132	685.42603	33.71619	50.3641
Totals :				1360.94208	73.20887	

Enantioenriched:

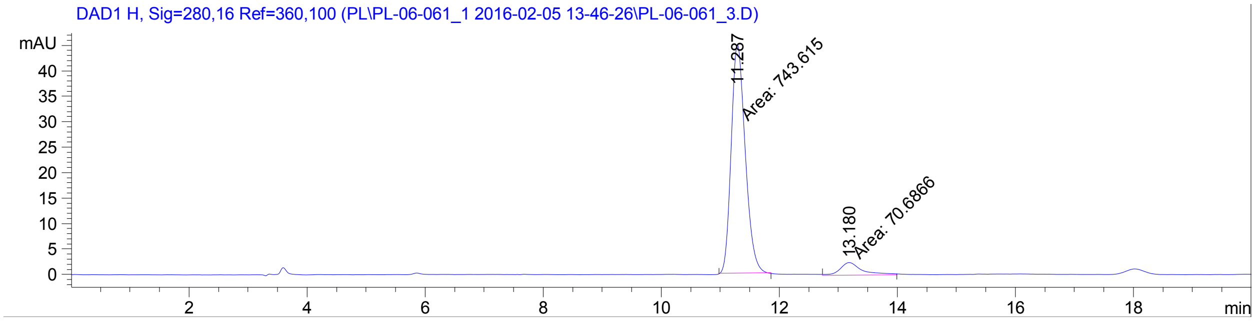

Peak #	RetTime [min]	Type	Width [min]	Area [mAU*s]	Height [mAU]	Area %
1	11.287	MM	0.2756	743.61511	44.97021	91.3194
2	13.180	MM	0.4612	70.68661	2.55429	8.6806
Totals :				814.30173	47.52450	

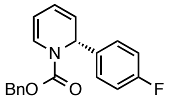

4

(*R*)-Benzyl 2-(4-fluorophenyl)pyridine-1(2H)-carboxylate (4): General procedure was followed with pyridine (40 mg, 0.50 mmol), providing **4** (109 mg, 0.35 mmol, 70% yield, 88% ee) as a clear, colorless oil.

^{1}H NMR (500 MHz, $CDCl_3$): 7.48–7.30 (m, 6H), 7.25–7.17 (m, 1 H), 7.00 (t, *J* = 8.5 Hz, 1H), 6.95–6.92 (m, 1H), 6.75 (d, *J* = 7.7 Hz, 1H), 6.02 (ddd, *J* = 37.3, 8.9, 5.5 Hz, 1H), 5.80 (dd, *J* = 85.0, 5.0 Hz, 1H), 5.69–5.50 (m, 1H), 5.36–5.19 (m, 2H), 5.19–5.10 (m, 1H) ppm.

^{13}C NMR (125 MHz, $CDCl_3$): δ 160.79 (d, J = 239.4 Hz), 150.84, 133.41, [129.51, 128.49*], [129.30 (d, J = 8.3 Hz), 129.03* (d, J = 12.1 Hz)], [128.71*, 128.65], [128.36, 128.29*], [125.96, 125.10*], [122.65*, 122.55], [121.15*, 120.75], 115.45 (d, J = 21.4 Hz), [105.20*, 104.97], 73.60, 68.18, [56.12, 55.11*] ppm.

^{19}F NMR (282 MHz, $CDCl_3$): δ [–114.39*, –114.52] ppm.

HRMS: (ESI-TOF) calculated for $C_{19}H_{17}FNO_2$ ($[M+H]^+$): 310.1238, found: 310.1241.

FTIR (thin film, cm^{-1}): 3038, 1775, 1710, 1508, 1392, 1323.

Optical rotation: $[\alpha]_D^{26}$ +494.7 (c 1.1, $CHCl_3$).

HPLC: Chiralpak AD-H, 2.5% IPA in hexanes, 30 min run, 1 mL/min.

Racemic Standard:

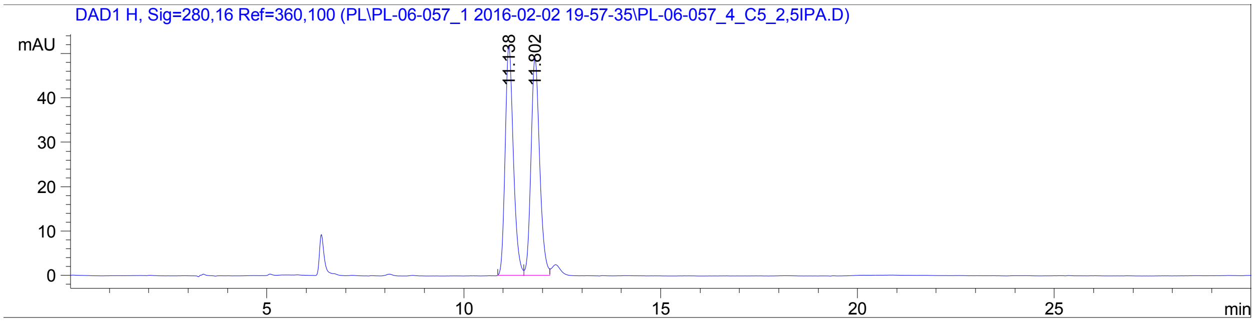

Peak #	RetTime [min]	Type	Width [min]	Area [mAU*s]	Height [mAU]	Area %
1	11.138	BV	0.2212	751.78955	51.83438	49.9090
2	11.802	VV	0.2351	754.53210	49.15238	50.0910
Totals :				1506.32166	100.98676	

Enantioenriched:

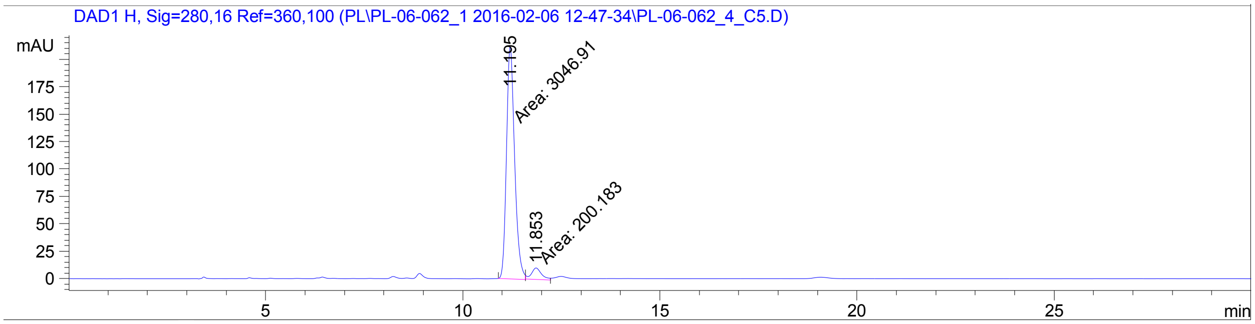

Peak #	RetTime [min]	Type	Width [min]	Area [mAU*s]	Height [mAU]	Area %
1	11.195	MM	0.2397	3046.91138	211.81783	93.8350
2	11.853	MM	0.3100	200.18286	10.76395	6.1650
Totals :				3247.09424	222.58178	

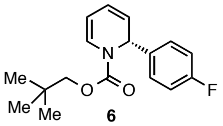

6

(*R*)-Neopentyl 2-(4-fluorophenyl)pyridine-1(2H)-carboxylate (6): General procedure was followed with pyridine (42 mg, 0.53 mmol), providing **6** (91 mg, 0.32 mmol, 60% yield, 92% ee) as a clear, colorless oil.

^{1}H NMR (500 MHz, $CDCl_3$): δ 7.53–7.28 (m, 2H), 7.05–6.65 (m, 3H), 6.16–5.91 (m, 1H), 5.80 (dd, *J* = 74.9, 5.0 Hz, 1H), 5.70–5.58 (m, 1H), 5.33–5.22 (m, 1H), 3.91–3.74 (m, 2H), 0.88 (d, *J* = 61.2 Hz, 9H) ppm.

^{13}C NMR (125 MHz, $CDCl_3$): δ 162.57 (d, *J* = 245.3 Hz), 153.91, [138.93, 136.97*], [129.30* (d, *J* = 8.1 Hz), 127.44 (d, *J* = 7.3 Hz)], [126.41, 125.07*], [122.65, 122.46*], [121.23*, 120.44], [115.61 (d, *J* = 22.2 Hz), 115.41* (d, *J* = 21.5 Hz)], [104.99*, 104.66], [75.95, 75.84*], [56.32, 54.93*], [31.70*, 30.46], [26.59*, 26.48] ppm.

^{19}F NMR (282 MHz, $CDCl_3$): δ [–114.51*, –114.78] ppm.

HRMS: (ESI-TOF) calculated for $C_{17}H_{21}FNO_2$ ($[M+H]^+$): 290.1551, found: 290.1534.

FTIR (thin film, cm^{-1}): 2961, 1709, 1508, 1384, 1323.

Optical rotation: $[\alpha]_D^{26}$ +531.3 (*c* 1.2, $CHCl_3$).

HPLC: Chiralcel OJ-H, 5% IPA in hexanes, 15 min run, 1 mL/min.

Racemic Standard:

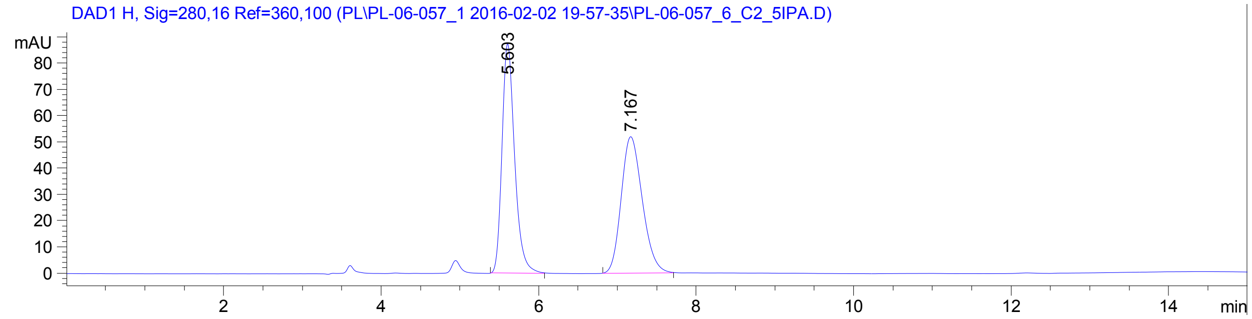

Peak #	RetTime [min]	Type	Width [min]	Area [mAU*s]	Height [mAU]	Area %
1	5.603	BB	0.1702	975.90582	87.48016	50.1826
2	7.167	BB	0.2892	968.80353	52.03045	49.8174

Totals : 1944.70935 139.51061

Enantioenriched:

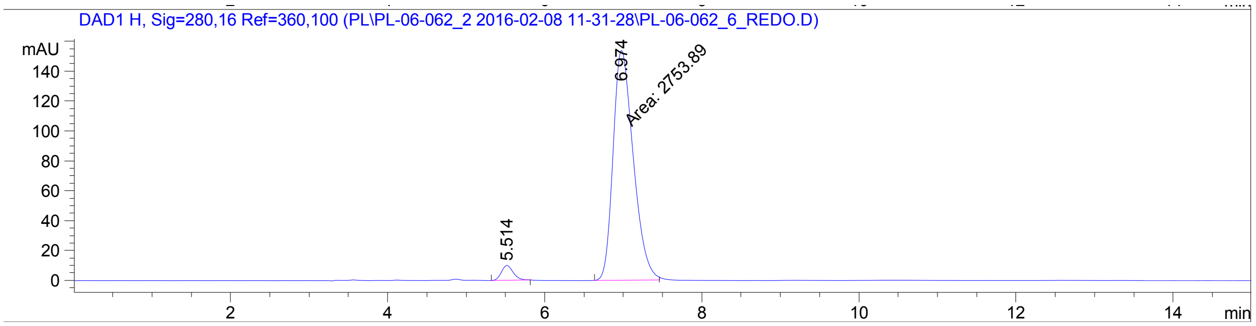

Peak #	RetTime [min]	Type	Width [min]	Area [mAU*s]	Height [mAU]	Area %
1	5.514	BB	0.1635	107.58785	10.00349	3.7599
2	6.974	MF	0.2983	2753.89111	153.85574	96.2401

Totals : 2861.47897 163.85924

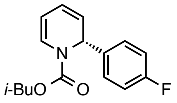

5a

(*R*)-Isobutyl 2-(4-fluorophenyl)pyridine-1(2H)-carboxylate (5a): General procedure was followed with pyridine (39 mg, 0.50 mmol), providing **5a** (97 mg, 0.35 mmol 71% yield, 91% ee) as a clear, colorless oil (run 1). Run 2 provided 65% yield, 90% ee (run 2).

^{1}H NMR (300 MHz, $CDCl_3$): δ 7.50–7.29 (m, 2H), 7.06–6.46 (m, 3H), 6.23–5.92 (m, 1H), 5.91–5.70 (m, 1H), 5.70–5.59 (m, 1H), 5.28 (q, *J* = 6.7 Hz, 1H), 4.03–3.77 (m, 2H), 2.10–1.73 (m, 1H), 0.99–0.66 (m, 6H) ppm.

^{13}C NMR (125 MHz, $CDCl_3$): δ 162.57 (d, *J* = 246.6 Hz), 153.86, 137.02, [129.29* (d, *J* = 7.8 Hz), 127.98 (d, *J* = 7.8 Hz)], [126.24, 125.17*], [122.59, 122.45*], [121.21*, 120.66], [115.52 (d, *J* = 21.4 Hz), 115.41* (d, *J* = 21.2 Hz)], [104.91*, 104.65], [72.62, 72.59*], [56.19, 54.92*], [28.02*, 27.96], 19.20, 19.09 ppm.

^{19}F NMR (282 MHz, $CDCl_3$): δ [–114.53*, –114.65] ppm.

HRMS: (ESI-TOF) calculated for $C_{16}H_{19}FNO_2$ ($[M+H]^+$): 276.1394, found: 276.1400.

FTIR (thin film, cm^{-1}): 2963, 2876, 1706, 1507, 1401, 1383, 1321, 1256, 1110.

Optical rotation: $[\alpha]_D^{26}$ +667.7 (*c* 0.96, $CHCl_3$).

HPLC: Chiralcel OJ-H, 2.5% IPA in hexanes, 30 min run, 0.5 mL/min.

Racemic Standard:

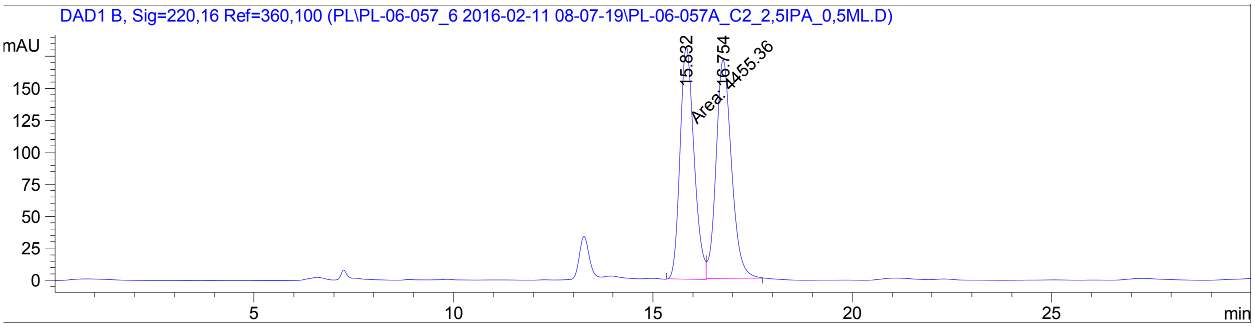

Peak #	RetTime [min]	Type	Width [min]	Area [mAU*s]	Height [mAU]	Area %
1	15.832	MM	0.4082	4455.35986	181.90912	49.1347
2	16.754	VB	0.4128	4612.28857	170.93433	50.8653
Totals :				9067.64844	352.84344	

Enantioenriched:

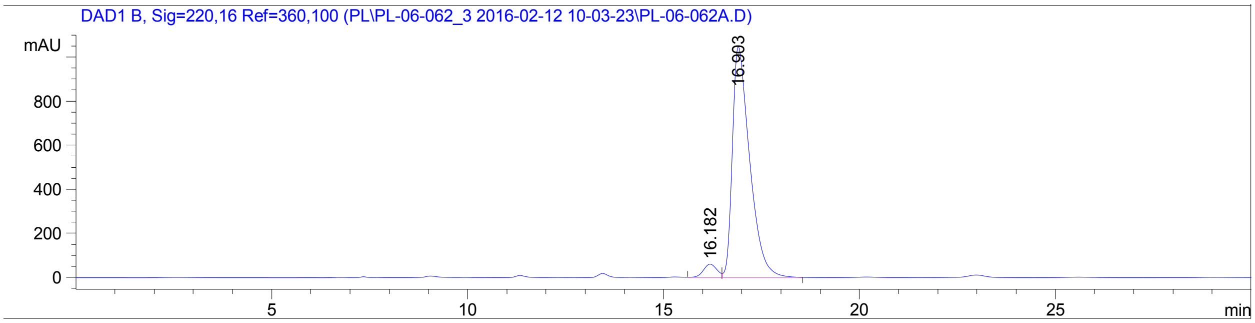

Peak #	RetTime [min]	Type	Width [min]	Area [mAU*s]	Height [mAU]	Area %
1	16.182	VV	0.3740	1475.15527	61.02147	4.3856
2	16.903	VB	0.4583	3.21608e4	1047.28564	95.6144

Totals : 3.36360e4 1108.30711

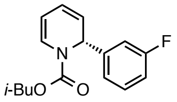

5b

(*R*)-Isobutyl 2-(3-fluorophenyl)pyridine-1(2H)-carboxylate (5b): General procedure was followed with pyridine (42 mg, 0.53 mmol), providing **5b** (77 mg, 0.28 mmol, 52% yield, 91% ee) as a clear, colorless oil (run 1). Run 2 provided 56% yield, 91% ee.

^{1}H NMR (300 MHz, $CDCl_3$): δ 7.29 (dd, *J* = 7.8, 5.8 Hz, 1H), 7.25–7.03 (m, 2H), 7.03–6.70 (m, 2H), 6.13–5.93 (m, 1H), 5.81 (dd, *J* = 46.1, 5.3 Hz, 1H), 5.725.57 (m, 1H), 5.36–5.16 (m, 1H), 4.04–3.76 (m, 2H), 2.13–1.75 (m, 1H), 1.06–0.70 (m, 6H) ppm.

^{13}C NMR (125 MHz, $CDCl_3$): δ 163.11 (d, *J* = 246.1 Hz), [154.39, 153.81*], [145.22 (d, *J* = 5.4 Hz), 143.81* (d, *J* = 6.4 Hz)], [130.23 (d, *J* = 8.2 Hz), 130.07* (d, *J* = 7.8 Hz)], [126.32, 125.30*], [122.75, 121.56*], [122.03, 122.00*], [121.38*, 120.92], 114.79 (app. t, *J* = 19.4 Hz), [114.14* (d, *J* = 21.9 Hz), 113.08 (d, *J* = 21.8 Hz)], [104.95*, 104.61], 72.64, [56.46, 55.17*], [28.01*, 27.91], 19.17, 19.01 ppm.

^{19}F NMR (282 MHz, $CDCl_3$): δ [–112.77, –112.91*] ppm

HRMS: (ESI-TOF) calculated for $C_{16}H_{19}FNO_2$ ($[M+H]^+$): 276.1394, found: 276.1389.

FTIR (thin film, cm^{-1}): 2963, 2875, 1707, 1589, 1400, 1383, 1320, 1254, 1110.

Optical rotation: $[\alpha]_D^{26}$ +508.6 (*c* 1.2, $CHCl_3$).

HPLC: Chiralcel OJ-H, 5% IPA in hexanes, 15 min run, 1 mL/min.

Racemic Standard:

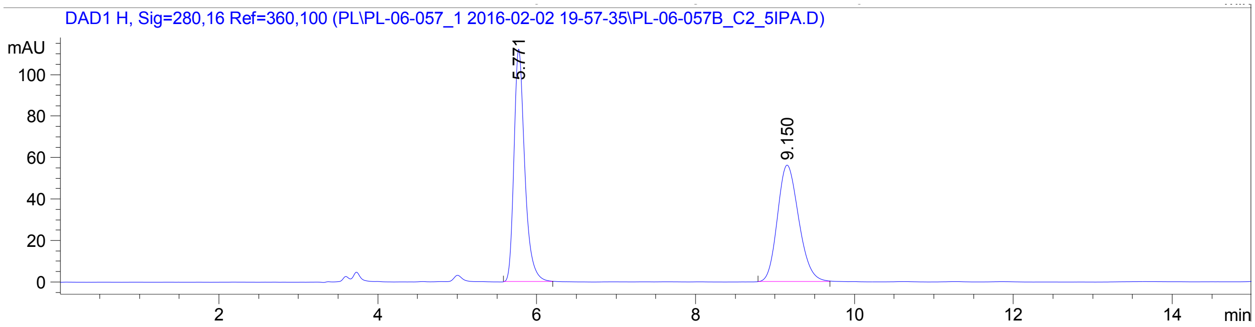

Peak #	RetTime [min]	Type	Width [min]	Area [mAU*s]	Height [mAU]	Area %
1	5.771	BB	0.1438	1057.17163	112.29976	50.1896
2	9.150	BB	0.2896	1049.18640	56.25233	49.8104

Totals : 2106.35803 168.55209

Enantioenriched:

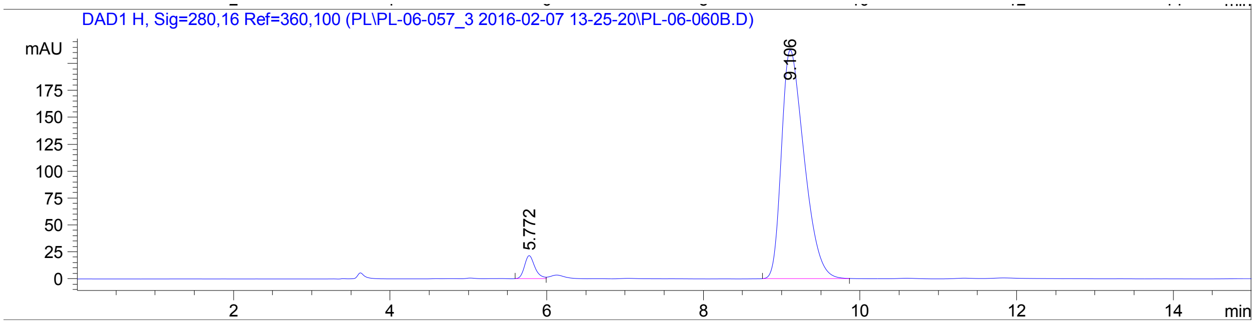

Peak #	RetTime [min]	Type	Width [min]	Area [mAU*s]	Height [mAU]	Area %
1	5.772	BV	0.1396	197.59418	21.41875	4.4165
2	9.106	BB	0.3087	4276.45166	212.56664	95.5835

Totals : 4474.04584 233.98538

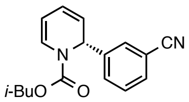

5c

(*R*)-Isobutyl 2-(3-cyanophenyl)pyridine-1(2H)-carboxylate (5c): General procedure was followed wity pyridine (41 mg, 0.52 mmol), with the following modification: *m*-CN-PhZnBr was prepared at –98 °C

(liquid nitrogen/MeOH bath, monitored with a thermocouple) instead of –78 °C. **5c** (82 mg, 0.29 mmol, 56% yield, 90% ee was isolated as a clear, yellow oil (run 1). Run 2 provided 54% yield, 91% ee (run 2).

^{1}H NMR (300 MHz, $CDCl_3$): δ 8.09–7.52 (m, 3H), 7.43 (t, *J* = 7.8 Hz, 1H), 6.88 (dd, *J* = 64.8, 8.1 Hz, 1H), 6.28–5.97 (m, 1H), 5.84 (dd, *J* = 46.6, 6.2 Hz, 1H), 5.71–5.54 (m, 1H), 5.41–5.23 (m, 1H), 4.01–3.78 (m, 2H), 2.10–1.79 (m, 1H), 1.14–0.61 (m, 6H) ppm.

^{13}C NMR (125 MHz, $CDCl_3$): δ 153.80, [143.94, 142.55*], [131.91, 131.65*], 131.14, [130.70*, 129.98], [129.67, 129.45*], [126.34, 125.31*], [122.10*, 121.67], [121.29, 121.21*], [119.03*, 118.81], [112.82, 112.77*], [105.01*, 104.80], 72.84, [56.19, 54.93*], [28.02*, 27.92], 19.18, 19.05 ppm.

HRMS: (ESI-TOF) calculated for $C_{17}H_{19}N_2O_2$ ($[M+H]^+$): 283.1441, found: 283.1423.

FTIR (thin film, cm^{-1}): 3053, 2962, 2875, 2230, 1706, 1401, 1383, 1320, 1256, 1111.

Optical rotation: $[\alpha]_D^{26}$ +620.7 (*c* 1.3, $CHCl_3$).

HPLC: Chiralpak AD-H, 2.5% IPA in hexanes, 30 min run, 1 mL/min.

Racemic Standard:

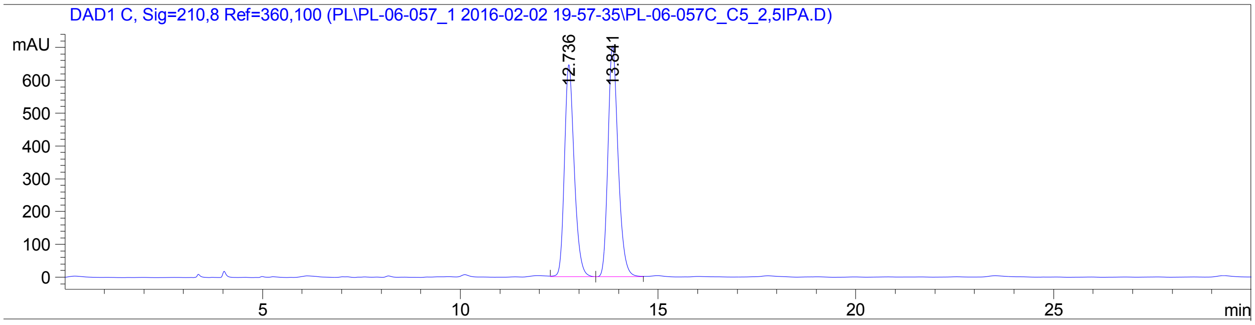

Peak #	RetTime [min]	Type	Width [min]	Area [mAU*s]	Height [mAU]	Area %
1	12.736	MM	0.2994	1.18291e4	658.53851	49.2768
2	13.841	MF	0.2906	1.21763e4	698.23047	50.7232

Totals : 2.40055e4 1356.76898

Enantioenriched:

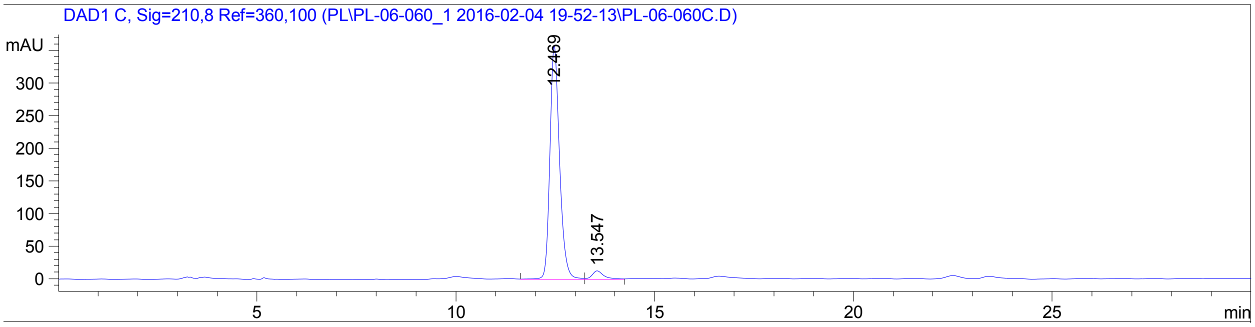

Peak #	RetTime [min]	Type	Width [min]	Area [mAU*s]	Height [mAU]	Area %
1	12.469	VV	0.2632	6102.66943	357.36920	95.3474
2	13.547	VV	0.3230	297.79077	13.41405	4.6526
Totals :				6400.46021	370.78325	

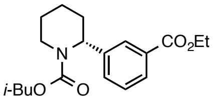

5d

(*R*)-Isobutyl 2-(3-(ethoxycarbonyl)phenyl)piperidine-1-carboxylate (5d): General procedure was followed with pyridine (40 mg, 0.5 mmol), with the following modifications: 3-(ethoxycarbonyl)phenylzinc iodide (0.5 M in THF) was purchased from Sigma-Aldrich. An increased catalyst/ligand loading of 20 mol% Ni(acac)$_2$ and 24 mol% **L3** was used. The dihydropyridine product was difficult to separate from the biaryl, so the dihydropyridine was reduced to piperidine **5d**, which was readily separated from the biaryl, with H_2 (100 psi) and Pd/C (5 mol%) in MeOH. **5d** (93 mg, 0.28 mmol, 56% yield, 93% ee) was isolated as a clear, colorless oil (run 1). Run 2 provided 47% yield, 93% ee (run 2).

^{1}H NMR (300 MHz, CDCl$_3$): δ 7.97–7.92 (m, 2H), 7.47–7.37 (m, 2H), 5.53 (d, *J* = 3.7 Hz, 1H), 4.40 (q, *J* = 7.1 Hz, 2H), 4.15 (d, *J* = 13.1 Hz, 1H), 3.95 (dd, *J* = 6.6, 2.9 Hz, 2H), 2.84 (ddd, *J* = 13.7, 11.3, 4.1 Hz, 1H), 2.45–2.32 (m, 1H), 2.09–1.86 (m, 2H), 1.76–1.57 (m, 3H), 1.57–1.46 (m, 1H), 1.42 (t, *J* = 7.1 Hz, 3H), 0.93 (d, *J* = 6.7 Hz, 6H) ppm.

^{13}C NMR (125 MHz, CDCl$_3$): δ 166.76, 156.51, 140.76, 131.22, 131.00, 128.77, 127.91, 127.72, 71.79, 61.14, 53.38, 40.50, 28.22, 28.16, 25.45, 19.43, 19.21, 14.47 ppm. (The two diastereotopic Me carbons have the same chemical shift.)

HRMS: (ESI-TOF) calculated for $C_{19}H_{28}NO_4$ ([M+H]$^+$): 334.2013, found: 334.1998.

FTIR (thin film, cm^{-1}): 2943, 2872, 1719, 1695, 1422, 1259.

Optical rotation: $[\alpha]_D^{26}$ +80.2 (*c* 1.1, CHCl$_3$).

HPLC: Chiralcel OD-H, 2.5% IPA in hexanes, 20 min run, 1 mL/min.

Racemic Standard:

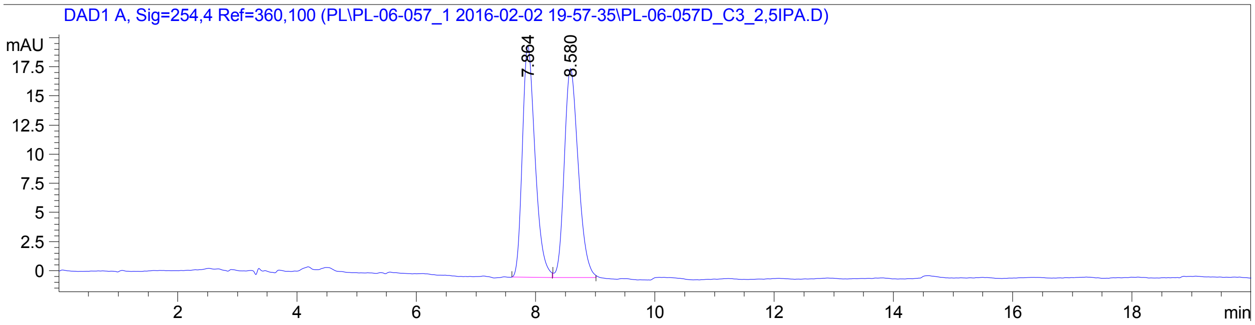

Peak #	RetTime [min]	Type	Width [min]	Area [mAU*s]	Height [mAU]	Area %
1	7.864	BB	0.2275	295.12170	19.84271	50.1068
2	8.580	BB	0.2510	293.86310	17.94593	49.8932
Totals :				588.98480	37.78863	

Enantioenriched:

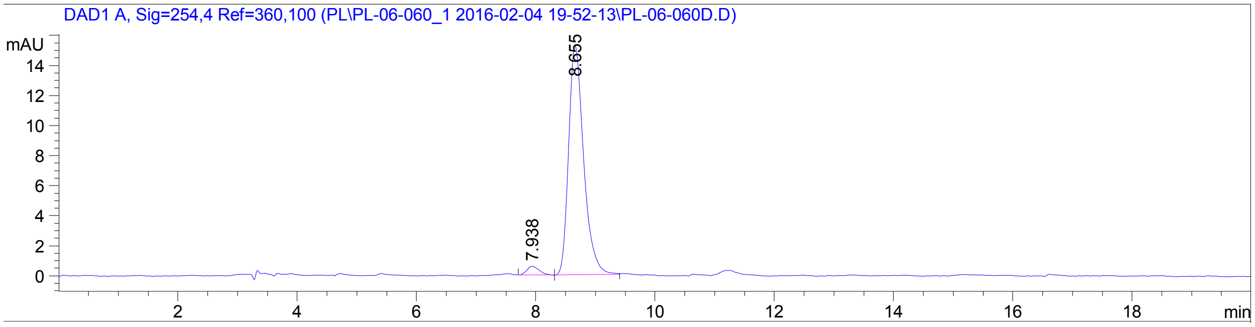

Peak #	RetTime [min]	Type	Width [min]	Area [mAU*s]	Height [mAU]	Area %
1	7.938	VV	0.2102	9.05619	6.06357e-1	3.2613
2	8.655	VB	0.2695	268.63400	15.25068	96.7387
Totals :				277.69019	15.85703	

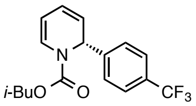

5e

(*R*)-Isobutyl 2-(4-(trifluoromethyl)phenyl)pyridine-1(2H)-carboxylate (5e): General procedure was followed with pyridine (40 mg, 0.50 mmol), providing **5e** (124 mg, 0.38 mmol, 76% yield, 70% ee) as a clear, colorless oil (run 1). Run 2 provided 64% yield, 70% ee.

^{1}H NMR (300 MHz, CDCl$_3$): δ 7.68–7.41 (m, 4H), 6.90 (dd, *J* = 64.7, 7.1 Hz, 1H), 6.07 (dd, *J* = 8.7, 5.9 Hz, 1H), 5.87 (dd, *J* = 41.8, 5.2 Hz, 1H), 5.73–5.56 (m, 1H), 5.29 (q, *J* = 8.4, 6.9 Hz, 1H), 4.07–3.80 (m, 2H), 2.12–1.73 (m, 1H), 1.12–0.47 (m, 6H) ppm.

^{13}C NMR (125 MHz, CDCl$_3$): δ [154.29, 153.81*], [146.60, 145.12*], 130.10 (q, *J* = 32.7 Hz), [127.46*, 126.43], [126.24*, 125.80], [125.68, 125.39*], 121.79, [121.59*, 121.17], [104.89*, 104.61], 72.74, [56.56, 55.38*], [28.03*, 27.92], 19.18, 19.00 ppm. (The CF$_3$ carbon was unable to be located by conventional ^{13}C NMR, but a ^{13}C,^{19}F-HMQC experiment showed the CF$_3$ signal at δ 116.91 ppm.)

^{19}F NMR (282 MHz, CDCl$_3$): δ –62.54 ppm.

HRMS: (ESI-TOF) calculated for $C_{17}H_{19}F_3NO_2$ ([M+H]$^+$): 326.1362, found: 326.1352.

FTIR (thin film, cm^{-1}): 1264, 1706, 1324, 1119, 1068.

Optical rotation: $[\alpha]_D^{26}$ +128.2 (*c* 1.1, CHCl$_3$).

HPLC: Chiralpak AD-H, 5% IPA in hexanes, 10 min run, 1 mL/min.

Racemic Standard:

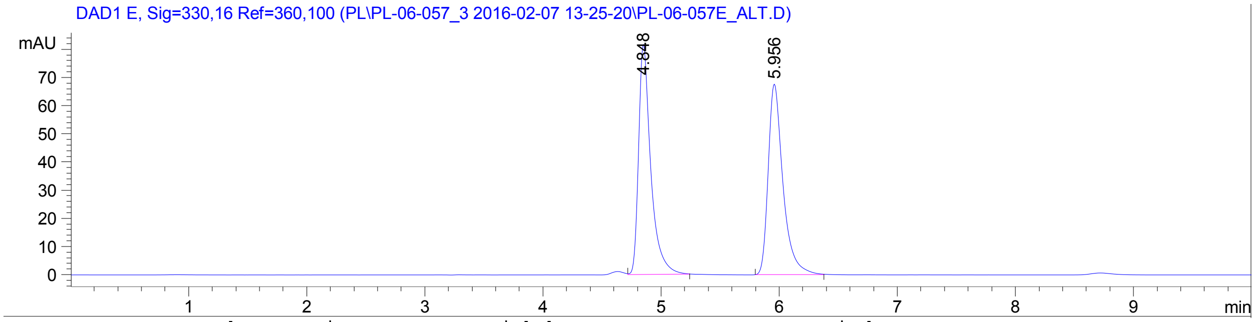

Peak #	RetTime [min]	Type	Width [min]	Area [mAU*s]	Height [mAU]	Area %
1	4.848	VB	0.1076	587.13165	81.90862	49.9204
2	5.956	BB	0.1295	589.00452	67.72691	50.0796

Totals : 1176.13617 149.63554

Enantioenriched:

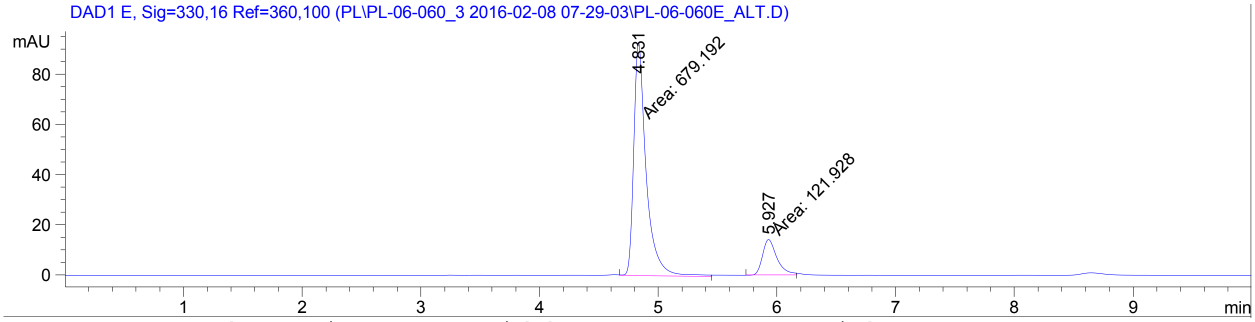

Peak #	RetTime [min]	Type	Width [min]	Area [mAU*s]	Height [mAU]	Area %
1	4.831	MM	0.1217	679.19208	93.05242	84.7803
2	5.927	MF	0.1425	121.92812	14.26353	15.2197

Totals : 801.12020 107.31595

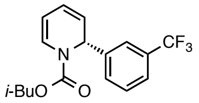

5f

(*R*)-Isobutyl 2-(3-(trifluoromethyl)phenyl)pyridine-1(2H)-carboxylate (5f): General procedure was followed with pyridine (39 mg, 0.50 mmol), providing **5f** (78 mg, 0.24 mmol, 48% yield, 65% ee) as a clear, colorless oil (run 1). Run 2 provided 54% yield, 65% ee.

^{1}H NMR (300 MHz, $CDCl_3$): δ 7.63 (dd, *J* = 19.4, 11.0 Hz, 2H), 7.53 (d, *J* = 7.7 Hz, 1H), 7.44 (t, *J* = 7.7 Hz, 1H), 6.88 (dd, *J* = 61.5, 6.8 Hz, 1H), 6.24–5.99 (m, 1H), 5.87 (dd, *J* = 46.7, 5.4 Hz, 1H), 5.74–5.59 (m, 1H), 5.31 (q, *J* = 7.5, 6.7 Hz, 1H), 4.08–3.74 (m, 2H), 2.13–1.72 (m, 1H), 0.96–0.78 (m, 6H) ppm.

^{13}C NMR (125 MHz, $CDCl_3$): δ [154.18, 153.70*], [143.41, 142.04*], 130.84 (q, *J* = 32.2 Hz), [130.60*, 129.62], [129.26, 129.98*], [129.21, 125.20*], 124.69, [123.92*, 122.88], 121.70, [121.60*, 121.13], [100.87*, 104.52], [77.64, 72.58*], [56.47, 55.10*], [27.90*, 27.76], 19.04*, 19.86 ppm. (The CF_3 carbon was unable to be located by conventional ^{13}C NMR, but a ^{13}C,^{19}F-HMQC experiment showed the CF_3 signal at δ [112.98, 110.95] ppm.)

^{19}F NMR (282 MHz, $CDCl_3$): δ [–62.53*, –62.64] ppm.

HRMS: (ESI-TOF) calculated for $C_{17}H_{19}F_3NO_2$ ($[M+H]^+$): 326.1362, found: 326.1357.

FTIR (thin film, cm^{-1}): 2966, 1707, 1328, 1165, 1125, 1074.

Optical rotation: $[\alpha]_D^{26}$ +155.9 (*c* 1.0, $CHCl_3$).

HPLC: Regis Whelk-O 1, 5% IPA in hexanes, 15 min run, 1 mL/min.

Racemic Standard:

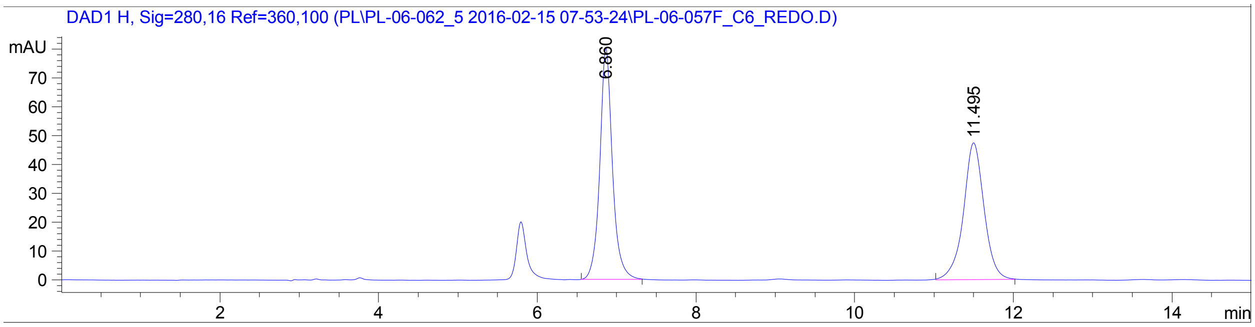

Peak #	RetTime [min]	Type	Width [min]	Area [mAU*s]	Height [mAU]	Area %
1	6.860	BB	0.1655	892.42267	80.38491	50.9121
2	11.495	BB	0.2713	860.44781	47.51920	49.0879
Totals :				1752.87048	127.90411	

Enantioenriched:

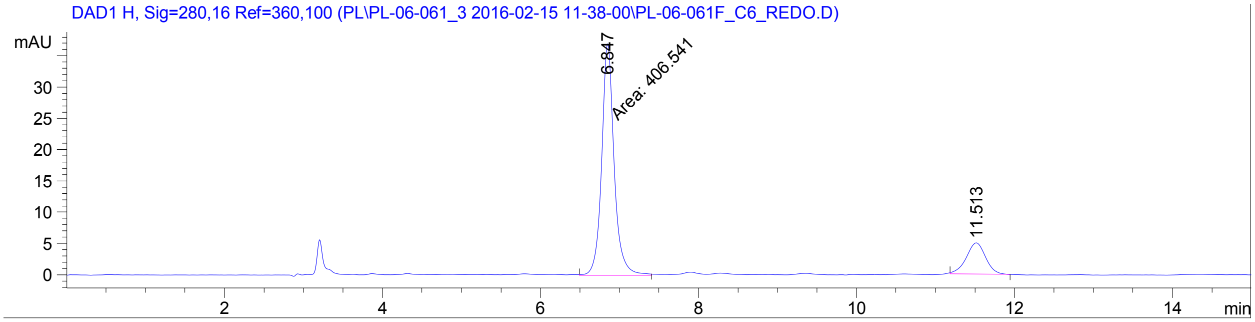

Peak #	RetTime [min]	Type	Width [min]	Area [mAU*s]	Height [mAU]	Area %
1	6.847	MM	0.1827	406.54095	37.07998	82.4662
2	11.513	BB	0.2607	86.43796	5.02345	17.5338
Totals :				492.97891	42.10342	

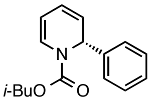

5g

(*R*)-Isobutyl 2-phenylpyridine-1(2H)-carboxylate (5g): General procedure was followed with pyridine (40 mg, 0.50 mmol), with the following modification: an increased catalyst/ligand loading of 20 mol% Ni(acac)$_2$ and 24 mol% **L3** was used. **5g** (62 mg, 0.24 mmol, 48% yield, 85% ee) was isolated as a clear, colorless oil (run 1). Run 2 provided 61% yield, 86% ee.

^{1}H NMR (300 MHz, $CDCl_3$): δ 7.58–7.27 (m, 5H), 6.88 (dd, *J* = 66.0, 6.3 Hz, 1H), 6.11–5.92 (m, 1H), 5.92–5.73 (m, 1H), 5.73–5.61 (m, 1H), 5.43–5.21 (m, 1H), 4.08–3.71 (m, 2H), 2.17–1.75 (m, 1H), 1.10–0.69 (m, 6H) ppm.

^{13}C NMR (125 MHz, $CDCl_3$): δ [154.61, 153.86*], [142.82, 141.33*], [128.73, 128.65*], [128.00*, 127.87], [127.26*, 126.04], [126.39, 125.34*], [122.80, 122.71*], [120.94*, 120.42], [104.95*, 104.59], [74.08, 72.52*], [56.98*, 55.72], [28.04, 27.93], 19.21, [19.08*, 19.06] ppm.

HRMS: (ESI-TOF) calculated for $C_{16}H_{20}NO_2$ ($[M+H]^+$): 258.1489, found: 258.1472.

FTIR (thin film, cm^{-1}): 3032, 2962, 2874, 1709, 1322, 1257, 1110.

Optical rotation: $[\alpha]_D^{26}$ +597.9 (*c* 1.0, $CHCl_3$).

HPLC: Chiralcel OD-H, 2.5% IPA to hexanes, 20 min run, 1 mL/min.

Racemic Standard:

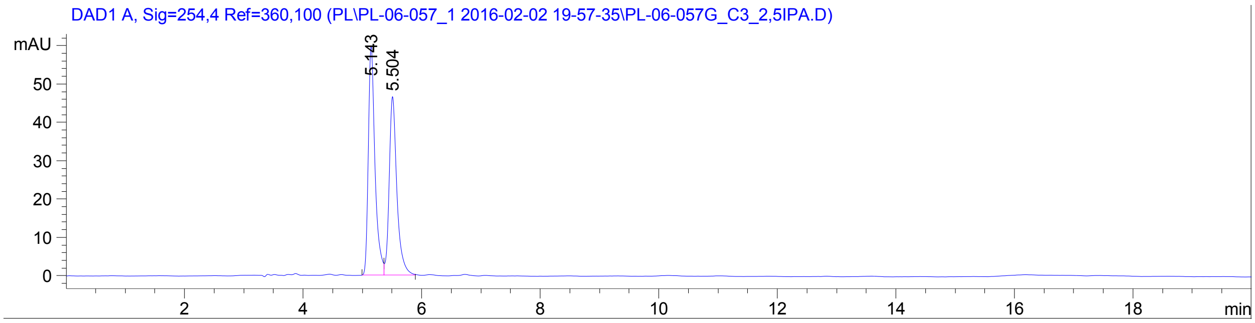

Peak #	RetTime [min]	Type	Width [min]	Area [mAU*s]	Height [mAU]	Area %
1	5.143	BV	0.1174	469.32428	59.93162	52.7012
2	5.504	VB	0.1354	421.21344	46.64292	47.2988

Totals : 890.53772 106.57454

Enantioenriched:

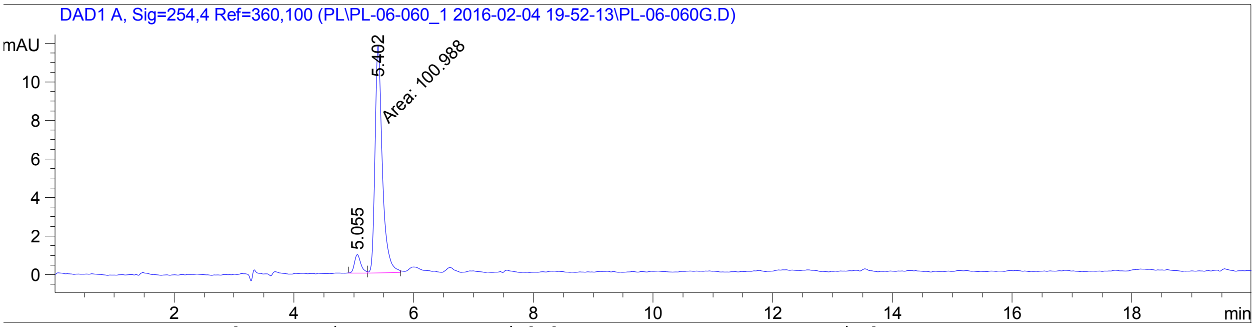

Peak #	RetTime [min]	Type	Width [min]	Area [mAU*s]	Height [mAU]	Area %
1	5.055	BV	0.1168	7.33036	9.63566e-1	6.7674
2	5.402	MF	0.1422	100.98844	11.83468	93.2326
Totals :				108.31880	12.79825	

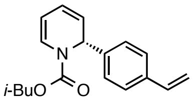

5h

(*R*)-Isobutyl 2-(4-vinylphenyl)pyridine-1(2H)-carboxylate (5h): General procedure was followed with pyridine (42 mg, 0.53 mmol), providing **5h** (90 mg, 0.32 mmol, 60% yield, 85% ee) as a clear, colorless oil (run 1). Run 2 provided 88% yield, 86% ee.

^{1}H NMR (300 MHz, $CDCl_3$): δ 7.43–7.30 (m, 4H), 6.87 (dd, *J* = 64.4, 7.7 Hz, 1H), 6.69 (dd, *J* = 17.6, 10.9 Hz, 1H), 6.15–5.92 (m, 1H), 5.91–5.59 (m, 3H), 5.34–5.25 (m, 1H), 5.22 (d, *J* = 11.3 Hz, 1H), 4.04–3.82 (m, 2H), 2.02–1.81 (m, 1H), 1.11–0.57 (m, 6H) ppm.

^{13}C NMR (125 MHz, $CDCl_3$): δ [140.86, 140.82*], [136.62*, 136.55], 131.84, [128.12, 127.92*], [127.52*, 126.67], [126.58, 126.53*], [126.31*, 125.32], [122.60, 122.52*], [121.04*, 120.57], 114.06, [104.93*, 104.67], [74.09, 72.54*], [56.65, 55.48*], [28.03*, 27.94], 19.21, 19.09 ppm.

HRMS: (ESI-TOF) calculated for $C_{18}H_{22}NO_2$ ($[M+H]^+$): 284.1645, found: 284.1630.

FTIR (thin film, cm^{-1}): 2962, 1708, 1403, 1383, 1323, 1258, 1114.

Optical rotation: $[\alpha]_D^{26}$ +1.3 (*c* 1.1, $CHCl_3$).

HPLC: Chiralcel OJ-H, 5% IPA in hexanes, 30 min run, 1 mL/min.

Racemic Standard:

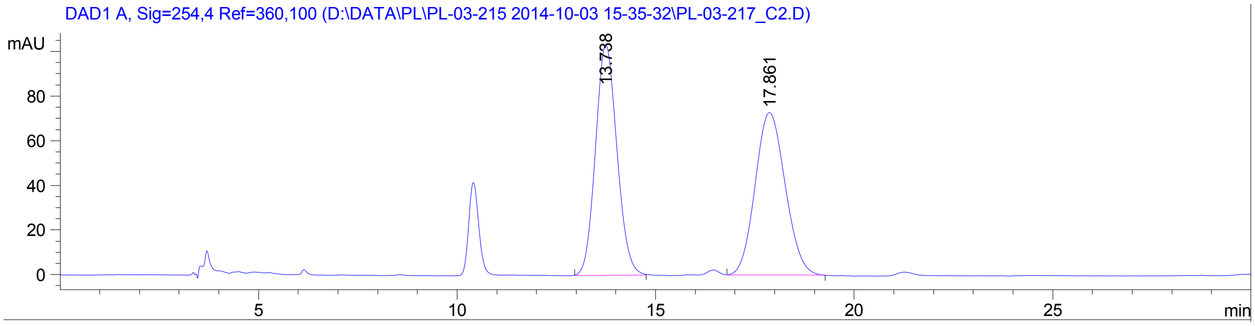

Peak #	RetTime [min]	Type	Width [min]	Area [mAU*s]	Height [mAU]	Area %
1	13.738	BB	0.5817	3878.08130	103.37643	50.1907
2	17.861	VB	0.8117	3848.60937	72.92225	49.8093
Totals :				7726.69067	176.29868	

Enantioenriched:

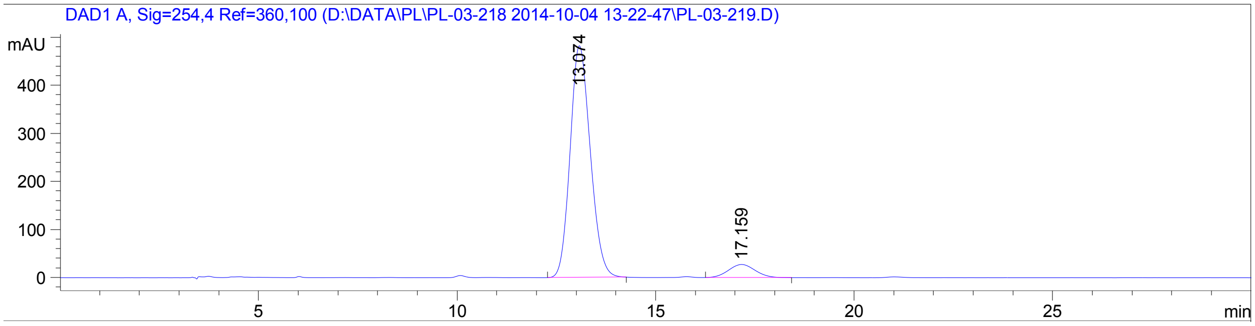

Peak #	RetTime [min]	Type	Width [min]	Area [mAU*s]	Height [mAU]	Area %
1	13.074	BB	0.5470	1.70628e4	482.03925	93.0671
2	17.159	BB	0.7350	1271.07141	27.21154	6.9329
Totals :				1.83338e4	509.25078	

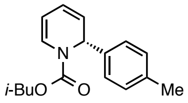

5i

(*R*)-Isobutyl 2-(p-tolyl)pyridine-1(2H)-carboxylate (5i): General procedure was followed with pyridine (41 mg, 0.52 mmol), providing **5i** (85 mg, 0.31 mmol, 60% yield, 76% ee) as a clear, colorless oil (run 1). Run 2 provided 59% yield, 76% ee.

^{1}H NMR (300 MHz, $CDCl_3$): δ 7.35 (d, *J* = 7.6 Hz, 1H), 7.26–7.23 (m, 1H), 7.12 (d, *J* = 7.5 Hz, 2H), 6.85 (dd, *J* = 62.4, 7.4 Hz, 1H), 5.99 (ddd, *J* = 27.2, 9.8, 4.8 Hz, 1H), 5.90–5.57 (m, 2H), 5.37–5.17 (m, 1H), 4.01–3.82 (m, 2H), 2.32 (s, 3H), 2.05–1.80 (m, 1H), 1.00–0.75 (m, 6H) ppm.

^{13}C NMR (125 MHz, $CDCl_3$): δ 139.73, [138.58, 138.40*], [137.78*, 137.60], 129.34, [127.30*, 126.14], [126.32, 125.27*], [122.95, 122.84*], [120.83*, 120.38], [104.94*, 104.75], [72.52, 72.47*], [56.56, 56.44*], [28.03*, 27.93], 21.30, [19.22*, 19.15], [19.08*, 19.07] ppm.

HRMS: (ESI-TOF) calculated for $C_{17}H_{22}NO_2$ ($[M+H]^+$): 272.1645, found: 272.1621.

FTIR (thin film, cm^{-1}): 2962, 2875, 1710, 1401, 1383, 1322, 1257, 1111.

Optical rotation: $[\alpha]_D^{26}$ +82.4 (*c* 1.0, $CHCl_3$).

HPLC: Chiralcel OJ-H, 5% IPA in hexanes, 15 min run, 1 mL/min.

Racemic Standard:

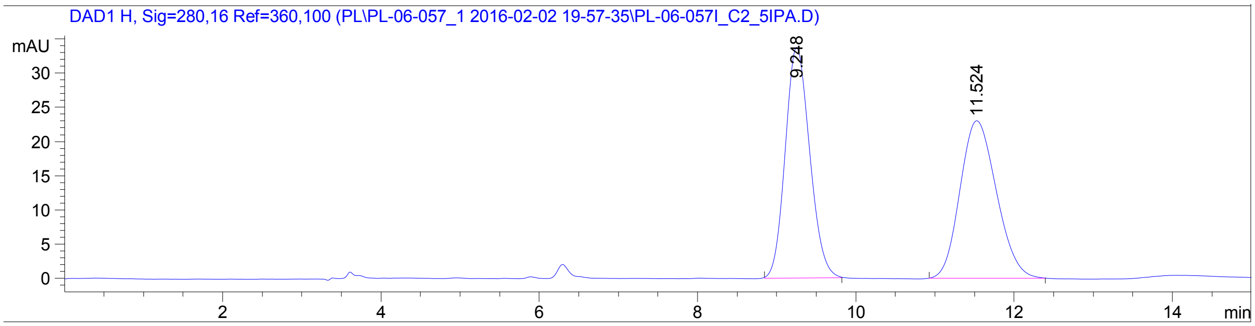

Peak #	RetTime [min]	Type	Width [min]	Area [mAU*s]	Height [mAU]	Area %
1	9.248	BB	0.3353	726.83502	33.74718	49.5531
2	11.524	BB	0.5043	739.94647	23.06237	50.4469
Totals :				1466.78149	56.80955	

Enantioenriched:

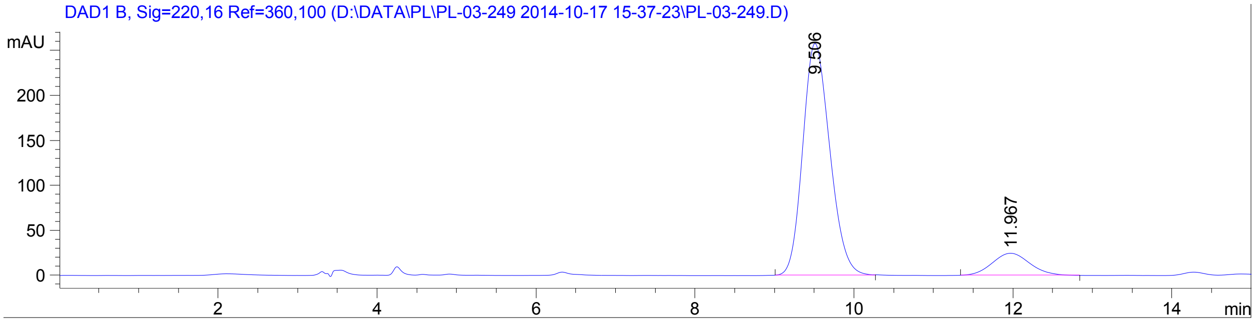

Peak #	RetTime [min]	Type	Width [min]	Area [mAU*s]	Height [mAU]	Area %
1	9.506	BB	0.3683	6108.27930	257.92078	88.1860
2	11.967	BB	0.5087	818.30481	24.68896	11.8140
Totals :				6926.58411	282.60974	

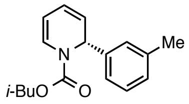

5j

(*R*)-Isobutyl 2-(m-tolyl)pyridine-1(2H)-carboxylate (5j): General procedure was followed with pyridine (40 mg, 0.51 mmol), providing **5j** (42 mg, 0.15 mmol, 30% yield, 95% ee) as a clear, colorless oil (run 1). Run 2 provided 52% yield, 94% ee.

^{1}H NMR (300 MHz, $CDCl_3$): δ 7.25–7.12 (m, 3H), 7.07 (d, *J* = 6.7 Hz, 1H), 6.88 (dd, *J* = 60.8, 7.8 Hz, 1H), 6.11–5.88 (m, 1H), 5.88–5.52 (m, 2H), 5.28 (q, *J* = 6.5, 6.0 Hz, 1H), 4.07–3.82 (m, 2H), 2.33 (s, 3H), 2.08–1.77 (m, 1H), 1.04–0.60 (m, 6H) ppm.

^{13}C NMR (125 MHz, MeOD): δ [154.65, 153.84*], [142.76, 141.40*], [139.81, 138.27*], [129.28*, 126.89], [128.78, 128.58*], [127.80*, 126.74], [126.38, 125.36*], [124.21*, 123.12], 122.88, [120.74*, 120.31], [104.91*, 104.58], [74.07, 72.48*], [56.98, 55.78*], [28.03, 27.92*], [21.65*, 21.45] [19.21*, 19.04], [19.07, 19.06*] ppm.

HRMS: (ESI-TOF) calculated for $C_{17}H_{22}NO_2$ ($[M+H]^+$): 272.1645, found: 272.1637.

FTIR (thin film, cm^{-1}): 2962, 2875, 1711, 1400, 1383, 1320, 1257, 1110.

Optical rotation: $[\alpha]_D^{26}$ +449.9 (*c* 1.0, $CHCl_3$).

HPLC: Chiralcel OJ-H, 5% IPA in hexanes, 15 min run, 1 mL/min.

Racemic Standard:

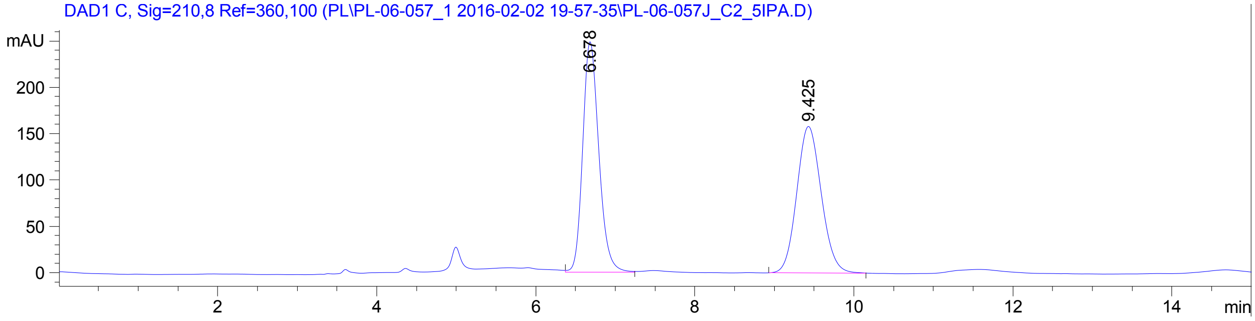

Peak #	RetTime [min]	Type	Width [min]	Area [mAU*s]	Height [mAU]	Area %
1	6.678	VB	0.2170	3522.23853	249.02133	50.9486
2	9.425	BB	0.3320	3391.07520	158.24355	49.0514

Totals : 6913.31372 407.26488

Enantioenriched:

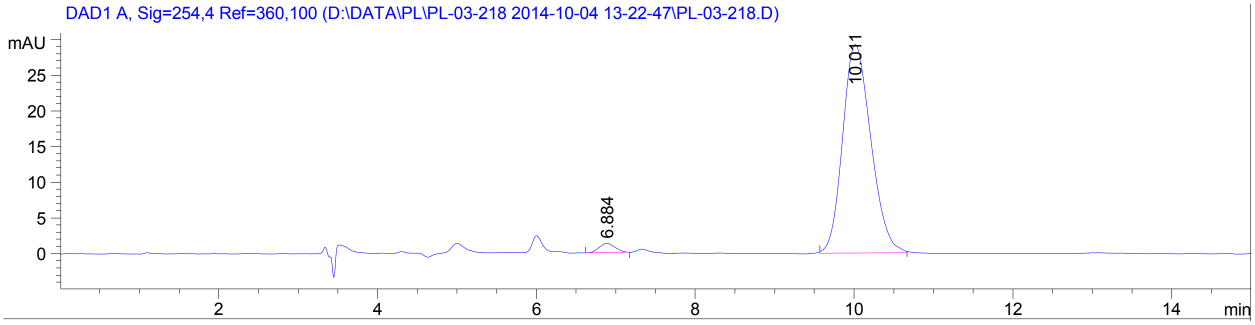

Peak #	RetTime [min]	Type	Width [min]	Area [mAU*s]	Height [mAU]	Area %
1	6.884	BV	0.2248	18.58300	1.31533	2.5046
2	10.011	BB	0.3847	723.35883	29.23611	97.4954

Totals : 741.94183 30.55144

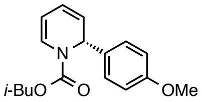

5k

(*R*)-Isobutyl 2-(4-methoxyphenyl)pyridine-1(2H)-carboxylate (5k): General procedure was followed with pyridine (39 mg, 0.49 mmol), providing **5k** (100 mg, 0.35 mmol, 71% yield, 91% ee) as a clear, colorless oil (run 1). Run 2 provided 65% yield, 91% ee.

^{1}H NMR (300 MHz, $CDCl_3$): δ 7.35 (dd, *J* = 30.9, 7.9 Hz, 2H), 6.99–6.65 (m, 3H), 6.28–5.92 (m, 1H), 5.91–5.52 (m, 2H), 5.41–5.16 (m, 1H), 4.00–3.86 (m, 2H), 3.78 (s, 3H), 2.18–1.78 (m, 1H), 1.00–0.75 (m, 6H) ppm.

^{13}C NMR (125 MHz, $CDCl_3$): δ [159.40*, 159.34], 153.88, [134.84, 133.43*], [128.90*, 127.70], [126.19, 125.12*], [122.99, 122.83*], [120.86*, 120.36], [113.95, 113.90*], [104.93*, 104.76], [72.52, 72.45*], [56.15*, 55.02], 55.38, [28.02, 28.00*], 19.21 ppm. (The two diastereotopic Me carbons have the same chemical shift.)

HRMS: (ESI-TOF) calculated for $C_{17}H_{22}NO_3$ ($[M+H]^+$): 288.1594, found: 288.1563.

FTIR (thin film, cm^{-1}): 2960, 2875, 2837, 1704, 1510, 1321, 1245.

Optical rotation: $[\alpha]_D^{26}$ +402.3 (*c* 1.1, $CHCl_3$).

HPLC: Chiralcel OJ-H, 5% IPA in hexanes, 20 min run, 1 mL/min.

Racemic Standard:

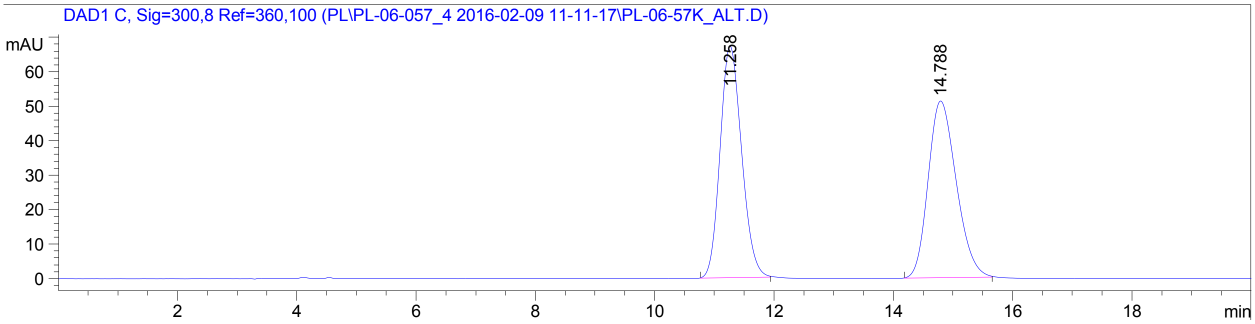

Peak #	RetTime [min]	Type	Width [min]	Area [mAU*s]	Height [mAU]	Area %
1	11.258	BB	0.3927	1686.92004	67.23863	50.0912
2	14.788	BB	0.5061	1680.77661	51.31408	49.9088
Totals :				3367.69666	118.55271	

Enantioenriched:

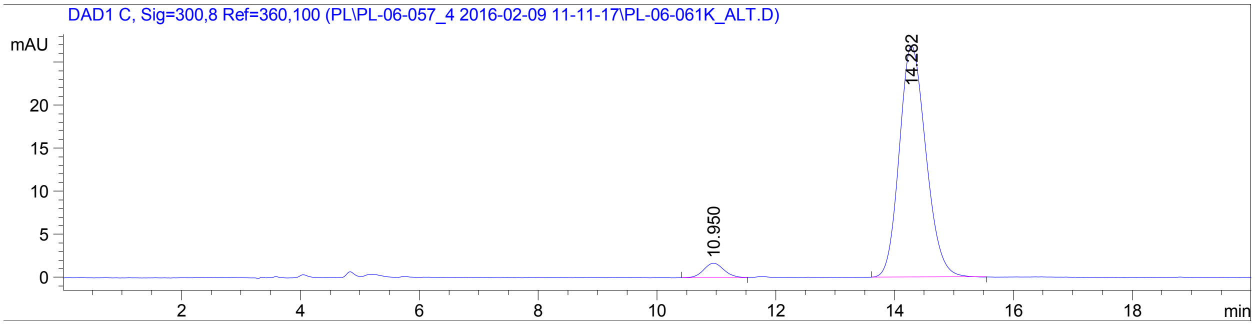

Peak #	RetTime [min]	Type	Width [min]	Area [mAU*s]	Height [mAU]	Area %
1	10.950	BV	0.3692	40.38046	1.68747	4.6026
2	14.282	BB	0.4793	836.95392	26.87584	95.3974
Totals :				877.33437	28.56331	

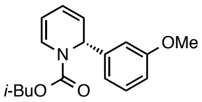

5l

(*R*)-Isobutyl 2-(3-methoxyphenyl)pyridine-1(2H)-carboxylate (5l): General procedure was followed with pyridine (39 mg, 0.49 mmol), providing **5l** (96 mg, 0.34 mmol, 69% yield, 95% ee) as a clear, colorless oil (run 1). Run 2 provided 71% yield, 96% ee.

^{1}H NMR (300 MHz, $CDCl_3$): δ 7.22 (d, *J* = 7.8 Hz, 1H), 7.10–6.86 (m, 2H), 6.79 (t, *J* = 8.7 Hz, 2H), 6.13–5.90 (m, 1H), 5.91–5.57 (m, 2H), 5.36–5.19 (m, 1H), 4.00–3.87 (m, 2H), 3.78 (s, 3H), 2.05–1.77 (m, 1H), 1.11–0.59 (m, 6H) ppm.

^{13}C NMR (125 MHz, $CDCl_3$): δ [159.96, 159.87*], [154.59, 153.86], [144.44, 143.04*], [129.74, 129.65*], [126.41, 125.39*], [122.68, 122.61*], [120.96*, 120.50], [119.44*, 118.33], [113.23*, 112.97], [112.86*, 111.87], [104.94*, 104.59], [72.63, 72.54*], [56.92, 55.66*] 55.33, [28.04*, 27.95], [19.22*, 19.19], 19.07 ppm.

HRMS: (ESI-TOF) calculated for $C_{17}H_{22}NO_3$ ($[M+H]^+$): 288.1594, found: 288.1581.

FTIR (thin film, cm^{-1}): 2961, 2836, 1706, 1315, 1259.

Optical rotation: $[\alpha]_D^{26}$ +556.0 (*c* 0.95, $CHCl_3$).

HPLC: Chiralcel OJ-H, 5% IPA in hexanes, 20 min run, 1 mL/min.

Racemic Standard:

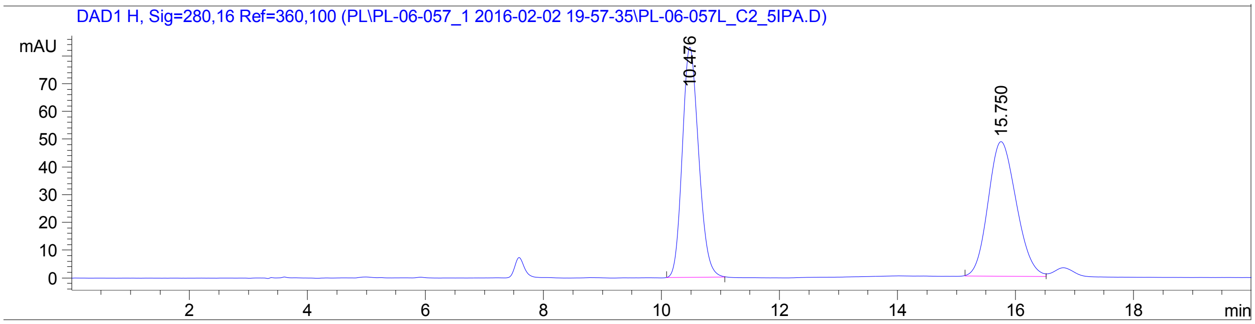

Peak #	RetTime [min]	Type	Width [min]	Area [mAU*s]	Height [mAU]	Area %
1	10.476	BB	0.2994	1604.45691	83.02817	49.8758
2	15.750	BV	0.5152	1612.44568	48.57981	50.1242

Totals : 3216.90259 131.60798

Enantioenriched:

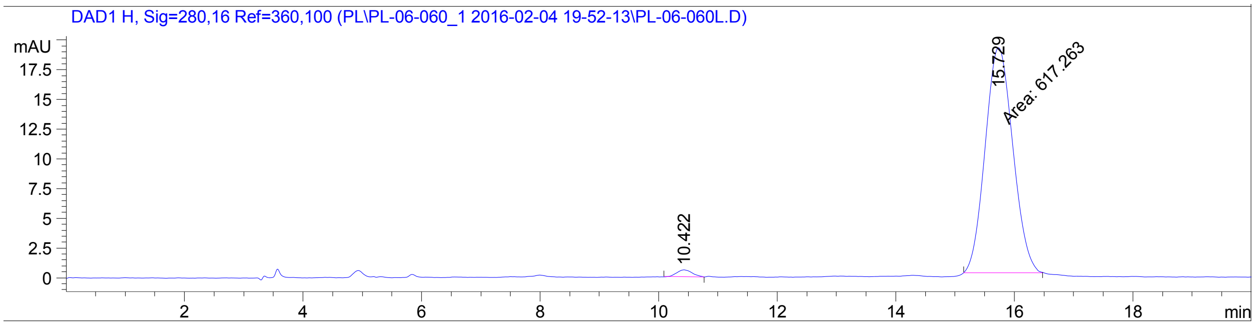

Peak #	RetTime [min]	Type	Width [min]	Area [mAU*s]	Height [mAU]	Area %
1	10.422	BV	0.2898	11.07084	5.98464e-1	1.7619
2	15.729	MM	0.5423	617.26318	18.97049	98.2381

Totals : 628.33402 19.56895

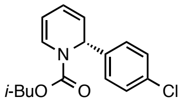

5m

(*R*)-Isobutyl 2-(4-chlorophenyl)pyridine-1(2H)-carboxylate (5m): General procedure was followed with pyridine (39 mg, 0.49 mmol), providing **5m** (79 mg, 0.27 mmol, 55% yield, 80% ee) as a clear, colorless oil (run 1). Run 2 provided 61% yield, 81% ee.

^{1}H NMR (500 MHz, $CDCl_3$): δ 7.50–7.27 (m, 4H), 6.85 (dd, *J* = 109.7, 7.6 Hz, 1H), 6.14–5.91 (m, 1H), 5.79 (dd, *J* = 77.4, 5.2 Hz, 1H), 5.64 (dq, *J* = 11.8, 6.2 Hz, 1H), 5.28 (dt, *J* = 20.0, 6.6 Hz, 1H), 4.00–3.79 (m, 2H), 1.91 (dtd, *J* = 40.2, 12.9, 6.5 Hz, 1H), 0.98–0.75 (m, 6H) ppm.

^{13}C NMR (125 MHz, $CDCl_3$): δ [154.40, 153.81*], [141.19, 139.66*], [133.82*, 133.64], 128.83, [128.78*, 127.55], [126.29, 125.23*], [122.23, 122.11*], [121.35*, 120.84], [104.90*, 104.65], 72.62, [56.27, 55.04*], [28.02*, 27.95], 19.19, 19.10 ppm.

HRMS: (ESI-TOF) calculated for $C_{16}H_{19}ClNO_2$ ($[M+H]^+$): 292.1099, found: 292.1099.

FTIR (thin film, cm^{-1}): 2963, 2875, 1708, 1401, 1383, 1323, 1258.

Optical rotation: $[\alpha]_D^{26}$ +493.5 (*c* 1.2, $CHCl_3$).

HPLC: Chiralcel OJ-H, 2.5% IPA in hexanes, 15 min run, 1 mL/min.

Racemic Standard:

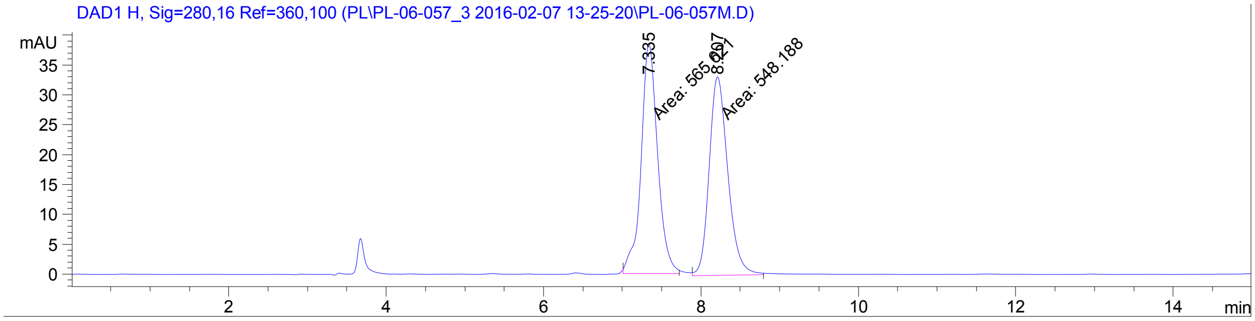

Peak #	RetTime [min]	Type	Width [min]	Area [mAU*s]	Height [mAU]	Area %
1	7.335	MF	0.2447	565.62067	38.52024	50.7826
2	8.207	MM	0.2750	548.18817	33.22602	49.2174
Totals :				1113.80884	71.74626	

Enantioenriched:

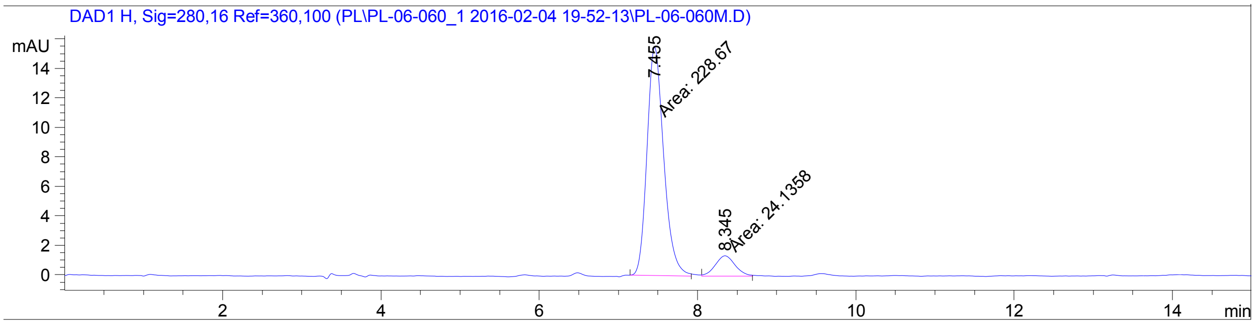

Peak #	RetTime [min]	Type	Width [min]	Area [mAU*s]	Height [mAU]	Area %
1	7.455	MM	0.2457	228.66983	15.50897	90.4528
2	8.345	MM	0.2914	24.13583	1.38050	9.5472
Totals :				252.80566	16.88947	

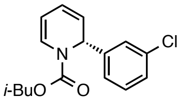

5n

(*R*)-Isobutyl 2-(3-chlorophenyl)pyridine-1(2H)-carboxylate (5n): General procedure was followed with pyridine (39 mg, 0.49 mmol), providing **5n** (115 mg, 0.394 mmol, 80 yield, 94% ee) as a clear, colorless oil (run 1). Run 2 provided 75% yield, 92% ee.

^{1}H NMR (300 MHz, $CDCl_3$): δ 7.73–7.46 (m, 1H), 7.46–7.29 (m, 2H), 7.24 (m, 1H), 6.87 (dd, *J* = 60.4, 6.5 Hz, 1H), 6.02 (ddd, *J* = 25.8, 8.7, 6.5 Hz, 1H), 5.91–5.56 (m, 2H), 5.36–5.21 (m, 1H), 3.92 (q, *J* = 7.2, 6.3 Hz, 2H), 2.07–1.81 (m, 1H), 1.02–0.62 (m, 6H) ppm.

^{13}C NMR (125 MHz, MeOD): δ [154.32, 153.76*], [144.58, 143.24*], 134.50, [130.04, 129.88*], [128.13, 127.99*], [127.36*, 126.36], [126.30, 125.41*], [125.29*, 124.25], 121.88, [121.44*, 121.00], [104.93*, 104.59], [72.68, 72.63*], [56.47, 55.21*], [28.00*, 27.89], 19.17, 19.02 ppm.

HRMS: (ESI-TOF) calculated for $C_{16}H_{19}ClNO_2$ ($[M+H]^+$): 292.1099, found: 292.1103.

FTIR (thin film, cm^{-1}): 2961, 1708, 1402, 1383, 1315.

Optical rotation: $[\alpha]_D^{26}$ +264.9 (*c* 0.97, $CHCl_3$).

HPLC: Chiralpak AS-H, 1% IPA in hexanes, 10 min run, 1 mL/min.

Racemic Standard:

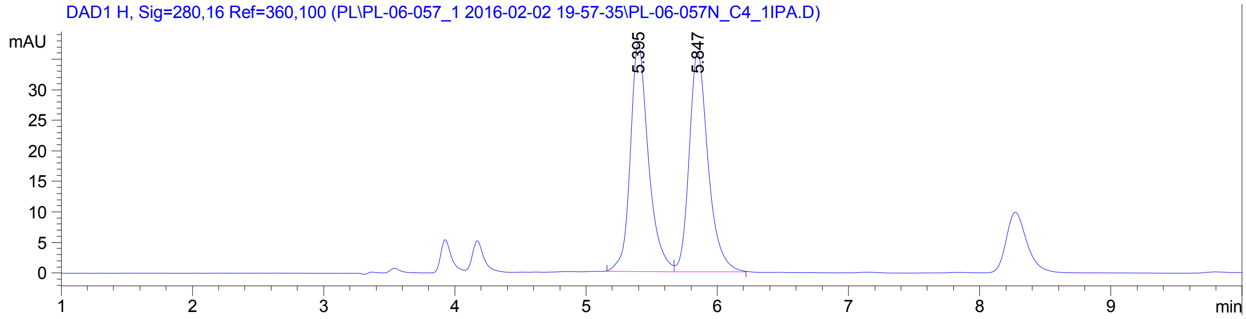

Peak #	RetTime [min]	Type	Width [min]	Area [mAU*s]	Height [mAU]	Area %
1	5.395	BV	0.1443	360.93839	37.49607	49.9769
2	5.847	VB	0.1509	361.27167	36.05886	50.0231

Totals : 722.21005 73.55493

Enantioenriched:

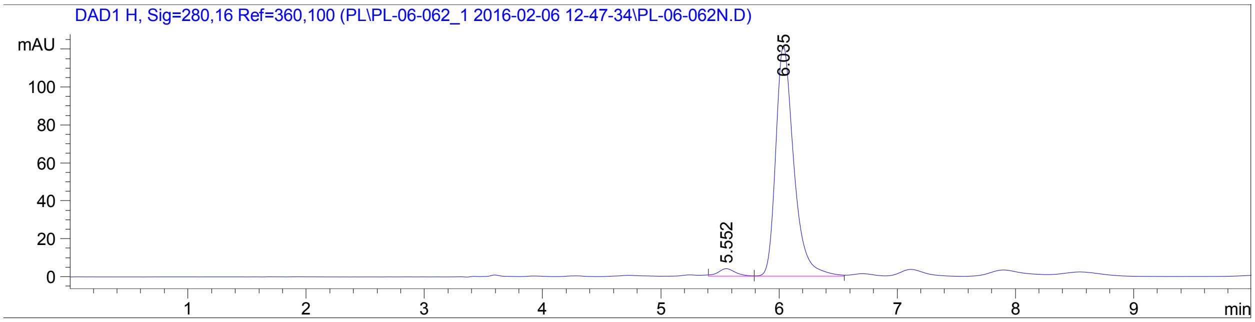

Peak #	RetTime [min]	Type	Width [min]	Area [mAU*s]	Height [mAU]	Area %
1	5.552	BV	0.1533	40.36875	3.94809	3.0252
2	6.035	VB	0.1623	1294.03906	121.47241	96.9748

Totals : 1334.40781 125.42050

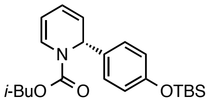

5o

(*R*)-Isobutyl 2-(4-((tert-butyldimethylsilyl)oxy)phenyl)pyridine-1(2H)-carboxylate (5o): General procedure was followed with pyridine (39 mg, 0.50 mmol), with the following modification: following column chromatography, **5o** was further purified on a prep plate (10% EtOAc in hexanes) to the product from biaryl. **5o** (86 mg, 0.22 mmol, 45% yield, 93% ee) was isolated as a clear, colorless oil (run 1). Run 2 provided 52% yield, 94% ee.

^{1}H NMR (300 MHz, $CDCl_3$): δ 7.31 (d, *J* = 6.1 Hz, 1H), 7.22 (d, *J* = 5.5 Hz, 1H), 7.01–6.67 (m, 3H), 6.15–5.86 (m, 1H), 5.86–5.53 (m, 2H), 5.36–5.10 (m, 1H), 4.00–3.82 (m, 2H), 2.01–1.82 (m, 1H), 1.01–0.76 (m, 15H), 0.17 (s, 6H) ppm.

^{13}C NMR (125 MHz, $CDCl_3$): δ [155.52*, 155.41], 146.87, [135.59, 133.86*], [128.84*, 127.55], [126.19, 125.14*], [123.13, 122.93*], [120.82, 120.15*], 119.90, [104.99*, 104.60], [72.49, 72.43*], [56.40, 55.01*], [28.04*, 27.97], 25.80, 19.21, 19.16, 18.31, –4.27 ppm.

HRMS: (ESI-TOF) calculated for $C_{22}H_{34}NO_3Si$ ($[M+H]^+$): 388.2302, found: 388.2294.

FTIR (thin film, cm^{-1}): 2961, 1738, 1717, 1508, 1371, 1217.

Optical rotation: $[\alpha]_D^{26}$ +8.2 (*c* 1.1, $CHCl_3$).

HPLC: Chiralpak AS-H, 1% IPA in hexanes, 30 min run, 1 mL/min.

Racemic Standard:

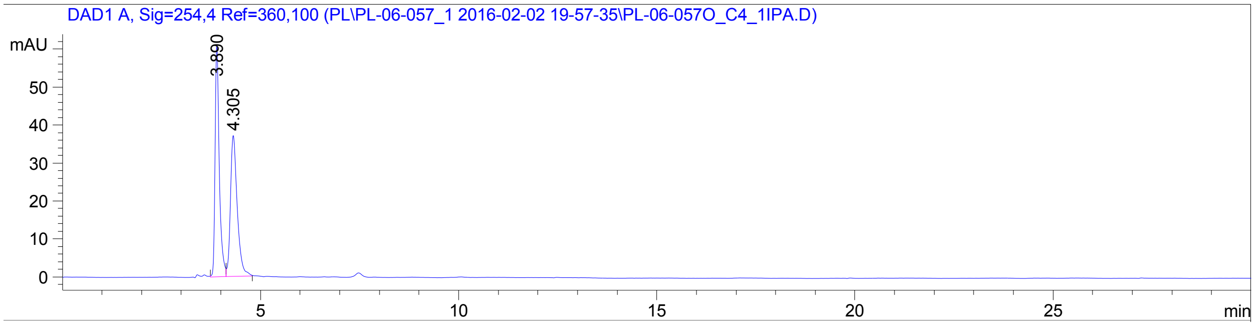

Peak #	RetTime [min]	Type	Width [min]	Area [mAU*s]	Height [mAU]	Area %
1	3.890	BV	0.1106	452.46188	60.98144	50.5285
2	4.305	VB	0.1792	442.99774	37.12979	49.4715
Totals :				895.45963	98.11123	

Enantioenriched:

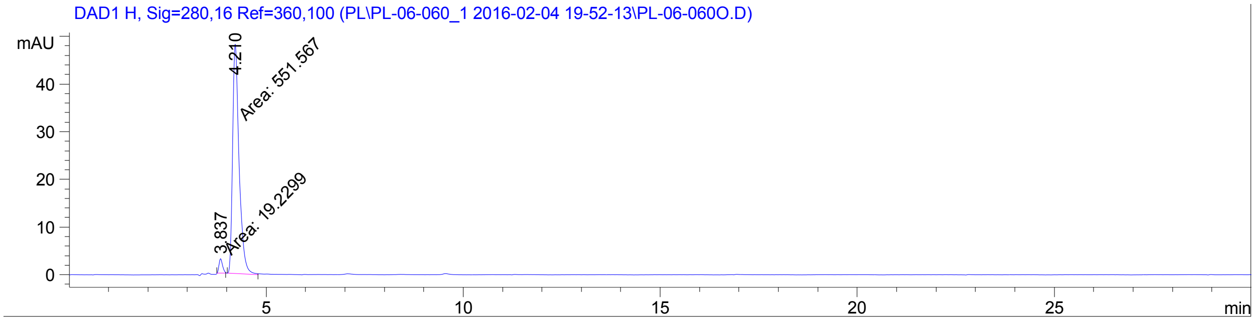

Peak #	RetTime [min]	Type	Width [min]	Area [mAU*s]	Height [mAU]	Area %
1	3.837	MM	0.1051	19.22992	3.04859	3.3690
2	4.210	MM	0.1908	551.56659	48.16879	96.6310

Totals : 570.79651 51.21738

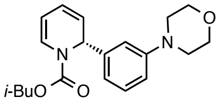

5p

(*R*)-Isobutyl 2-(3-morpholinophenyl)pyridine-1(2H)-carboxylate (5p): General procedure was followed with pyridine (39 mg, 0.49 mmol), providing **5p** (113 mg, 0.330 mmol, 74% yield, 94% ee) as a clear, colorless oil (run 1). Run 2 provided 63% yield, 94% ee.

^{1}H NMR (500 MHz, $CDCl_3$): δ 7.23–7.10 (m, 1H), 7.07–6.62 (m, 4H), 6.10–5.89 (m, 1H), 5.76–5.61 (m, 2H), 5.37–5.21 (m, 1H), 4.03–3.66 (m, 6H), 3.14 (br. s, 4H), 2.02–1.80 (m, 1H), 1.02–0.77 (m, 6H) ppm.

^{13}C NMR (125 MHz, $CDCl_3$): δ [154.63, 153.86*], [151.75, 151.55*], [143.99, 142.32*], [129.50, 129.44*], [126.43, 125.39*], [122.96, 122.77*], [120.93*, 120.32], [118.96*, 117.75], 115.12, [114.75*, 113.25], [104.93*, 104.55], 72.50, [67.07*, 66.91], [57.28, 55.95*], [49.44, 49.38*], [28.03*, 27.99], [19.22*, 19.13], [19.07*, 19.04] ppm.

HRMS: (ESI-TOF) calculated for $C_{20}H_{27}N_2O_3$ ($[M+H]^+$): 343.2016, found: 343.2011.

FTIR (thin film, cm^{-1}): 2961, 1708, 1323, 1255, 1239, 1118.

Optical rotation: $[\alpha]_D^{26}$ +270.3 (*c* 0.27, $CHCl_3$).

HPLC: Chiralpak AS-H, 5% IPA in hexanes, 20 min run, 1 mL/min.

Racemic Standard:

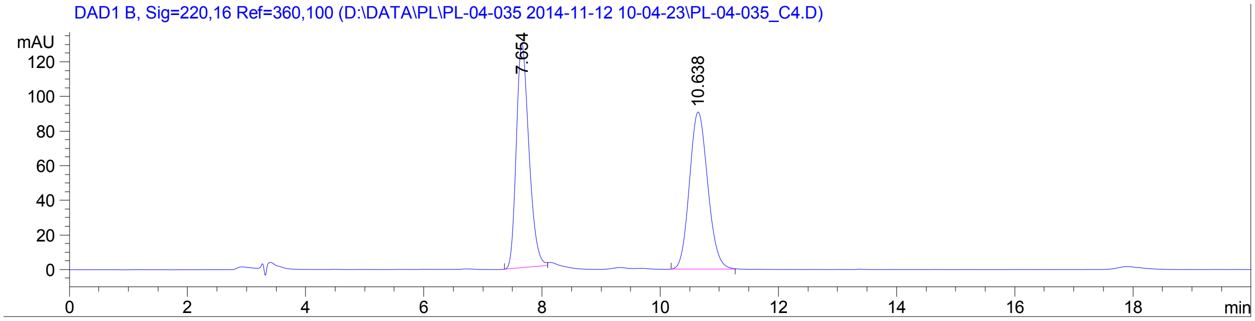

Peak #	RetTime [min]	Type	Width [min]	Area [mAU*s]	Height [mAU]	Area %
1	7.654	BB	0.2273	1922.91711	129.48032	49.5952
2	10.638	BB	0.3351	1954.30640	90.80654	50.4048
Totals :				3877.22351	220.28686	

Enantioenriched:

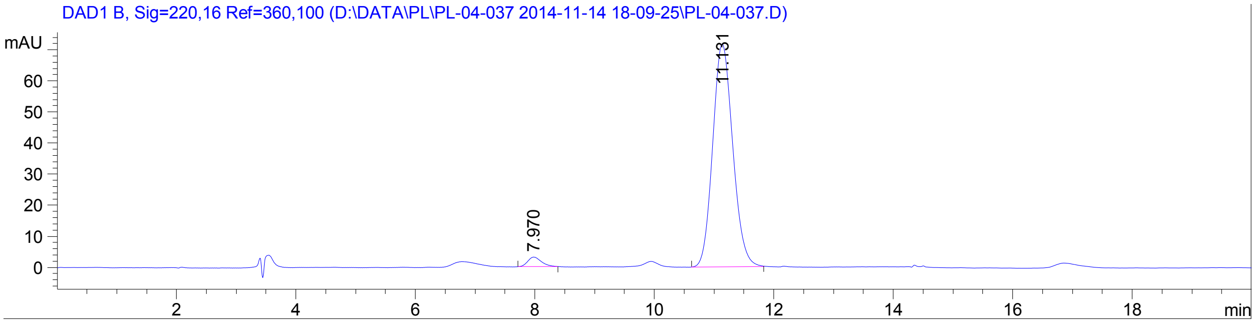

Peak #	RetTime [min]	Type	Width [min]	Area [mAU*s]	Height [mAU]	Area %
1	7.970	BB	0.1962	51.47332	3.15735	2.9863
2	11.131	BB	0.3480	1672.18152	71.71393	97.0137
Totals :				1723.65484	74.87127	

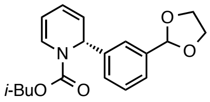

5q

(*R*)-Isobutyl 2-(3-(1,3-dioxolan-2-yl)phenyl)pyridine-1(2H)-carboxylate (5q): General procedure was followed with pyridine (40 mg, 0.51 mmol), with the following modification: 1.3 equiv. *i*-BuOCOCl (0.63 mmol, 81 µL) was used instead of 3 equiv. Compound **5q** (99 mg, 0.27 mmol, 53% yield, 92% ee) was isolated as a clear, colorless oil (run 1). Run 2 provided 41% yield, 92% ee.

^{1}H NMR (300 MHz, $CDCl_3$): δ 7.72–7.29 (m, 4H), 6.86 (dd, *J* = 65.4, 9.3 Hz, 1H), 6.17–5.74 (m, 3H), 5.74–5.59 (m, 1H), 5.35–5.06 (m, 1H), 4.20–3.97 (m, 4H), 3.96–3.81 (m, 2H), 2.07–1.74 (m, 1H), 1.07–0.70 (m, 6H) ppm.

^{13}C NMR (125 MHz, $CDCl_3$): δ 153.82, [142.81, 141.38*], [138.38, 138.24*], [128.85, 128.74*], [128.31*, 127.11], [126.34, 125.34*], [126.02*, 125.66], 124.40, [122.62, 122.53*], [121.16*, 120.65], [105.00*, 104.69], [103.83*, 103.73], [72.59, 72.52*], [65.45, 65.42*], [56.85, 55.53*], [28.02, 27.90], [19.21*, 19.13], [19.09*, 19.05] ppm.

HRMS: (ESI-TOF) calculated for $C_{19}H_{24}NO_4$ ($[M+H]^+$): 330.1700, found: 330.1690.

FTIR (thin film, cm^{-1}): 2961, 2879, 1708, 1322, 1258, 1106.

Optical rotation: $[\alpha]_D^{26}$ +1015.1 (*c* 0.56, $CHCl_3$).

HPLC: Chiralpak AS-H, 5% IPA in hexanes, 20 min run, 1 mL/min.

Racemic Standard:

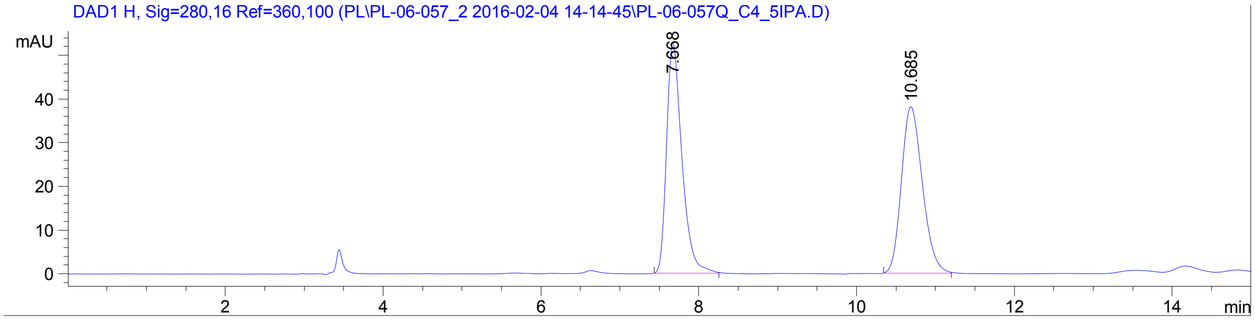

Peak #	RetTime [min]	Type	Width [min]	Area [mAU*s]	Height [mAU]	Area %
1	7.668	BB	0.2061	716.23511	52.85177	50.4087
2	10.685	BB	0.2857	704.62024	38.10961	49.5913
Totals :				1420.85535	90.96138	

Enantioenriched:

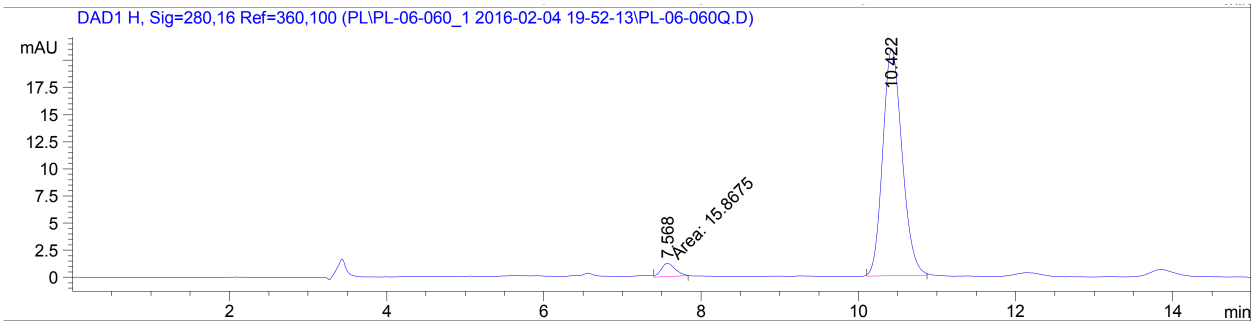

Peak #	RetTime [min]	Type	Width [min]	Area [mAU*s]	Height [mAU]	Area %
1	7.568	MM	0.2117	15.86748	1.24936	4.2354
2	10.422	BB	0.2660	358.77386	20.92804	95.7646

Totals : 374.64135 22.17740

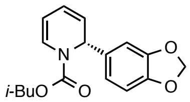

5r

(*R*)-Isobutyl 2-(benzo[d][1,3]dioxol-5-yl)pyridine-1(2H)-carboxylate (5r): General procedure was followed with pyridine (40 mg, 0.51 mmol), providing **5r** (75 mg, 0.25 mmol, 49% yield, 94% ee) as a clear, colorless oil (run 1). Run 2 provided 55% yield, 94% ee.

^{1}H NMR (300 MHz, $CDCl_3$): δ 7.04–6.88 (m, 2H), 6.74 (d, *J* = 7.8 Hz, 2H), 6.10–5.95 (m, 1H), 5.92 (s, 2H), 5.87–5.56 (m, 2H), 5.34–5.17 (m, 1H), 4.03–3.77 (m, 2H), 2.05–1.83 (m, 1H), 1.03–0.73 (m, 6H) ppm.

^{13}C NMR (125 MHz, $CDCl_3$): δ [154.56, 153.87*], [147.95, 147.87*], [147.35*, 147.27], [136.65, 135.30*], [126.24, 125.19*], [122.82, 122.73*], [120.92*, 120.52], 119.65, [108.20*, 108.11], 107.14, [104.89*, 104.69], 101.14, [72.59, 72.53*], [56.53, 55.40*], [28.02*, 28.00], 19.21, 19.15 ppm.

HRMS: (ESI-TOF) calculated for $C_{17}H_{20}NO_4$ ($[M+H]^+$): 302.1387, found: 302.1372.

FTIR (thin film, cm^{-1}): 2962, 2896, 1707, 1488, 1325, 1250, 1236, 1039.

Optical rotation: $[\alpha]_D^{26}$ +499.0 (*c* 0.65, $CHCl_3$).

HPLC: Chiralcel OJ-H, 5% IPA in hexanes, 15 min run, 1 mL/min.

Racemic Standard:

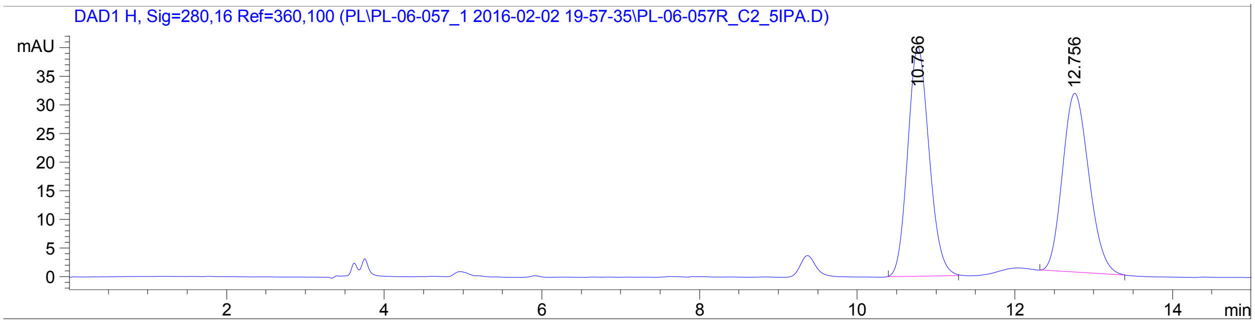

Peak #	RetTime [min]	Type	Width [min]	Area [mAU*s]	Height [mAU]	Area %
1	10.766	BB	0.2931	759.41602	40.07277	51.0012
2	12.756	BB	0.3642	729.60065	31.27133	48.9988
Totals :				1489.01666	71.34410	

Enantioenriched:

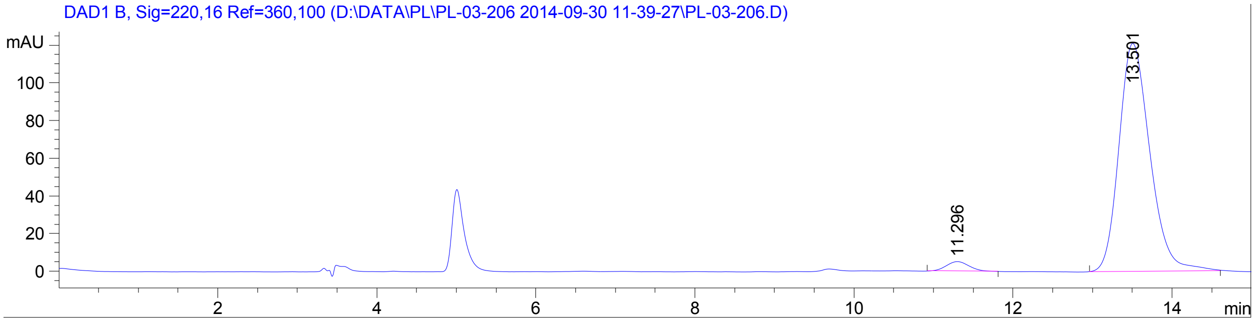

Peak #	RetTime [min]	Type	Width [min]	Area [mAU*s]	Height [mAU]	Area %
1	11.296	BB	0.3060	104.41981	5.11933	3.0835
2	13.501	BB	0.4182	3281.97119	121.08748	96.9165
Totals :				3386.39100	126.20681	

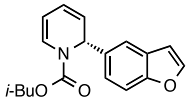

5s

(*R*)-Isobutyl 2-(benzofuran-5-yl)pyridine-1(2H)-carboxylate (5s): General procedure was followed with pyridine (40 mg, 0.50 mmol), with the following modification: an increased catalyst/ligand loading of 20 mol% $Ni(acac)_2$ and 24 mol% **L3** was used. **5s** (79 mg, 0.27 mmol, 53% yield, 89% ee) was isolated as a clear, colorless oil (run 1). Run 2 provided 60% yield, 89% ee.

^{1}H NMR (300 MHz, $CDCl_3$): δ 7.65 (d, *J* = 28.0 Hz, 2H), 7.53–7.28 (m, 2H), 7.08–6.69 (m, 2H), 6.16–5.80 (m, 2H), 5.76–5.65 (m, 1H), 5.43–5.22 (m, 1H), 4.09–3.84 (m, 2H), 2.04–1.81 (m, 1H), 1.07–0.72 (m, 6H) ppm.

^{13}C NMR (125 MHz, $CDCl_3$): δ [154.76, 154.64], 153.91, [145.56, 145.41*], [137.61, 136.04*], [127.67, 127.56*], [126.32, 125.23*], [124.09*, 122.83], [123.28, 123.16*], [120.80, 120.25*], 118.90, 111.47, [106.94*, 106.79], [104.92*, 104.65], [72.55, 72.50*], [56.94, 55.77*], [28.02*, 27.96] 19.21, 19.11 ppm.

HRMS: (ESI-TOF) calculated for $C_{18}H_{20}NO_3$ ($[M+H]^+$): 298.1438, found: 298.1433.

FTIR (thin film, cm^{-1}): 2962, 2874, 1706, 1324, 1259, 1109.

Optical rotation: $[\alpha]_D^{26}$ +587.4 (*c* 1.2, $CHCl_3$).

HPLC: Chiralpak AS-H, 5% IPA in hexanes, 15 min run, 1 mL/min.

Racemic Standard:

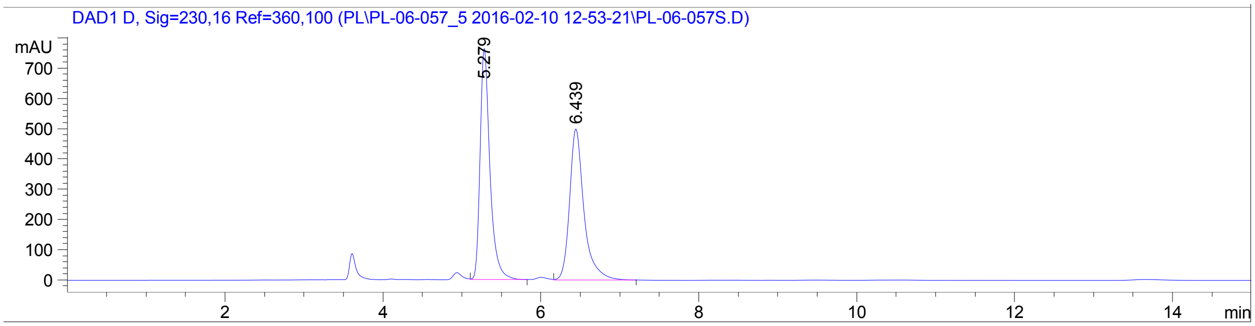

Peak #	RetTime [min]	Type	Width [min]	Area [mAU*s]	Height [mAU]	Area %
1	5.279	VB	0.1296	6660.96436	765.11633	51.2374
2	6.439	VB	0.1882	6339.22510	499.19629	48.7626
Totals :				1.30002e4	1264.31262	

Enantioenriched:

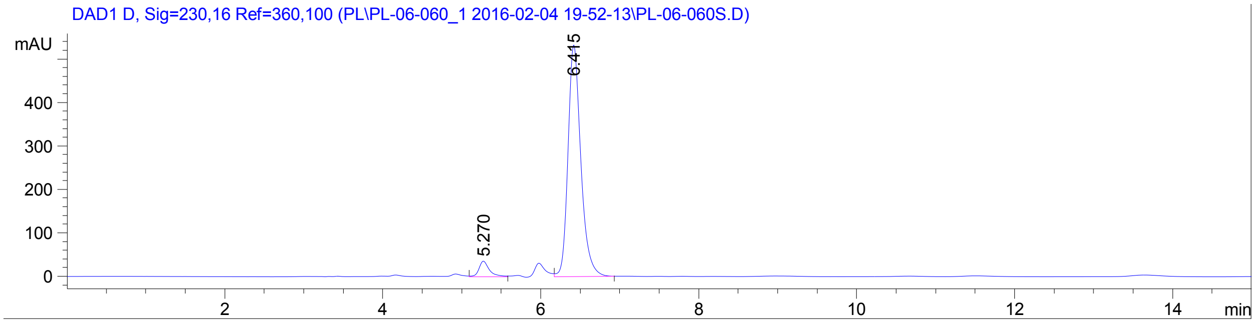

Peak #	RetTime [min]	Type	Width [min]	Area [mAU*s]	Height [mAU]	Area %
1	5.270	VB	0.1406	353.18173	36.59095	5.5430
2	6.415	VB	0.1717	6018.49023	533.09760	94.4570

Totals : 6371.67197 569.68855

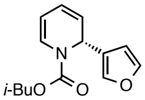

5t

(*R*)-Isobutyl 2-(furan-3-yl)pyridine-1(2H)-carboxylate (5t): General procedure was followed with pyridine (42 mg, 0.53 mmol), with the following modification: an increased catalyst/ligand loading of 20 mol% Ni(acac)$_2$ and 24 mol% **L3** was used. **5t** (64 mg, 0.26 mmol, 49% yield, 96% ee) was isolated as a clear, colorless oil (run 1). Run 2 provided 64% yield, 95% ee.

^{1}H NMR (300 MHz, CDCl$_3$): δ 7.43–7.30 (m, 2H), 6.86–6.58 (m, 1H), 6.46–6.33 (m, 1H), 6.05 (dd, *J* = 9.4, 5.5 Hz, 1H), 5.91–5.72 (m, 1H), 5.66 (dd, *J* = 9.7, 5.4 Hz, 1H), 5.41–5.23 (m, 1H), 3.97 (t, *J* = 7.7 Hz, 2H), 2.08–1.84 (m, 1H), 0.95 (d, *J* = 6.7 Hz, 6H) ppm.

^{13}C NMR (125 MHz, CDCl$_3$): δ 153.71, [143.37, 143.20*], [141.11*, 140.18], [125.67, 124.73*], 124.82, [122.05, 121.89*], [120.95*, 120.86], [110.25*, 109.63], [105.70, 105.33*], [72.60, 72.51*], [47.81, 47.11*], 28.02, 19.26, [19.22*, 19.08] ppm.

HRMS: (ESI-TOF) calculated for $C_{14}H_{18}NO_3$ ($[M+H]^+$): 248.1281, found: 248.1270.

FTIR (thin film, cm^{-1}): 2962, 2876, 1706, 1326, 1257, 1110, 1014.

Optical rotation: $[\alpha]_D^{26}$ +404.3 (*c* 1.1, CHCl$_3$).

HPLC: Chiralcel OJ-H, 2.5% IPA in hexanes, 10 min run, 1 mL/min.

Racemic Standard:

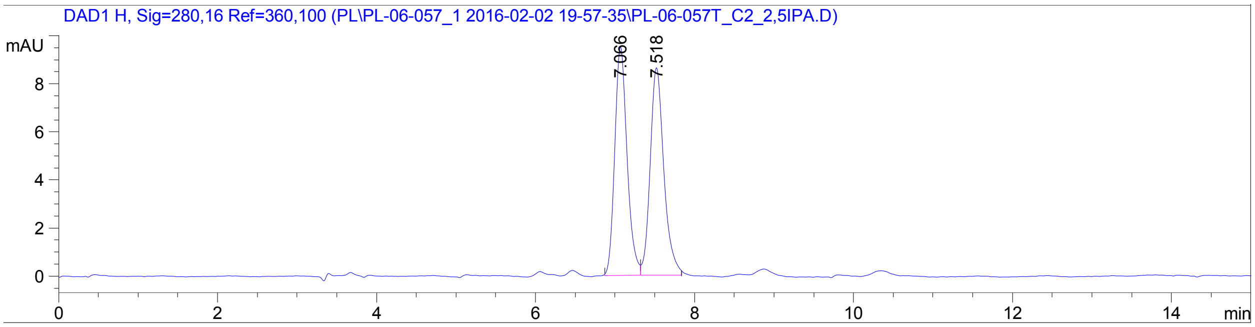

Peak #	RetTime [min]	Type	Width [min]	Area [mAU*s]	Height [mAU]	Area %
1	7.066	BV	0.1661	103.04958	9.53345	49.8869
2	7.518	VB	0.1817	103.51673	8.64455	50.1131
Totals :				206.56631	18.17800	

Enantioenriched:

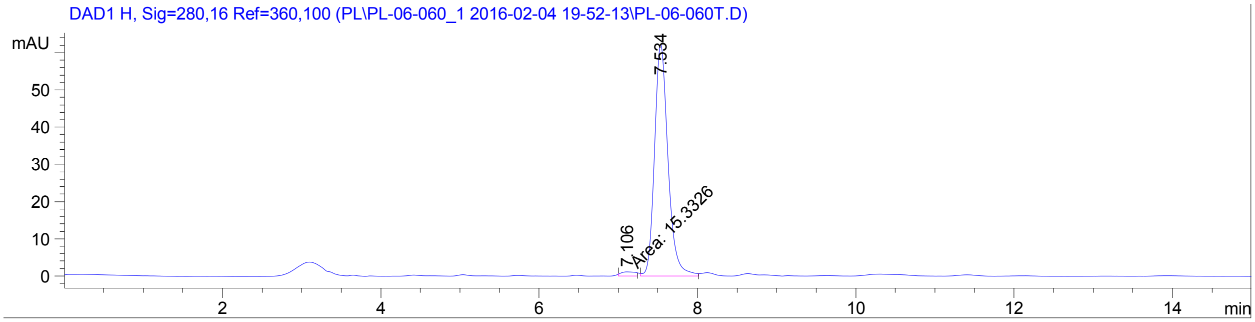

Peak #	RetTime [min]	Type	Width [min]	Area [mAU*s]	Height [mAU]	Area %
1	7.106	MF	0.2142	15.33261	1.19317	2.0267
2	7.534	VV	0.1870	741.19159	62.20142	97.9733
Totals :				756.52419	63.39459	

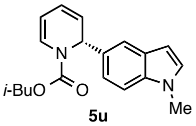

5u

(*R*)-Isobutyl 2-(1-methyl-1H-indol-5-yl)pyridine-1(2H)-carboxylate (5u): General procedure was followed with pyridine (40 mg, 0.50 mmol), providing **5u** (68 mg, 0.22 mmol, 43% yield, 90% ee) as a clear, colorless oil (run 1). Run 2 provided 49% yield, 90% ee.

^{1}H NMR (300 MHz, $CDCl_3$): δ 7.77–7.59 (m, 1H), 7.58–7.35 (m, 1H), 7.11–6.99 (m, 1H), 6.99–6.69 (m, 1H), 6.59–6.37 (m, 1H), 6.19–5.80 (m, 2H), 5.80–5.65 (m, 1H), 5.41–5.19 (m, 1H), 4.17–3.81 (m, 2H), 3.76 (s, 3H), 2.04–1.83 (m, 1H), 1.00–0.71 (m, 6H) ppm.

^{13}C NMR (125 MHz, $CDCl_3$): δ [154.83, 153.92*], [136.55*, 136.48], 133.75, 132.33, [129.34, 129.29*], [126.26, 125.16*], [123.79, 123.62*], 121.63, [120.42*, 119.88], [120.06*, 118.76], [109.36, 109.24*], [104.97*, 104.79], [101.41*, 101.23], [72.46, 72.33*], [57.20, 56.09*], 33.00, [28.00*, 27.97], 19.23, 19.17 ppm.

HRMS: (ESI-TOF) calculated for $C_{19}H_{23}N_2O_2$ ($[M+H]^+$): 311.1754, found: 311.1738.

FTIR (thin film, cm^{-1}): 2960, 1703, 1320, 1252, 1108.

Optical rotation: $[\alpha]_D^{26}$ +529.0 (*c* 1.1, $CHCl_3$).

HPLC: Chiralpak AD-H, 2.5% IPA in hexanes, 20 min run, 1 mL/min.

Racemic Standard:

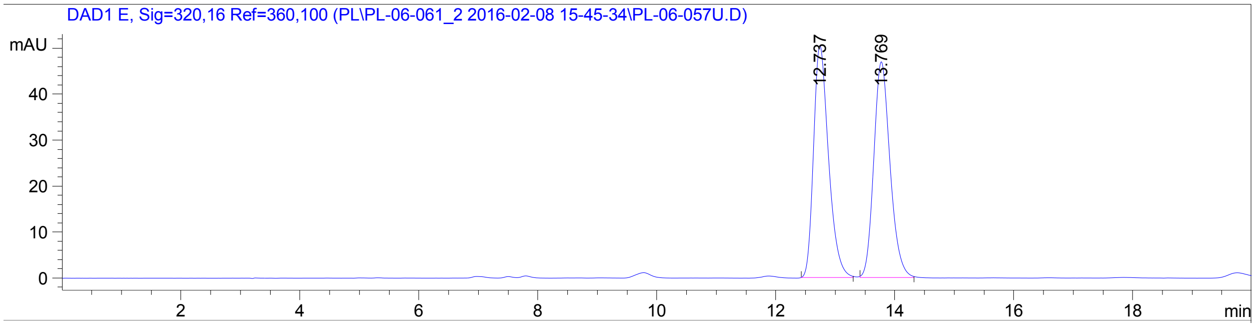

Peak #	RetTime [min]	Type	Width [min]	Area [mAU*s]	Height [mAU]	Area %
1	12.737	BB	0.2690	887.98657	50.54144	49.8844
2	13.769	BB	0.2915	892.10291	46.98204	50.1156
Totals :				1780.08948	97.52348	

Enantioenriched:

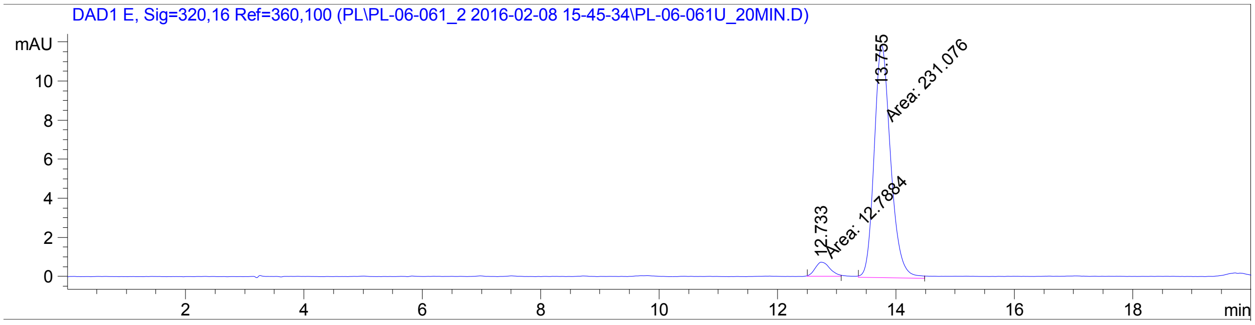

Peak #	RetTime [min]	Type	Width [min]	Area [mAU*s]	Height [mAU]	Area %
1	12.733	MF	0.2919	12.78836	7.30267e-1	5.2440
2	13.755	MM	0.3234	231.07634	11.90786	94.7560
Totals :				243.86470	12.63813	

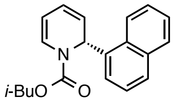

5v

(*R*)-Isobutyl 2-(naphthalen-1-yl)pyridine-1(2H)-carboxylate (5v): General procedure was followed with pyridine (39 mg, 0.50 mmol) with the following modification: an increased catalyst/ligand loading of 20 mol% Ni(acac)$_2$ and 24 mol% **L3** was used. **5v** (38 mg, 0.12 mmol, 25% yield, 37% ee) was isolated as a clear, colorless oil (run 1). Run 2 provided 17% yield, 37% ee.

^{1}H NMR (300 MHz, CDCl$_3$): δ 8.38–7.99 (m, 1H), 7.86 (d, *J* = 8.7 Hz, 1H), 7.77 (d, *J* = 8.2 Hz, 1H), 7.63 (d, *J* = 7.2 Hz, 1H), 7.58–7.31 (m, 3H), 7.24–6.96 (m, 1H), 6.61 (d, *J* = 20.2 Hz, 1H), 5.82 (br. s, 2H), 5.33–5.22 (m, 1H), 4.03–3.63 (m, 2H), 2.21–1.38 (m, 1H), 1.10–0.78 (m, 4H), 0.50–0.35 (m, 2H) ppm.

^{13}C NMR (125 MHz, CDCl$_3$): δ [154.73, 153.75], [140.18*, 138.94], 134.01, 133.87, [129.03*, 128.94], [128.35, 128.07*], [126.72, 125.97], 126.50, 126.15, [125.89, 125.71*], [123.42, 122.66], [123.22, 122.44*], 122.93, 119.82, [103.92, 103.25*], [72.56, 72.45*], [54.45*, 53.80], [28.04, 27.63], 19.21, 18.51 ppm.

HRMS: (ESI-TOF) calculated for $C_{20}H_{22}NO_2$ ([M+H]$^+$): 308.1645, found: 308.1616.

FTIR (thin film, cm^{-1}): 2944, 2871, 1694, 1414, 1237.

Optical rotation: $[\alpha]_D^{26}$ –109.1 (*c* 0.35, CHCl$_3$).

HPLC: Chiralpak AS-H, 5% IPA in hexanes, 10 min run, 1 mL/min.

Racemic Standard:

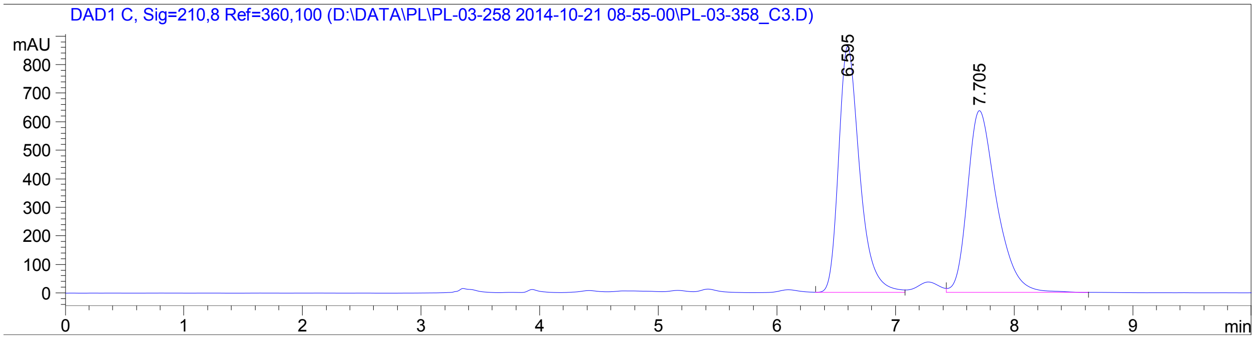

Peak #	RetTime [min]	Type	Width [min]	Area [mAU*s]	Height [mAU]	Area %
1	6.595	VV	0.1885	1.06864e4	863.02271	49.4787
2	7.705	VB	0.2576	1.09115e4	637.87769	50.5213
Totals :				2.15979e4	1500.90039	

Enantioenriched:

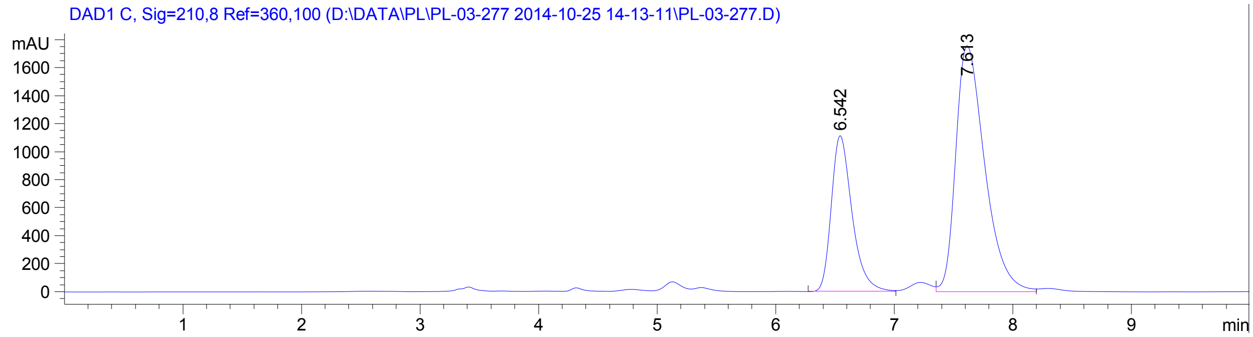

Peak #	RetTime [min]	Type	Width [min]	Area [mAU*s]	Height [mAU]	Area %
1	6.542	VV	0.1864	1.35907e4	1113.33264	31.2826
2	7.613	VV	0.2604	2.98543e4	1755.84302	68.7174
Totals :				4.34450e4	2869.17566	

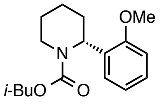

5w

(*R*)-Isobutyl 2-(2-methoxyphenyl)piperidine-1-carboxylate (5w): General procedure was followed with pyridine (40 mg, 0.50 mmol), with the following modifications: an increased catalyst/ligand loading of 20 mol% Ni(acac)$_2$ and 24 mol% **L3** was used. The dihydropyridine product was found to be difficult to

separate from the biaryl, so the dihydropyridine was reduced to piperidine **5w**, which was readily separated from the biaryl, with H_2 (100 psi) and Pd/C (5 mol%) in MeOH. **5w** (64 mg, 0.22 mmol, 43% yield, 74% ee) was isolated as a clear, colorless oil (run 1). Run 2 provided 39% yield, 74% ee.

^{1}H NMR (300 MHz, $CDCl_3$): δ 7.23–7.08 (m, 2H), 6.93–6.81 (m, 2H), 5.49 (dd, *J* = 6.0, 3.6 Hz, 1H), 4.31–4.14 (m, 1H), 3.87–3.71 (m, 4H), 3.48 (q, *J* = 7.1 Hz, 1H), 3.36–3.24 (m, 1H), 2.14–1.97 (m, 1H), 1.96–1.82 (m, 1H), 1.81–1.67 (m, 2H), 1.47–1.34 (m, 2H), 1.21 (app. t, *J* = 7.0 Hz, 1H), 0.75 (app. t, *J* = 6.8 Hz, 6H) ppm.

^{13}C NMR (125 MHz, $CDCl_3$): δ 156.75, 156.59, 131.62, 127.59, 126.58, 120.23, 110.64, 71.40, 55.36, 50.86, 41.88, 28.82, 28.09. 24.85, 19.15, 18.99, 18.94 ppm.

HRMS: (ESI-TOF) calculated for $C_{17}H_{26}NO_3$ ($[M+H]^+$): 292.1907, found: 292.1873.

FTIR (thin film, cm^{-1}): 2937, 2869, 1695, 1414, 1237.

Optical rotation: $[\alpha]_D^{26}$ −29.1 (*c* 1.1, $CHCl_3$).

HPLC: Chiralcel OJ-H, 5% IPA in hexanes, 15 min run, 1 mL/min.

Racemic Standard:

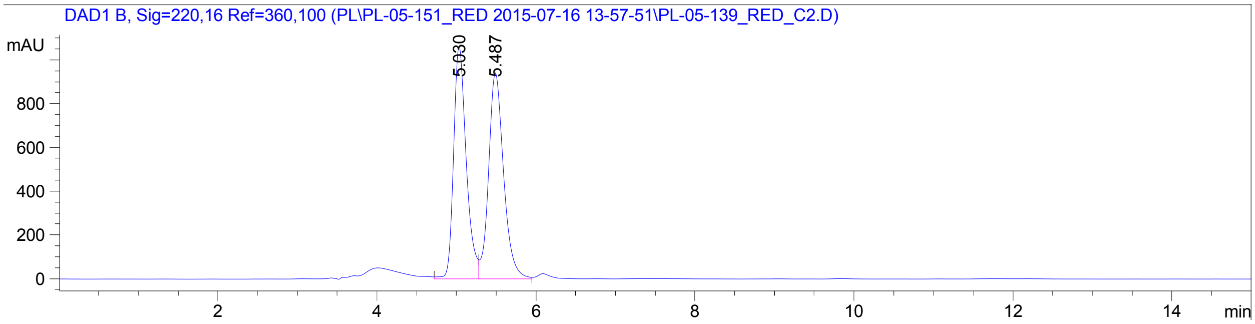

Peak #	RetTime [min]	Type	Width [min]	Area [mAU*s]	Height [mAU]	Area %
1	5.030	VV	0.1691	1.17763e4	1064.23950	48.8330
2	5.487	VV	0.1988	1.23391e4	942.06964	51.1670

Totals : 2.41154e4 2006.30914

Enantioenriched:

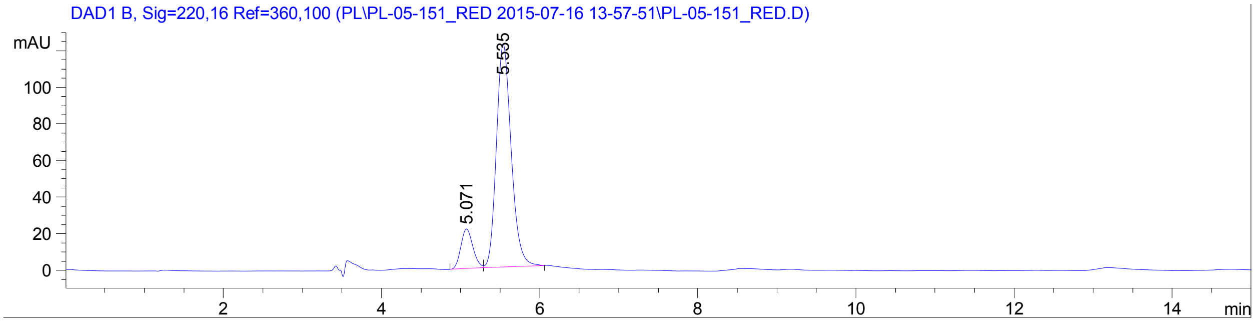

Peak #	RetTime [min]	Type	Width [min]	Area [mAU*s]	Height [mAU]	Area %
1	5.070	BV	0.1556	15.70452	1.48288	12.7406
2	5.534	VB	0.2030	107.55946	8.09679	87.2594
Totals :				123.26398	9.57967	

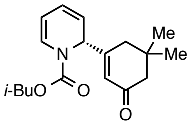

5x

Isobutyl 2-(5,5-dimethyl-3-oxocyclohex-1-en-1-yl)pyridine-1(2H)-carboxylate (5x): General procedure was followed with pyridine (40 mg, 0.50 mmol) with the following modifications: the vinylzinc iodide was used, and was prepared according to literature procedure.[8] No reaction was observed at the typical reaction temperature of –40 °C; instead, the reaction was allowed to warm from –78 °C → rt overnight. **5x** (84 mg, 0.28 mmol 55% yield, 55% ee) was isolated as a clear, yellow oil. (run 1). Run 2 provided 56% yield, 55% ee.

^{1}H NMR (300 MHz, $CDCl_3$): δ 6.87 (dd, *J* = 39.2, 8.5 Hz, 1H), 5.99 (dd, *J* = 9.5, 5.6 Hz, 1H), 5.90 (d, *J* = 8.9 Hz, 1H), 5.62–5.39 (m, 1H), 5.37–5.11 (m, 2H), 4.04–3.90 (m, 2H), 2.27 (s, 2H), 2.22 (s, 2H), 2.10–1.79 (m, 1H), 1.03 (s, 6H), 0.99–0.87 (m, 6H) ppm.

^{13}C NMR (125 MHz, $CDCl_3$): δ 200.50, 160.25, [126.79, 125.77*], [124.66, 123.97], [123.45, 123.03*], [122.60*, 122.32], [118.44*, 118.18], [104.75*, 104.49], 72.89, [58.15, 56.82*], 51.44, [39.69*, 39.11], [34.01*, 33.90], [28.77, 28.49*], [28.17*, 28.06], [28.00, 27.87*], [19.19*, 19.05] ppm. (The two diastereotopic isobutyl Me carbons have the same chemical shift.)

HRMS: (ESI-TOF) calculated for $C_{18}H_{26}NO_3$ ($[M+H]^+$): 304.1907, found: 304.1873.

FTIR (thin film, cm^{-1}): 2959, 1711, 1669, 1279, 1246.

[8] Knochel, P.; Rao, C. J. *Tetrahedron*, **1993**, *49*, 29–48.

Optical rotation: $[\alpha]_D^{26}$ +88.0 (*c* 0.59, $CHCl_3$).

HPLC: Chiralcel OD-H, 5% IPA in hexanes, 30 min run, 1 mL/min.

Racemic Standard:

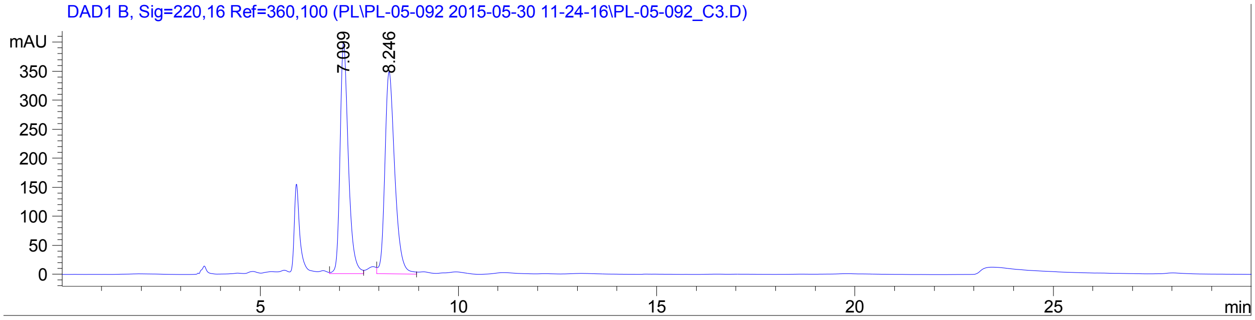

Peak #	RetTime [min]	Type	Width [min]	Area [mAU*s]	Height [mAU]	Area %
1	7.099	VV	0.2195	5795.53125	398.97430	48.8004
2	8.246	VB	0.2633	6080.45801	348.89514	51.1996
Totals :				1.18760e4	747.86945	

Enantioenriched:

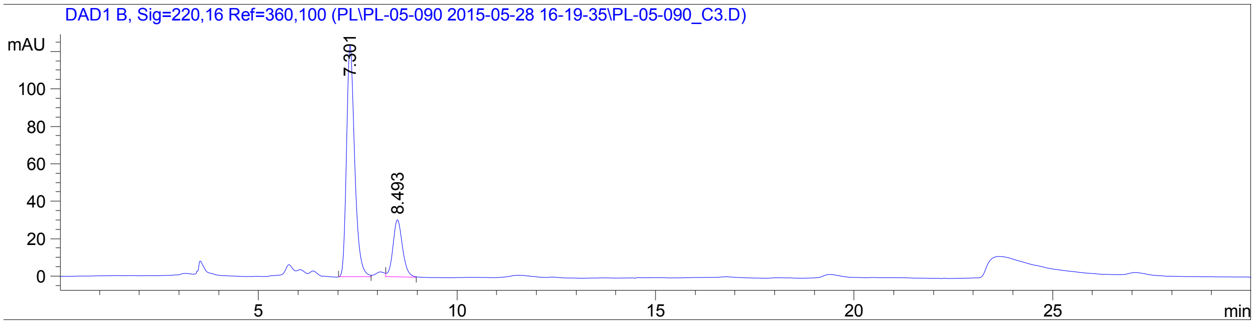

Peak #	RetTime [min]	Type	Width [min]	Area [mAU*s]	Height [mAU]	Area %
1	7.301	BB	0.2168	1765.17200	123.48444	77.4268
2	8.493	VB	0.2562	514.62146	30.60568	22.5732
Totals :				2279.79346	154.09013	

IV. Derivatization Procedures/Characterization of Compounds 7–15

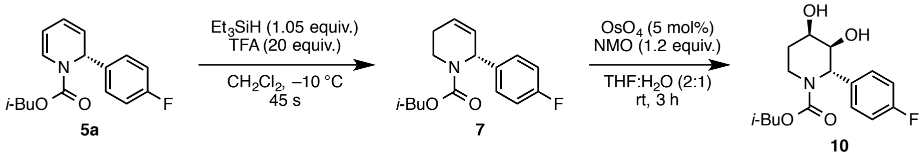

(*R*)-Isobutyl 6-(4-fluorophenyl)-3,6-dihydropyridine-1(2H)-carboxylate (7)[9]: In a threaded 16 mm x 100 mm glass vial equipped with a stir bar, **5a** (103 mg, 0.374 mmol, 1.0 equiv.) was dissolved in CH_2Cl_2 (2 mL). A solution of triethylsilane (63 µL, 0.39 mmol, 1.1 equiv.) in CH_2Cl_2 (1 mL) was added. The solution was cooled to –10 °C in a Cryocool, and trifluoroacetic acid (0.57 mL, 7.5 mmol, 20 equiv.) was added in one portion. The reaction was allowed to stir at –10 °C for 45 s, then the reaction was neutralized with sat. aq. $NaHCO_3$. The reaction mixture was extracted with CH_2Cl_2 (3 x 10 mL), and the combined organic fractions were dried over $MgSO_4$ and concentrated *in vacuo*. The crude mixture was purified by automated column chromatography (1→8% EtOAc in hexanes) to give **7** as a clear, colorless oil (62 mg, 0.22 mmol, 60% yield, 88% ee).

^{1}H NMR (300 MHz, $CDCl_3$): δ 7.36 (br. s, 2H), 7.01 (t, *J* = 8.7 Hz, 2H), 6.13–5.98 (m, 1H), 5.89–5.79 (m, 1), 5.71–5.45 (m, 1H), 4.31–4.01 (m, 1H), 4.00–3.79 (m, 2H), 2.91 (ddd, *J* = 13.3, 12.0, 4.0 Hz, 1H), 2.46–2.21 (m, 1H), 2.16–1.97 (m, 1H), 1.93 (sept., *J* = 6.7 Hz, 1H), 0.92 (d, *J* = 6.7 Hz, 6H) ppm.

^{13}C NMR (125 MHz, $CDCl_3$): δ 162.28 (d, *J* = 246.2 Hz), 155.62, 136.82, 129.56 (d, *J* = 71.8 Hz), 127.05, 126.79, 115.28 (d, *J* = 21.3 Hz), 71.79, 54.23, 36.97, 28.17, 25.06, 19.30 ppm. (The two diastereotopic Me carbons have the same chemical shift.)

^{19}F NMR (282 MHz, $CDCl_3$): δ –115.23 ppm.

HRMS: (ESI-TOF) calculated for $C_{16}H_{21}FNO_2$ ($[M+H]^+$): 278.1551, found: 278.1525.

FTIR (thin film, cm^{-1}): 2962, 1695, 1508, 1425, 1223, 1109.

Optical rotation: $[\alpha]_D^{26}$ –1.9 (*c* 1.0, $CHCl_3$).

HPLC: Chiralcel OJ-H, 5% IPA in hexanes, 15 min run, 1 mL/min.

[9] Mancheño, O. G.; Asmus, S.; Zurro, M.; Fischer, T. *Angew. Chem. Int. Ed.* **2015**, *54*, 8823–8827.

Racemic Standard:

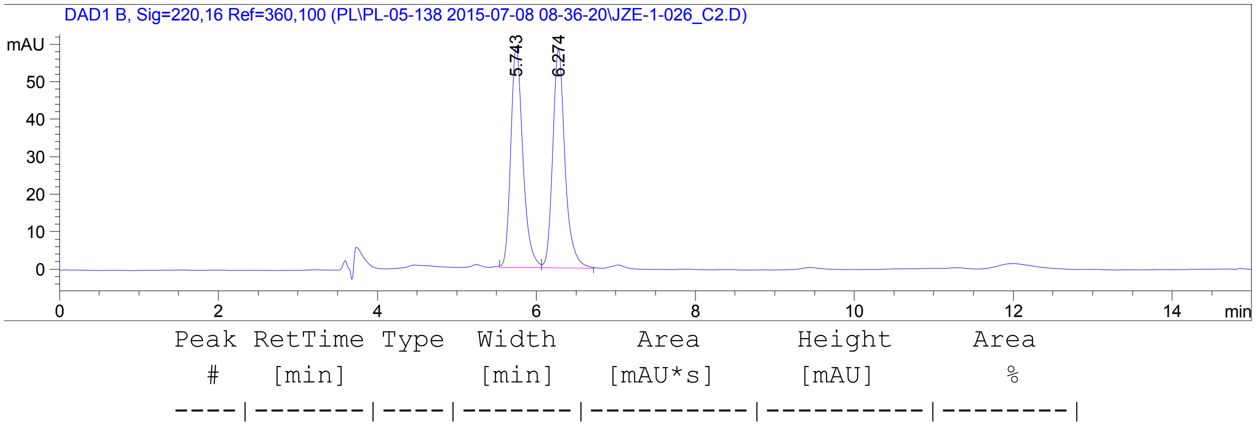

Peak #	RetTime [min]	Type	Width [min]	Area [mAU*s]	Height [mAU]	Area %
1	5.743	BV	0.1612	625.90216	59.27720	49.7585
2	6.274	VB	0.1640	631.97870	58.51051	50.2415

Totals : 1257.88086 117.78771

Enantioenriched:

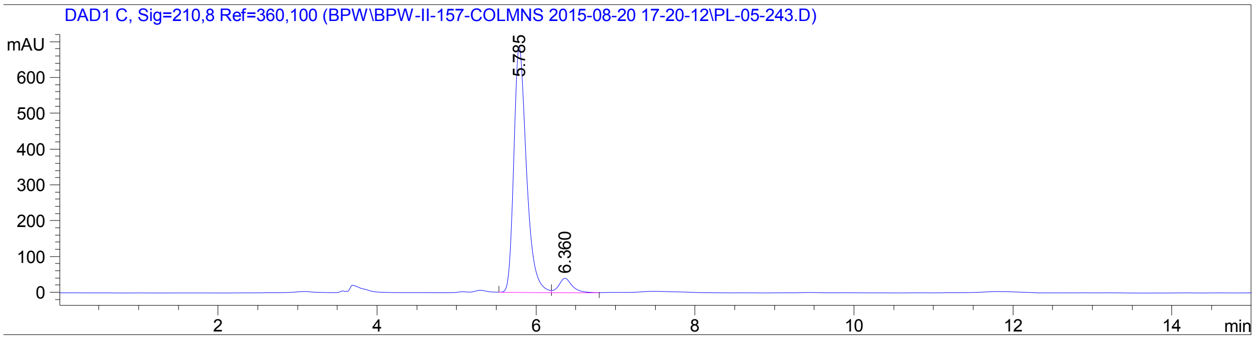

Peak #	RetTime [min]	Type	Width [min]	Area [mAU*s]	Height [mAU]	Area %
1	5.785	VV	0.1672	7608.74902	686.81000	94.0579
2	6.360	VB	0.1762	480.68155	40.57185	5.9421

Totals : 8089.43057 727.38185

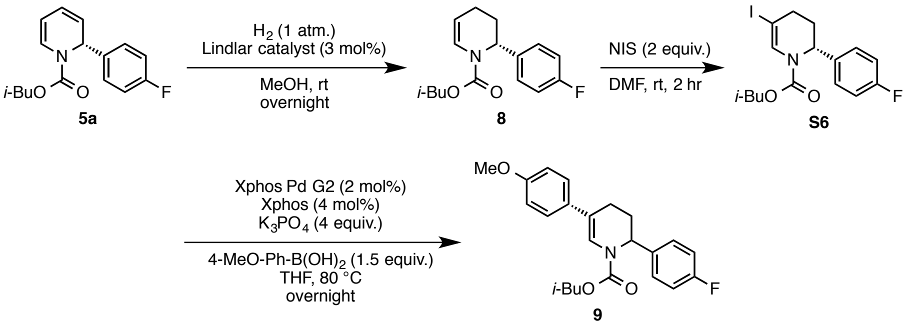

(*R*)-Isobutyl 2-(4-fluorophenyl)-3,4-dihydropyridine-1(2H)-carboxylate (8)[10]: Compound **5a** (104 mg, 0.376 mmol, 1.0 equiv.) was dissolved in MeOH (2 mL) in a 2-dram vial charged with a stir bar. Lindlar catalyst (24 mg, 0.011 mmol, 3.0 mol%) was added, and the vial was capped with a septum cap. The vial was evacuated and refilled with a balloon of H_2. The reaction was allowed to stir at rt under 1 atm H_2 overnight. The reaction mixture was filtered through Celite with Et_2O and concentrated *in vacuo* to give **8** (96 mg, 0.35 mmol, 92% yield, 90% ee) as a clear, colorless oil, which was used without further purification.

^{1}H NMR (300 MHz, $CDCl_3$): δ 7.21–6.92 (m, 5H), 5.53–5.17 (m, 1H), 5.11–4.84 (m, 1H) 4.11–3.72 (m, 2H), 2.19–1.66 (m, 5H), 1.12–0.58 (m, 6H) ppm.

^{13}C NMR (125 MHz, $CDCl_3$): δ 161.87 (d, *J* = 244.2 Hz), 149.82, 136.98, 128.84 (d, *J* = 8.4 Hz), [127.75, 127.06*], [125.33*, 124.82], [115.80 (d, *J* = 21.5 Hz), 115.32* (d, *J* = 21.4 Hz)], [106.35, 106.17], [72.31, 72.03*], [54.41*, 53.72], [27.87*, 27.56], [19.29*, 19.23], [18.85, 18.83*], [17.30*, 16.99] ppm.

^{19}F NMR (282 MHz, $CDCl_3$): δ [–116.36*, –116.51] ppm.

HRMS: (ESI-TOF) calculated for $C_{16}H_{21}FNO_2$ ($[M+H]^+$): 278.1551, found: 278.1538.

FTIR (thin film, cm^{-1}): 2961, 2875, 1701, 1512, 1407, 1229.

Optical rotation: $[\alpha]_D^{26}$ +48.5 (*c* 1.1, $CHCl_3$).

HPLC: Chiralcel OJ-H, 5% IPA in hexanes, 15 min run, 1 mL/min.

[10] Trost, B. M.; Biannic, B. *Org. Lett.* **2015**, *17*, 1433–1436.

Racemic Standard:

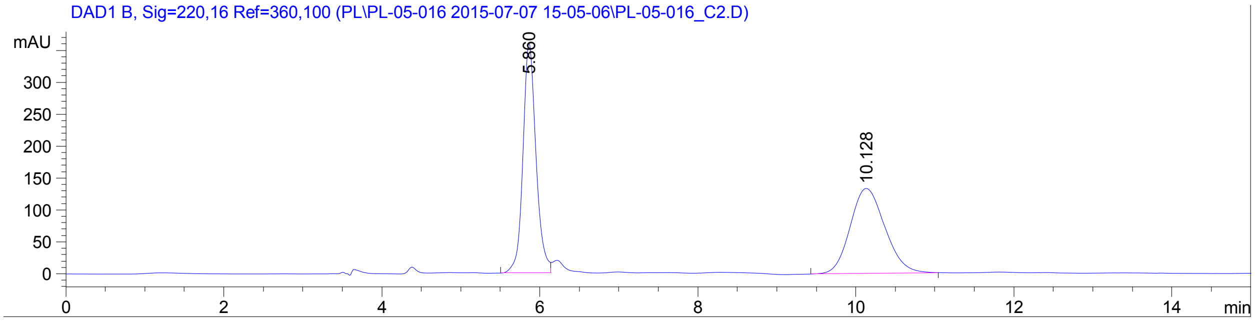

Peak #	RetTime [min]	Type	Width [min]	Area [mAU*s]	Height [mAU]	Area %
1	5.860	BV	0.1740	4137.99707	360.33514	50.3411
2	10.128	BB	0.4713	4081.91797	133.30258	49.6589

Totals : 8219.91504 493.63773

Enantioenriched:

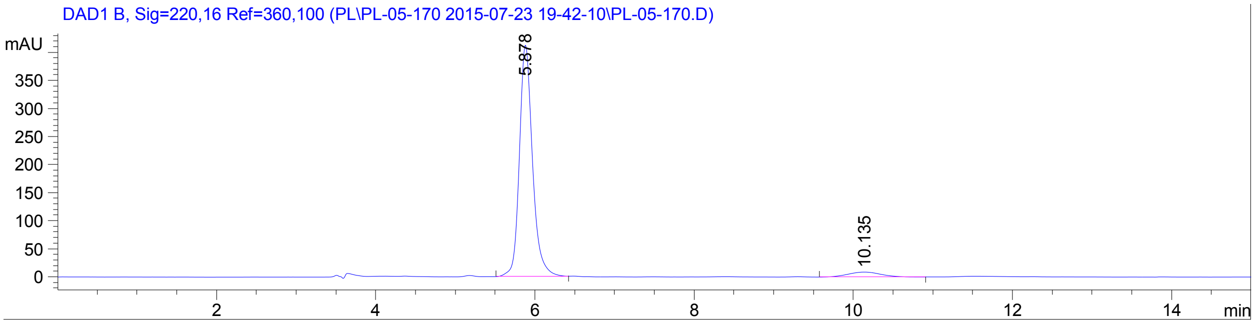

Peak #	RetTime [min]	Type	Width [min]	Area [mAU*s]	Height [mAU]	Area %
1	5.878	BB	0.1755	4719.14893	412.39029	94.9467
2	10.135	BB	0.4447	251.16324	8.75399	5.0533

Totals : 4970.31216 421.14428

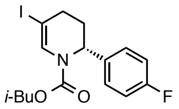

S6

(*R*)-Isobutyl 2-(4-fluorophenyl)-5-iodo-3,4-dihydropyridine-1(2H)-carboxylate (S6): In a 25-mL round-bottomed flask equipped with a stir bar, **8** (110 mg, 0.396 mmol, 1.00 equiv.) was dissolved in DMF (2 mL). *N*-Iodosuccinimide (recrystalized from 1:1 Et_2O:dioxanes, 178 mg, 0.793 mmol, 2.00 equiv.) was added, and the reaction was allowed to stir at rt for 2 h. The reaction was quenched with 10% aq.

$Na_2S_2O_3$ (2 mL) and extracted with Et_2O (3 x 5 mL). The combined organic fractions were washed with brine (1 x 10 mL), dried over $MgSO_4$, and concentrated *in vacuo*. The crude product was purified by automated column chromatography (1→10% EtOAc in hexanes) to give **S6** (101 mg, 0.249 mmol, 63% yield, 90% ee) as a clear, colorless oil.

^{1}H NMR (300 MHz, $CDCl_3$): δ 7.70–7.40 (m, 1H), 7.18–7.07 (m, 2H), 7.01 (t, *J* = 8.7 Hz, 2H), 5.40 (d, *J* = 28.0 Hz, 1H), 3.97–3.63 (m, 2H), 2.45–2.07 (m, 3H), 2.08–1.89 (m, 2H), 1.08–0.59 (m, 6H) ppm.

^{19}F NMR (282 MHz, $CDCl_3$): δ [–115.58, –115.73*] ppm.

HPLC: Chiralcel OJ-H, 5% IPA in hexanes, 15 min run, 1 mL/min.

Racemic Standard:

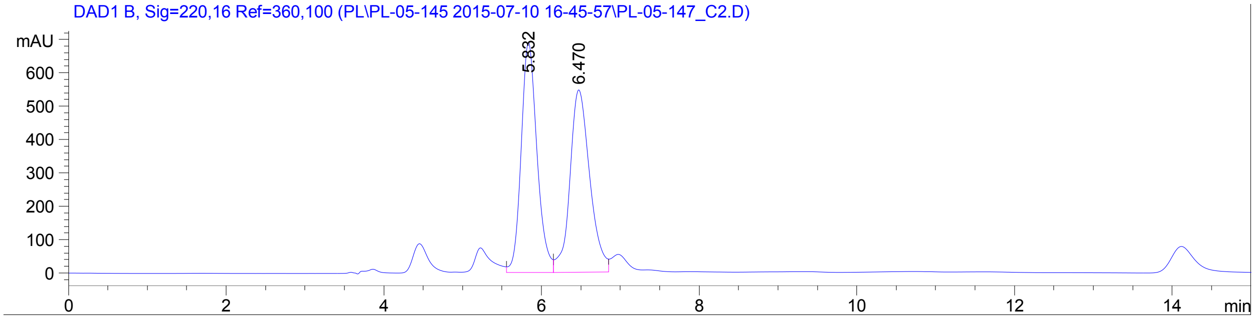

Peak #	RetTime [min]	Type	Width [min]	Area [mAU*s]	Height [mAU]	Area %
1	5.832	VV	0.2118	9693.67676	690.53967	50.2199
2	6.470	VV	0.2689	9608.78027	547.20123	49.7801
Totals :				1.93025e4	1237.74091	

Enantioenriched:

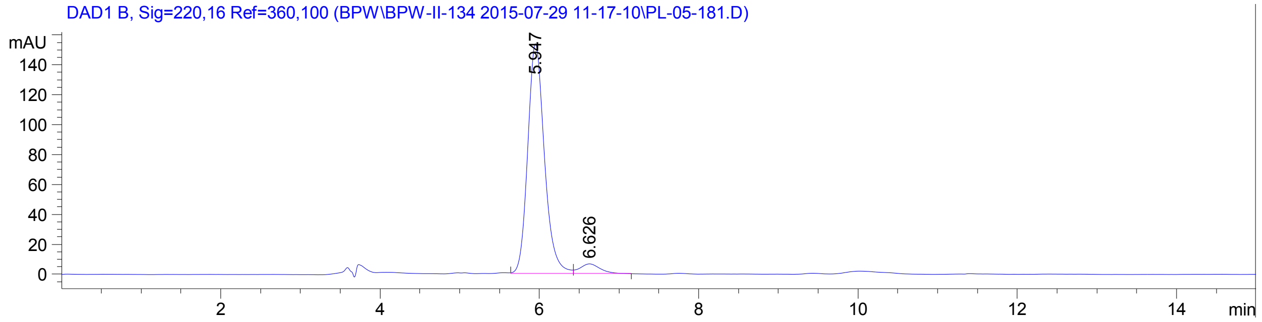

Peak #	RetTime [min]	Type	Width [min]	Area [mAU*s]	Height [mAU]	Area %
1	5.947	BB	0.2243	2245.85547	153.88699	94.8279
2	6.626	BB	0.2674	122.49378	6.69539	5.1721

Totals : 2368.34925 160.58238

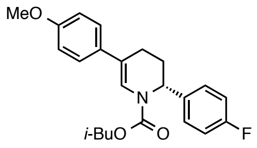

9

(*R*)-Isobutyl 2-(4-fluorophenyl)-5-(4-methoxyphenyl)-3,4-dihydropyridine-1(2H)-carboxylate (9): To a threaded 16 mm x 100 mm glass vial equiped with a stir bar were added sequentially Xphos Pd G2 (1.8 mg, 2.3 µmol, 2.0 mol%), Xphos (2.2 mg, 4.5 µmol, 4.0 mol%), K_3PO_4 (48 mg, 0.23 mmol, 2.0 equiv.), and (4-methoxyphenyl)boronic acid (26 mg, 0.17 mmol, 1.5 equiv.). The vial was evacuated and refilled with N_2 (x3), and **S6** (45.7 mg, 0.113 mmol, 1.0 equiv.) was added as a solution in dry THF (1 mL). The reaction was allowed to stir at 80 °C overnight, then quenched with H_2O (10 mL). The resulting mixture was extracted with Et_2O (3 x 10 mL), and the combined organic fractions were dried over $MgSO_4$ and concentrated *in vacuo*. Automated column chromatography (1→15% EtOAc in hexanes) provided **9** (26 mg, 0.068 mmol, 60% yield, 90% ee) as a clear, colorless oil.

^{1}H NMR (300 MHz, $CDCl_3$): δ 7.52 (d, *J* = 51.3 Hz, 1H), 7.33 (d, *J* = 8.4 Hz, 2H), 7.20–7.10 (m, 2H), 6.97 (t, *J* = 8.7 Hz, 2H), 6.87 (d, *J* = 8.6 Hz, 2H), 5.40 (d, *J* = 33.3 Hz, 1H), 4.06–3.85 (m, 2H), 3.81 (s, 3H), 2.33 (d, *J* = 14.9 Hz, 1H), 2.24–2.04 (m, 3H), 1.96–1.69 (m, 1H), 1.06–0.62 (m, 6H) ppm.

^{13}C NMR (125 MHz, $CDCl_3$): δ 161.93 (d, *J* = 245.0 Hz), 158.37, [137.94*, 137.34], [133.59, 132.61*], [128.23, 126.79*], [127.12*, 127.06], 125.50, [121.32*, 120.83], [115.50, 115.33*], [114.08, 114.03*], [72.49, 72.24*], [71.88*, 71.79], 55.49, [54.01*, 53.39], [28.18*, 28.10], [27.96*, 27.75], [20.11, 19.62*], [19.29*, 19.23], [18.92, 18.84] ppm.

^{19}F NMR (282 MHz, $CDCl_3$): δ [–116.11*, –116.26] ppm.

HRMS: (ESI-TOF) calculated for $C_{23}H_{27}FNO_3$ ($[M+H]^+$): 384.1969, found: 384.1941.

FTIR (thin film, cm^{-1}): 2959, 1702, 1506, 1403, 1322, 1249.

Optical rotation: $[\alpha]_D^{26}$ +97.0 (*c* 0.88, $CHCl_3$).

HPLC: Chiralcel OD-H, 5% IPA in hexanes, 30 min run, 1 mL/min.

Racemic Standard:

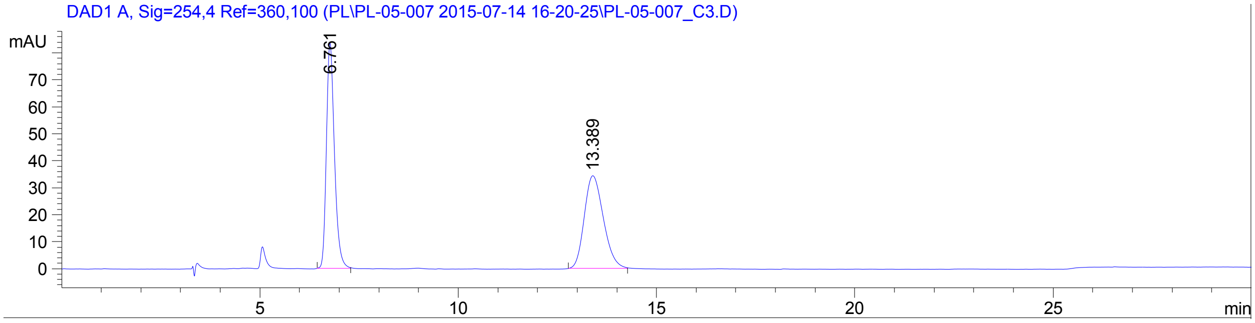

Peak #	RetTime [min]	Type	Width [min]	Area [mAU*s]	Height [mAU]	Area %
1	6.761	BB	0.2149	1183.37732	83.71516	50.6544
2	13.389	BB	0.5185	1152.80237	34.43817	49.3456
Totals :				2336.17969	118.15333	

Enantioenriched:

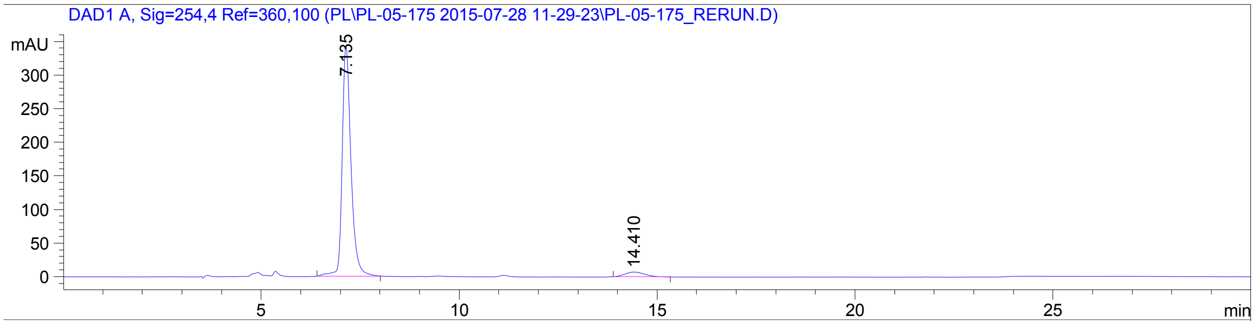

Peak #	RetTime [min]	Type	Width [min]	Area [mAU*s]	Height [mAU]	Area %
1	7.135	BB	0.2332	5326.82422	343.01379	95.2312
2	14.410	BB	0.5419	266.74881	7.41152	4.7688
Totals :				5593.57303	350.42531	

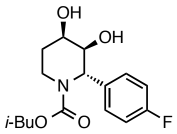

10

(2*S*,3*S*,4*R*)-Isobutyl 2-(4-fluorophenyl)-3,4-dihydroxypiperidine-1-carboxylate (10): In a 2-dram vial equipped with a stir bar, **7** (30 mg, 0.11 mmol, 1.0 equiv.) was dissolved in THF:H_2O (2:1, 0.3 mL). *N*-Methylmorpholine *N*-oxide (50 wt% in water, 30 µL, 0.13 mmol, 1.2 equiv.) was added, followed by OsO_4 (4 wt% in water, 34 µL, 5.4 µmol, 0.050 equiv.). The reaction was allowed to stir at rt for 3 h, then 2 mL water was added. The reaction mixture was extracted with Et_2O (6 x 2 mL), and the combined organic fractions were dried over $MgSO_4$ and concentrated *in vacuo* to provide **10** (24 mg, 0.076 mmol, 70% yield, >20:1 dr) as a clear, amber oil.

^{1}H NMR (300 MHz, $CDCl_3$): δ 7.20 (dd, *J* = 8.0, 5.3 Hz, 2H), 7.04 (t, *J* = 8.6 Hz, 2H), 5.61 (s, 1H), 4.49 (s, 1H), 4.22 (d, *J* = 13.4 Hz, 1H), 3.93 (dd, *J* = 6.6, 2.1 Hz, 2H), 3.80–3.56 (m, 1H), 2.87 (td, *J* = 13.6, 3.0 Hz, 1H), 2.50–2.31 (m, 1H), 2.07–1.81 (m, 3H), 1.82–1.56 (m, 1H), 0.90 (d, *J* = 6.7 Hz, 6H) ppm.

^{13}C NMR (125 MHz, $CDCl_3$): δ 162.00 (d, *J* = 246.6 Hz), 157.35, 132.49 (d, *J* = 3.2 Hz), 127.95 (d, *J* = 8.0 Hz), 115.96 (d, *J* = 21.4 Hz), 72.26, 70.55, 66.83, 59.43, 39.08, 28.13, 19.19, 6.74, 5.93 ppm.

^{19}F NMR (282 MHz, $CDCl_3$): δ –115.21 ppm.

HRMS: (ESI-TOF) calculated for $C_{16}H_{23}FNO_4$ ($[M+H]^+$): 312.1606, found: 312.1598.

FTIR (thin film, cm^{-1}): 3409, 2961, 1673, 1510, 1430, 1327.

Optical rotation: $[\alpha]_D^{26}$ –6.8 (*c* 1.1, $CHCl_3$).

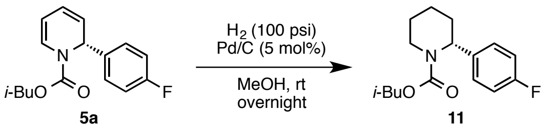

5a **11**

(*R*)-Isobutyl 2-(4-fluorophenyl)piperidine-1-carboxylate (11): In a 2-dram vial equipped with a stir bar, **5a** (99 mg, 0.36 mmol, 1.0 equiv.) was dissolved in MeOH (2 mL). Palladium on carbon (19 mg of 10 wt% catalyst, 0.018 mmol, 5.0 mol%) was added and the vial was capped with a septum cap pierced with an 18 G needle. The vial was transferred to a Parr reactor, which was filled with H_2 (100 psi). The reaction was left to stir at rt overnight. The crude reaction mixture was filtered through Celite with Et_2O, and the solvent was removed *in vacuo* to provide **11** (93 mg, 0.33 mmol, 92% yield, 89% ee) as a clear, colorless oil, which was used without further purification.

^{1}H NMR (300 MHz, $CDCl_3$): δ 7.19 (dd, *J* = 8.0, 5.4 Hz, 2H), 7.02 (t, *J* = 8.7 Hz, 2H), 5.44 (d, *J* = 4.5 Hz, 1H), 4.09 (d, *J* = 12.6 Hz, 1H), 3.92 (dd, *J* = 6.6, 4.1 Hz, 2H), 2.78 (ddd, *J* = 13.5, 11.5, 3.9 Hz, 1H), 2.27 (d, *J* = 14.4 Hz, 1H), 2.01–1.78 (m, 2H), 1.73–1.57 (m, 2H), 1.53–1.37 (m, 2H), 0.90 (d, *J* = 6.7 Hz, 6H) ppm.

^{13}C NMR (125 MHz, $CDCl_3$): δ 161.67 (d, *J* = 244.8 Hz), 156.50, 135.78 (d, *J* = 3.1 Hz), 128.26 (d, *J* = 7.9 Hz), 115.50 (d, *J* = 21.2 Hz), 71.79, 52.99, 40.34, 28.24, 28.18, 25.54, 19.39, 19.24, 19.23 ppm.

^{19}F NMR (282 MHz, $CDCl_3$): δ –116.93 ppm.

HRMS: (ESI-TOF) calculated for $C_{16}H_{23}FNO_2$ ($[M+H]^+$): 280.1707, found: 280.1686.

FTIR (thin film, cm^{-1}): 2944, 2871, 1693, 1509, 1423, 1258, 1158.

Optical rotation: $[\alpha]_D^{26}$ +80.2 (*c* 1.0, $CHCl_3$).

HPLC: Chiralcel OJ-H, 5% IPA in hexanes, 15 min run, 1 mL/min.

Racemic Standard:

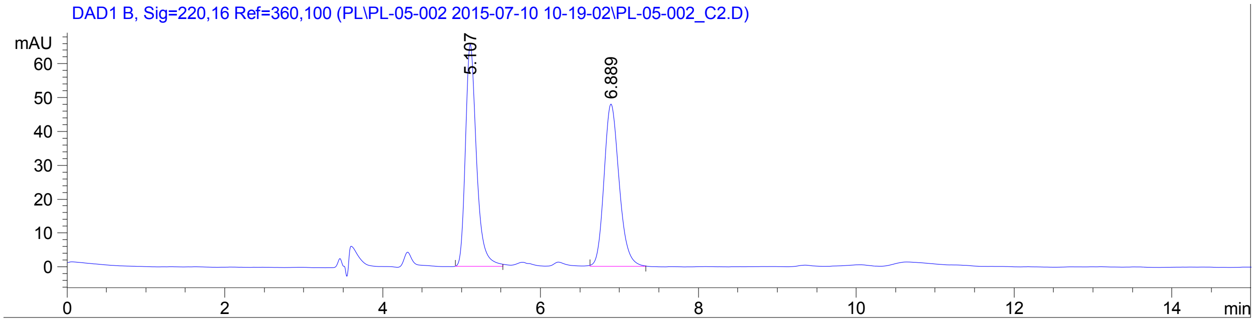

Peak #	RetTime [min]	Type	Width [min]	Area [mAU*s]	Height [mAU]	Area %
1	5.107	BB	0.1477	640.28522	65.67620	49.9236
2	6.889	BB	0.2039	642.24493	48.05778	50.0764

Totals : 1282.53015 113.73398

Enantioenriched:

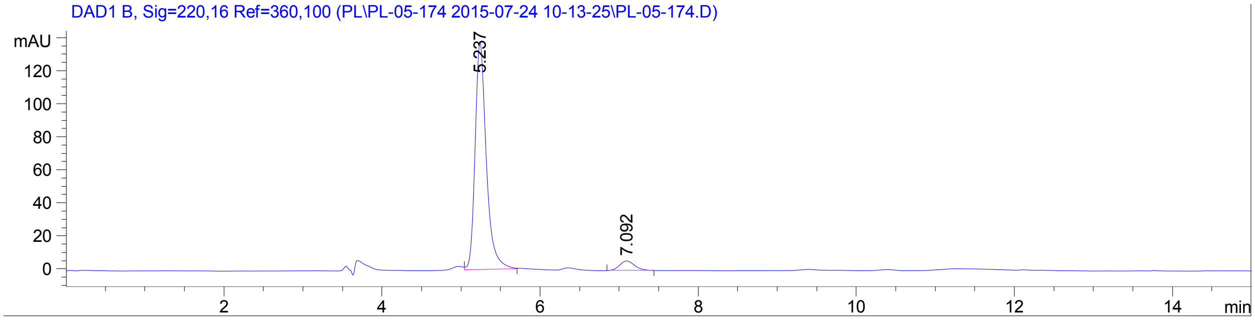

Peak #	RetTime [min]	Type	Width [min]	Area [mAU*s]	Height [mAU]	Area %
1	5.237	VB	0.1491	1356.38782	137.49344	94.4637
2	7.092	BB	0.2086	79.49426	5.84854	5.5363
Totals :				1435.88207	143.34198	

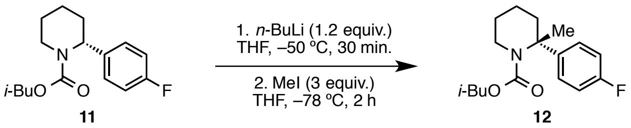

(*R*)-Isobutyl 2-(4-fluorophenyl)-2-methylpiperidine-1-carboxylate (12)[11]: In a threaded 16 mm x 100 mm glass vial under N_2 and equipped with a stir bar, **11** (164 mg, 0.587 mmol, 1.0 equiv.) was dissolved in dry THF (4 mL). The vial was cooled to –78 °C and *n*-BuLi (titrated as 2.4 M in hexanes, 0.29 mL, 0.70 mmol, 1.2 equiv.) was added. The reaction was moved to a Cryocool at –50 °C and allowed to stir at this temperature for 30 min. At this point, the reaction was again cooled to –78 °C and MeI (0.11 mL, 1.8 mmol, 3.0 equiv.) was added. The reaction was allowed to stir 2 h at –78 °C, then quenched with glacial acetic acid (0.5 mL). The crude reaction mixture was filtered through Celite and concentrated *in vacuo*, then purified by automated column chromatography (1→15% EtOAc in hexanes) to give **12** (141 mg, 0.481 mmol, 82% yield, 88% ee) as a clear, colorless oil.

^{1}H NMR (300 MHz, $CDCl_3$): δ 7.23 (d, *J* = 5.3 Hz, 2H), 6.97 (t, *J* = 8.8 Hz, 2H), 3.73–3.59 (m, 4H), 1.91–1.79 (m, 2H), 1.76 (s, 3H), 1.76–1.66 (m, 2H), 1.65–1.56 (m, 3H), 0.71 (d, *J* = 6.1 Hz, 6H) ppm.

^{13}C NMR (125 MHz, $CDCl_3$): δ 161.17 (d, *J* = 243.7 Hz), 157.03, 144.85 (d, *J* = 2.5 Hz), 126.18 (d, *J* = 7.8 Hz), 114.92 (d, *J* = 21.1 Hz), 71.57, 41.81, 41.03, 27.88, 25.13, 23.49, 23.28, 19.19, 19.14, 18.14 ppm.

^{19}F NMR (470 MHz, $CDCl_3$): δ –118.21 (tt, *J* = 8.5, 4.9 Hz) ppm.

[11] Sheikh, N. S.; Leonori, D.; Barker, G.; Firth, J. D.; Campos, K. R.; Meijer, A. J. H. M.; O'Brien, P.; Coldham, I. *J. Am. Chem. Soc.* **2012**, *134*, 5300–5308.

HRMS: (ESI-TOF) calculated for $C_{17}H_{25}FNO_2$ ($[M+H]^+$): 294.1864, found: 294.1846.

FTIR (thin film, cm^{-1}): 2947, 2872, 1687, 1509, 1263, 1226, 1070.

Optical rotation: $[\alpha]_D^{26}$ +11.8 (*c* 1.0, $CHCl_3$).

HPLC: Chiralcel OD-H, 2.5% IPA in hexanes, 30 min run, 1 mL/min.

Racemic Standard:

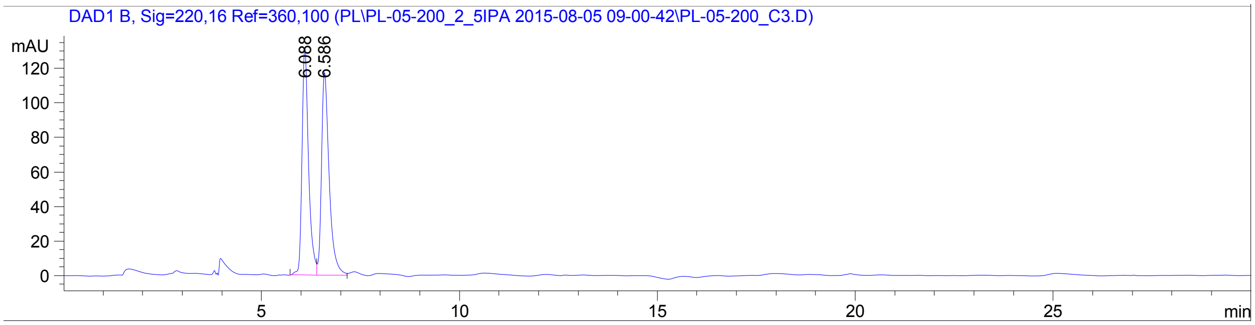

Peak #	RetTime [min]	Type	Width [min]	Area [mAU*s]	Height [mAU]	Area %
1	6.088	BV	0.1724	1521.17444	132.00325	48.9333
2	6.586	VB	0.2018	1587.49255	117.40365	51.0667
Totals :				3108.66699	249.40690	

Enantioenriched:

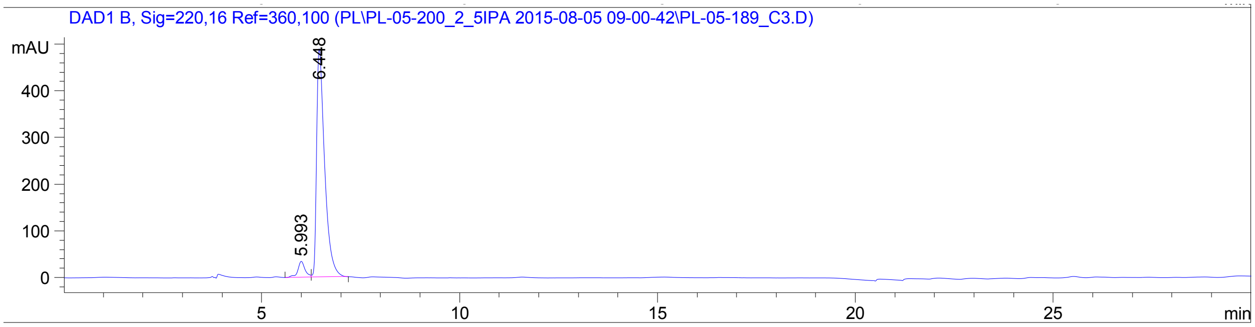

Peak #	RetTime [min]	Type	Width [min]	Area [mAU*s]	Height [mAU]	Area %
1	5.993	VV	0.1887	443.39261	34.32303	6.0467
2	6.448	VB	0.2103	6889.36572	489.27774	93.9533
Totals :				7332.75833	523.60077	

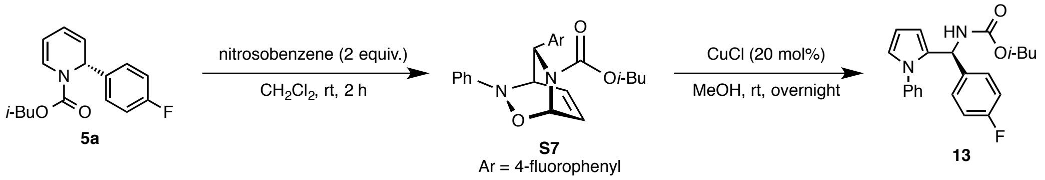

Isobutyl 6-(4-fluorophenyl)-2-phenyl-3-oxa-2,5-diazabicyclo[2.2.2]oct-7-ene-5-carboxylate (S7)[12]: In a 25-mL round-bottomed flask equipped with a stir bar, **5a** (107 mg, 0.387 mmol, 1.0 equiv.) was dissolved in CH_2Cl_2 (1 mL). Nitrosobenzene (83 mg, 0.77 mmol, 2.0 equiv.) was added, and the reaction was allowed to stir at rt for 2 h. The reaction mixture was concentrated *in vacuo*, then purified by automated column chromatography (1→10% EtOAc in hexanes) to give **S7** (129 mg, 0.337 mmol, 87% yield) as a tan solid.

^{1}H NMR (300 MHz, $CDCl_3$): δ 7.27–6.94 (m, 9H), 6.82 (ddd, *J* = 7.7, 5.7, 1.9 Hz, 1H), 6.53–6.42 (m, 1H), 5.94–5.80 (m, 1H), 5.35–5.15 (m, 1H), 4.65–4.48 (m, 1H), 3.95–3.79 (m, 1H), 3.79–3.68 (m, 1H), 1.74–1.59 (m, 1H), 1.07–0.77 (m, 2H), 0.60 (d, *J* = 6.0 Hz, 4H) ppm.

^{19}F NMR (282 MHz, $CDCl_3$): δ –114.91 ppm.

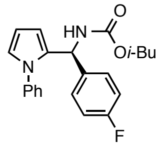

13

(*S*)-Isobutyl ((4-fluorophenyl)(1-phenyl-1H-pyrrol-2-yl)methyl)carbamate (13): In a 2-dram vial equipped with a stir bar, **S7** (128 mg, 0.335 mmol, 1.00 equiv.) was dissolved in MeOH (4 mL). CuCl (6.6 mg, 0.067 mmol, 0.20 equiv.) was added, and the reaction was allowed to stir at rt overnight. The reaction was quenched with water (10 mL) and extracted with CH_2Cl_2 (3 x 10 mL). The combined organic fractions were dried with $MgSO_4$, concentrated *in vacuo*, and purified by automated column chromatography to give **13** (26 mg, 0.070 mmol, 21% yield, 89% ee) as a clear, colorless oil.

^{1}H NMR (300 MHz, $CDCl_3$): δ 7.43 (dd, *J* = 8.4, 5.5 Hz, 2H), 7.16 (t, *J* = 7.9 Hz, 2H), 6.99 (t, *J* = 8.7 Hz, 2H), 6.73 (t, *J* = 7.3 Hz, 1H), 6.55 (d, *J* = 7.8 Hz, 2H), 6.24 (dd, *J* = 10.1, 5.9 Hz, 1H), 6.10 (dd, *J* = 10.4, 3.7 Hz, 1H), 5.76–5.61 (m, 2H), 4.42–4.24 (m, 1H), 3.87 (s, 1H), 3.73–3.59 (m, 1H), 1.93–1.62 (m, 1H), 1.05–0.51 (m, 6H) ppm.

^{13}C NMR (125 MHz, $CDCl_3$): δ 162.07 (d, *J* = 246.0 Hz), 145.90, 129.86 (d, *J* = 6.8 Hz), [129.75, 129.66*], [129.25, 128.91], [127.81*, 127.75], [126.82, 126.53*], [118.75, 118.50*], [115.36 (d, *J* = 20.9 Hz), 115.10* (d, *J* = 21.2 Hz)], [114.15, 113.85*], 113.54, 78.84, [72.54, 72.45*], 66.01, [55.90, 54.40], 49.28, [29.85*, 27.95], [18.93*, 18.72] ppm.

^{19}F NMR (282 MHz, $CDCl_3$): δ –115.16 ppm.

[12] Berti, F.; Di Bussolo, V.; Pineschi, M. *J. Org. Chem.* **2013**, *78*, 7324–7329.

HRMS: (ESI-TOF) calculated for $C_{22}H_{24}FN_2O_2$ ($[M+H]^+$): 367.1816, found: 367.1802.

FTIR (thin film, cm^{-1}): 2963, 1705, 1602, 1509, 1228.

Optical rotation: $[\alpha]_D^{26}$ –84.0 (*c* 0.57, $CHCl_3$).

HPLC: Chiralpak AD-H, 5% IPA in hexanes, 30 min run, 1 mL/min.

Racemic Standard:

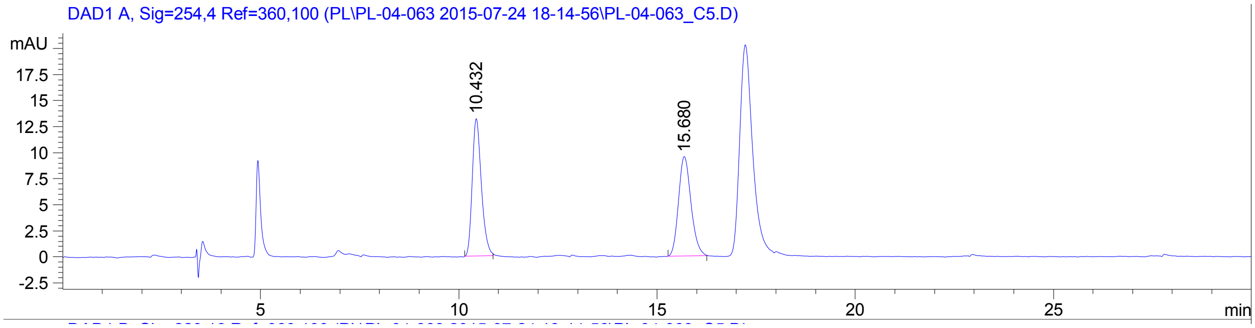

Peak #	RetTime [min]	Type	Width [min]	Area [mAU*s]	Height [mAU]	Area %
1	10.432	BB	0.2453	147.34619	9.17585	50.3352
2	15.680	BB	0.3343	145.38367	6.61667	49.6648
Totals :				292.72986	15.79253	

Enantioenriched:

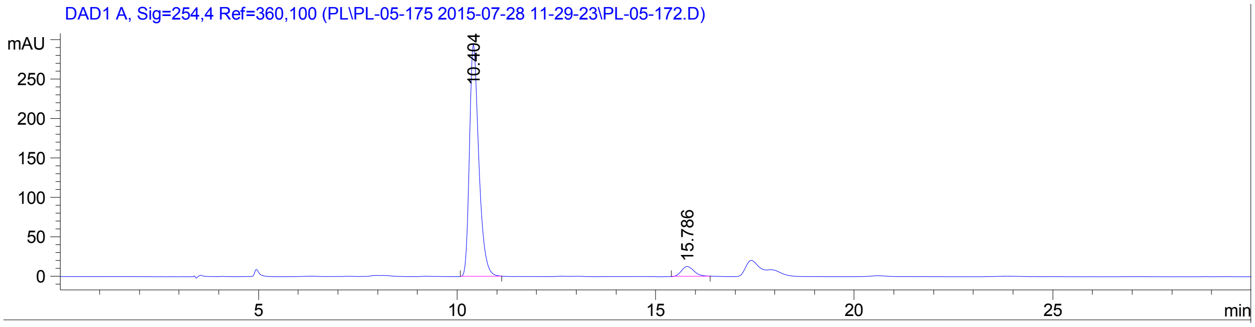

Peak #	RetTime [min]	Type	Width [min]	Area [mAU*s]	Height [mAU]	Area %
1	10.404	BB	0.2467	4805.47510	293.92603	94.3767
2	15.786	BB	0.3223	286.33002	12.73737	5.6233
Totals :				5091.80511	306.66339	

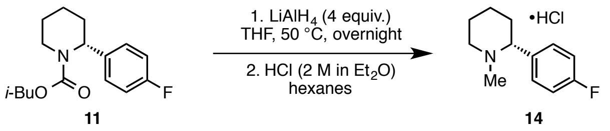

(*R*)-2-(4-Fluorophenyl)-1-methylpiperidine hydrochloride salt (14): In a 25-mL round-bottomed flask equipped with a stir bar, **11** (198 mg, 0.709 mmol, 1.0 equiv.) was dissolved in THF (6 mL). $LiAlH_4$ (110 mg, 2.90 mmol, 4.0 equiv) was added, and the reaction was allowed to stir at 50 °C overnight. The reaction was cooled to 0 °C and H_2O (0.10 mL), 10% aq. NaOH (0.10 mL), and H_2O (0.30 mL) were added sequentially. The crude reaction mixture was filtered through Celite, then filtered through $MgSO_4$ and concentrated *in vacuo*. The concentrated material was dissolved in hexanes and HCl (2 M in Et_2O, 1 mL) was added to precipitate the HCl salt. The precipitate was washed several times with hexanes to provide **14** (128 mg, 0.557 mmol, 79% yield) as a white solid.

In order to determine the ee, the free base was obtained by treatment of **14** with 10% aq. NaOH, followed by extraction with Et_2O, drying with $MgSO_4$, and concentration *in vacuo*. The ee of the free base was determined by gas chromatography (89% ee).

^{1}H NMR (500 MHz, $CDCl_3$): δ 12.49 (br. s, 1H), 7.75 (s, 2H), 7.14 (br. s, 2H), 3.67 (d, *J* = 25.0 Hz, 2H), 2.76 (d, *J* = 84.3 Hz, 2H), 2.49 (s, 3H), 2.14–1.85 (m, 3H), 1.62 (br. s, 2H) ppm.

^{13}C NMR (125 MHz, $CDCl_3$): δ 163.43 (d, *J* = 249.8 Hz), 131.33 (d, *J* = 3.2 Hz), 130.57 (d, *J* = 7.6 Hz), 116.88, 71.71, 58.23, 42.64, 32.69, 23.66, 23.23 ppm.

^{19}F NMR (282 MHz, $CDCl_3$): δ –110.91 ppm.

HRMS: (ESI-TOF) calculated for $C_{12}H_{17}FN$ ($[M]^+$): 194.1340, found: 194.1330.

FTIR (thin film, cm^{-1}): 3400, 2947, 2663, 1607, 1515, 1230.

Optical rotation (for the HCl salt): $[\alpha]_D^{26}$ +4.4 (*c* 1.0, $CHCl_3$).

GC (for the free base): Cyclodex-B column, 110 °C isotherm, 1 mL/min.

Racemic Standard (for the free base):

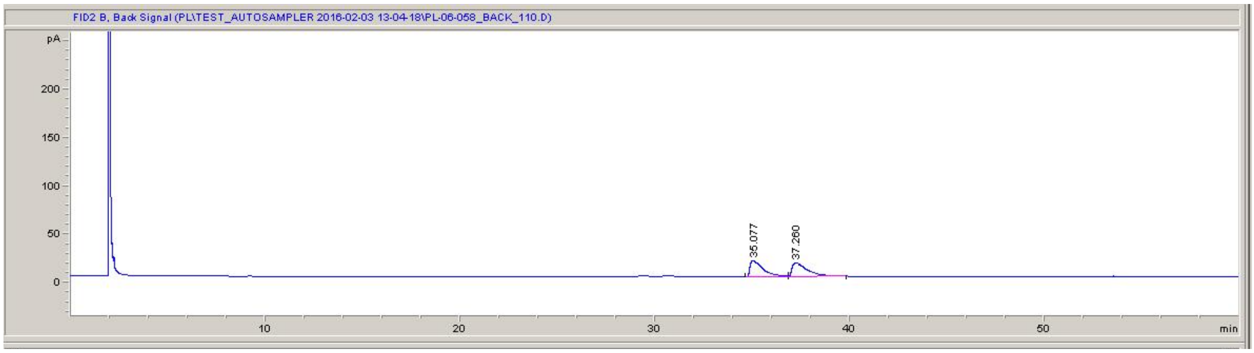

#	Time	Area	Height	Width	Area%	Symmetry
1	35.077	746.6	16.5	0.5914	49.797	0.274
2	37.26	752.7	13.8	0.7037	50.203	0.273

Enantioenriched (for the free base):

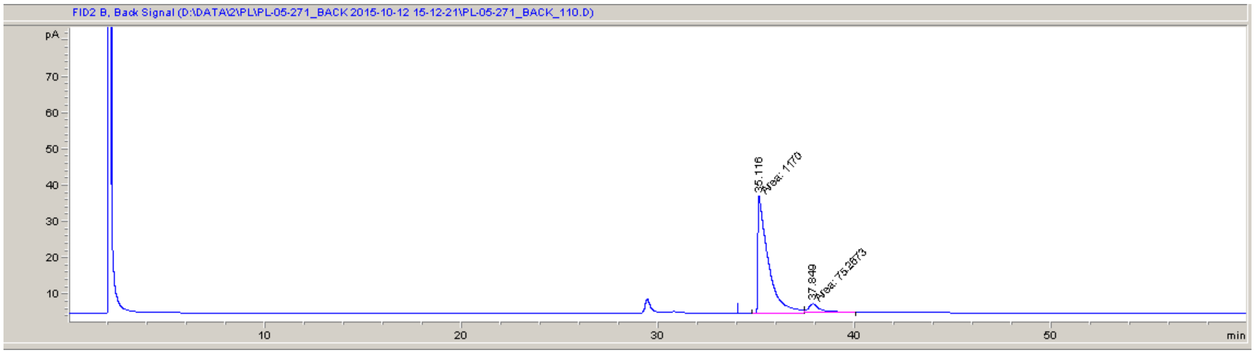

#	Time	Area	Height	Width	Area%	Symmetry
1	35.116	1170	32.2	0.6054	93.956	0.127
2	37.849	75.3	2.2	0.576	6.044	0.64

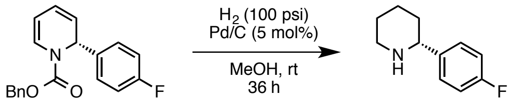

4 **15**

(*R*)-2-(4-Fluorophenyl)piperidine (15): In a 2-dram vial equipped with a stir bar, **4** (110 mg, 0.356 mmol, 1.0 equiv.) was dissolved in MeOH (2 mL). Palladium on carbon (19 mg of 10 wt% catalyst, 0.018 mmol, 5.0 mol%) was added and the vial was capped with a septum cap pierced with an 18 G needle. The vial was transferred to a Parr reactor, which was filled with H_2 (100 psi). The reaction was left to stir at rt for 36 h. The crude reaction mixture was filtered through Celite with Et_2O, and the solvent was removed *in vacuo* to provide **15** (50 mg, 0.28 mmol, 79% yield) as a light yellow solid.

To determine the ee, **15** was subjected to Boc protection with Boc_2O and K_2CO_3. The Boc-protected piperidine was formed in 88% ee, showing that no degradation of ee had occurred during the Cbz deprotection.

^{1}H NMR (500 MHz, $CDCl_3$): δ 7.55 (dd, *J* = 8.5, 5.2 Hz, 2H), 7.02 (d, *J* = 17.1 Hz, 2H), 3.84 (d, *J* = 14.8 Hz, 1H), 3.08 (d, *J* = 13.5 Hz, 1H), 2.75 (t, *J* = 14.4 Hz, 1H), 2.20–1.85 (m, 4H), 1.74 (d, *J* = 12.6 Hz, 1H), 1.53 (dt, *J* = 13.0, 3.4 Hz, 1H), 1.35–1.06 (m, 1H) ppm.

^{13}C NMR (125 MHz, $CDCl_3$): δ 163.01 (d, *J* = 248.4 Hz), 133.23, 129.99 (d, *J* = 8.3 Hz), 115.98 (d, *J* = 21.7 Hz), 60.83, 45.90, 30.90, 23.52, 22.15 ppm.

^{19}F NMR (282 MHz, $CDCl_3$): δ –112.29 ppm

HRMS: (ESI-TOF) calculated for $C_{11}H_{15}FN$ ($[M+H]^+$): 180.1183, found: 180.1176.

FTIR (thin film, cm^{-1}): 3395, 2935, 2810, 1606, 1514, 1227.

Optical rotation (for the unprotected piperidine): $[\alpha]_D^{26}$ +8.7 (*c* 1.1, $CHCl_3$).

HPLC (for the Boc-Protected Piperidine): Chiralpak AD-H, 5% IPA in hexanes, 10 min run, 1 mL/min.

Racemic Standard (for the Boc-protected piperidine):

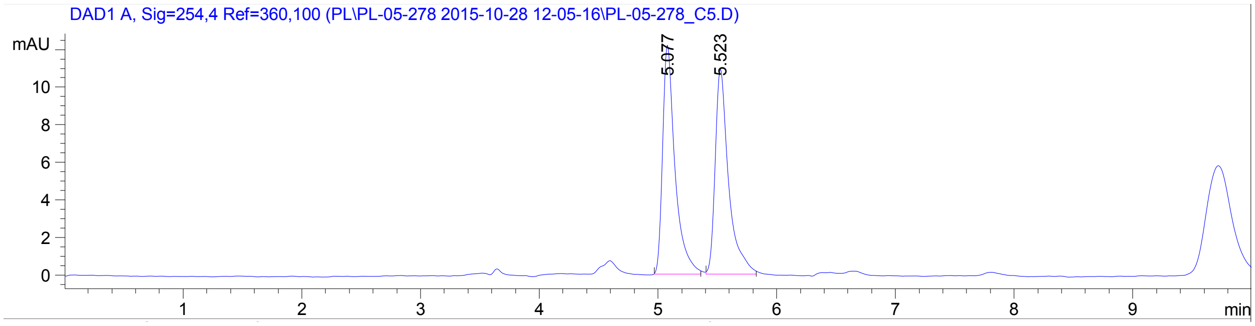

Peak #	RetTime [min]	Type	Width [min]	Area [mAU*s]	Height [mAU]	Area %
1	5.077	BB	0.1072	89.16976	12.21269	50.7411
2	5.523	BB	0.1162	86.56518	10.95045	49.2589

Totals : 175.73494 23.16314

Enantioenriched (for the Boc-protected piperidine):

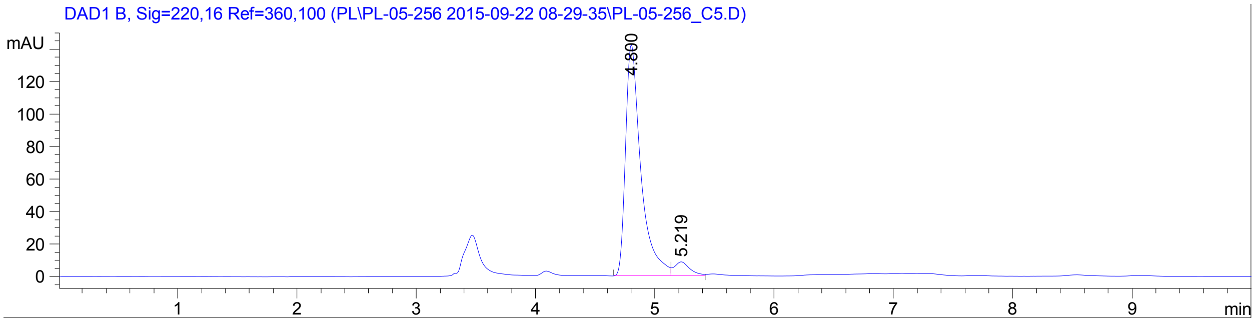

Peak #	RetTime [min]	Type	Width [min]	Area [mAU*s]	Height [mAU]	Area %
1	4.800	BV	0.1325	1275.18359	142.41821	94.1833
2	5.219	VV	0.1325	78.75392	8.63079	5.8167

Totals : 1353.93752 151.04900

VII.VT-NMR Studies of 5a

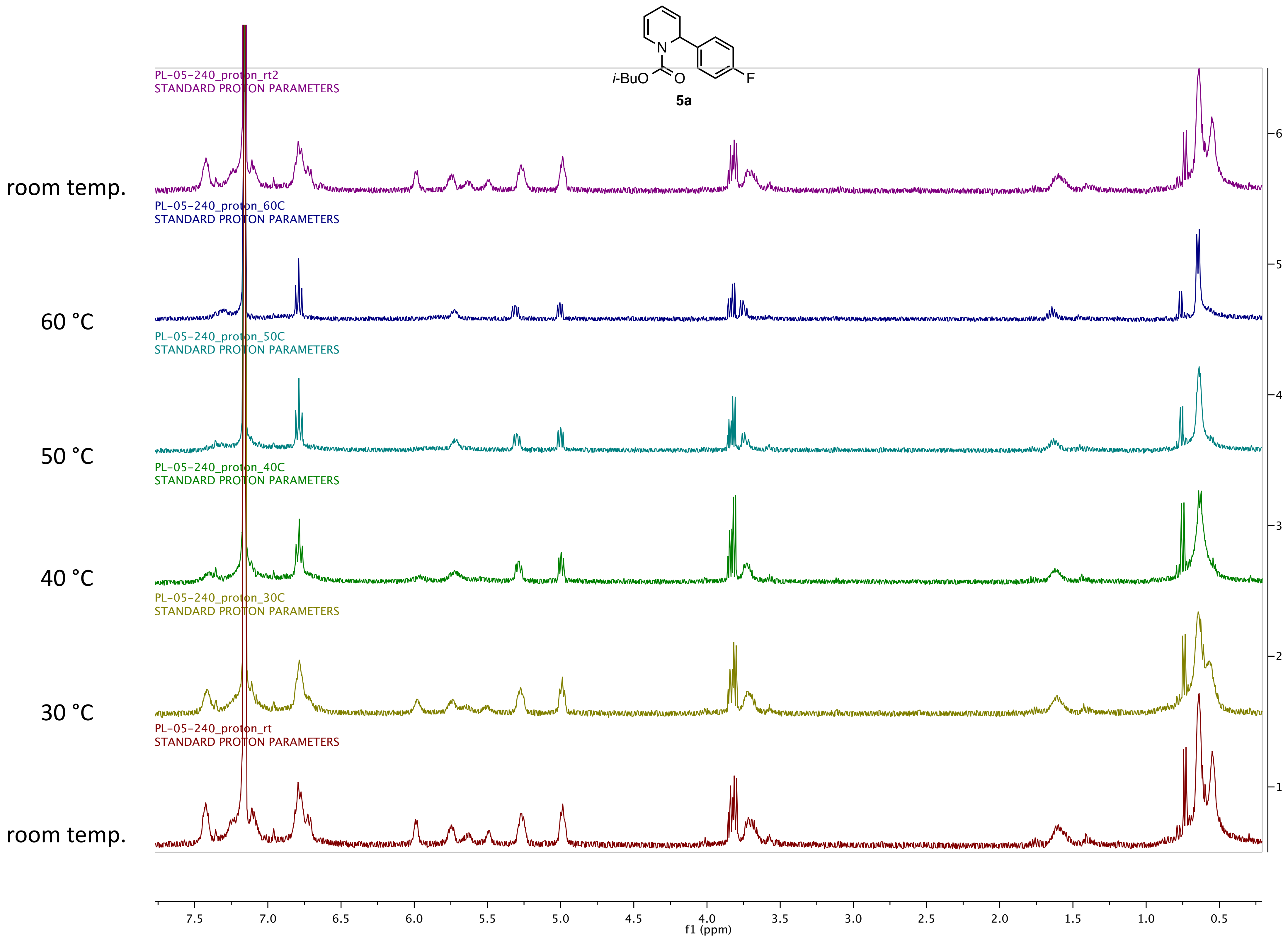

400 MHz ^{1}H-VT-NMR spectra of **5a** in C_6D_6.

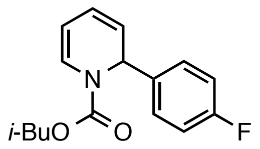

5a

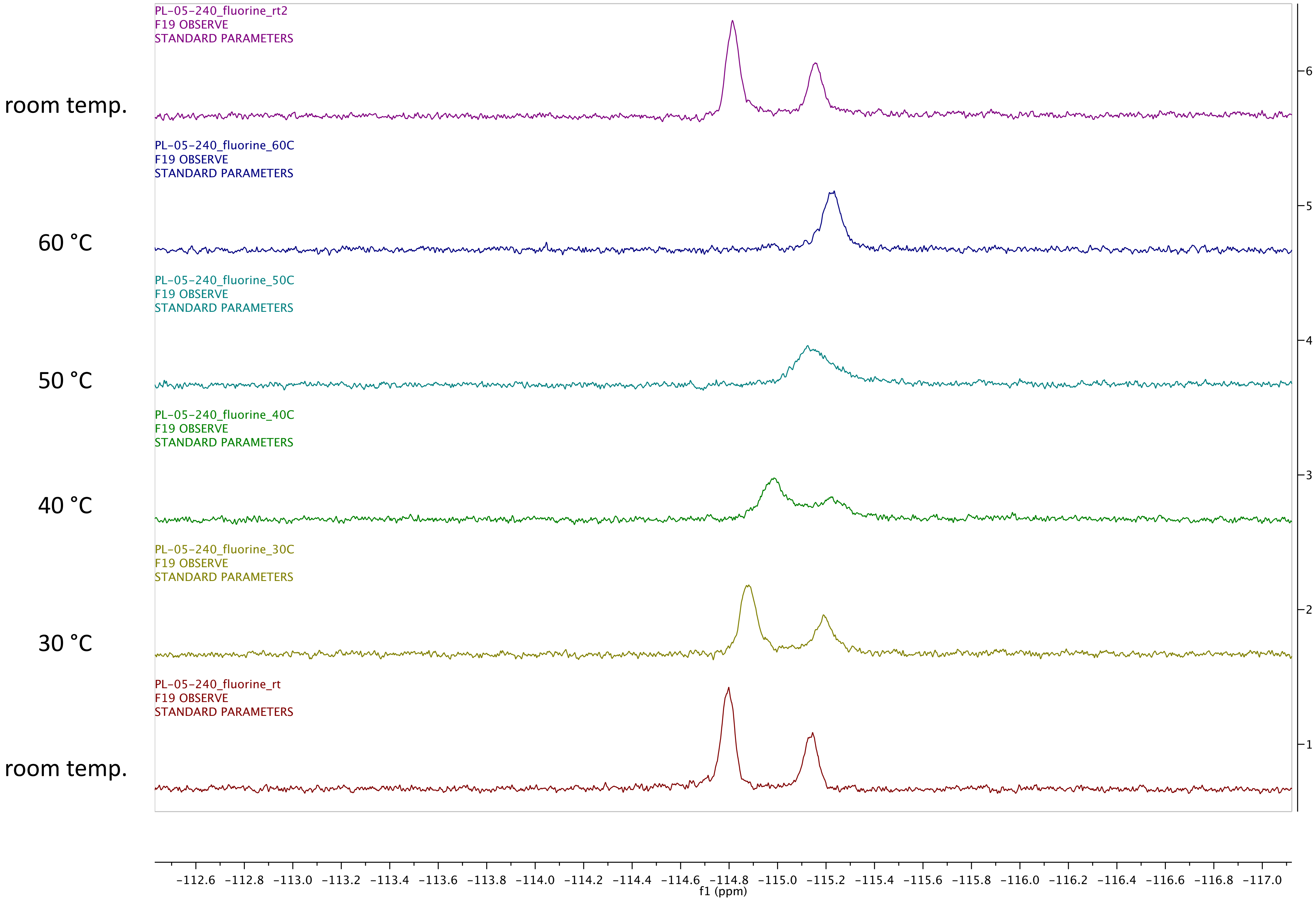

376 MHz ^{19}F-VT-NMR spectra of **5a** in C_6D_6.

VIII. Crude NMR of 5a

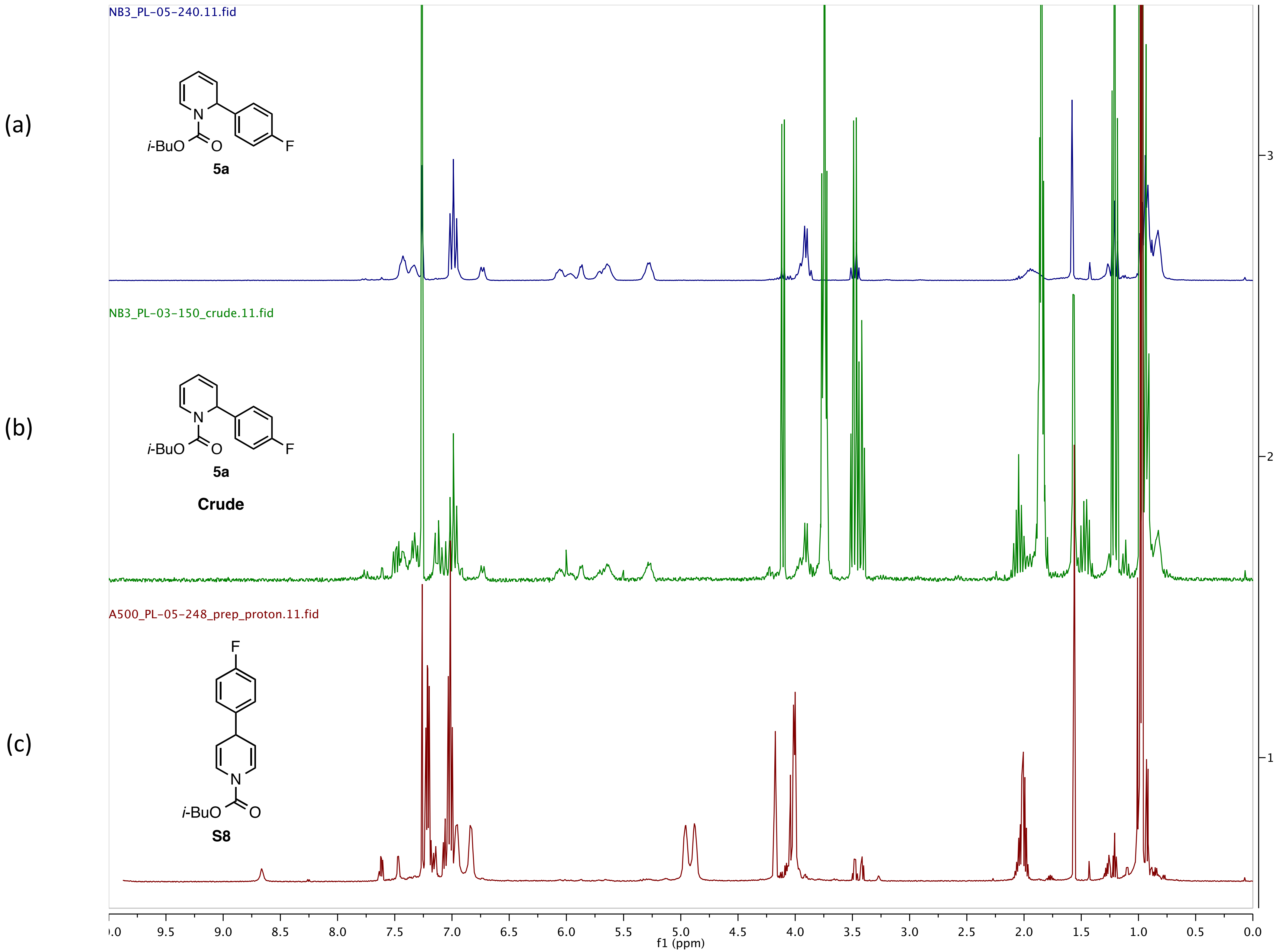

(a) 300 MHz ^{1}H-NMR spectrum of **5a** in $CDCl_3$. (b) 300 MHz ^{1}H-NMR spectrum of crude **5a** in $CDCl_3$. (c) 500 MHz ^{1}H-NMR spectrum of **S8** in $CDCl_3$.

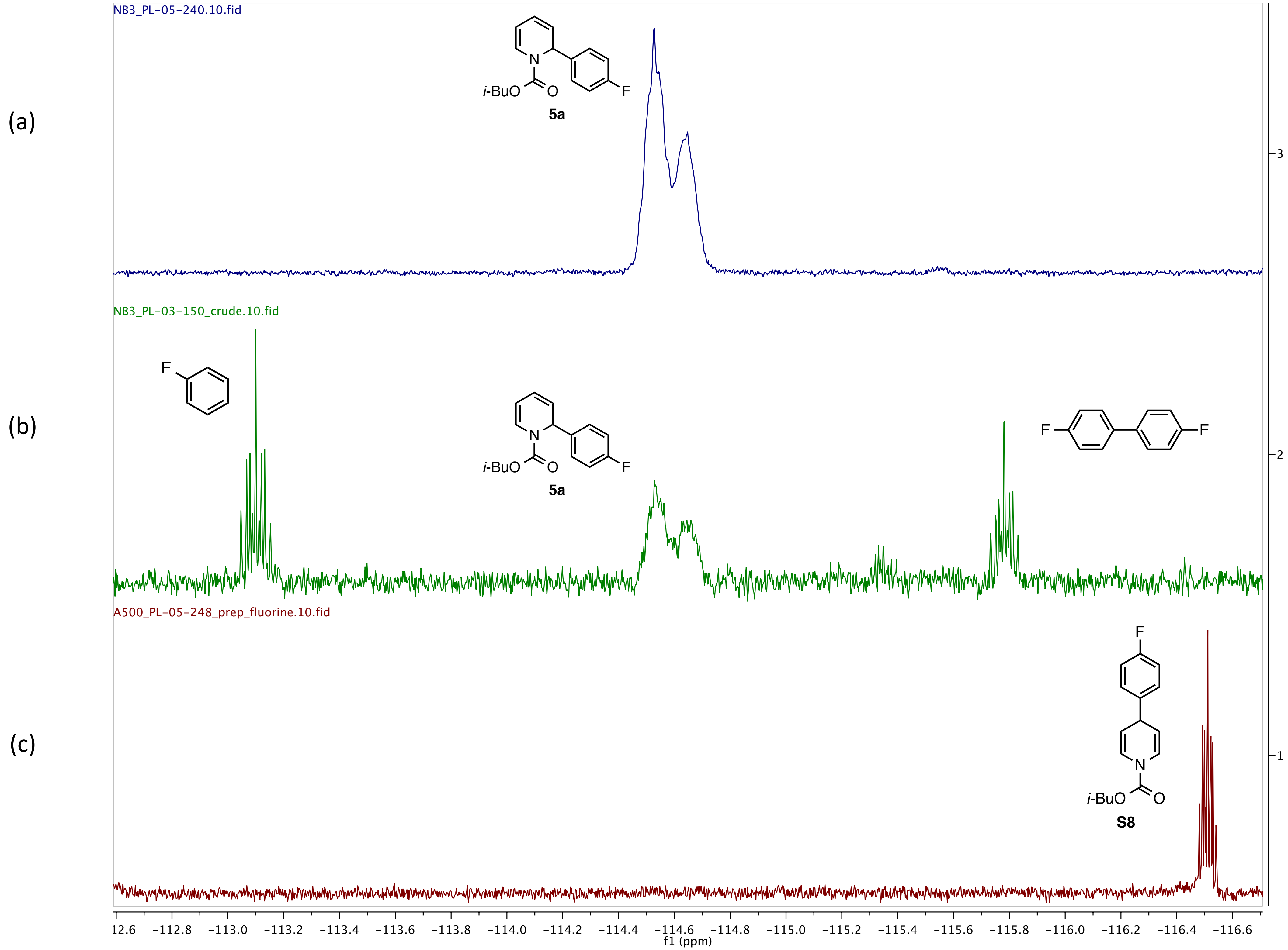

(a) 282 MHz ^{19}F-NMR spectrum of **5a** in $CDCl_3$. (b) 282 MHz ^{19}F-NMR spectrum of crude **5a** in $CDCl_3$. (c) 470 MHz ^{19}F-NMR spectrum of **S8** in $CDCl_3$.

IX. NMR Spectra

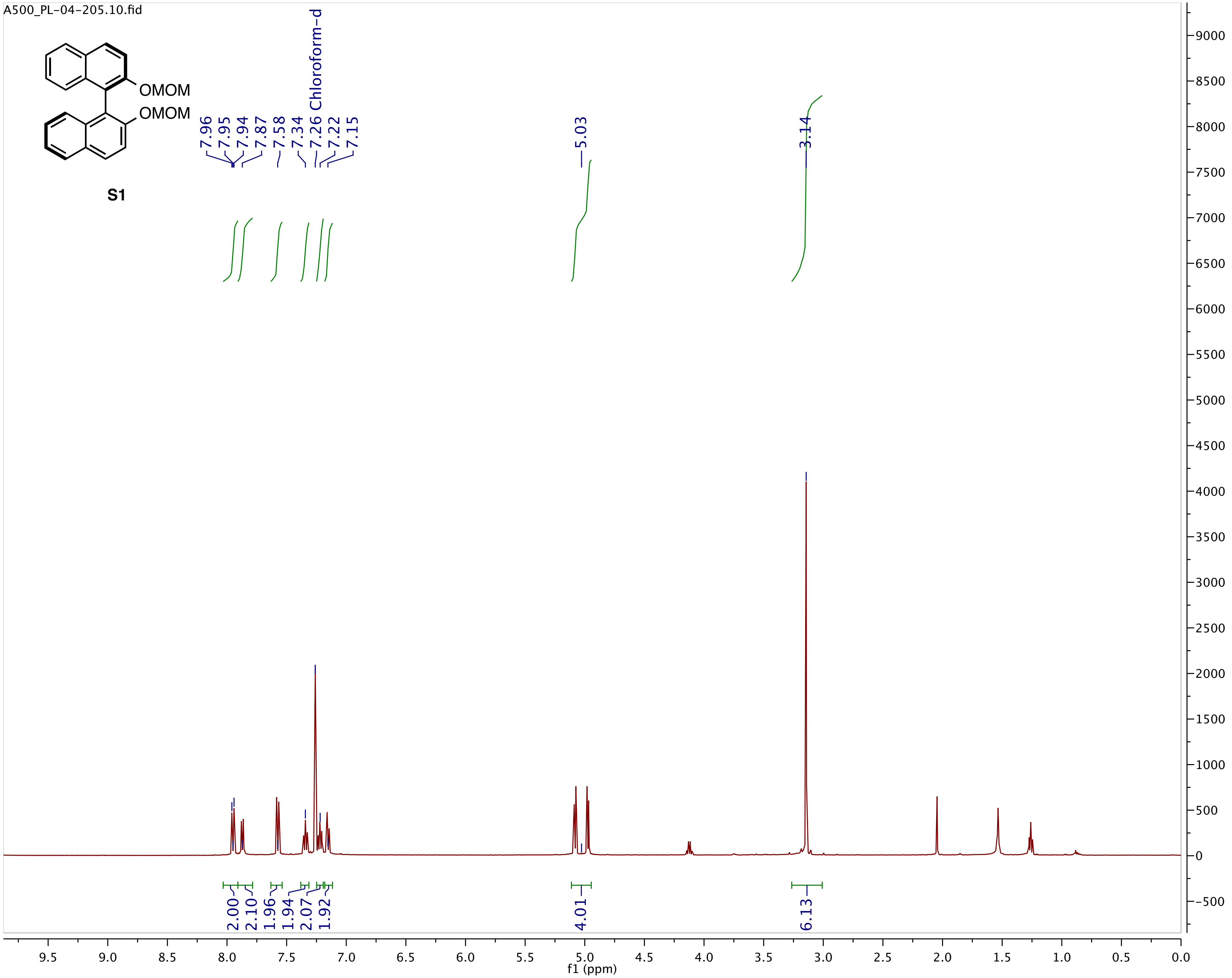

500 MHz ¹H-NMR spectrum of **S1** in $CDCl_3$

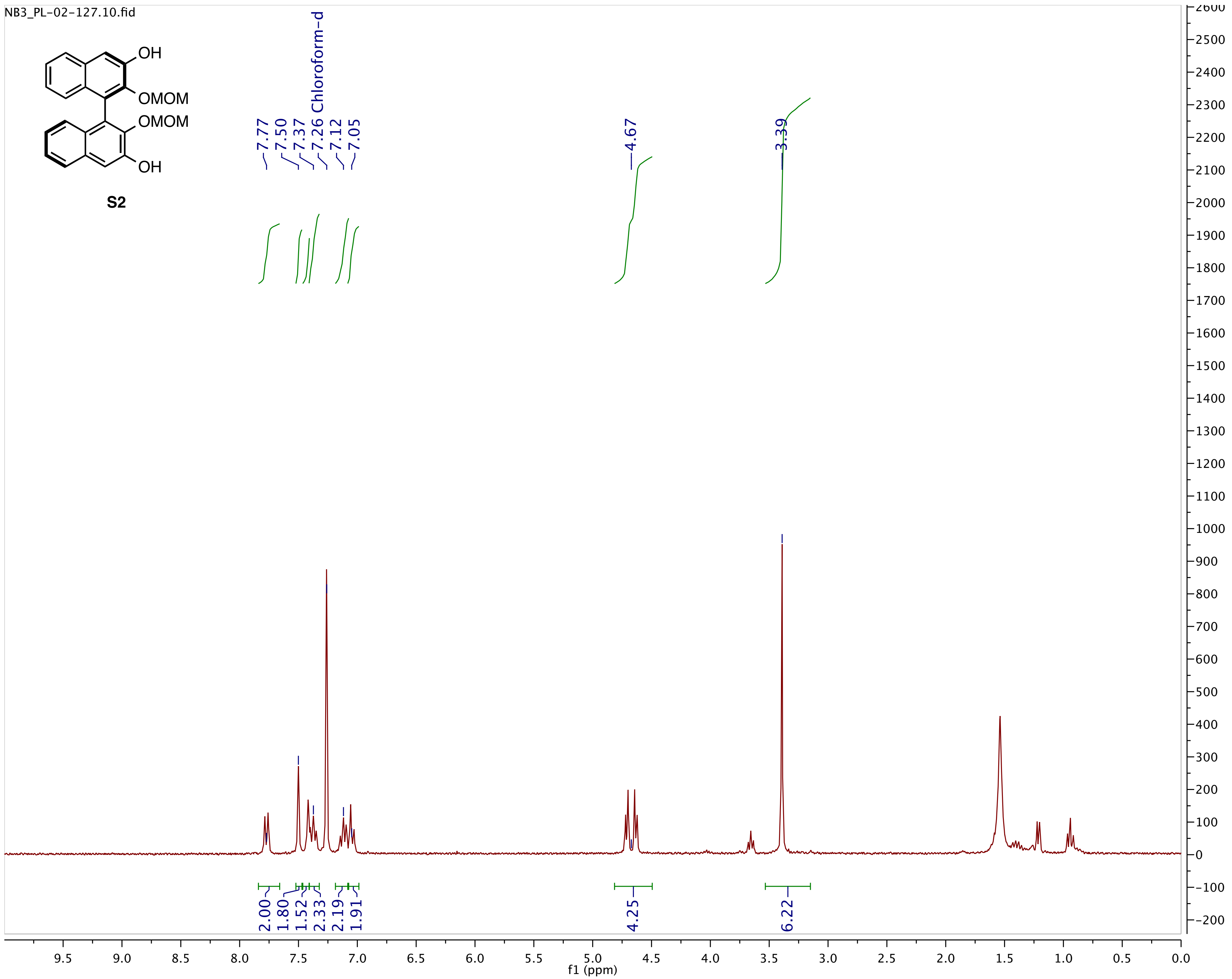

300 MHz ¹H-NMR spectrum of crude **S2** in $CDCl_3$

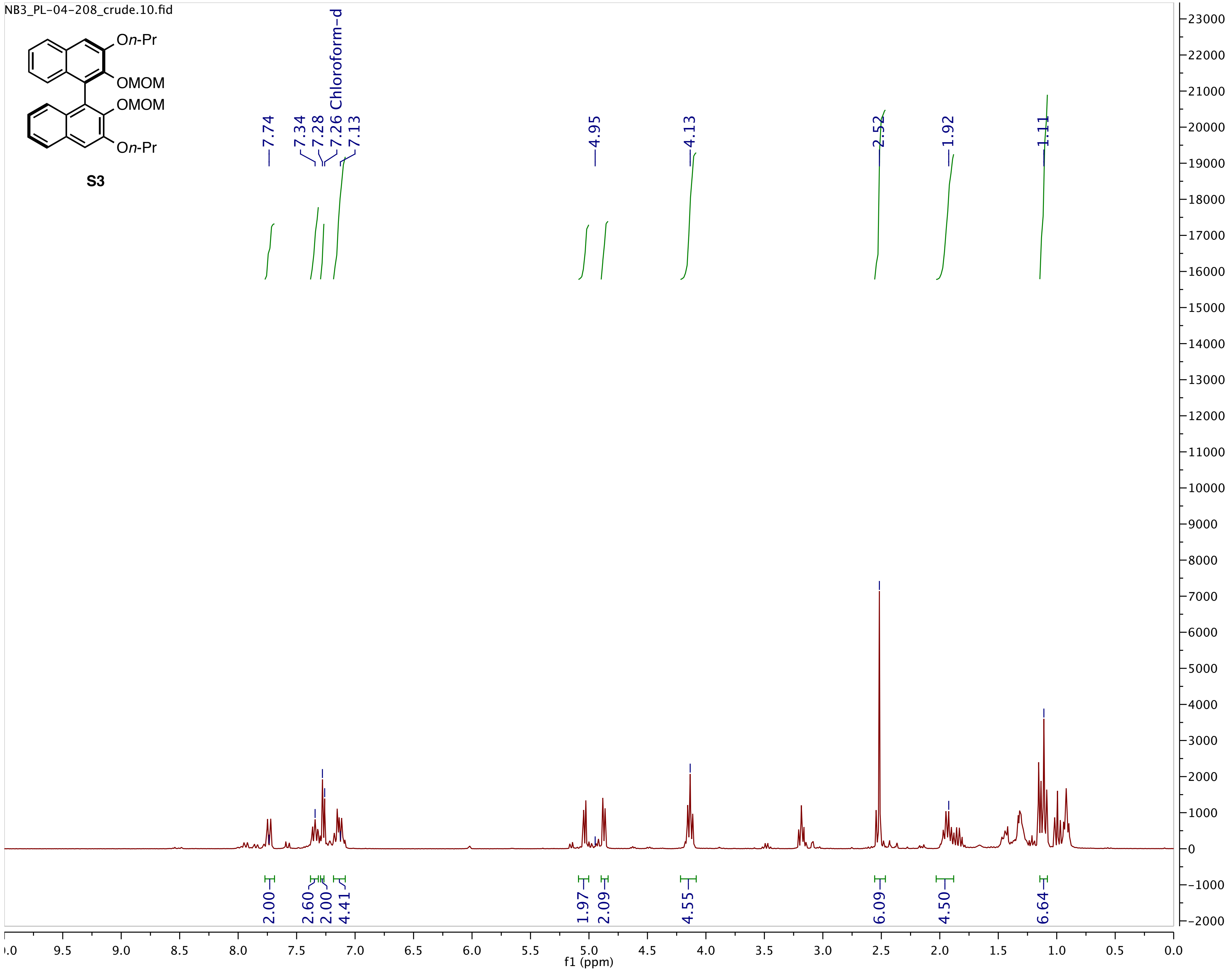

300 MHz ^{1}H-NMR spectrum of crude **S3** in $CDCl_3$

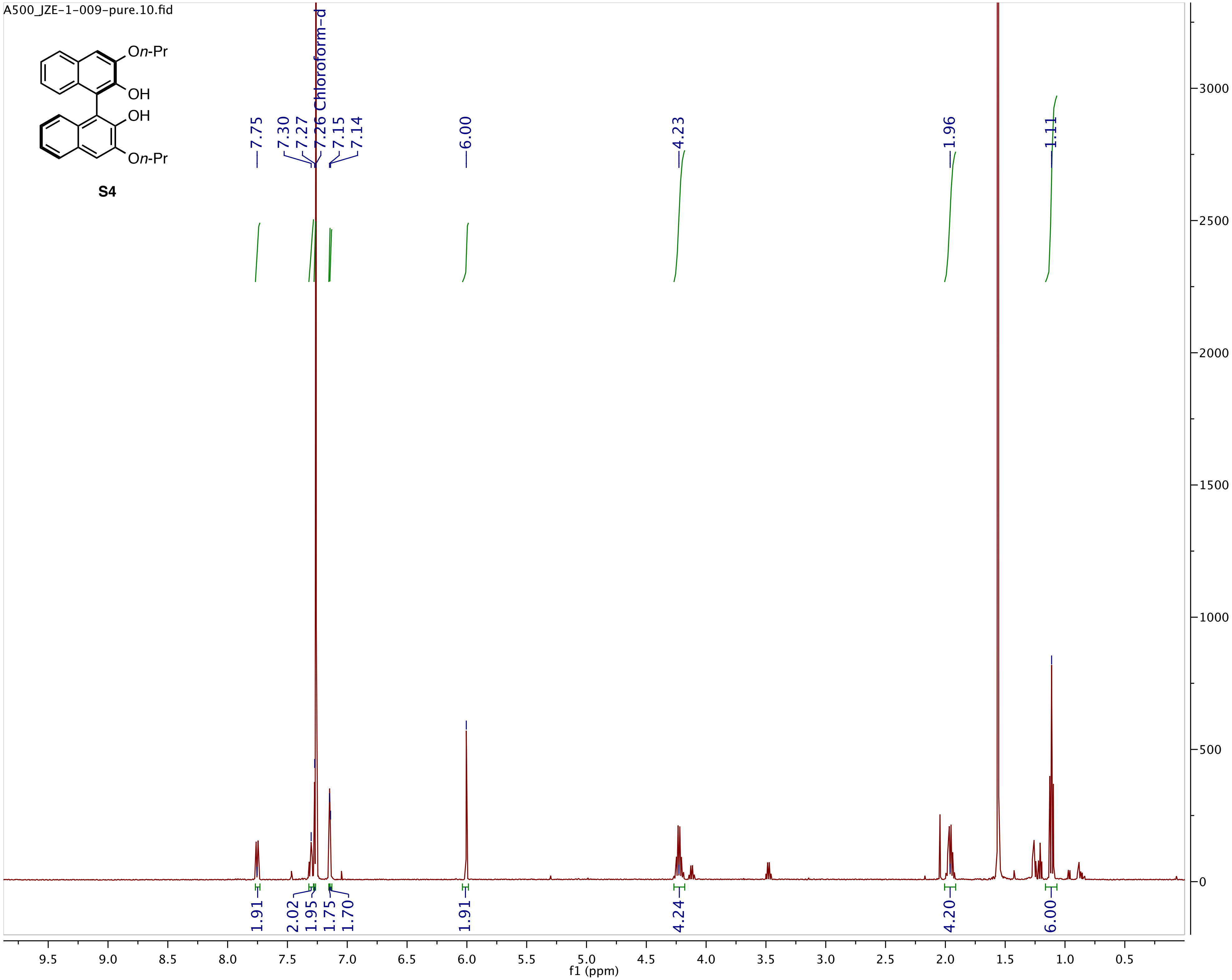

500 MHz ^{1}H-NMR spectrum of **S4** in $CDCl_3$

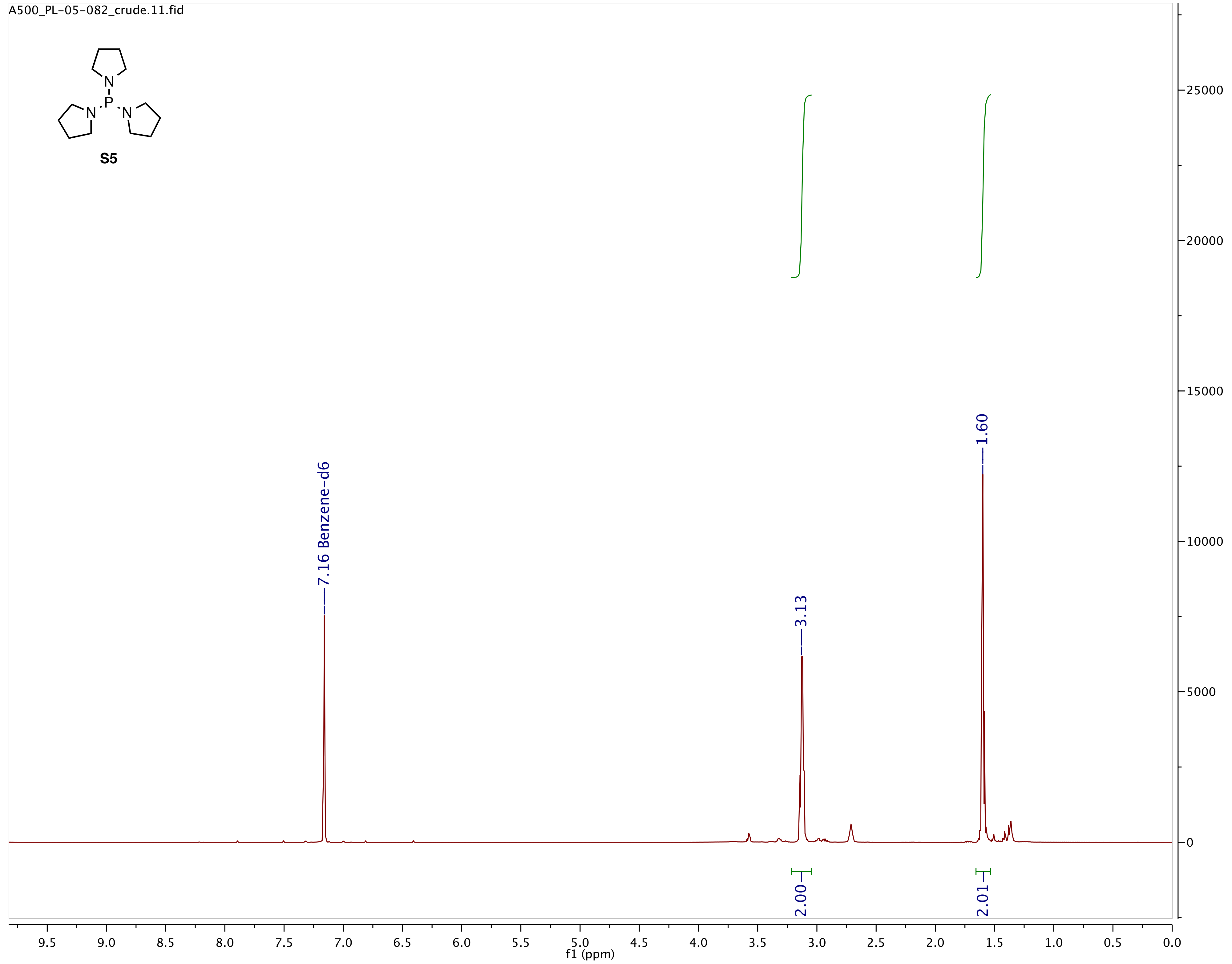

500 MHz ^{1}H-NMR spectrum of crude **S5** in C_6D_6

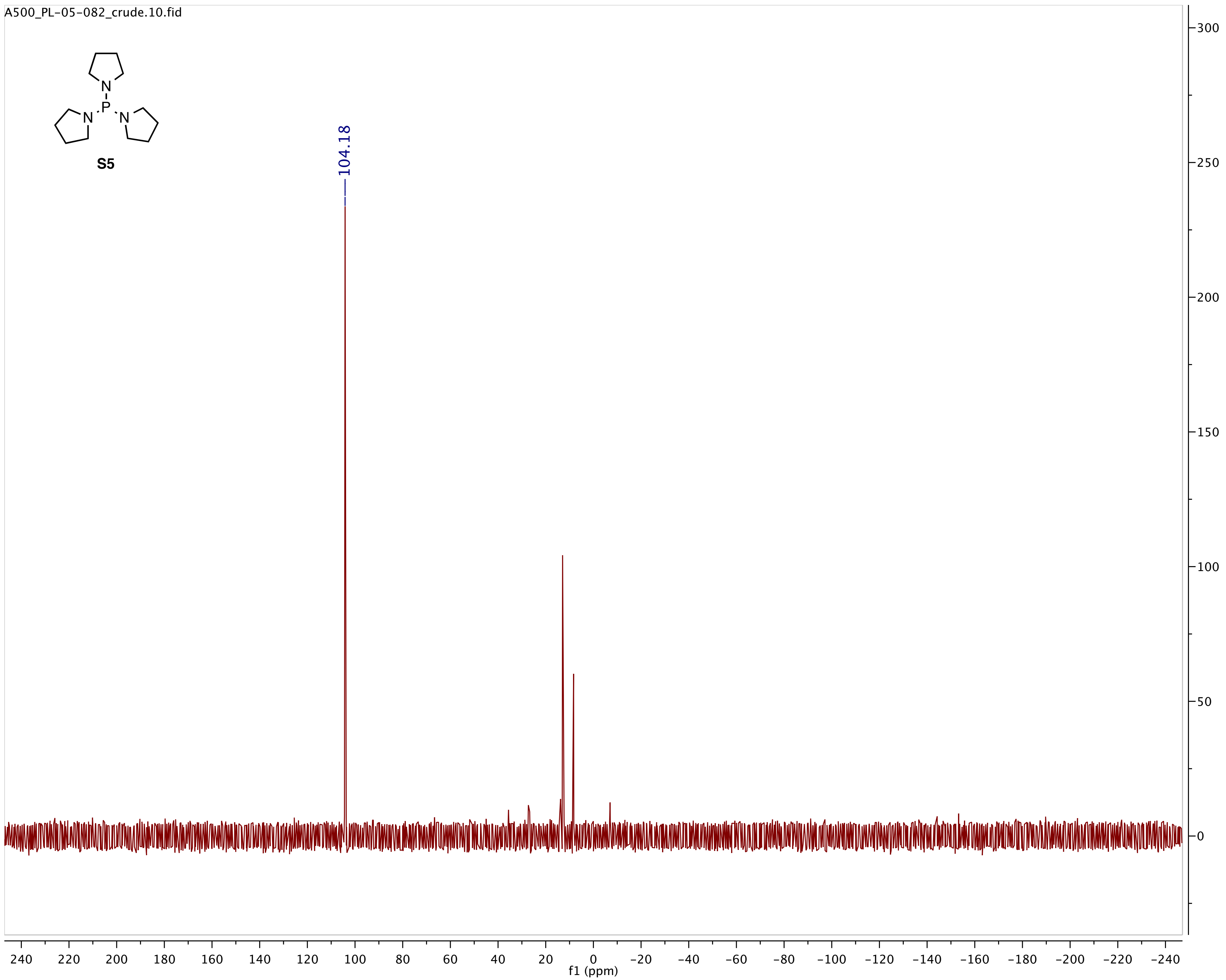

121 MHz ^{31}P-NMR spectrum of crude **S5** in C_6D_6

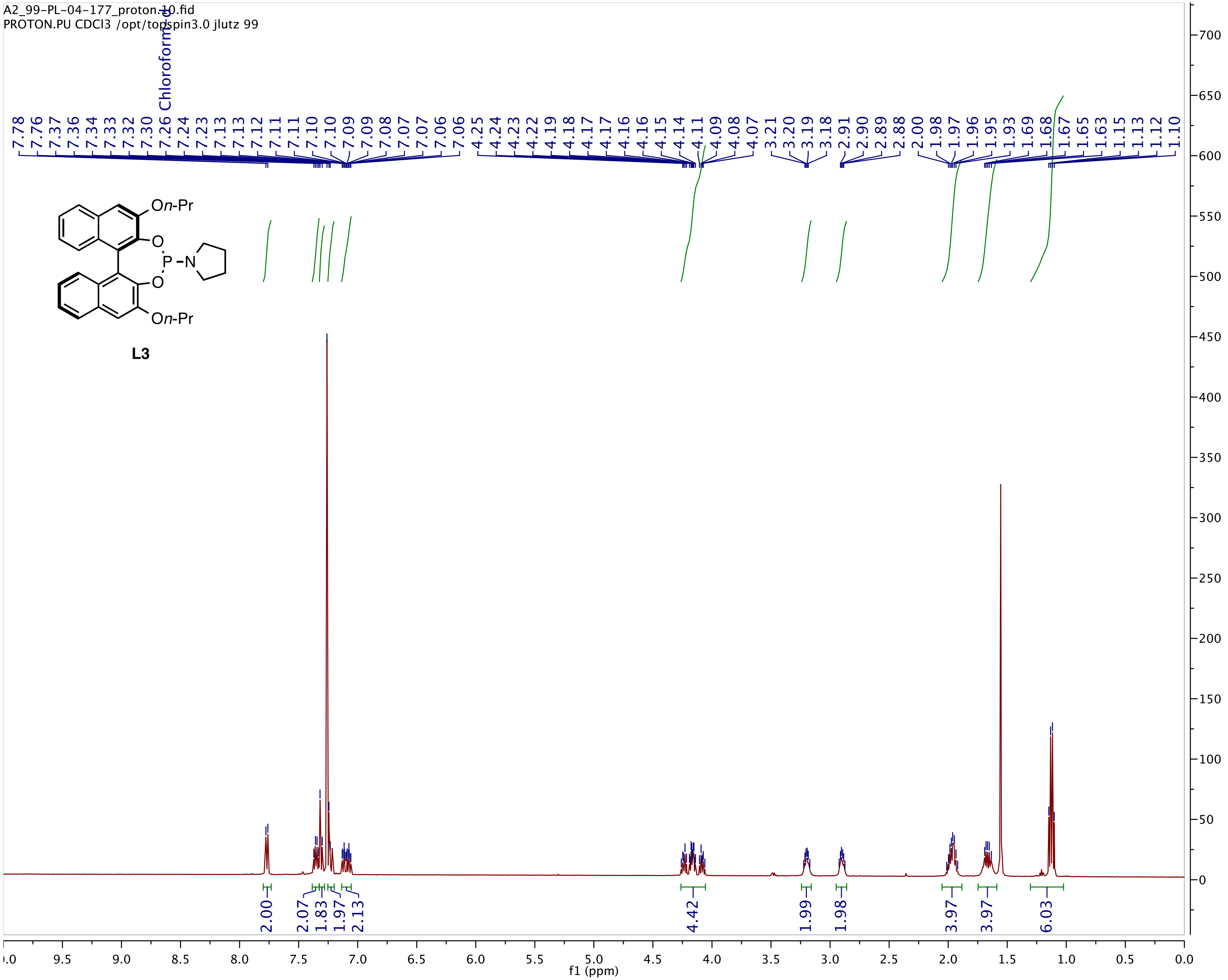

500 MHz ^{1}H-NMR spectrum of **L3** in $CDCl_3$

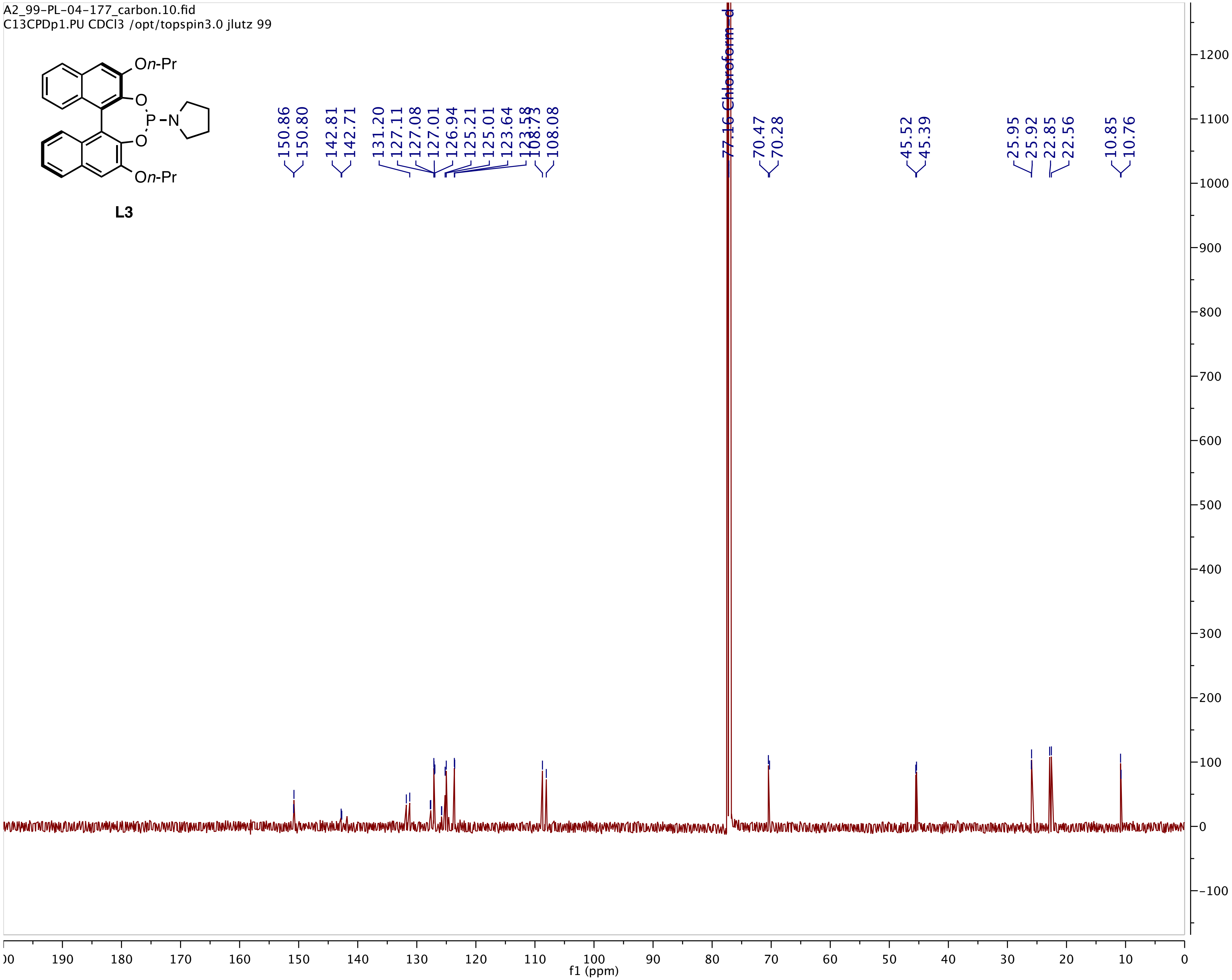

125 MHz ^{13}C-NMR spectrum of **L3** in $CDCl_3$

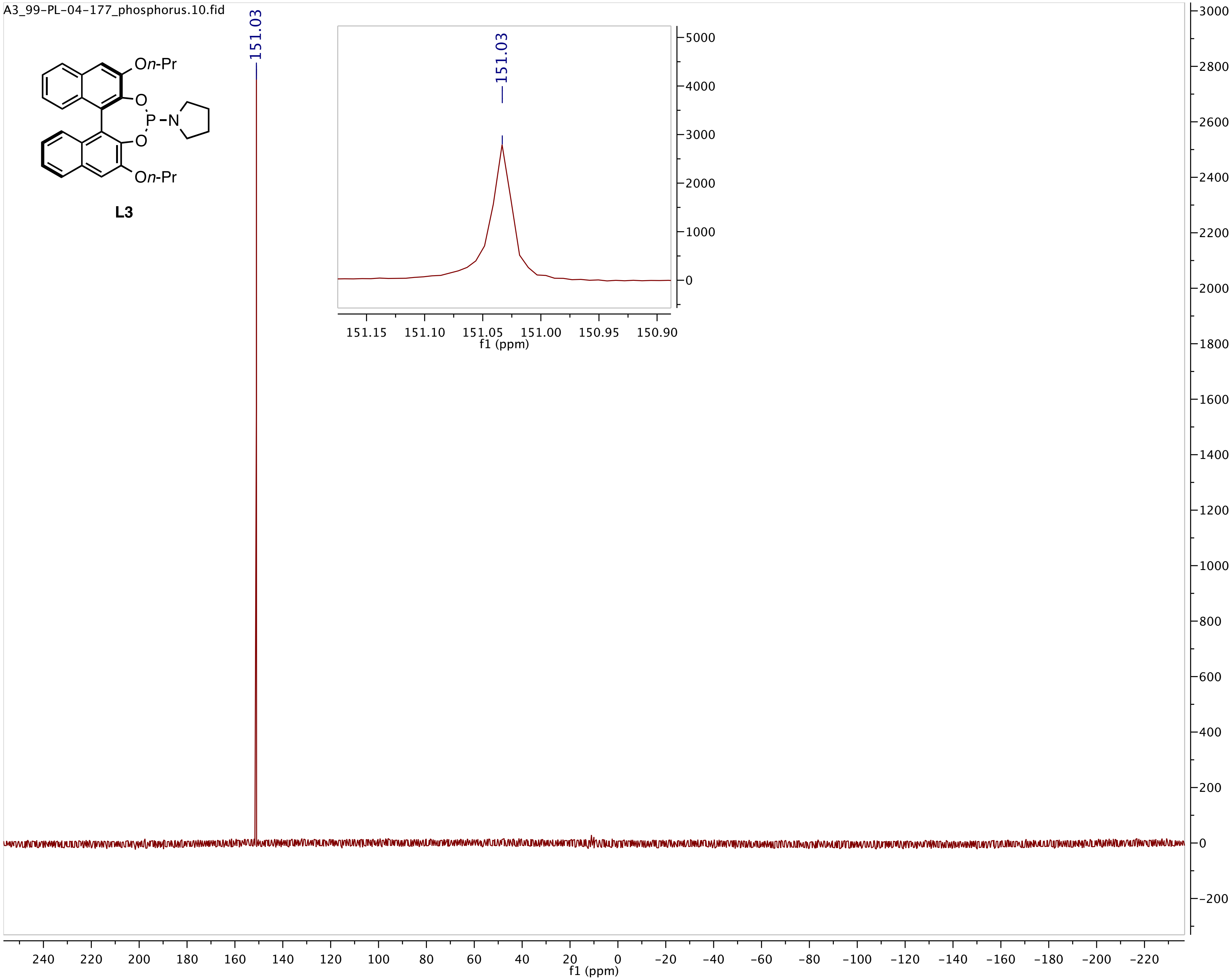

121 MHz ^{31}P-NMR spectrum of **L3** in $CDCl_3$

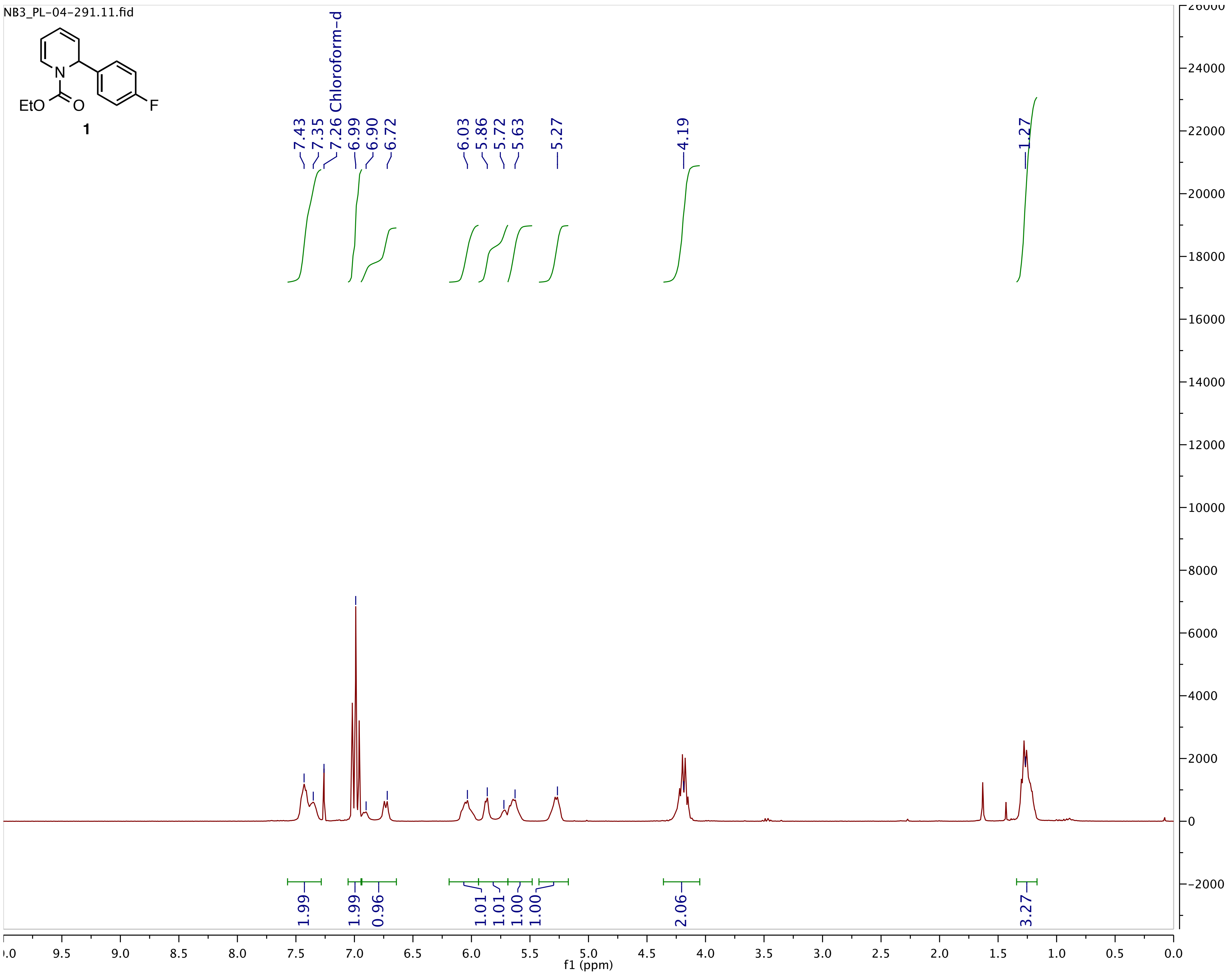

300 MHz ^{1}H-NMR spectrum of **1** in $CDCl_3$

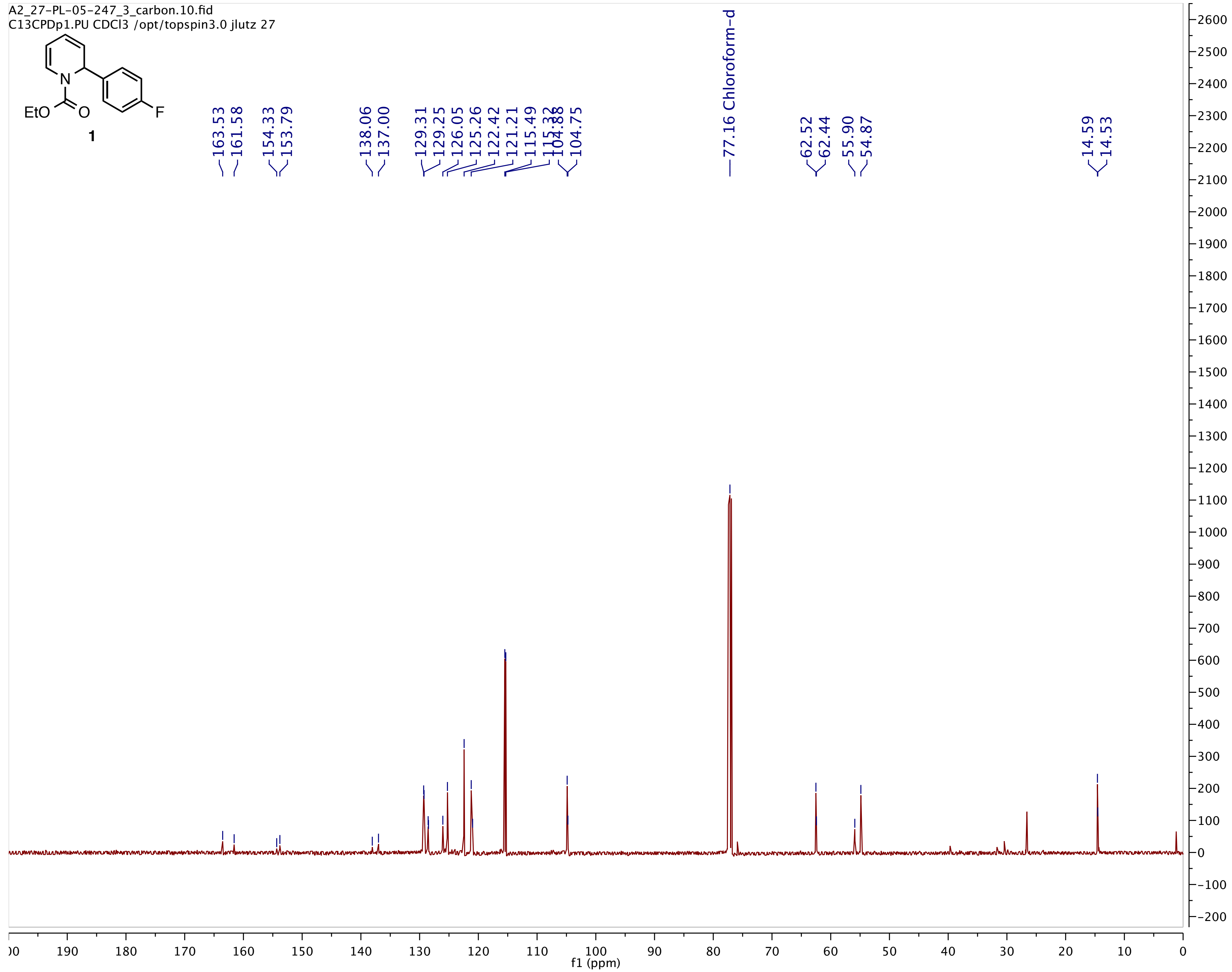

125 MHz ^{13}C-NMR spectrum of **1** in $CDCl_3$

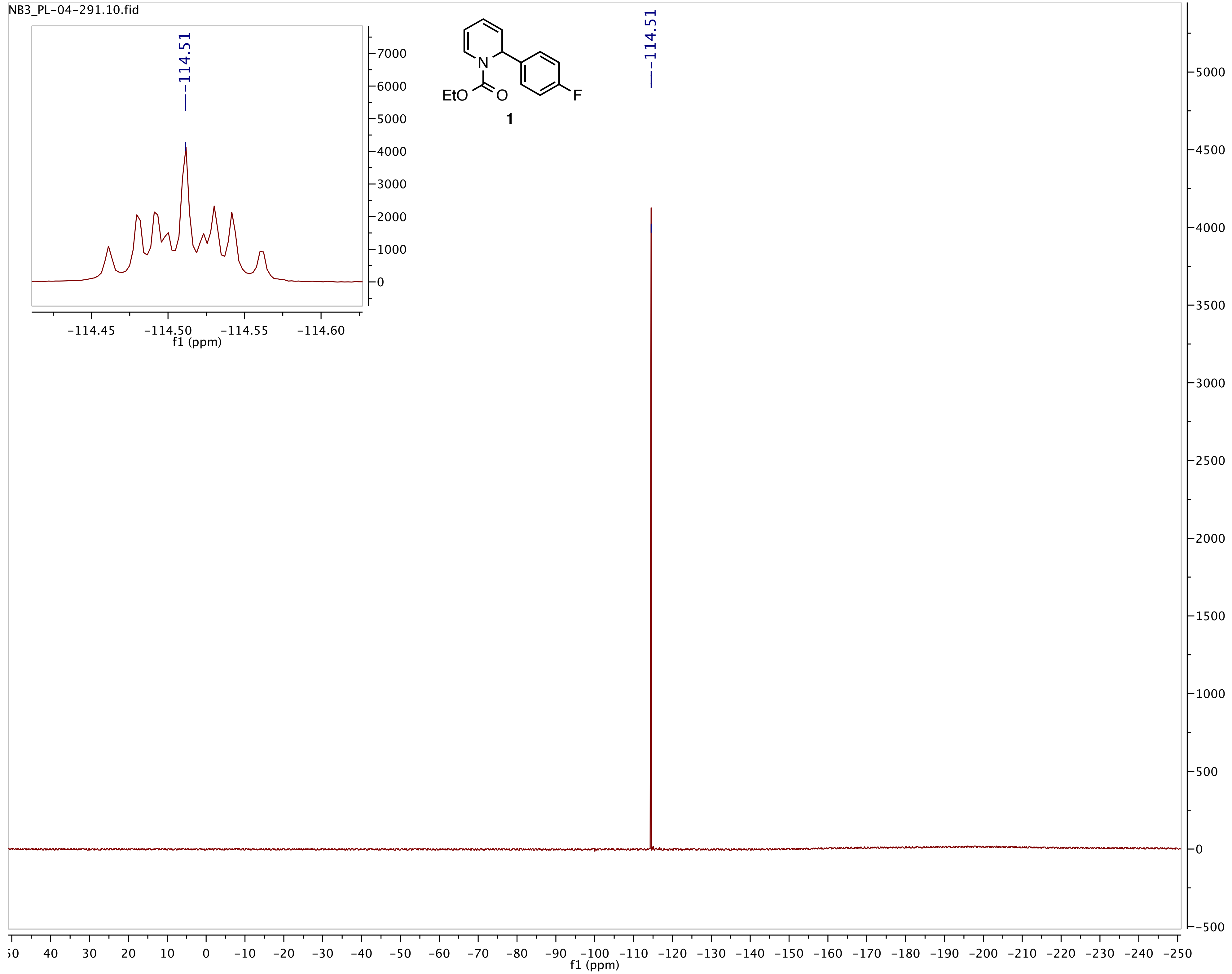

282 MHz ^{19}F-NMR spectrum of **1** in $CDCl_3$

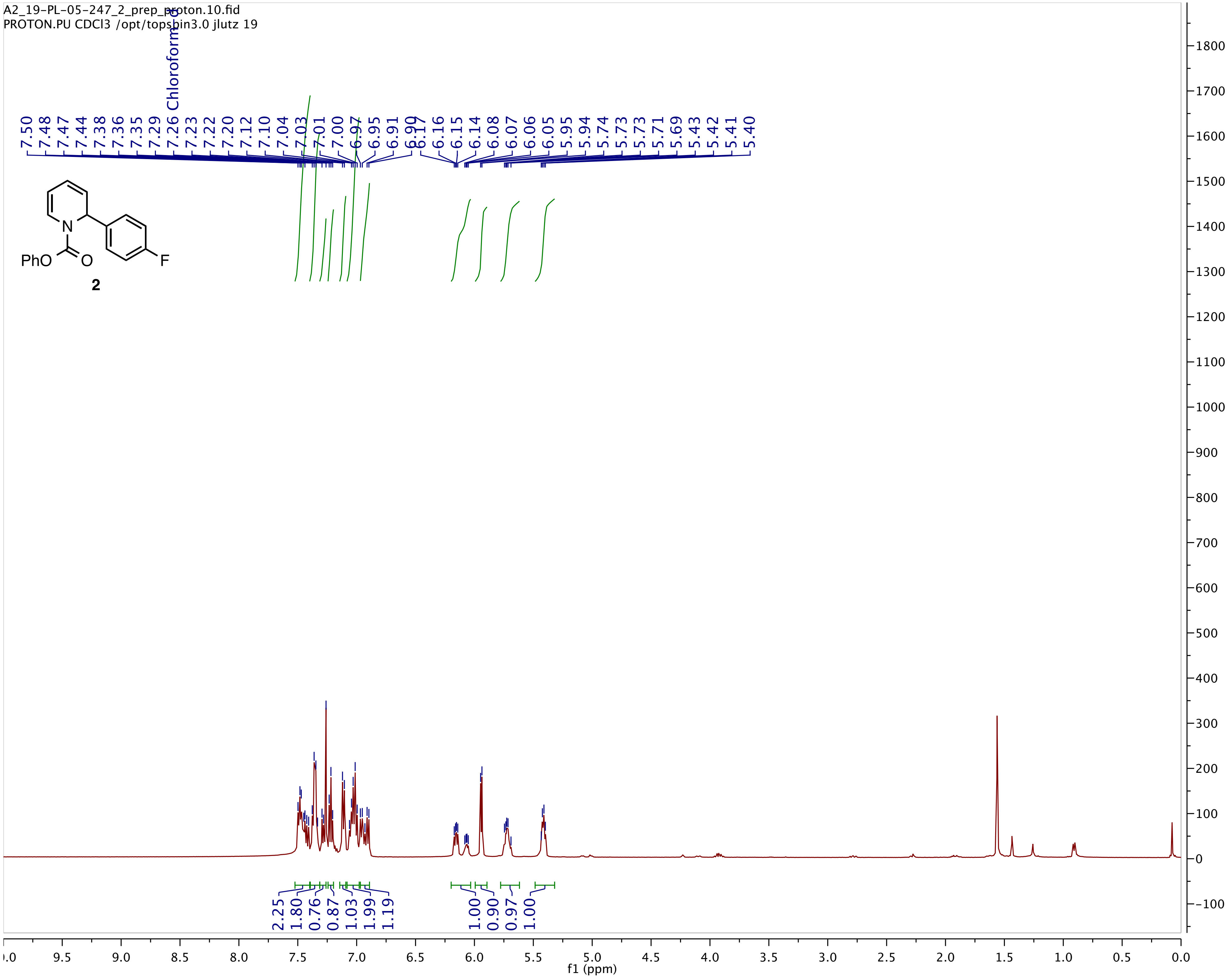

500 MHz ^{1}H-NMR spectrum of **2** in $CDCl_3$

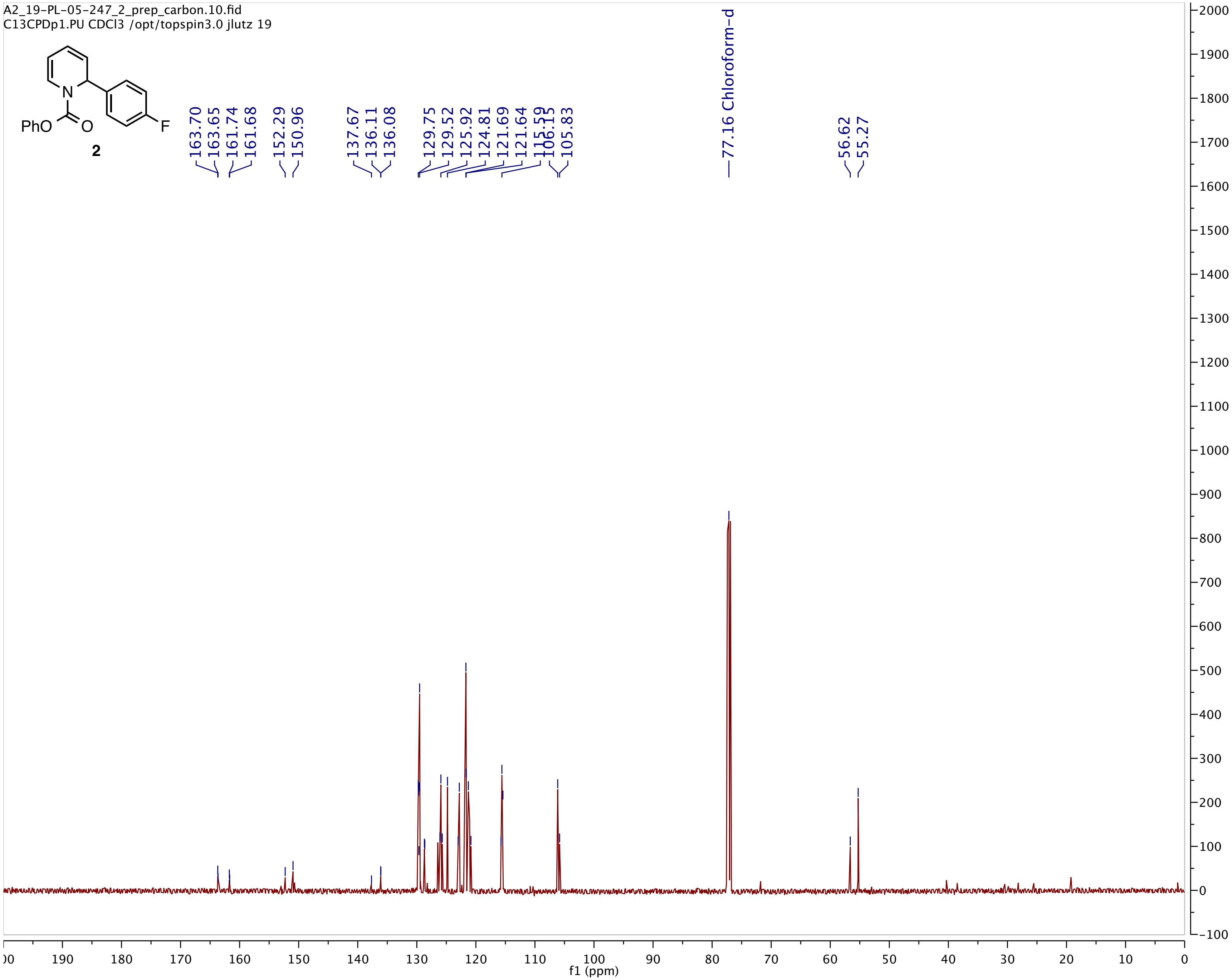

125 MHz ^{13}C-NMR spectrum of **2** in $CDCl_3$

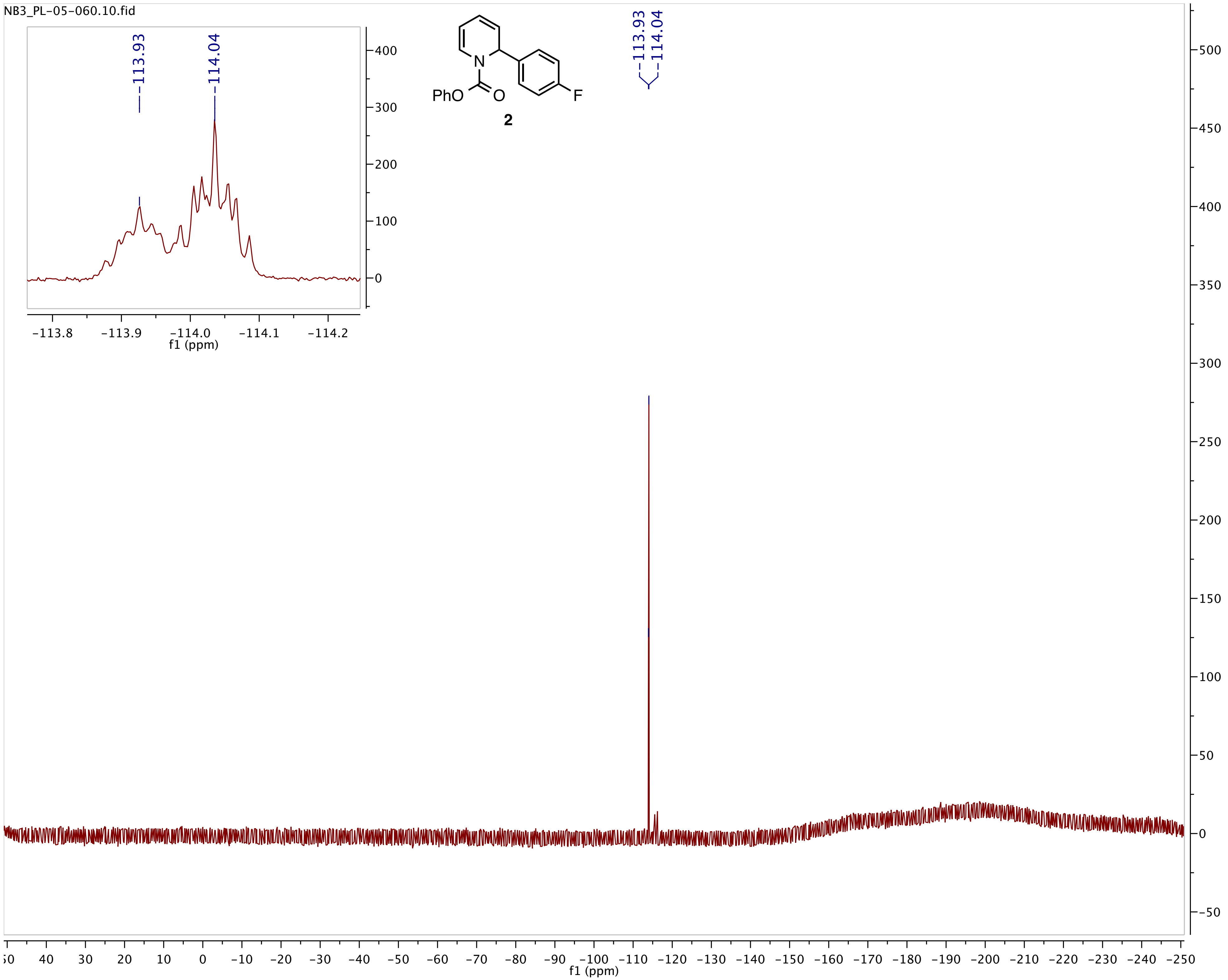

282 MHz ^{19}F-NMR spectrum of **2** in $CDCl_3$

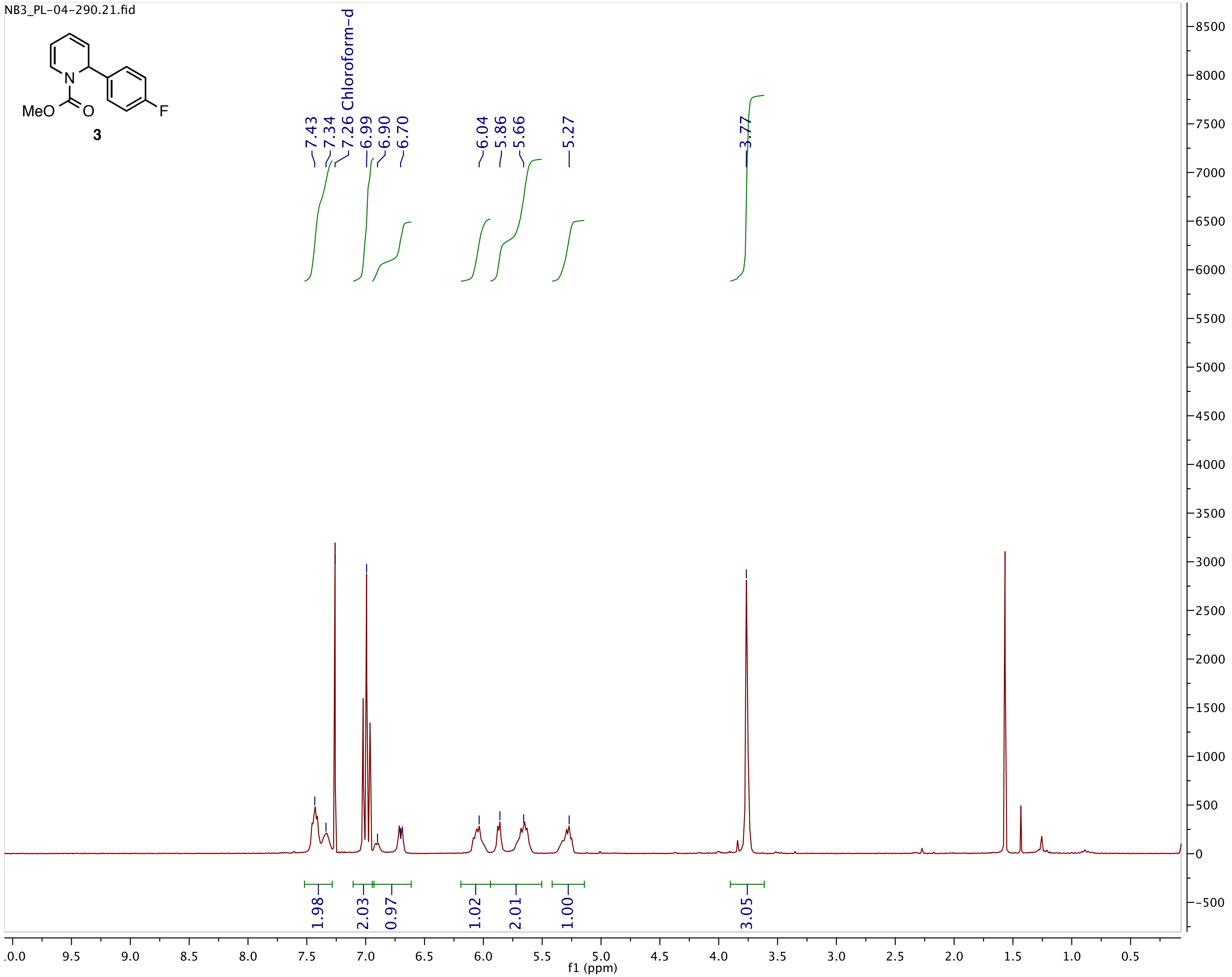

300 MHz ^{1}H-NMR spectrum of **3** in $CDCl_3$

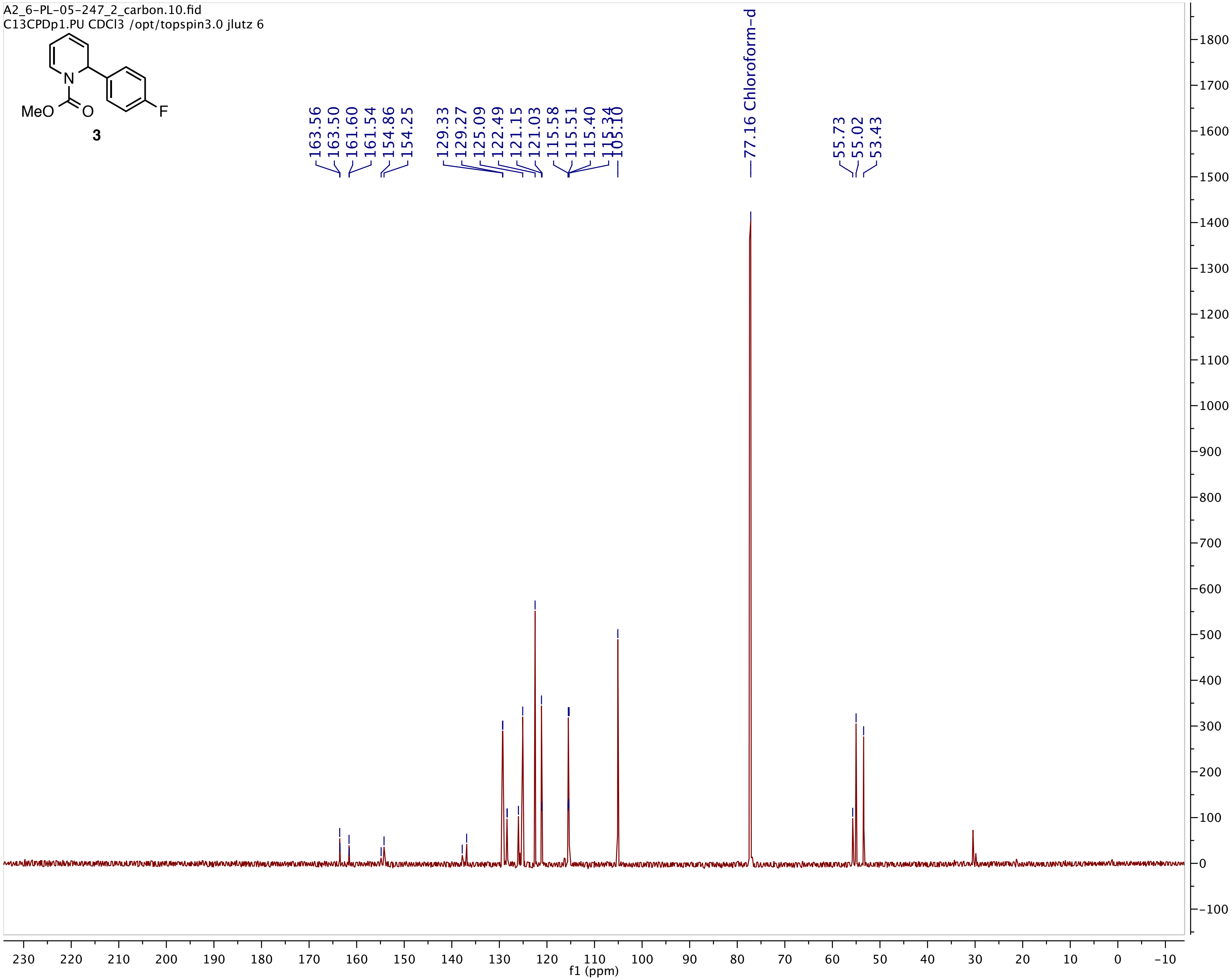

125 MHz ^{13}C-NMR spectrum of **3** in $CDCl_3$

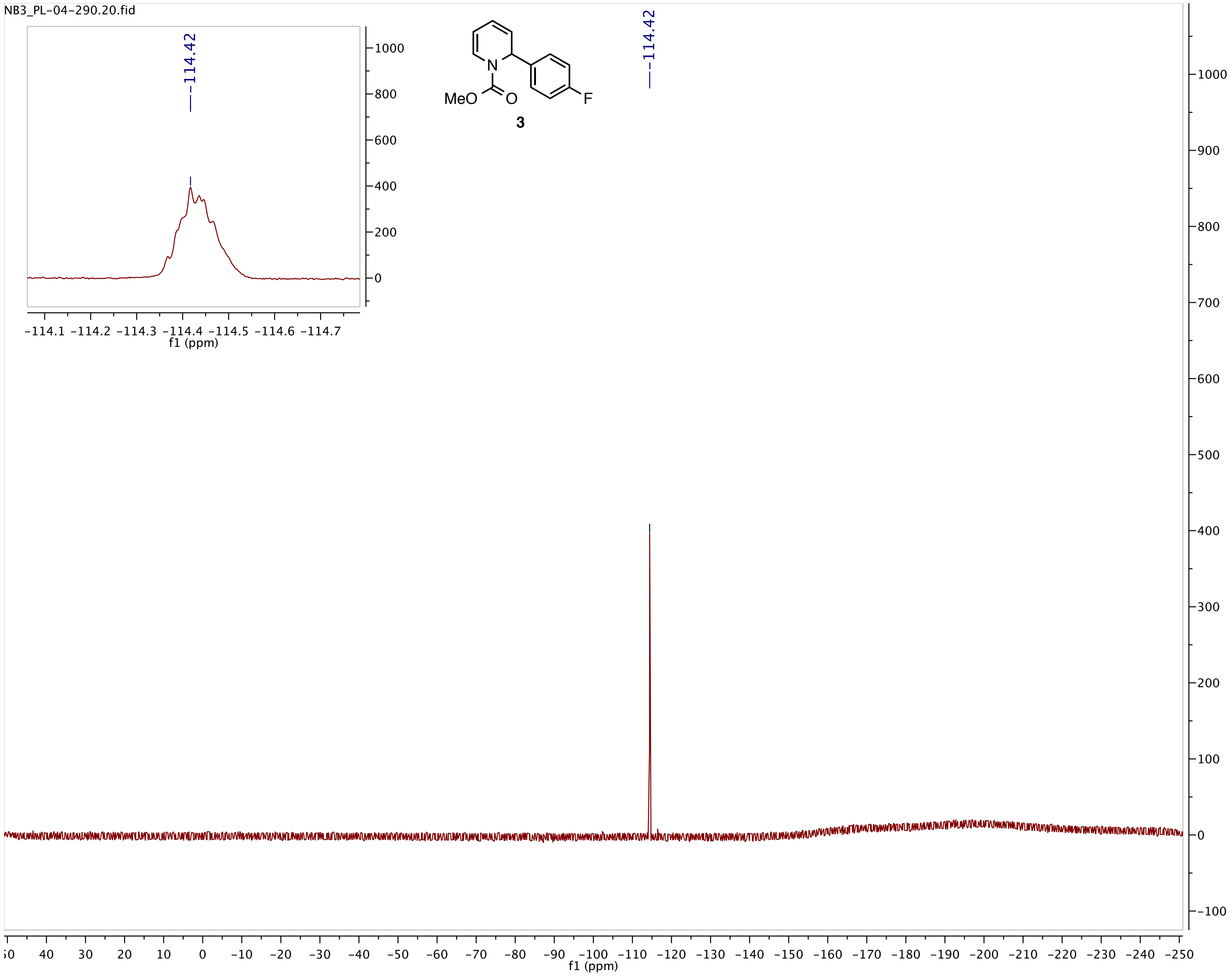

282 MHz ^{19}F-NMR spectrum of **3** in $CDCl_3$

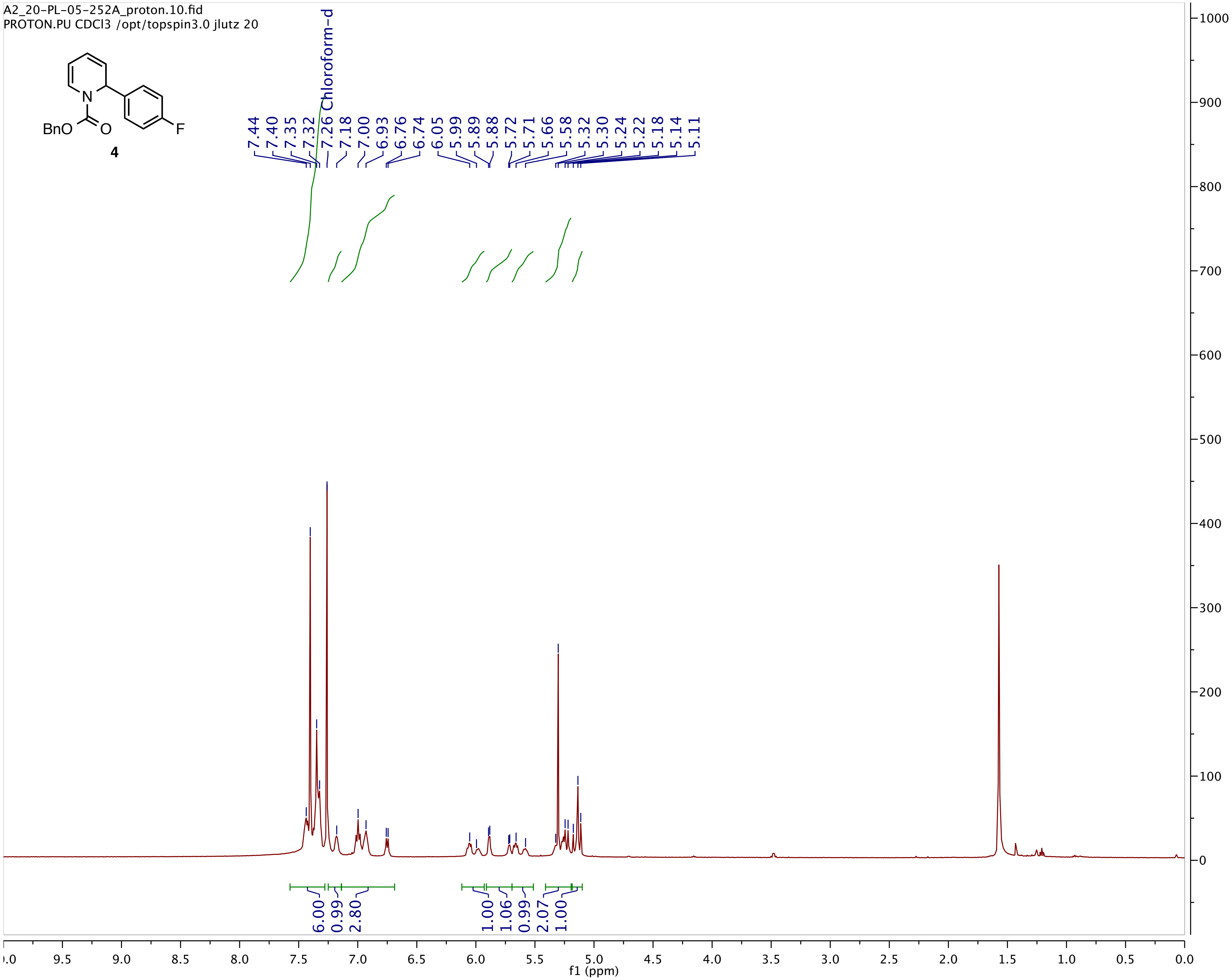

500 MHz ¹H-NMR spectrum of **4** in $CDCl_3$

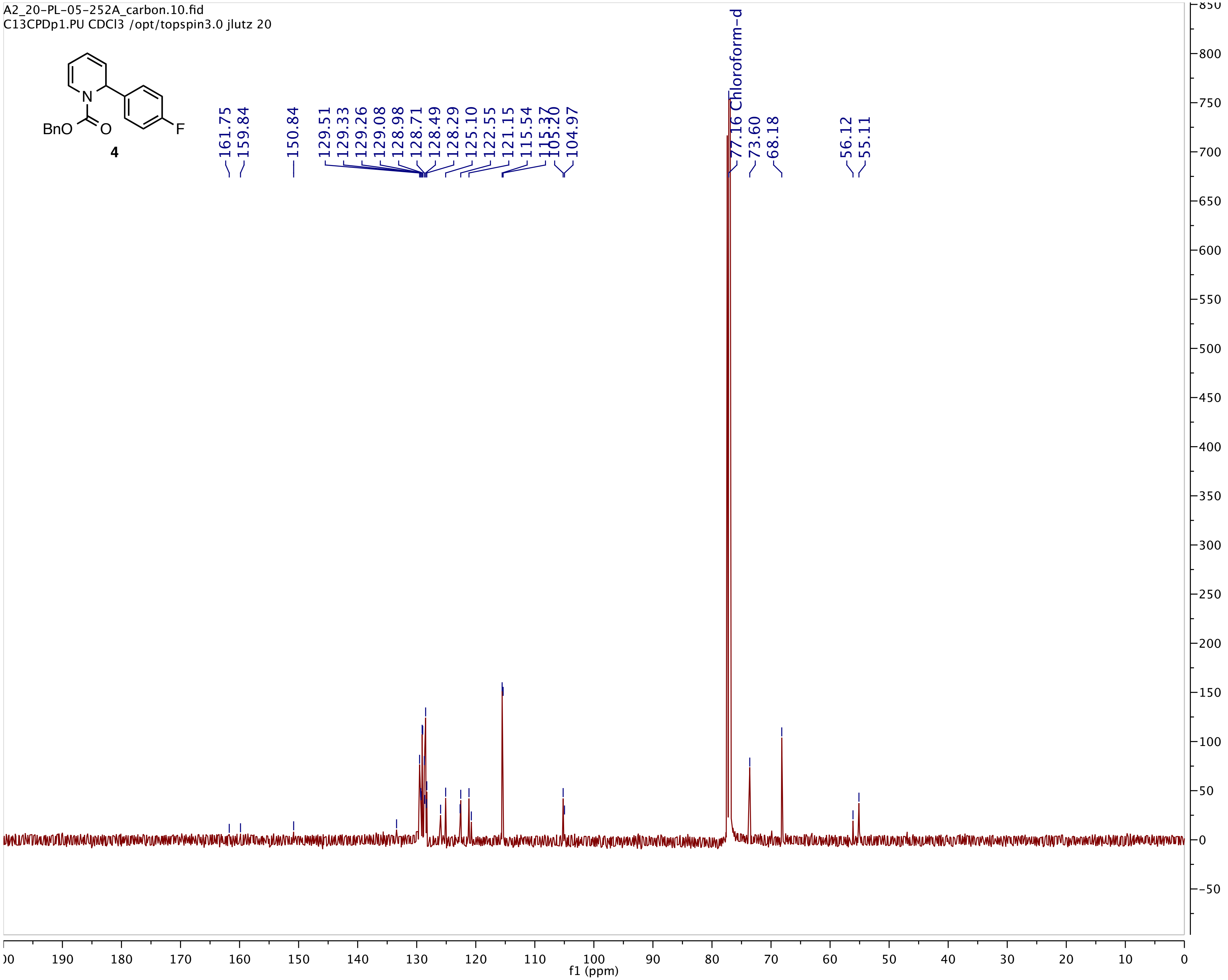

125 MHz ^{13}C-NMR spectrum of **4** in $CDCl_3$

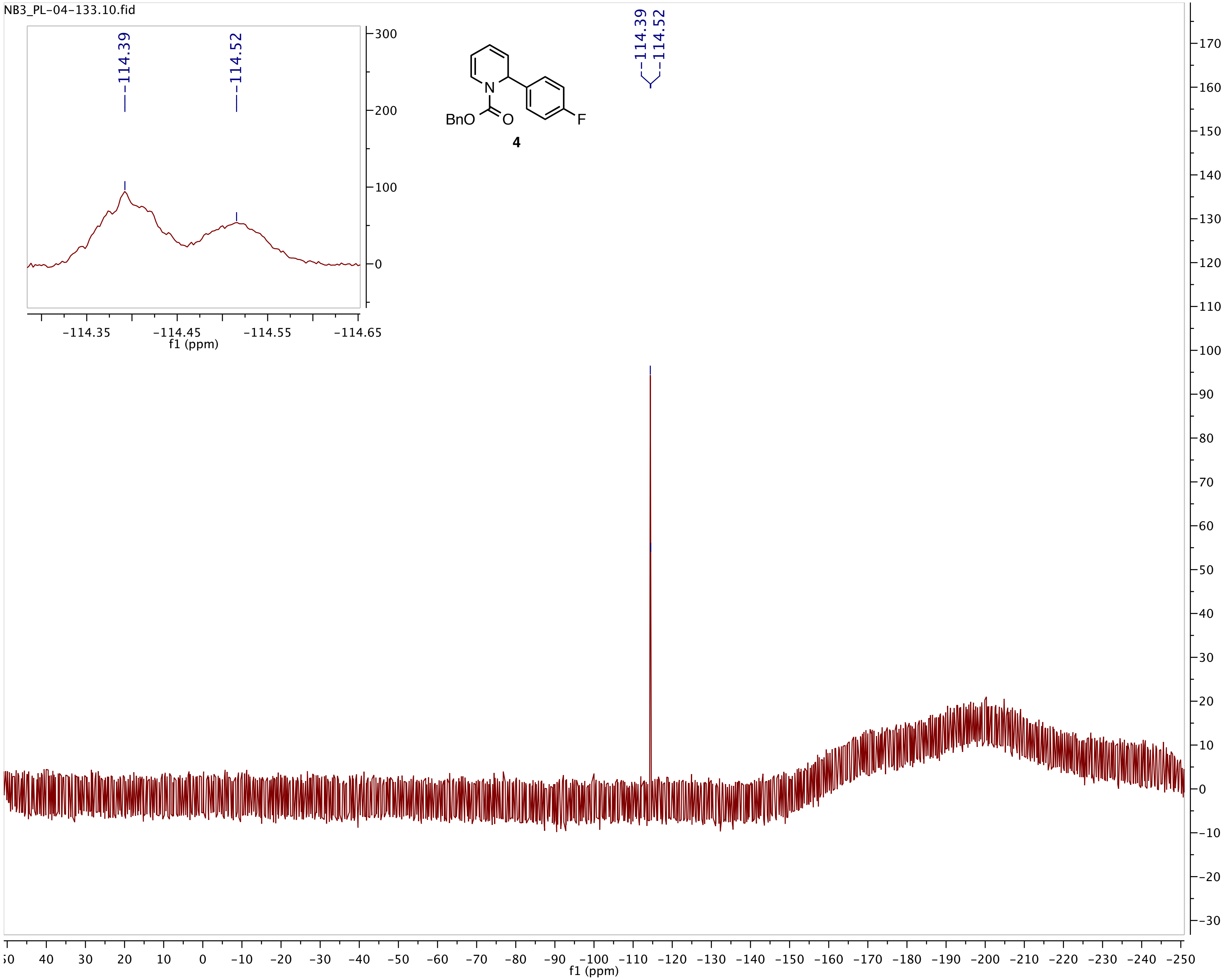

282 MHz ^{19}F-NMR spectrum of **4** in $CDCl_3$

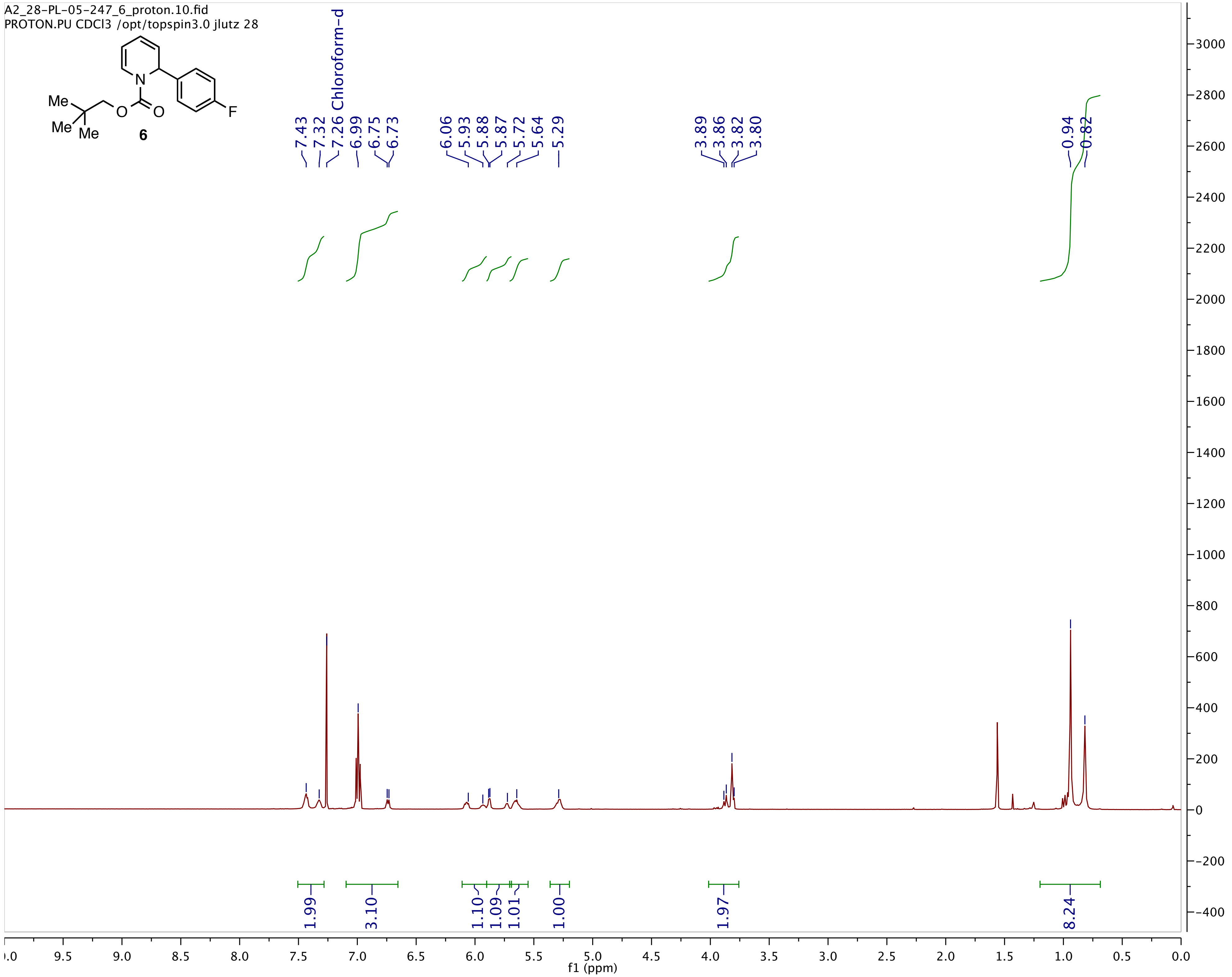

500 MHz ^{1}H-NMR spectrum of **6** in $CDCl_3$

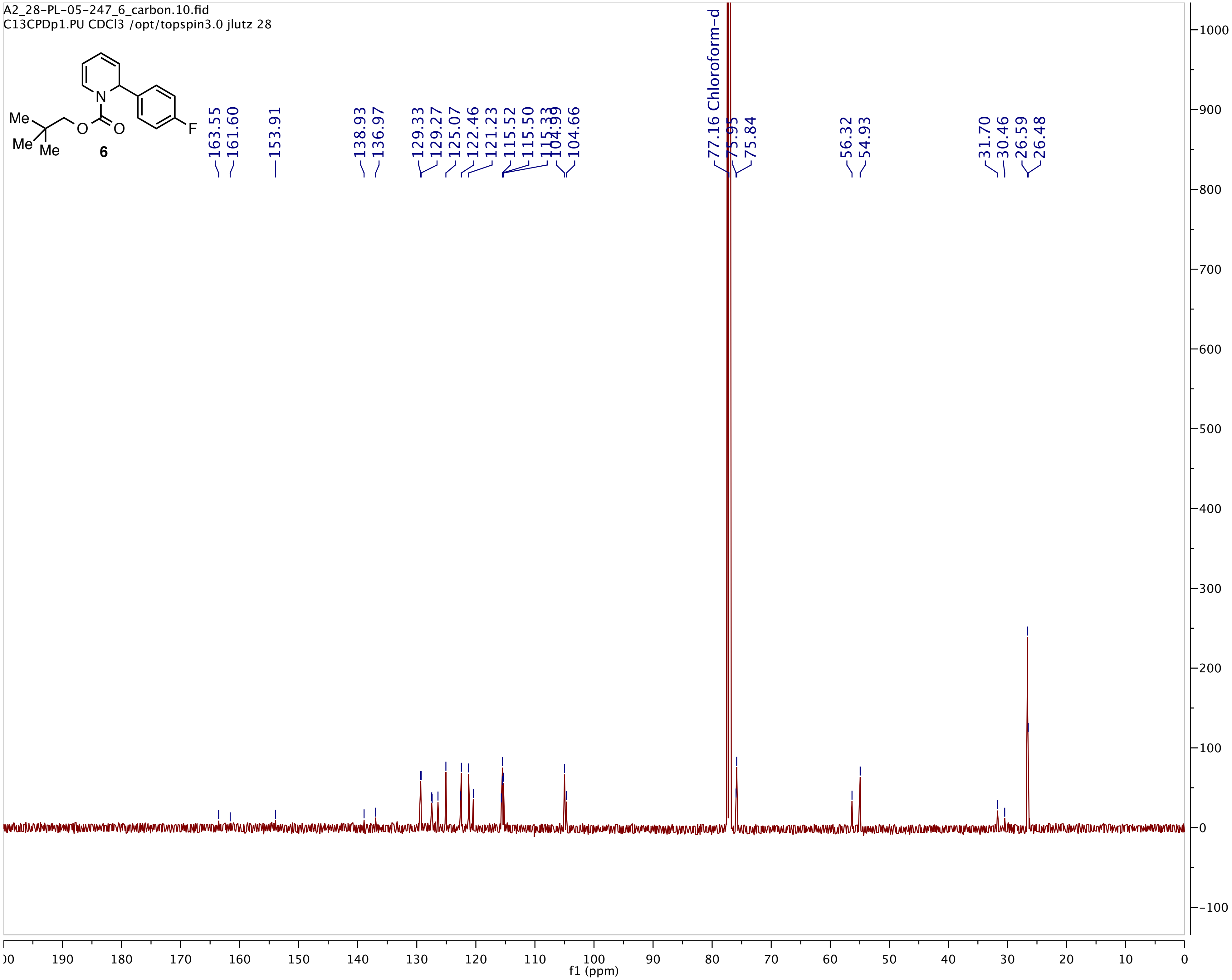

125 MHz ^{13}C-NMR spectrum of **6** in $CDCl_3$

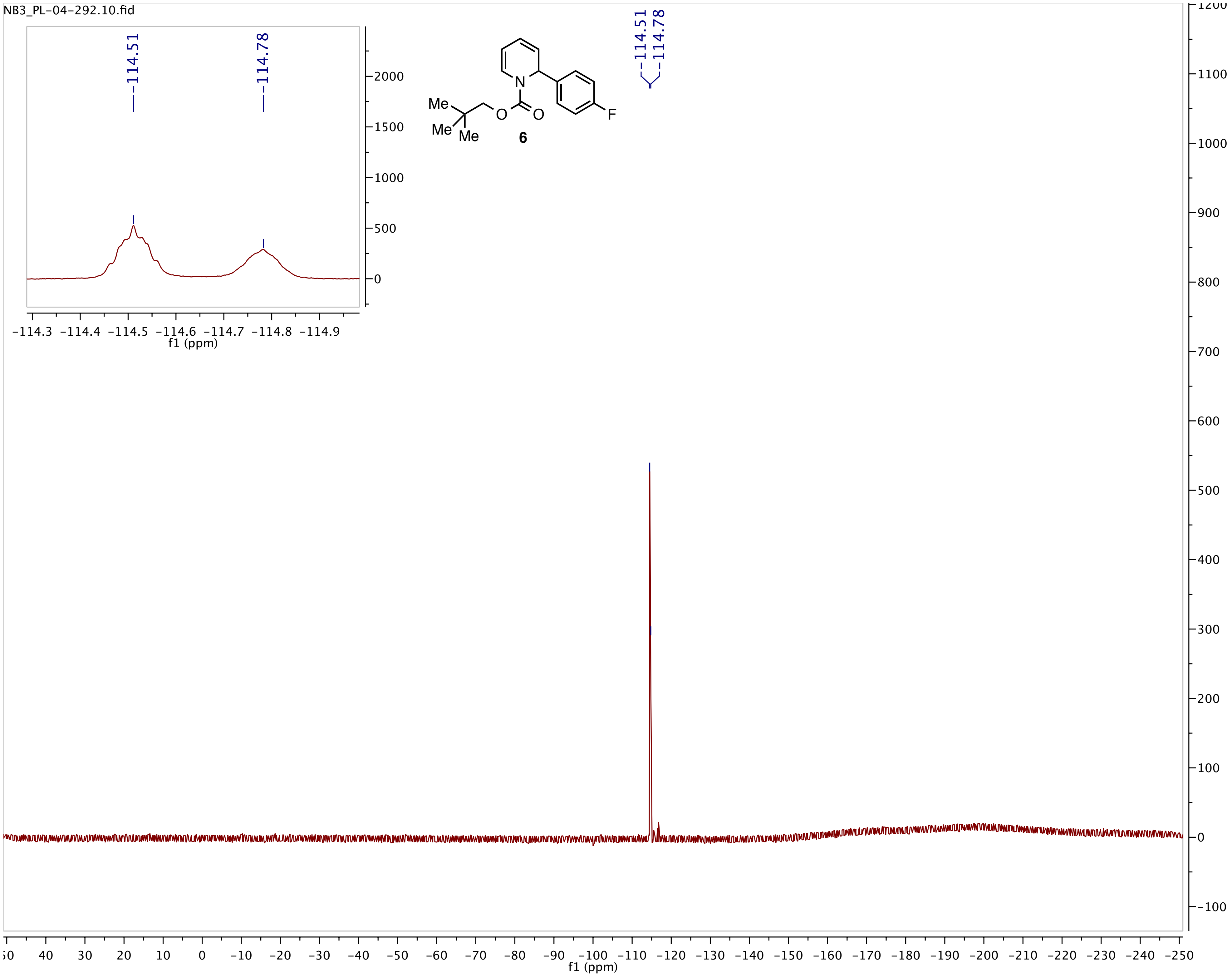

282 MHz ^{19}F-NMR spectrum of **6** in $CDCl_3$

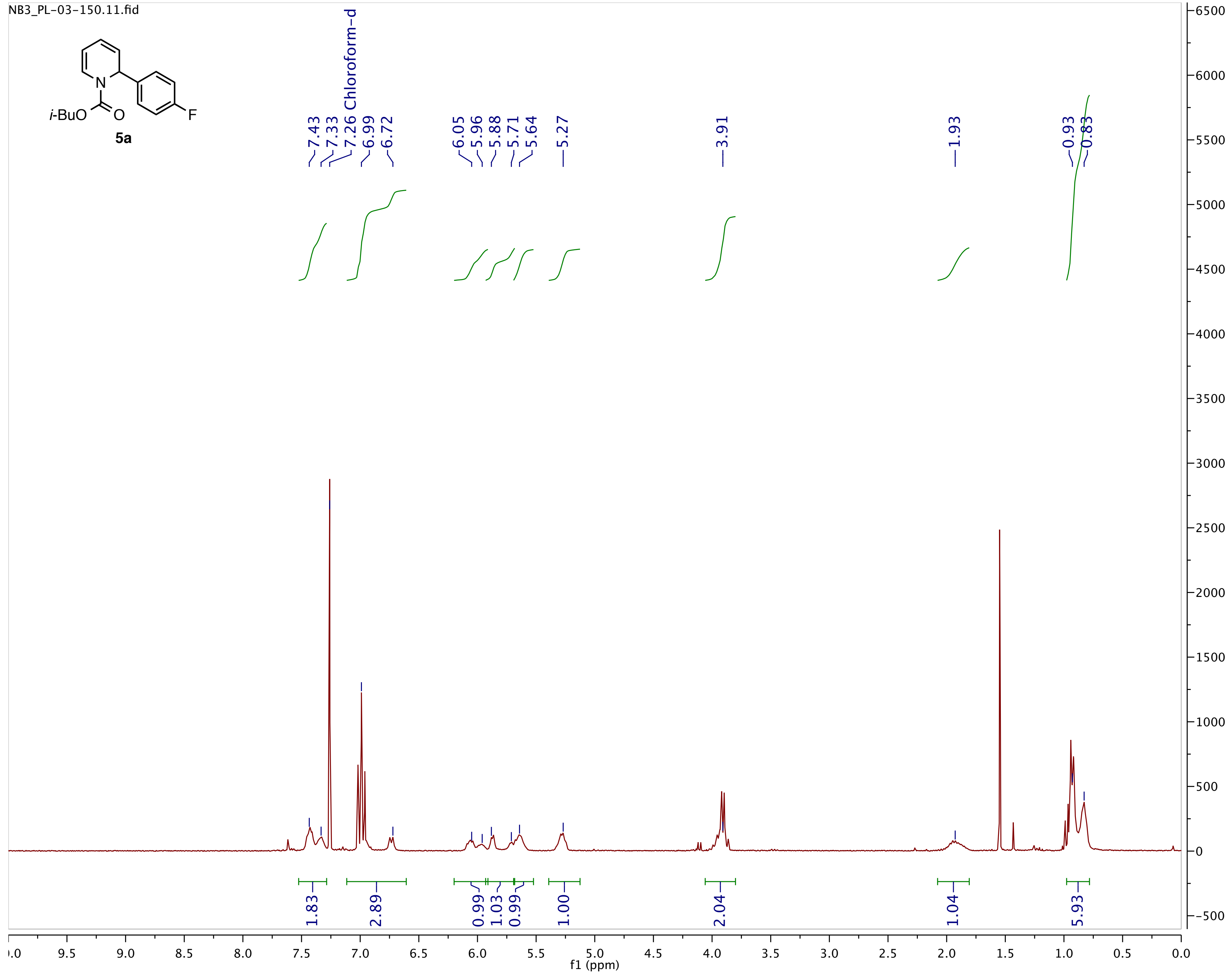

300 MHz ^{19}F-NMR spectrum of **5a** in $CDCl_3$

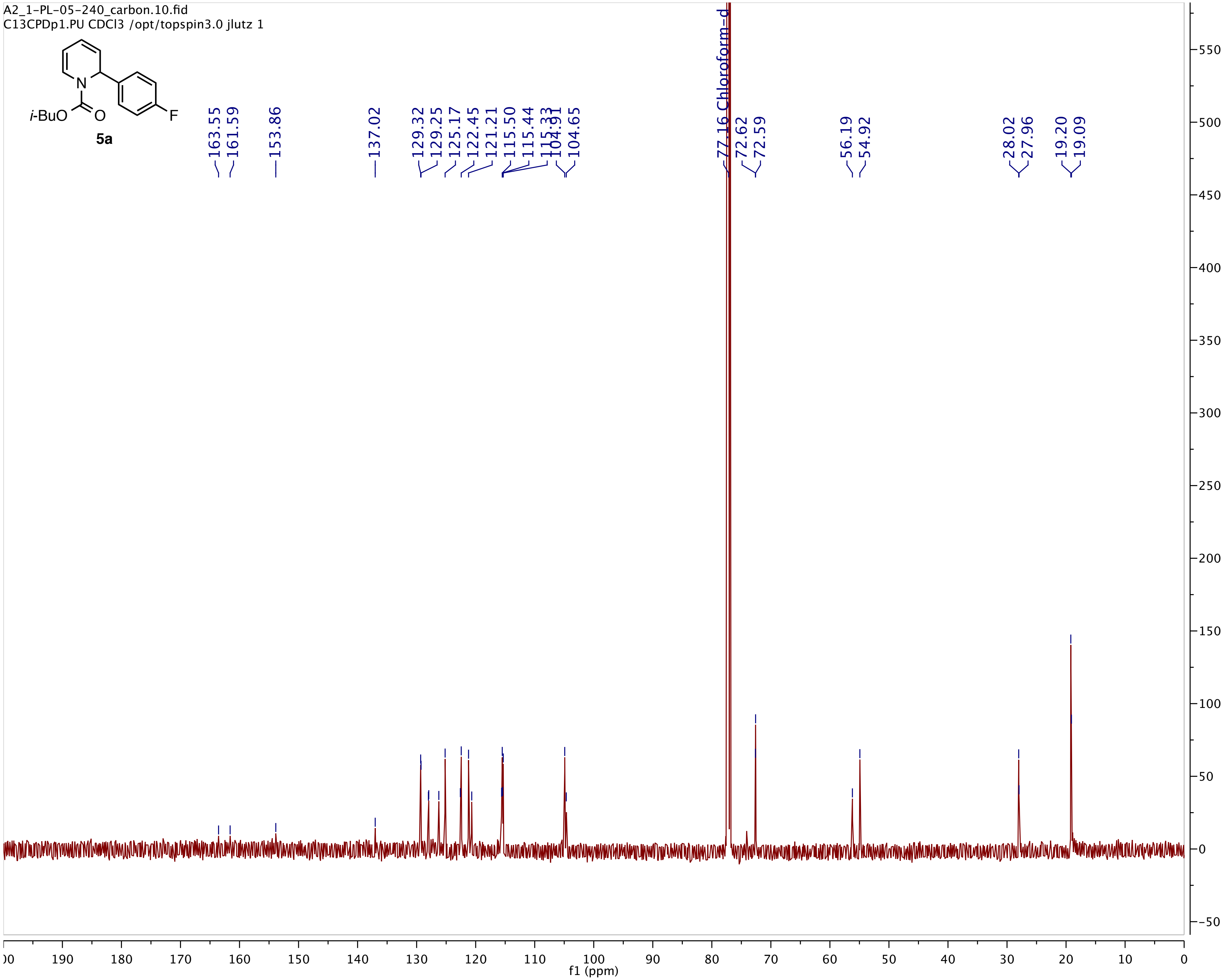

125 MHz ^{13}C-NMR spectrum of **5a** in $CDCl_3$

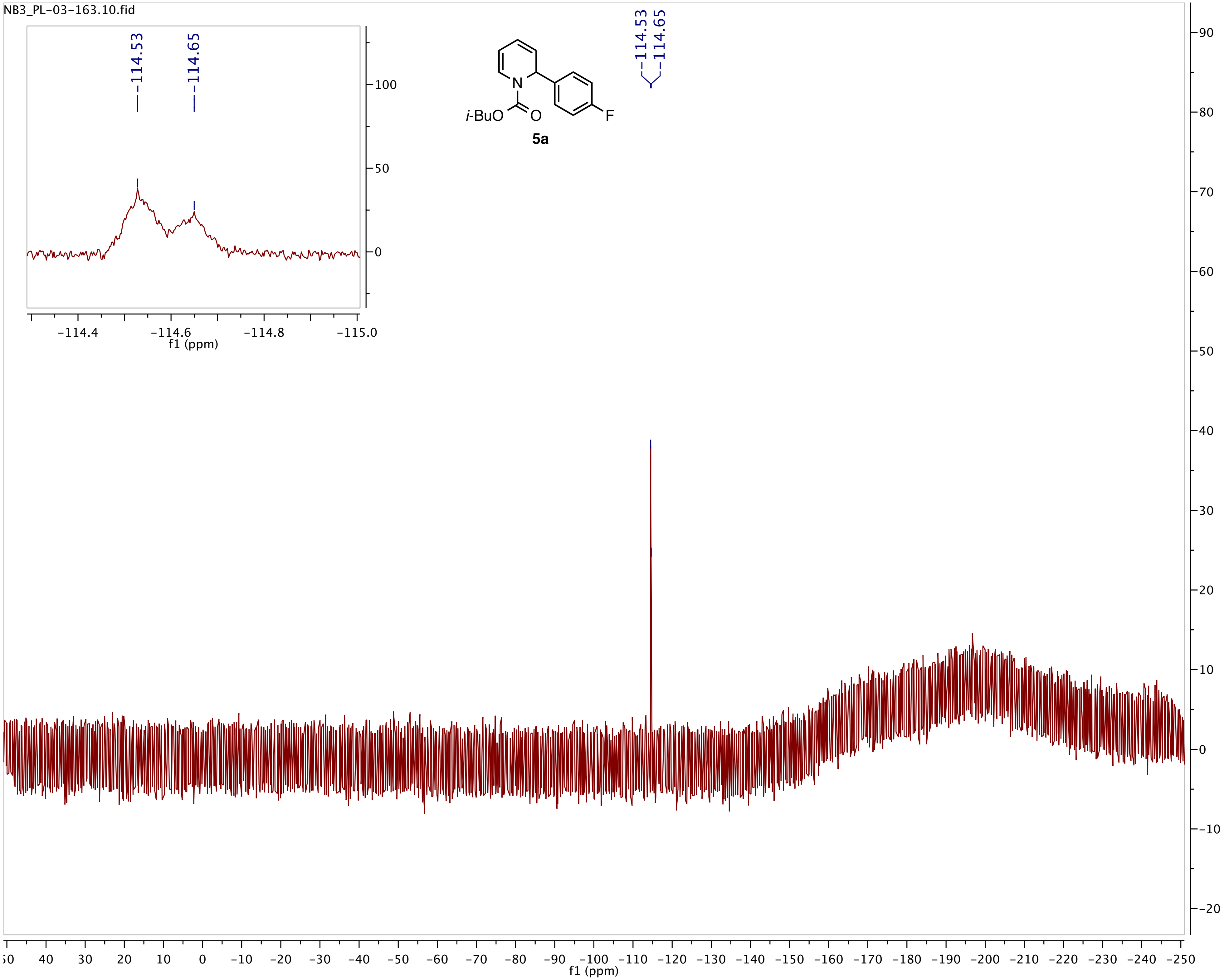

282 MHz ^{19}F-NMR spectrum of **5a** in $CDCl_3$

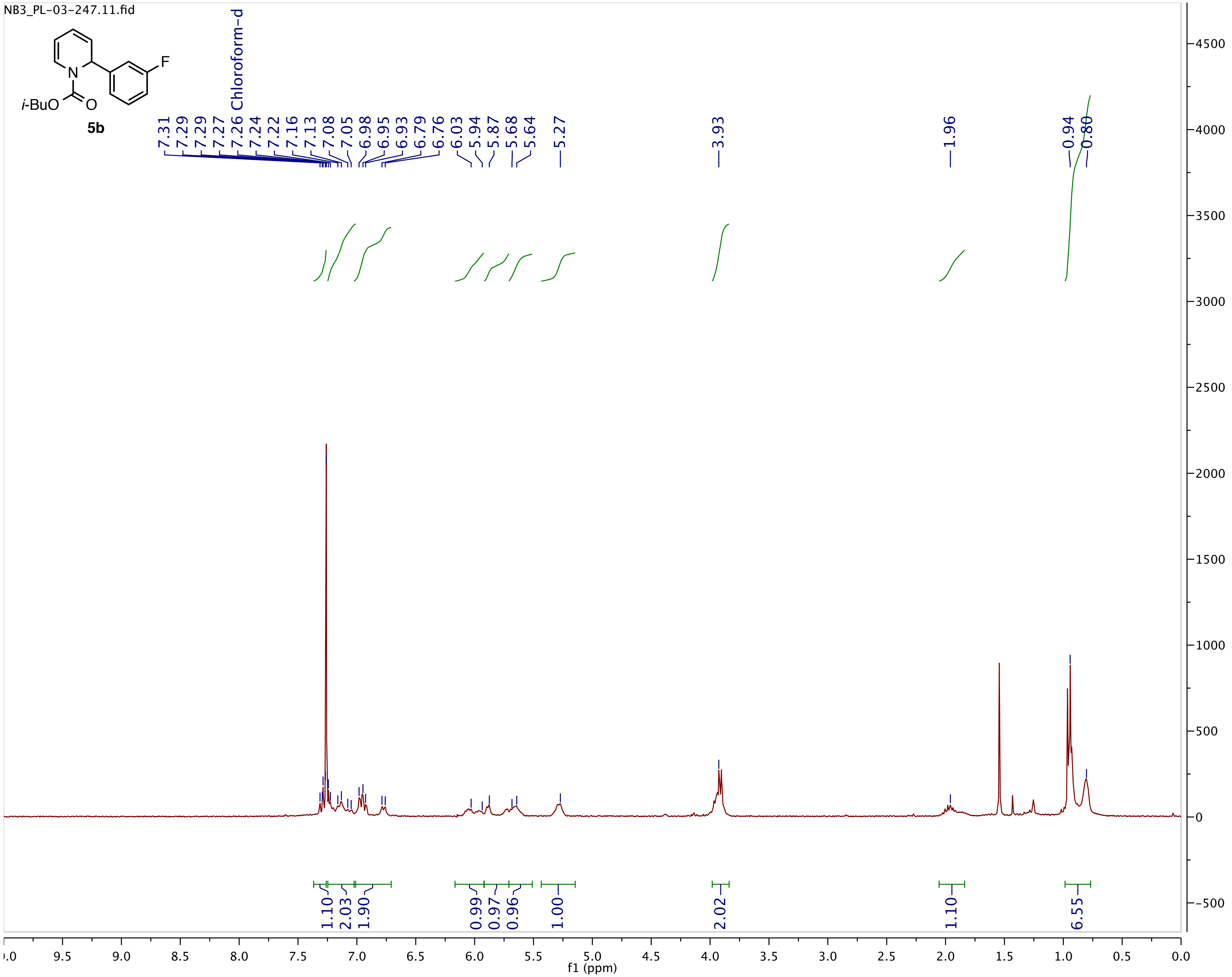

300 MHz ¹H-NMR spectrum of **5b** in $CDCl_3$

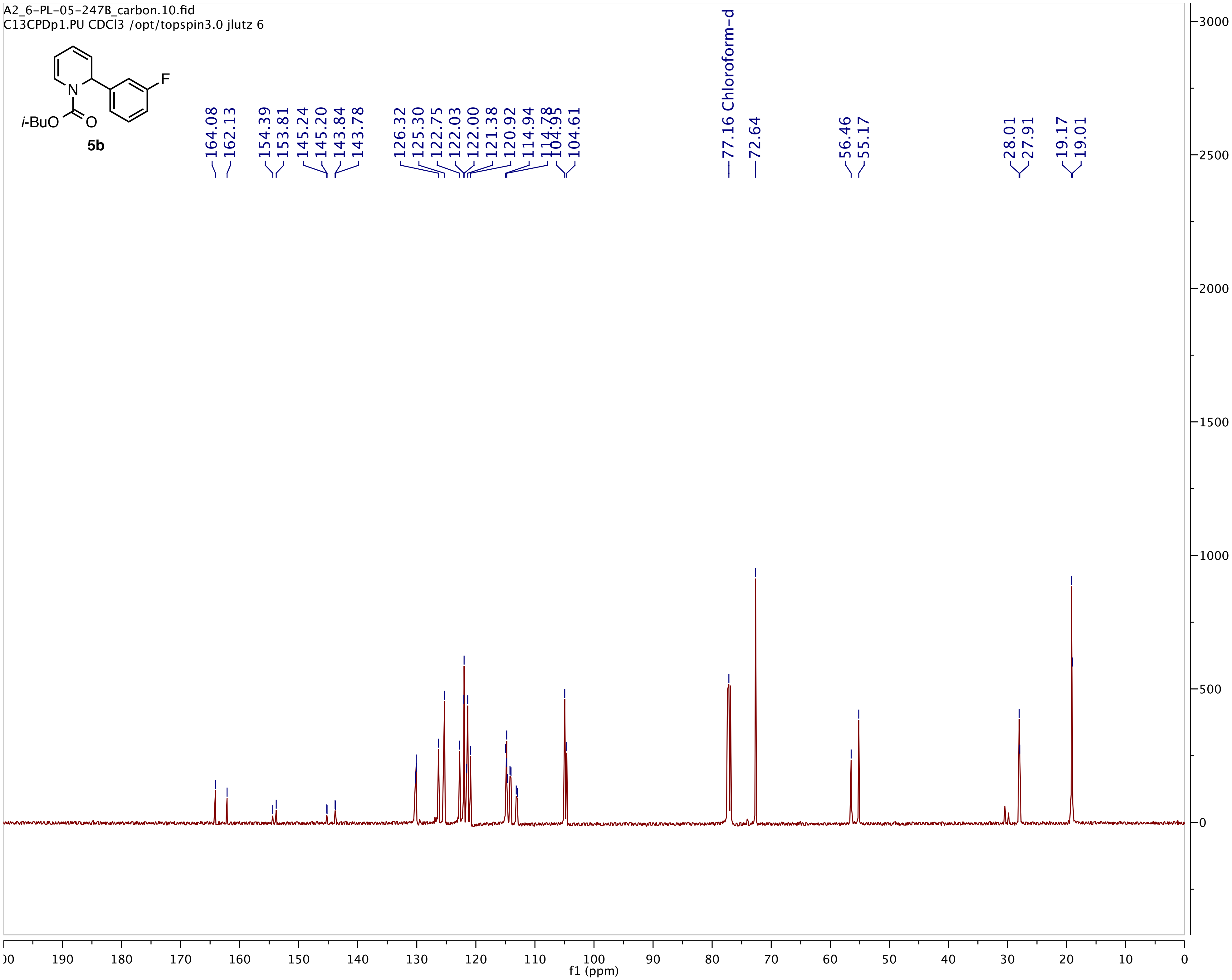

125 MHz ^{13}C-NMR spectrum of **5b** in $CDCl_3$

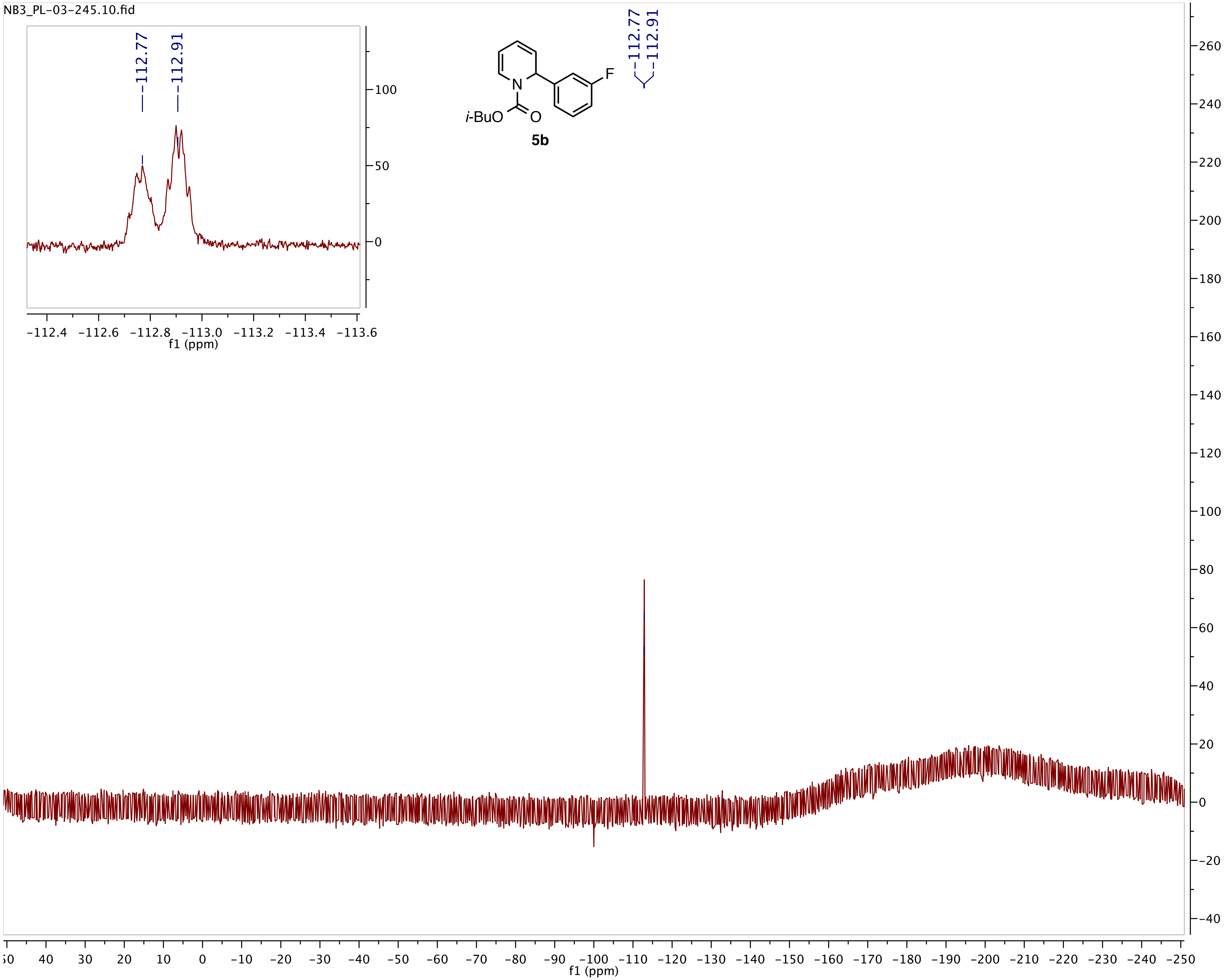

282 MHz ^{19}F-NMR spectrum of **5b** in $CDCl_3$

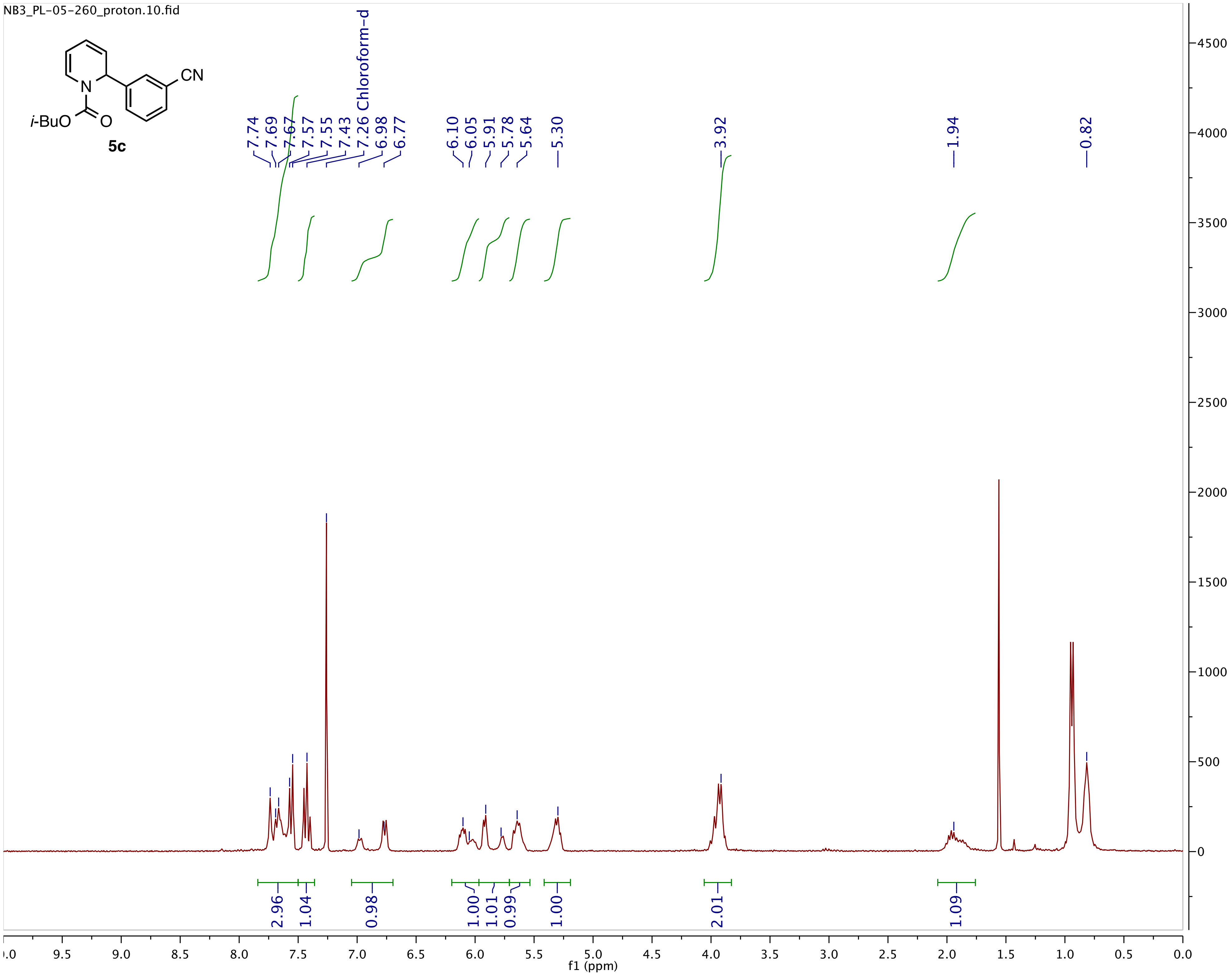

300 MHz ^{1}H-NMR spectrum of **5c** in $CDCl_3$

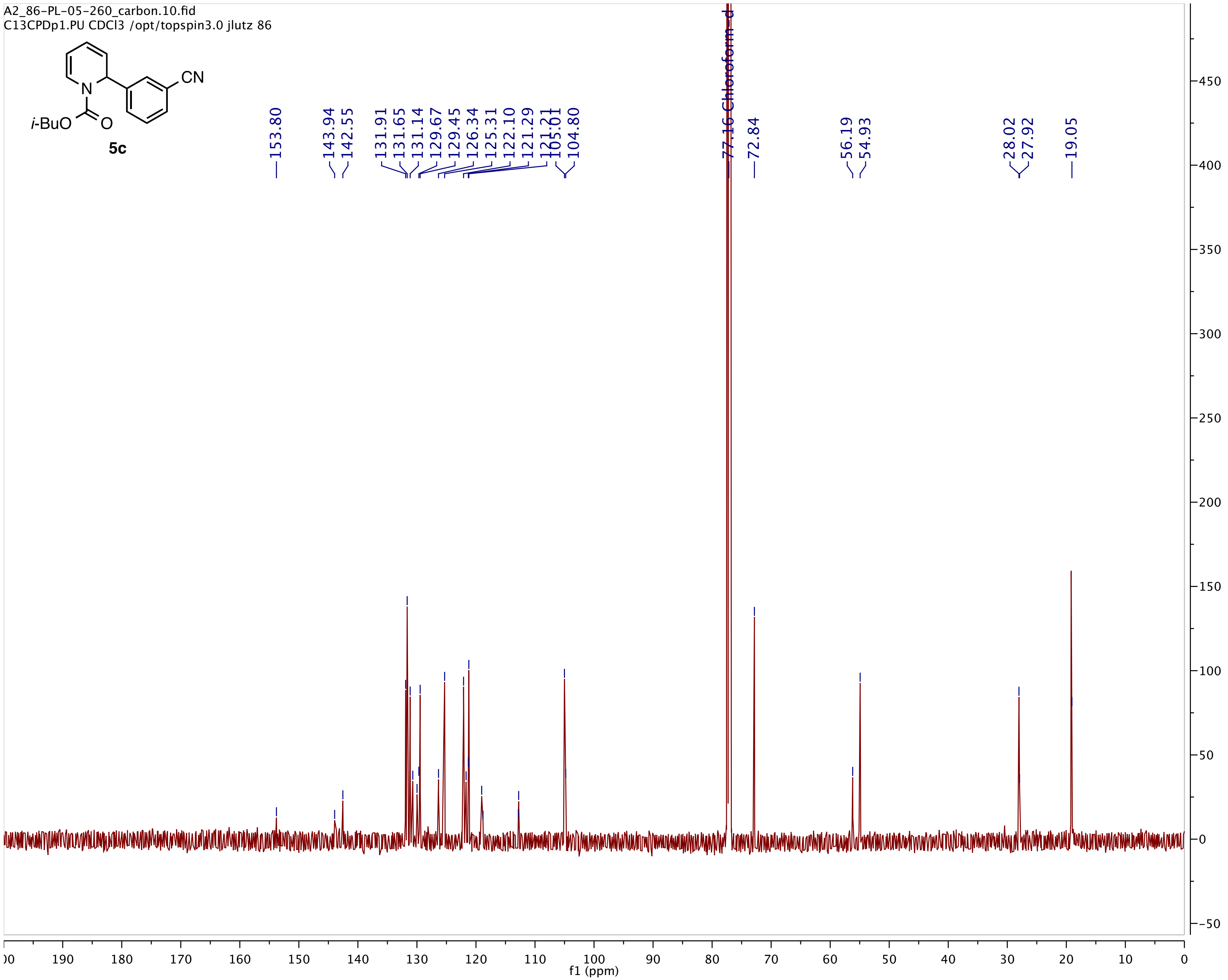

125 MHz ^{13}C-NMR spectrum of **5c** in $CDCl_3$

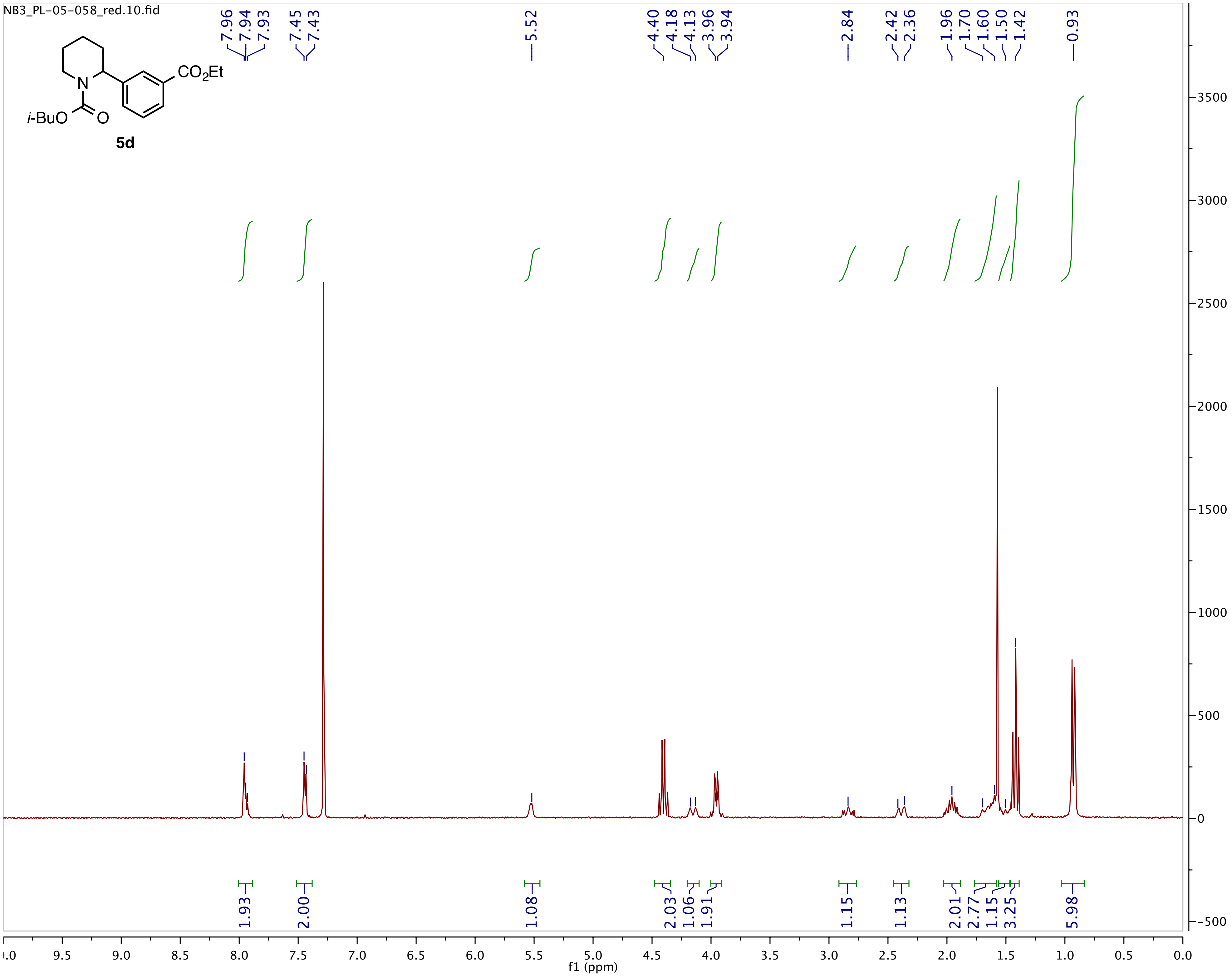

300 MHz ¹H-NMR spectrum of **5d** in $CDCl_3$

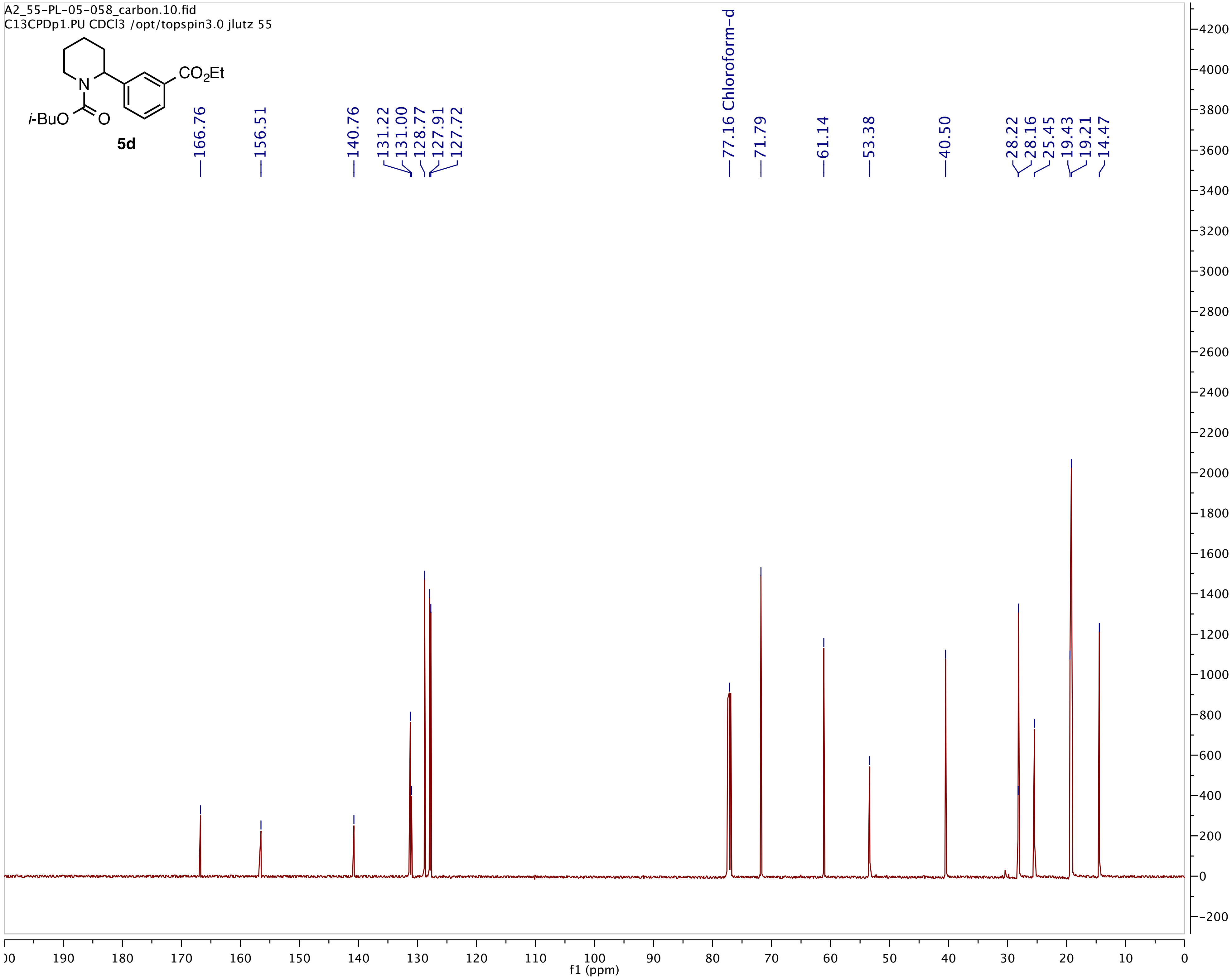

125 MHz ^{13}C-NMR spectrum of **5d** in $CDCl_3$

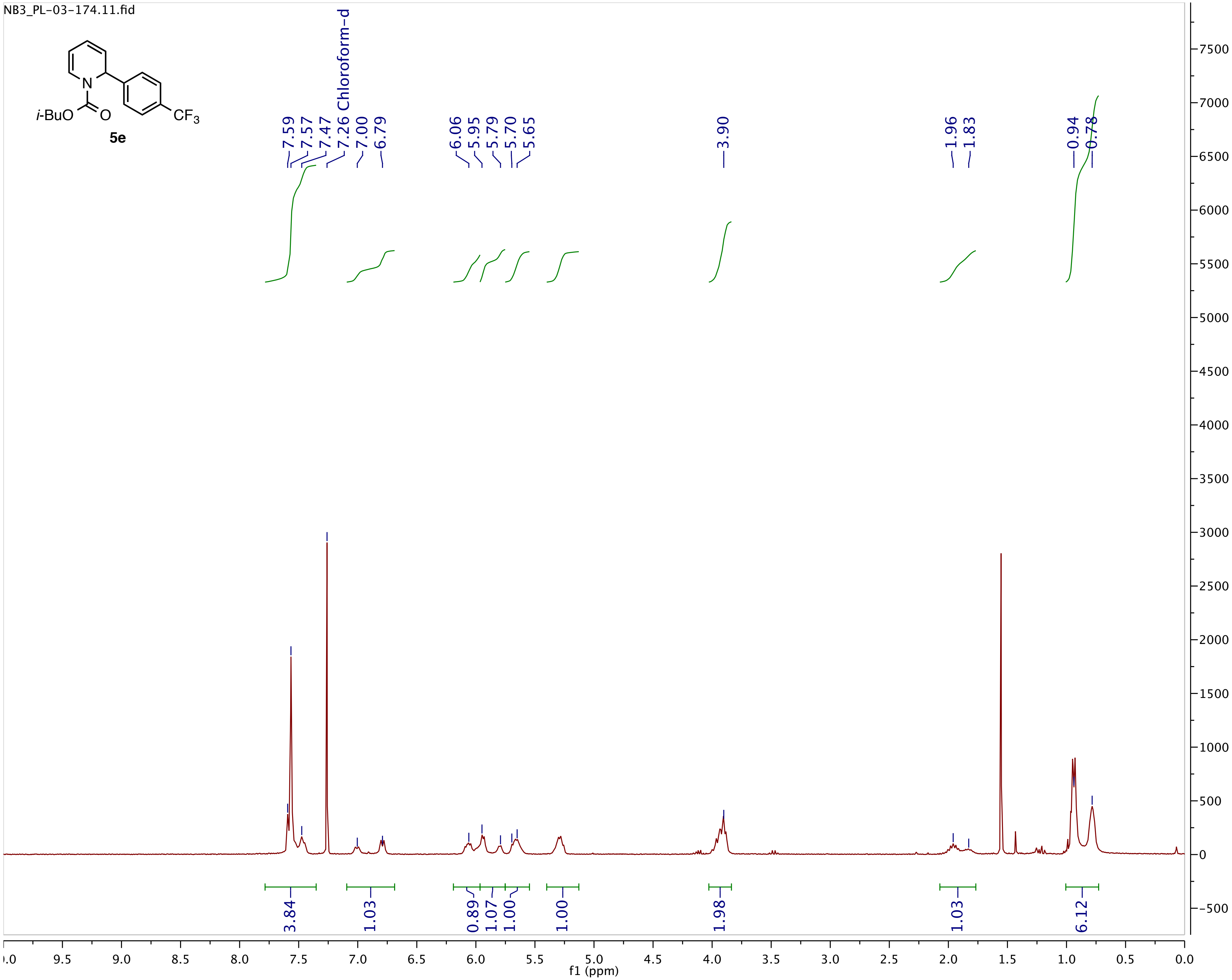

300 MHz ^{1}H-NMR spectrum of **5e** in $CDCl_3$

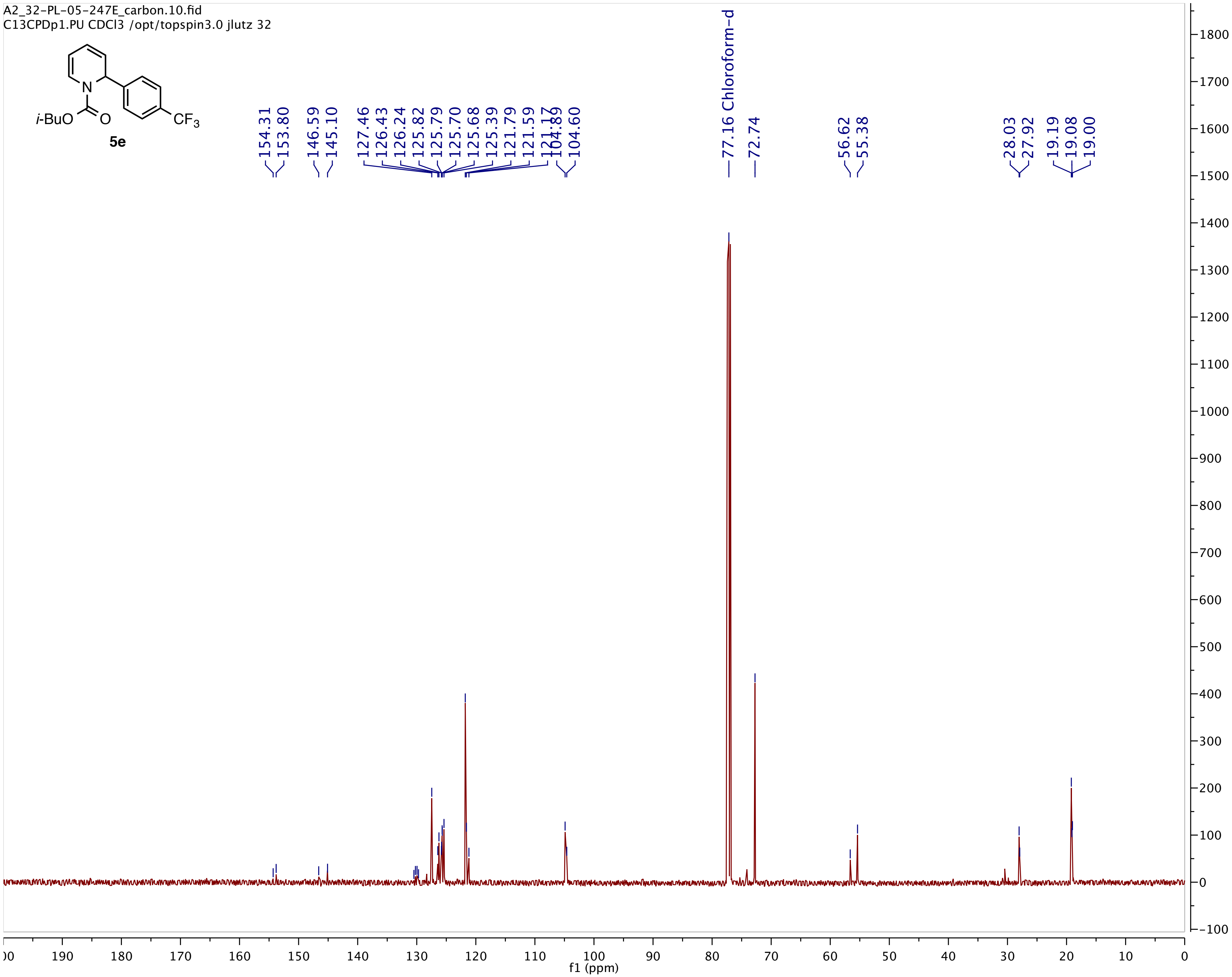

125 MHz ^{13}C-NMR spectrum of **5e** in $CDCl_3$

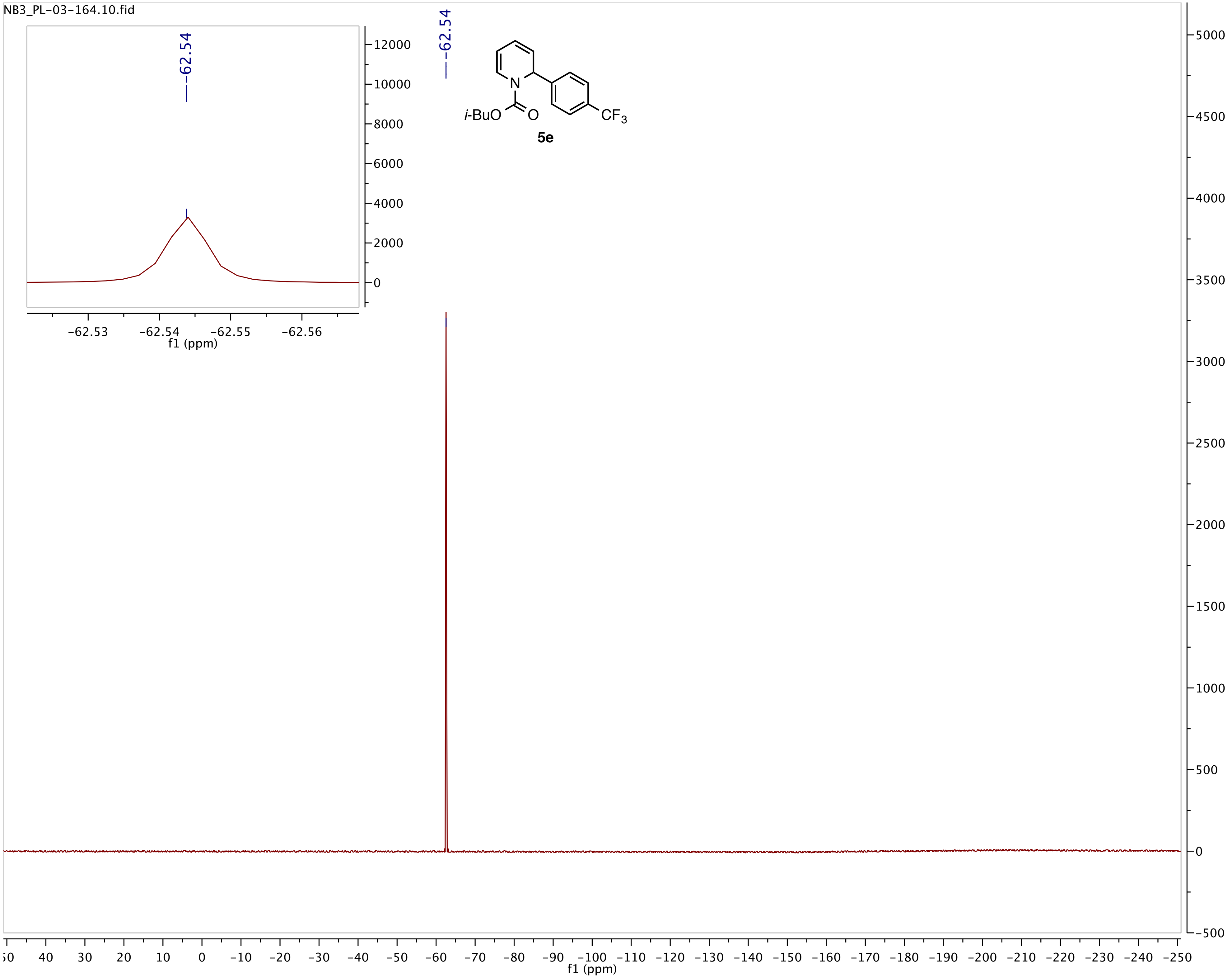

282 MHz ^{19}F-NMR spectrum of **5e** in $CDCl_3$

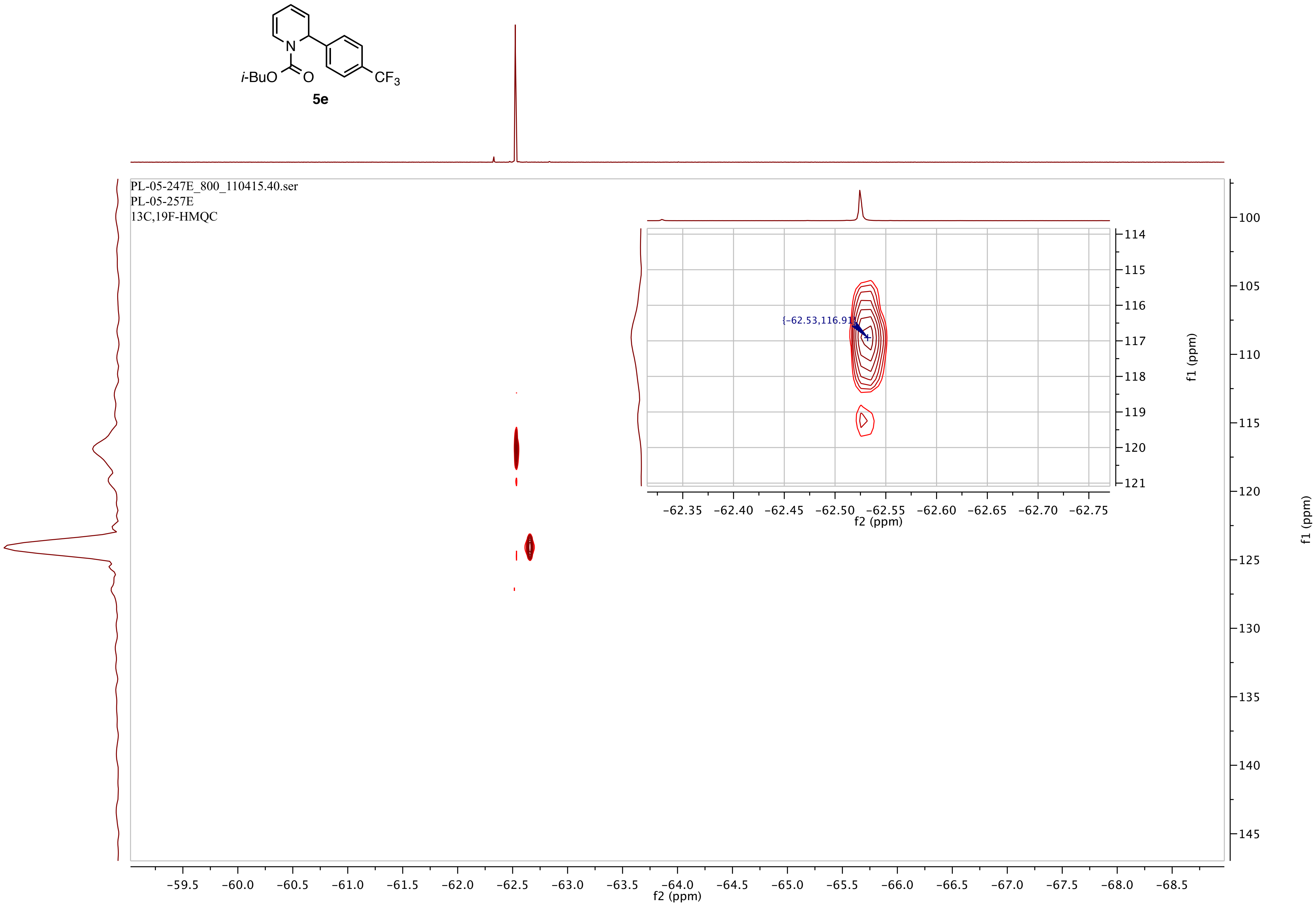

^{13}C,^{19}F-HMQC spectrum of **5e** in $CDCl_3$

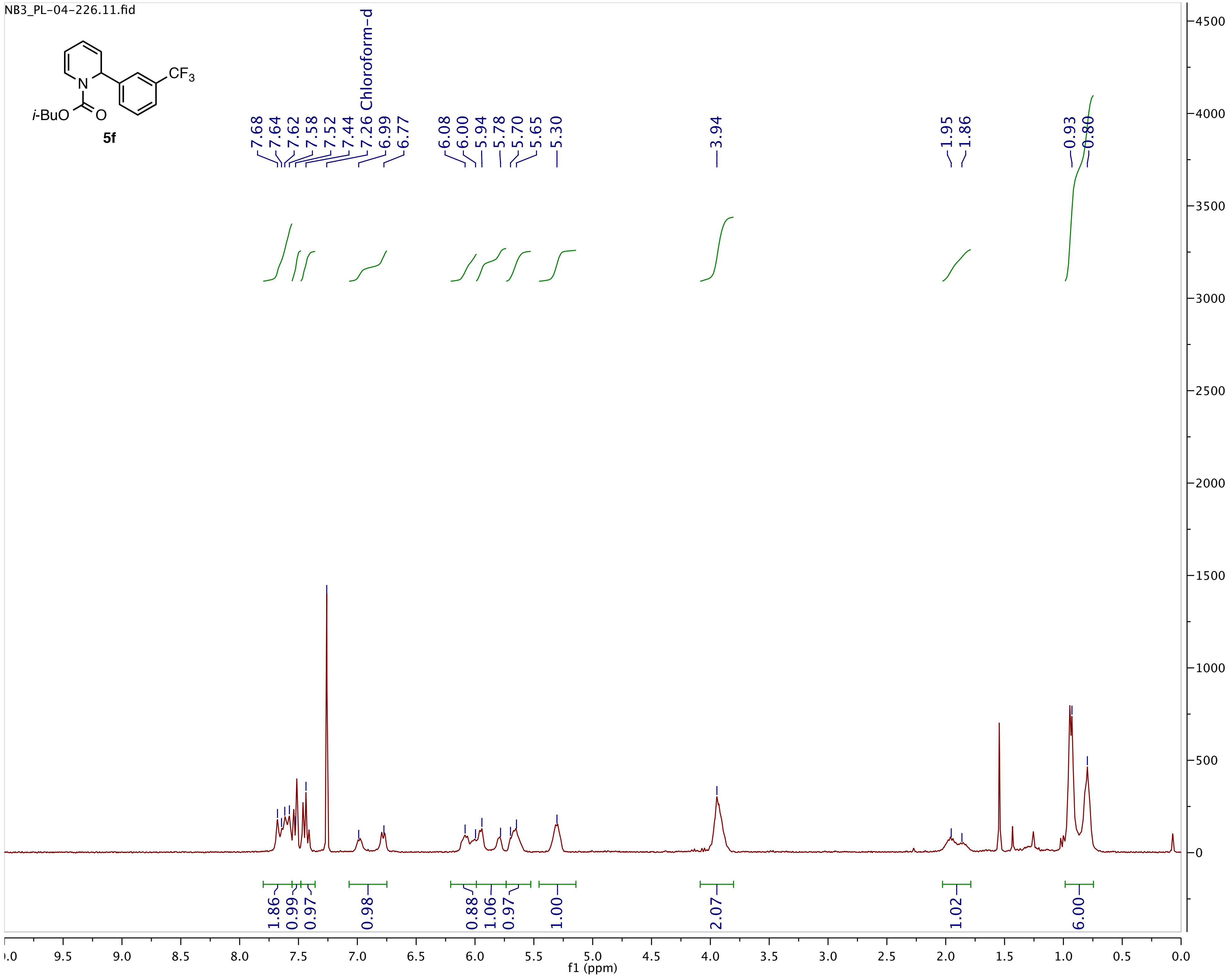

300 MHz ^{1}H-NMR spectrum of **5f** in $CDCl_3$

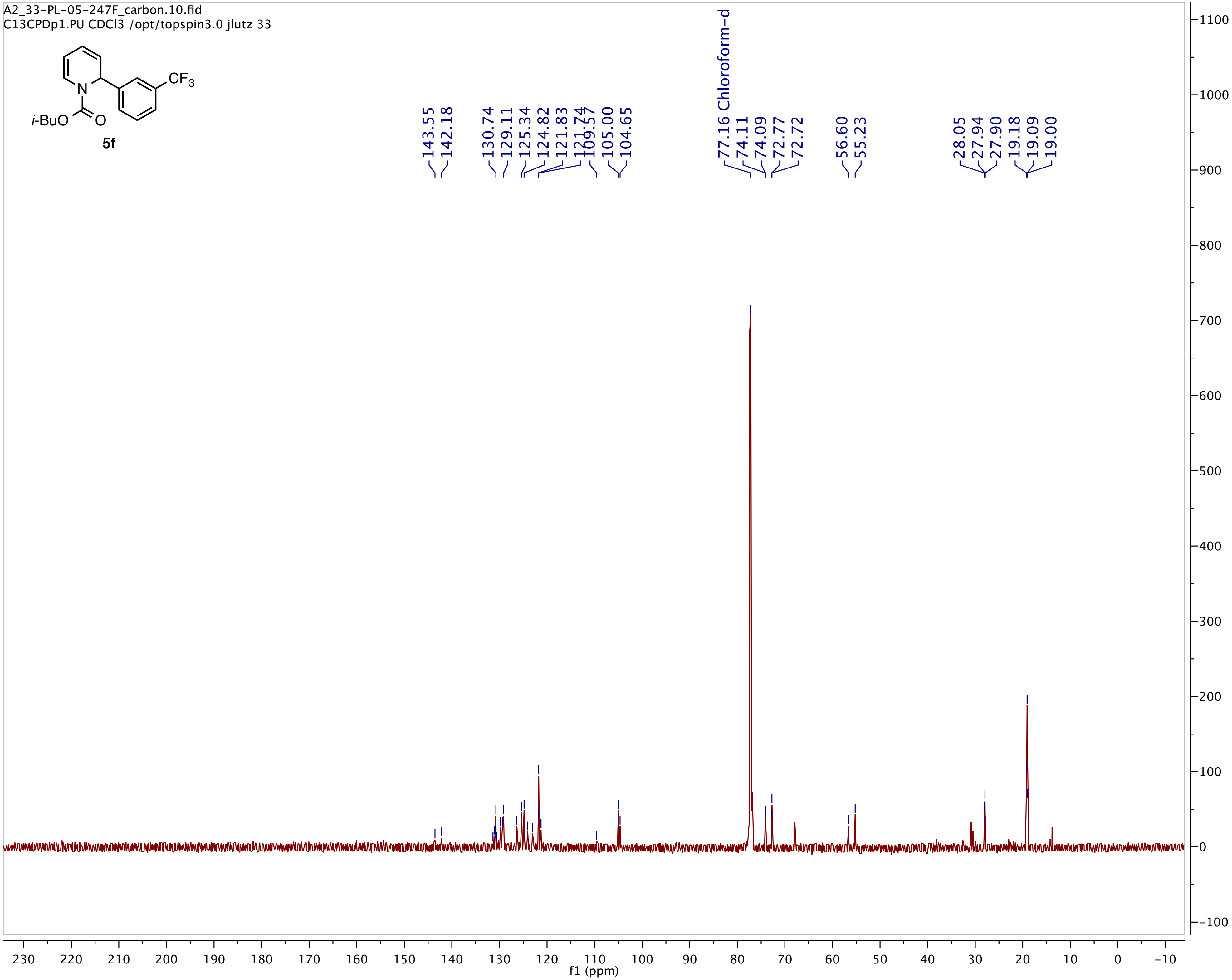

125 MHz ^{13}C-NMR spectrum of **5f** in $CDCl_3$

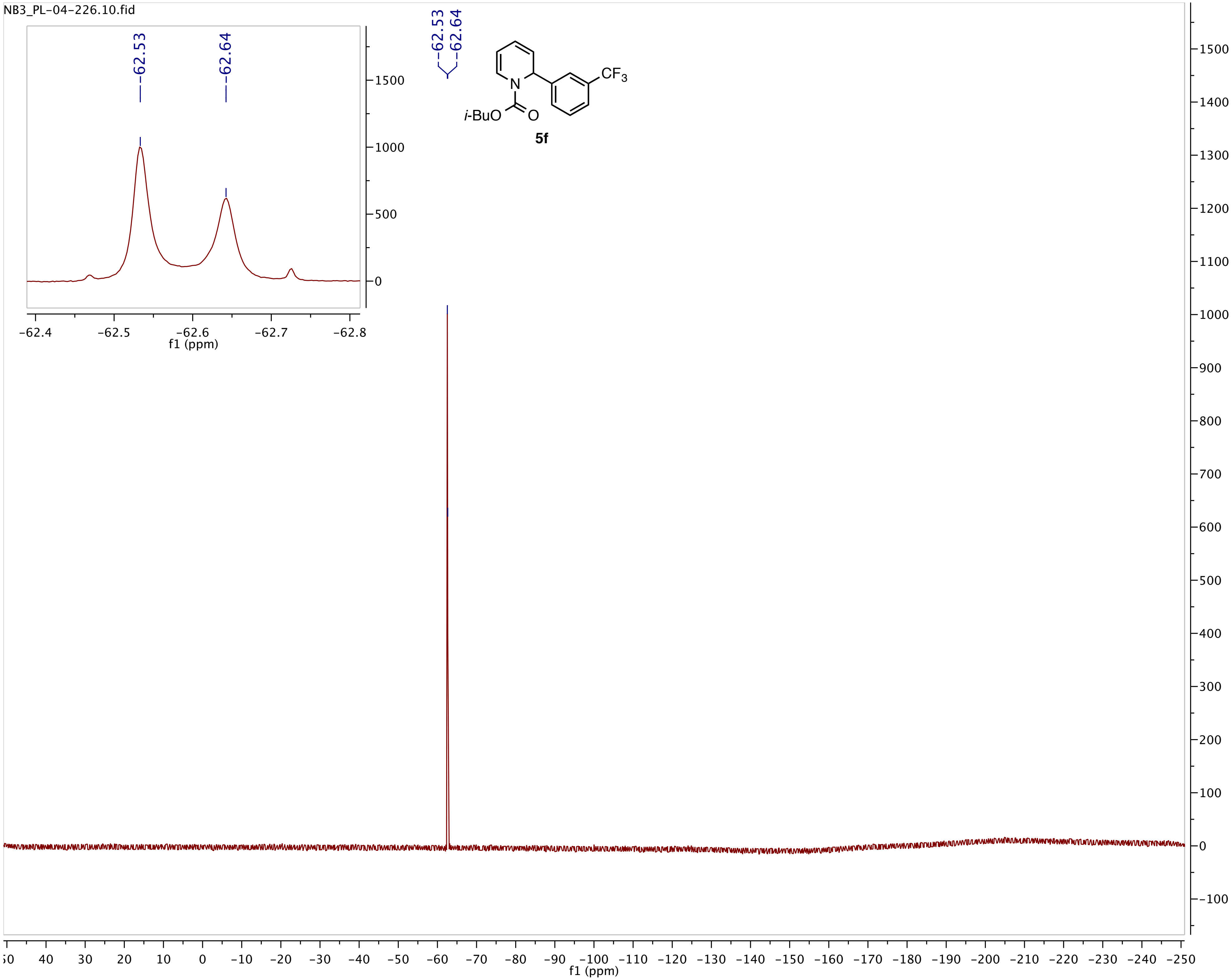

282 MHz ^{19}F-NMR spectrum of **5f** in $CDCl_3$

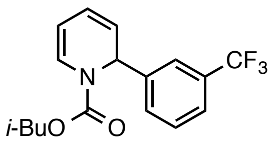

5f

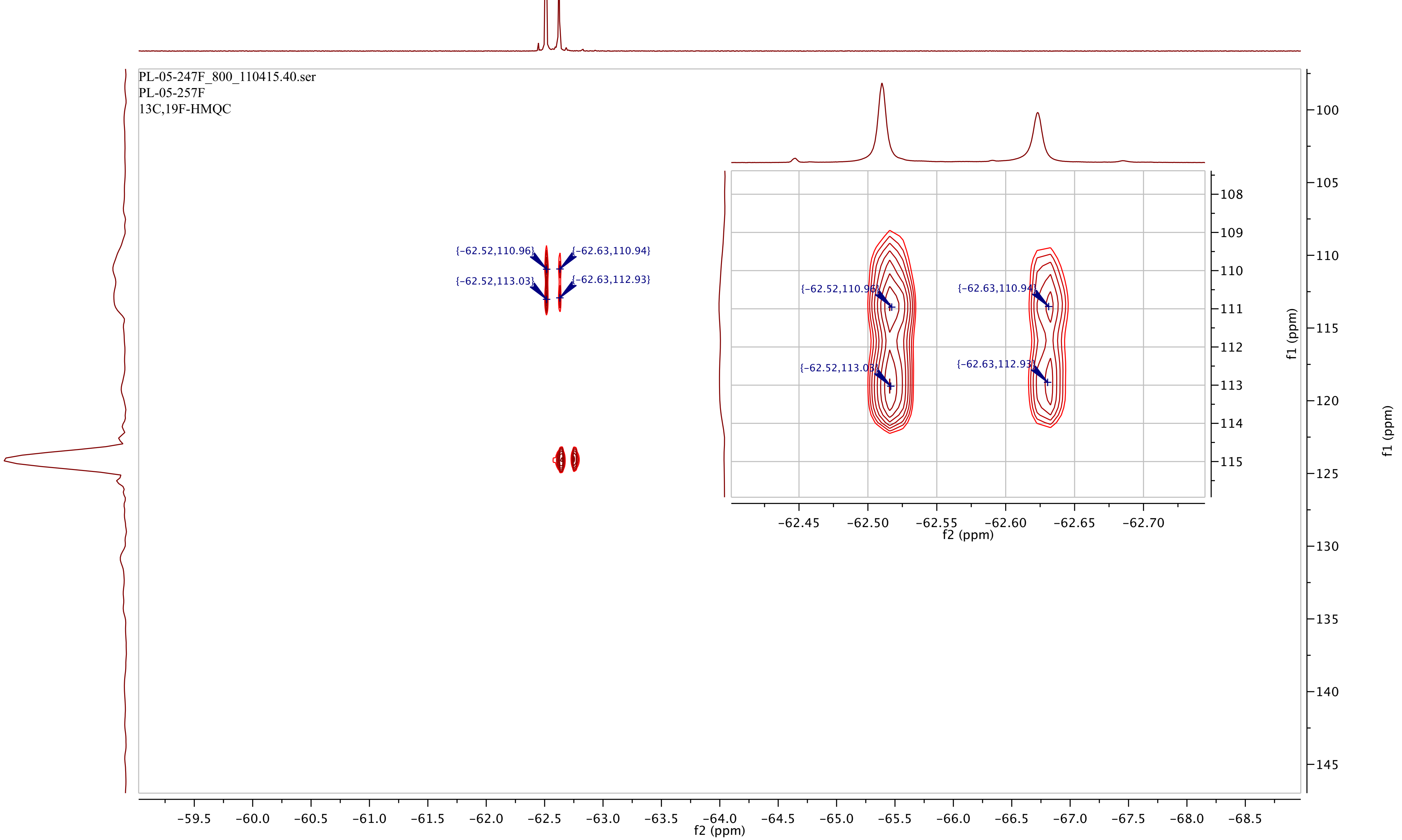

^{13}C,^{19}F-HMQC spectrum of **5f** in $CDCl_3$

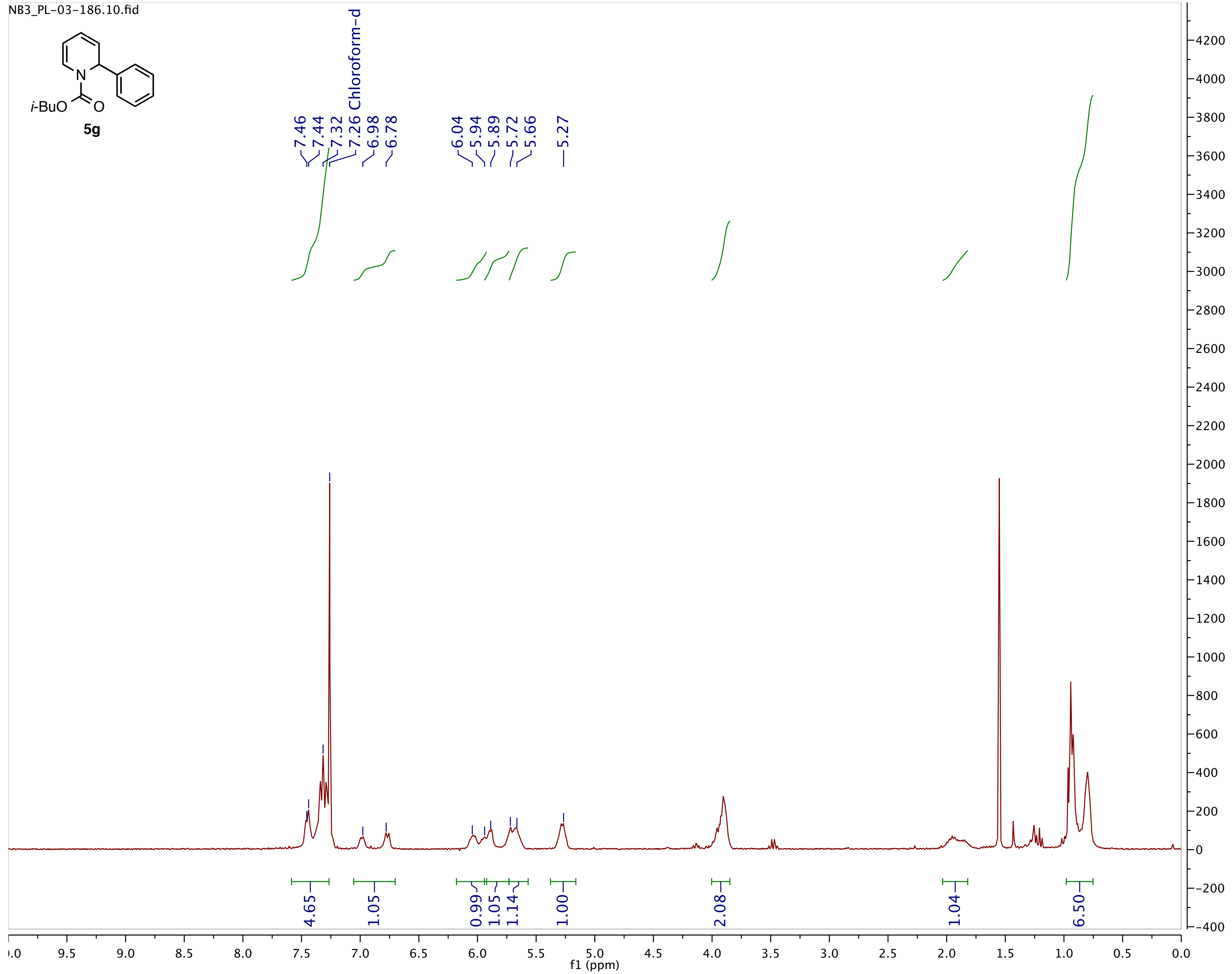

300 MHz ^{1}H-NMR spectrum of **5g** in $CDCl_3$

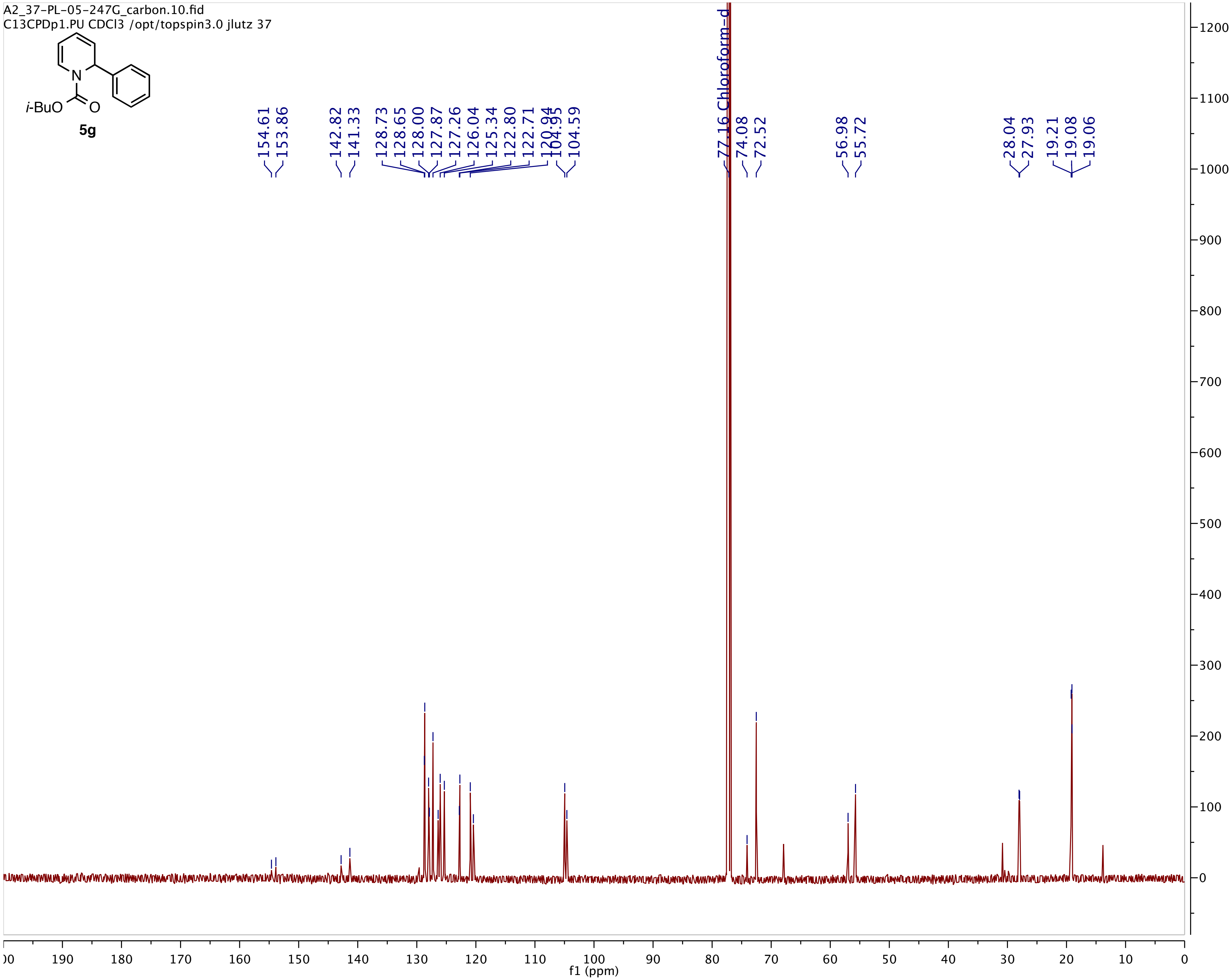

125 MHz ^{13}C-NMR spectrum of **5g** in $CDCl_3$

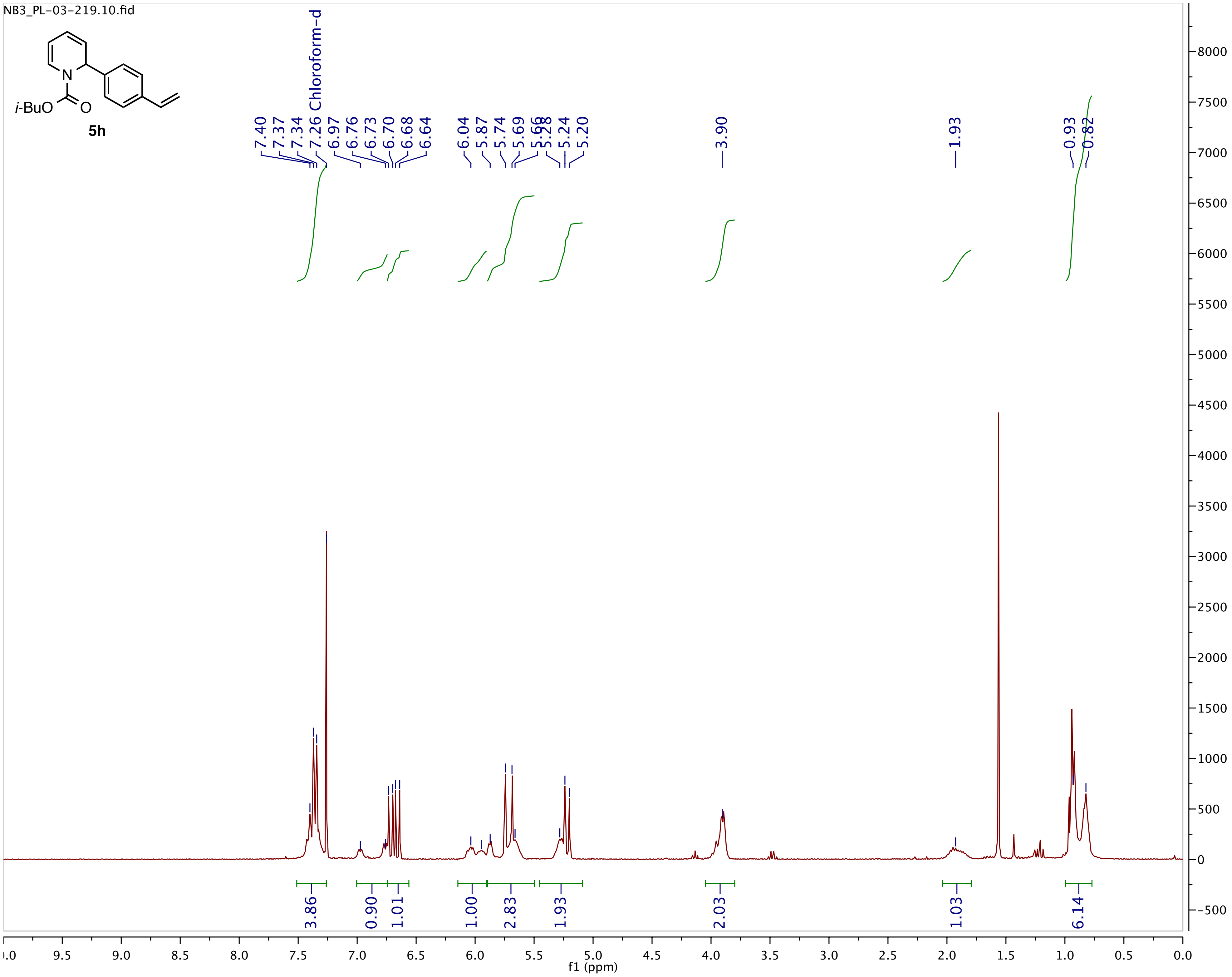

300 MHz ^{1}H-NMR spectrum of **5h** in $CDCl_3$

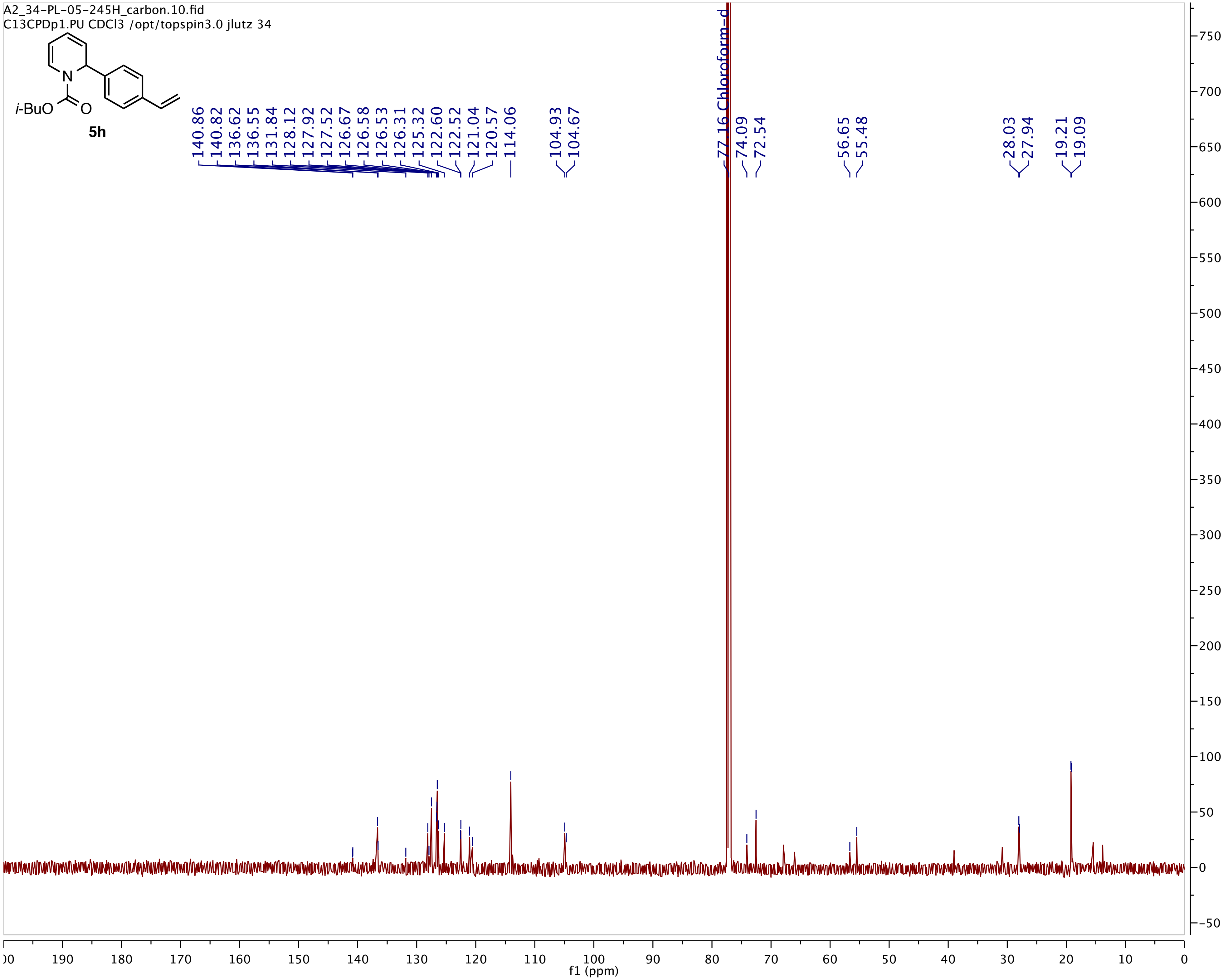

125 MHz ^{13}C-NMR spectrum of **5h** in $CDCl_3$

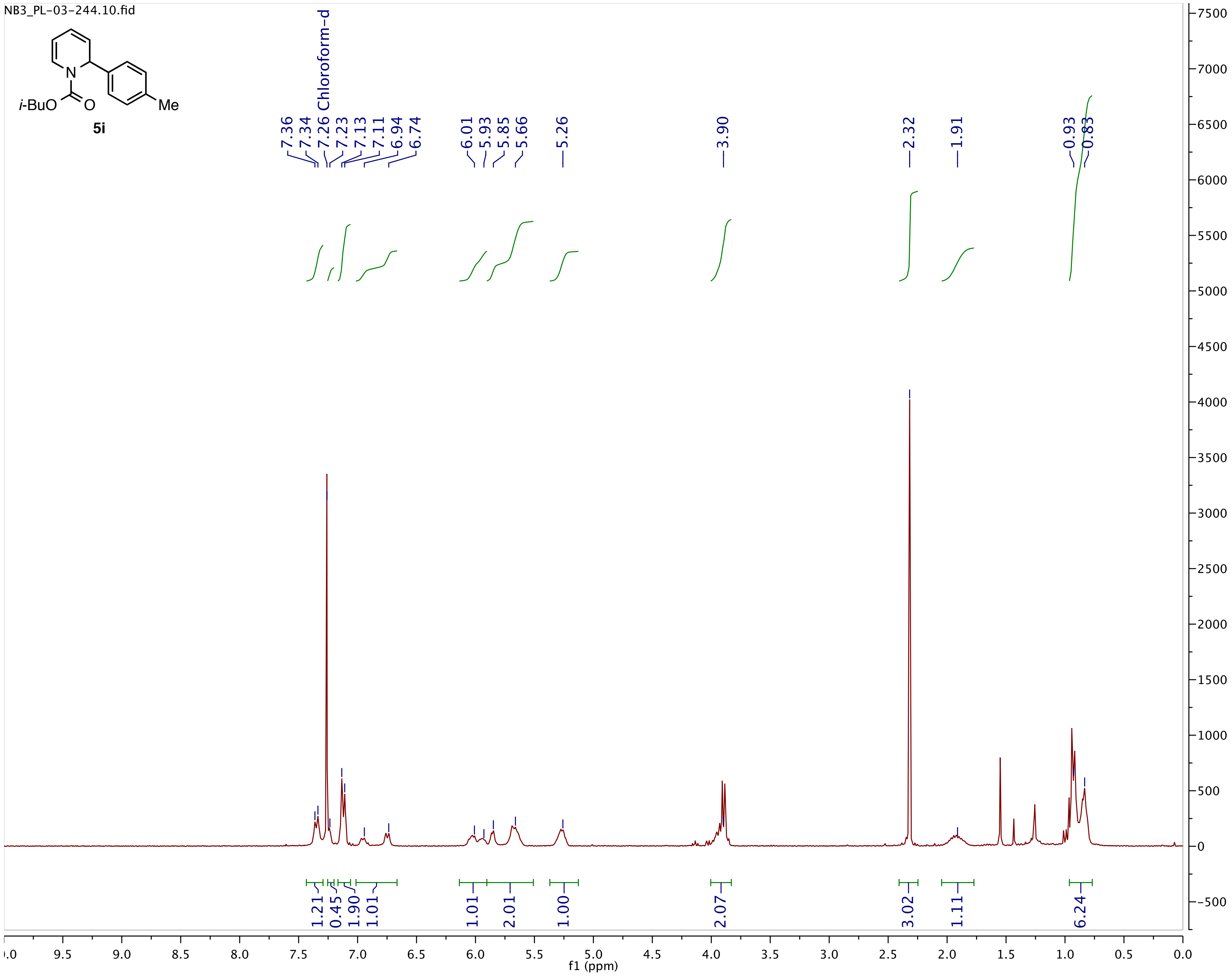

300 MHz ¹H-NMR spectrum of **5i** in $CDCl_3$

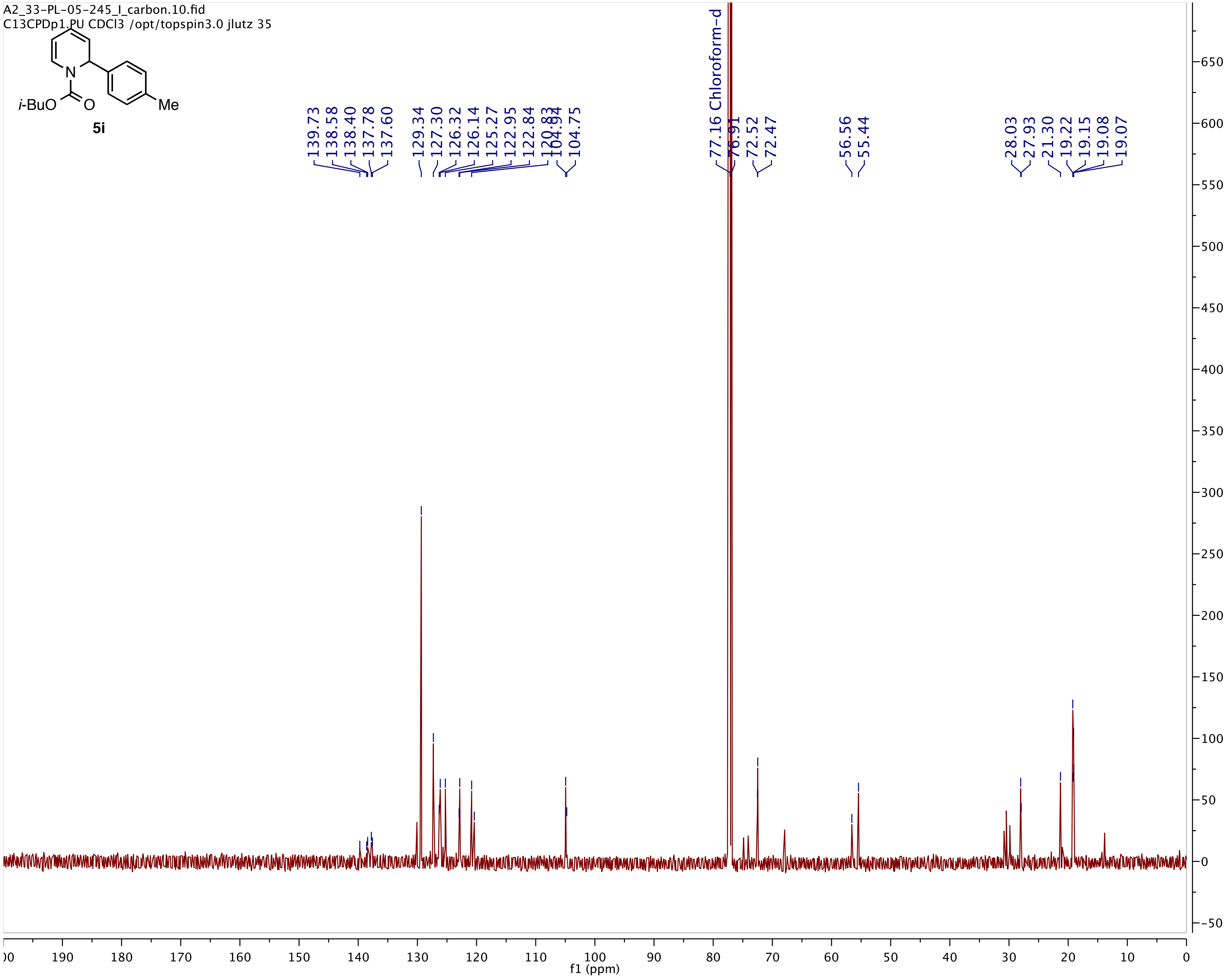

125 MHz ^{13}C-NMR spectrum of **5i** in $CDCl_3$

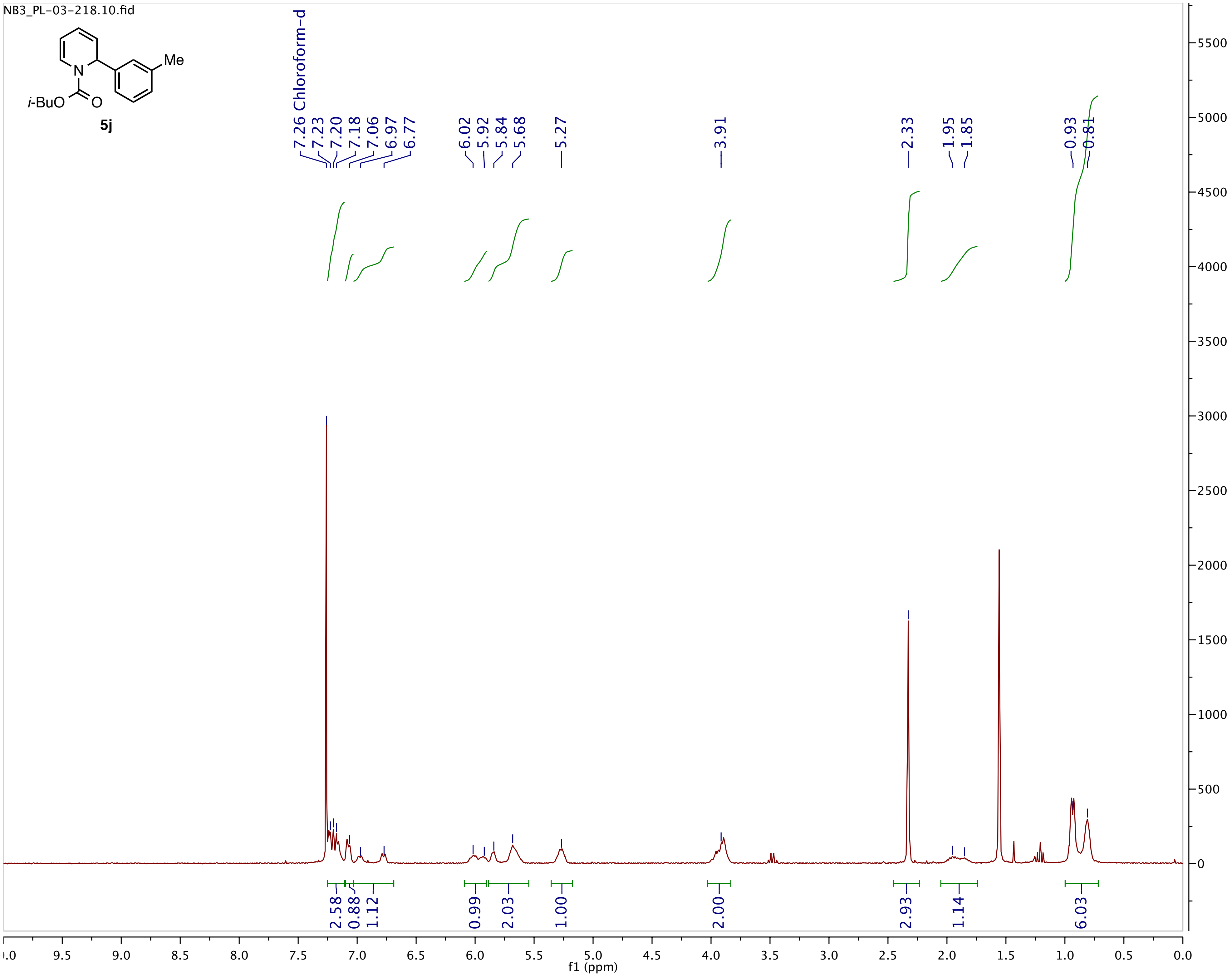

300 MHz ^{1}H-NMR spectrum of **5j** in $CDCl_3$

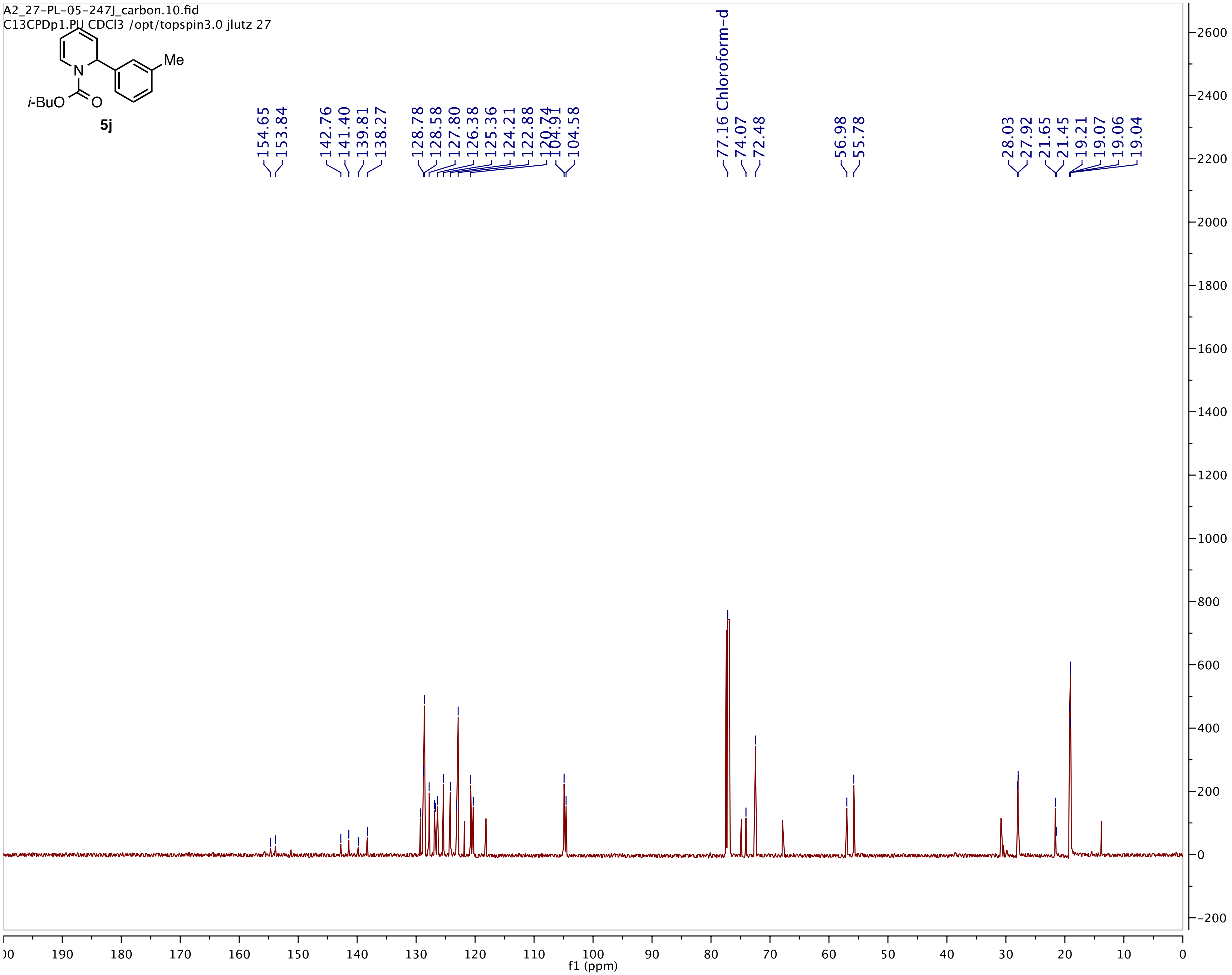

125 MHz ^{13}C-NMR spectrum of **5j** in $CDCl_3$

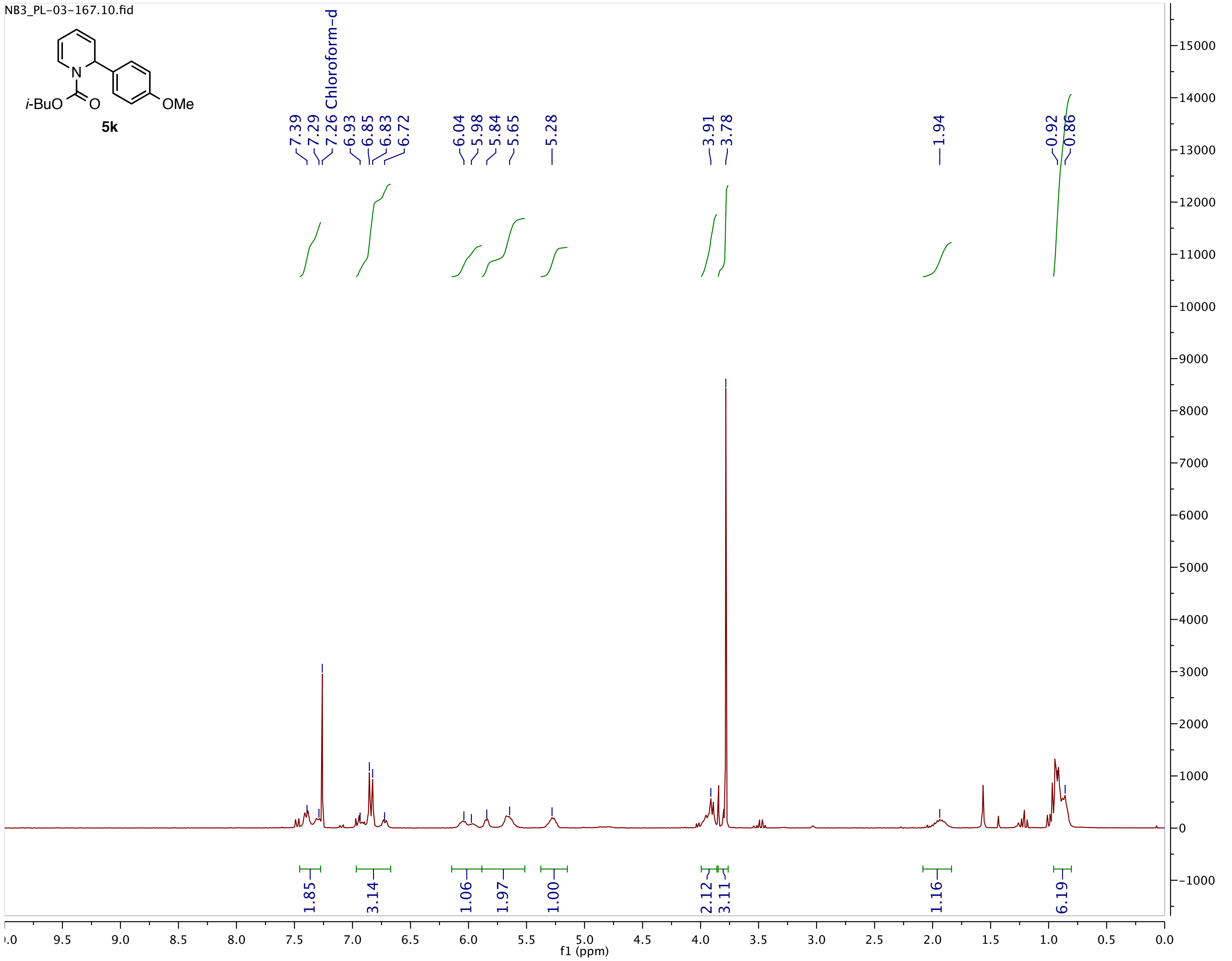

300 MHz ¹H-NMR spectrum of **5k** in $CDCl_3$

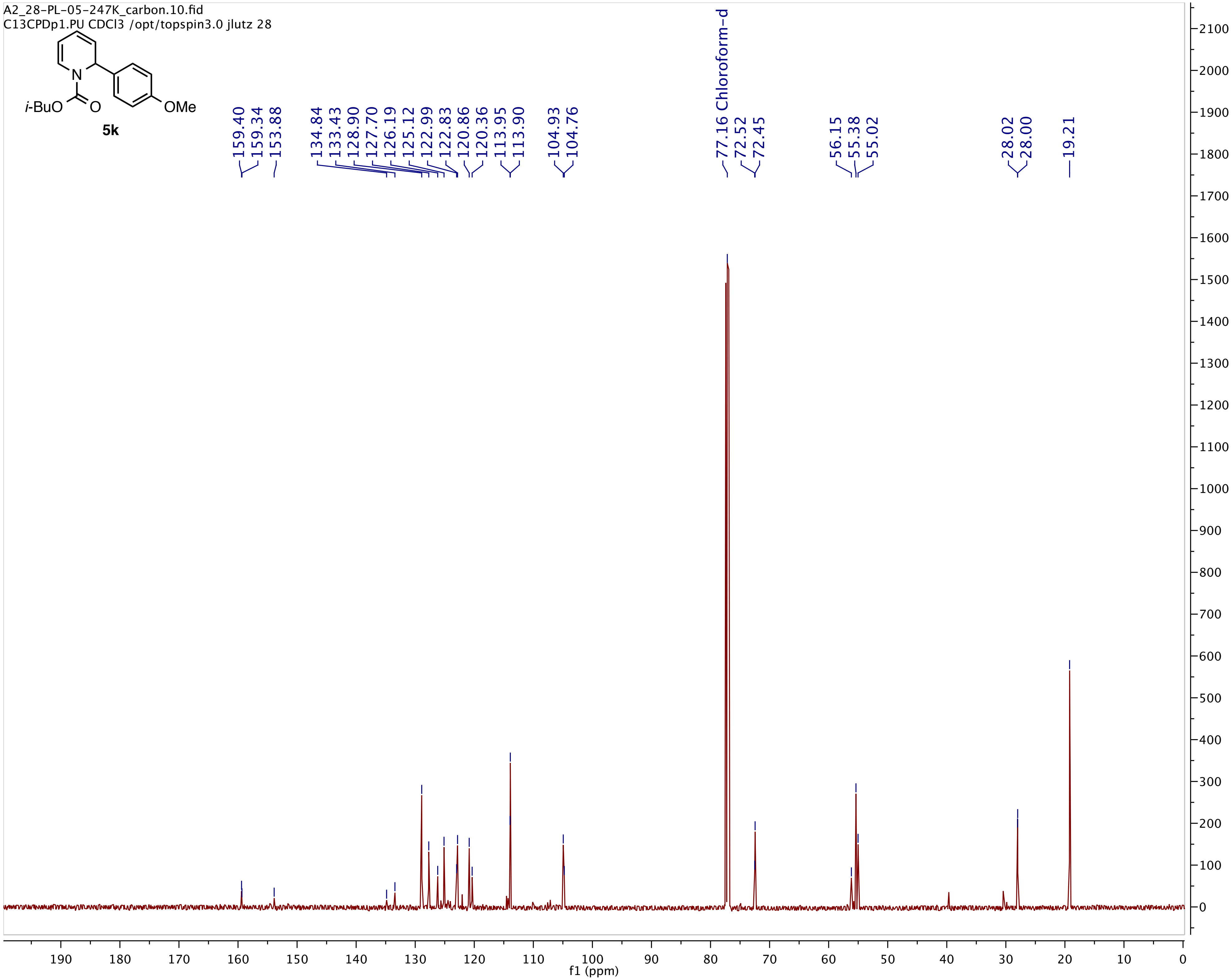

125 MHz ^{13}C-NMR spectrum of **5k** in $CDCl_3$

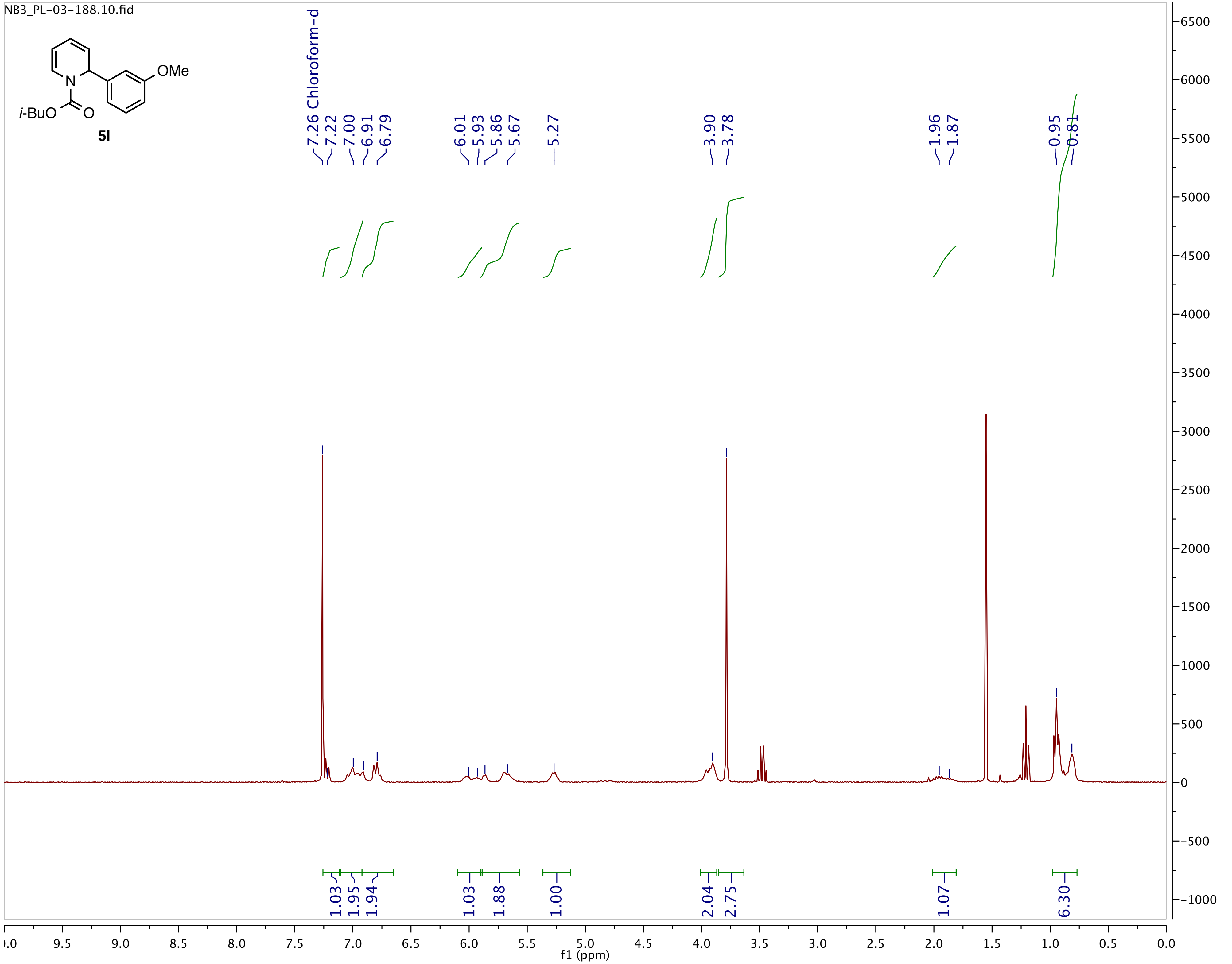

300 MHz ^{1}H-NMR spectrum of **5l** in $CDCl_3$

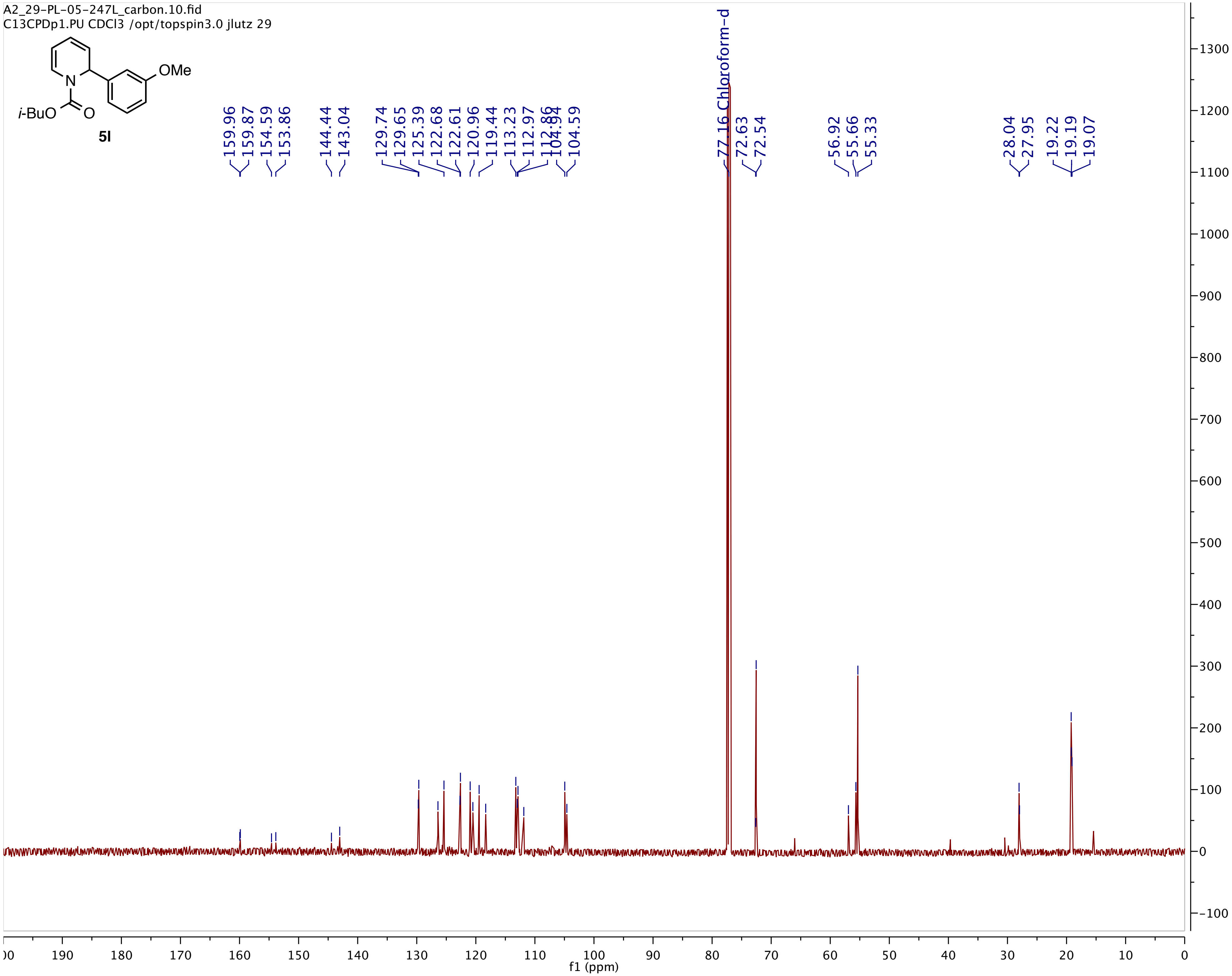

125 MHz ^{13}C-NMR spectrum of **5l** in $CDCl_3$

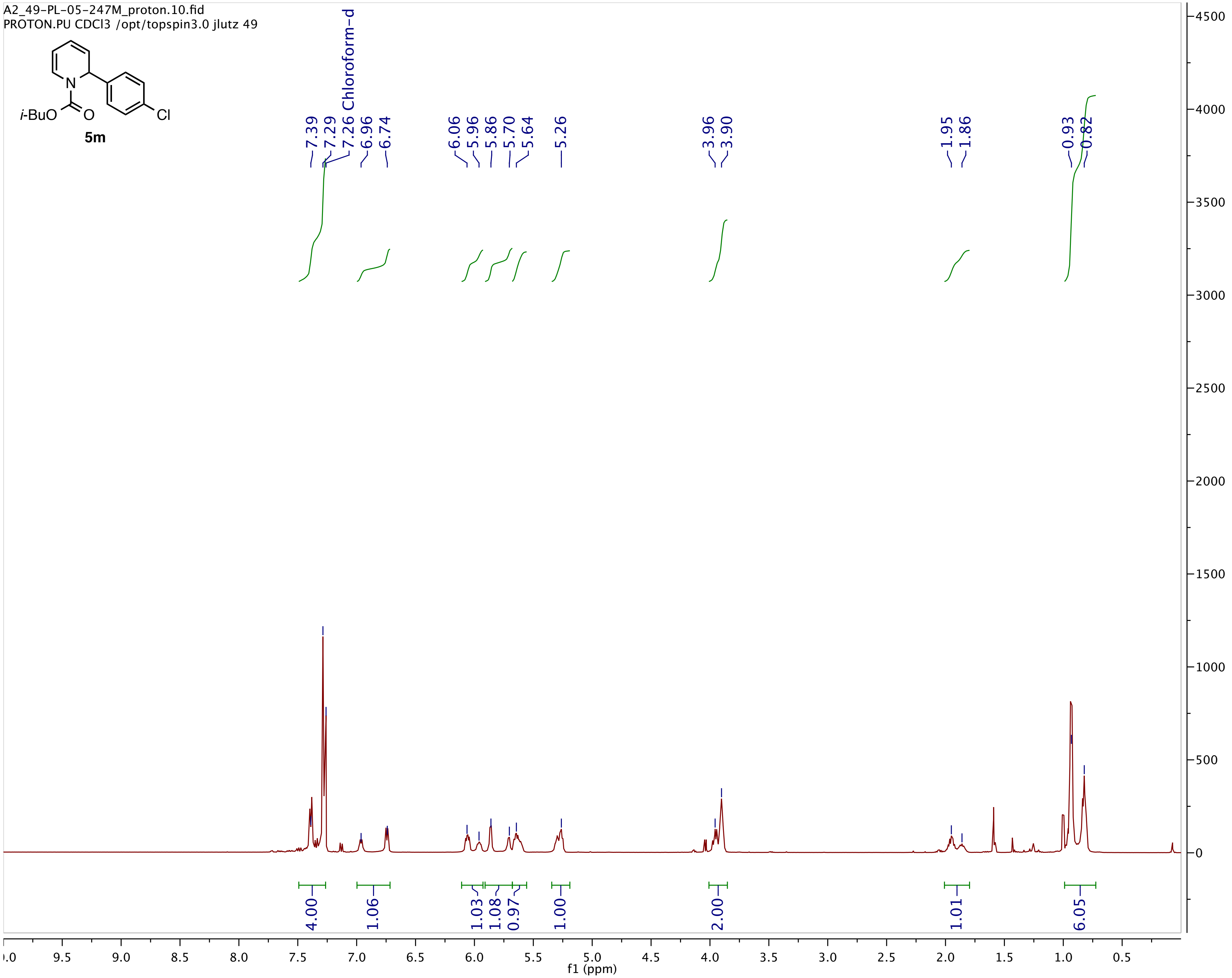

500 MHz ^{1}H-NMR spectrum of **5m** in $CDCl_3$

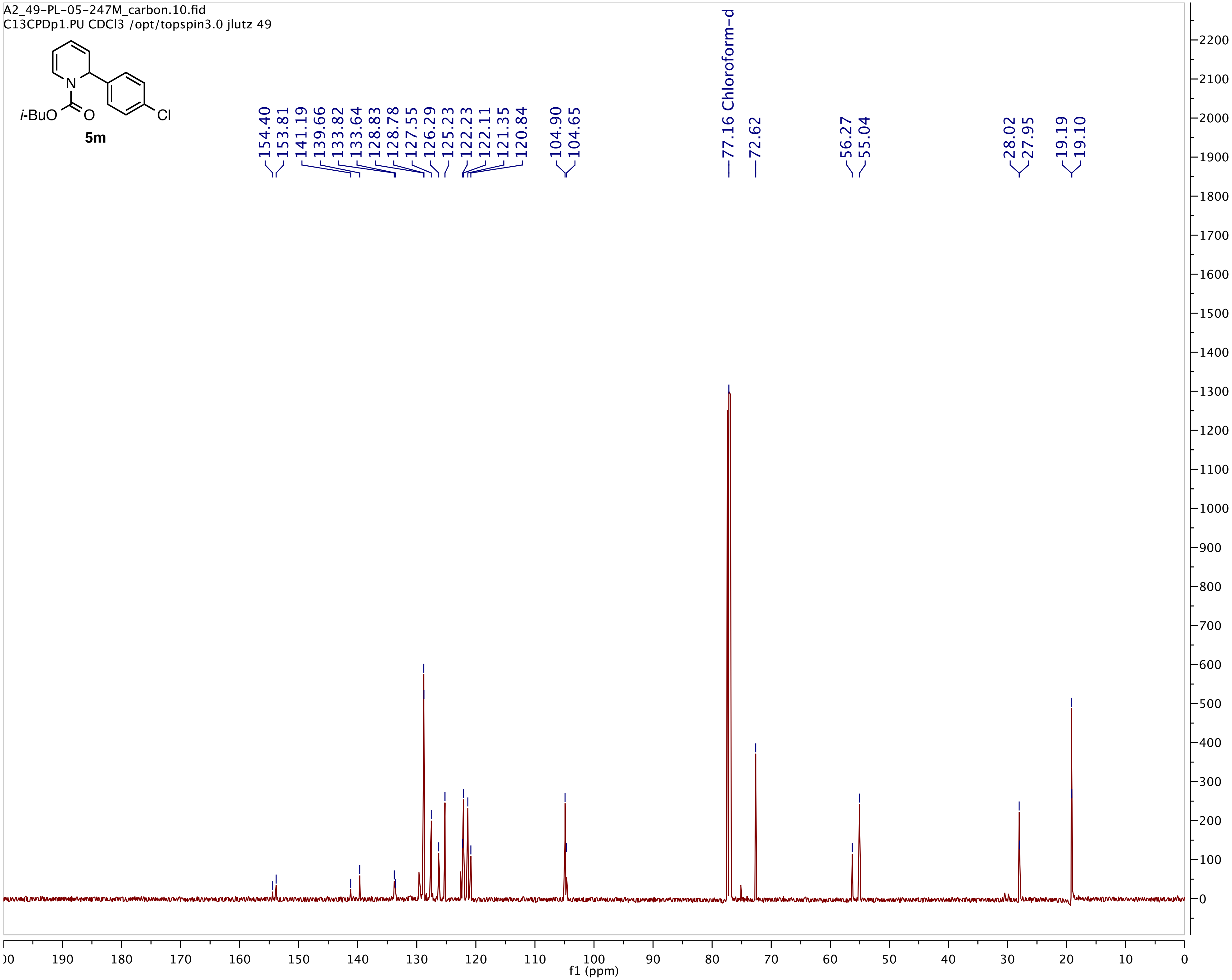

125 MHz ^{13}C-NMR spectrum of **5m** in $CDCl_3$

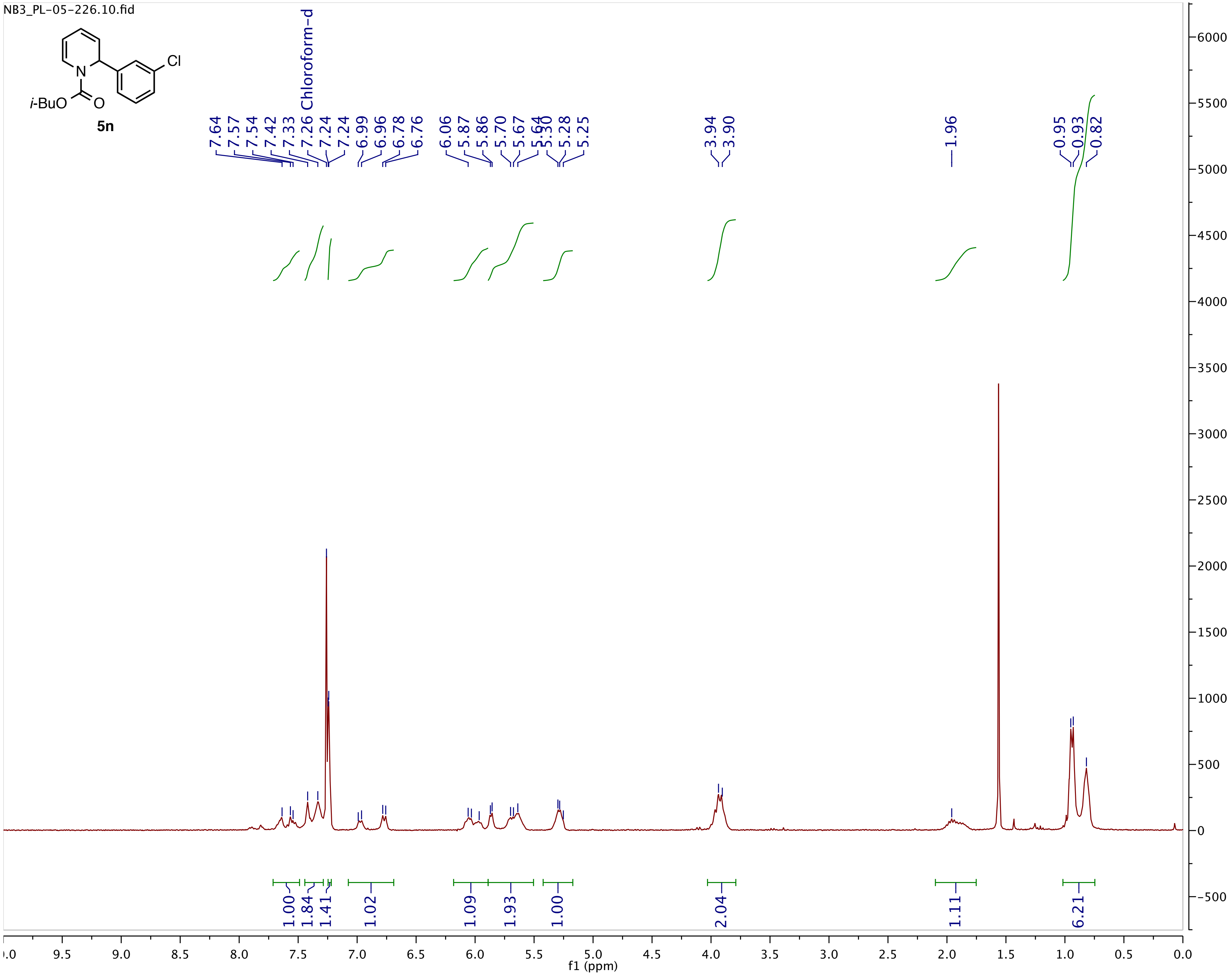

300 MHz ^{1}H-NMR spectrum of **5n** in $CDCl_3$

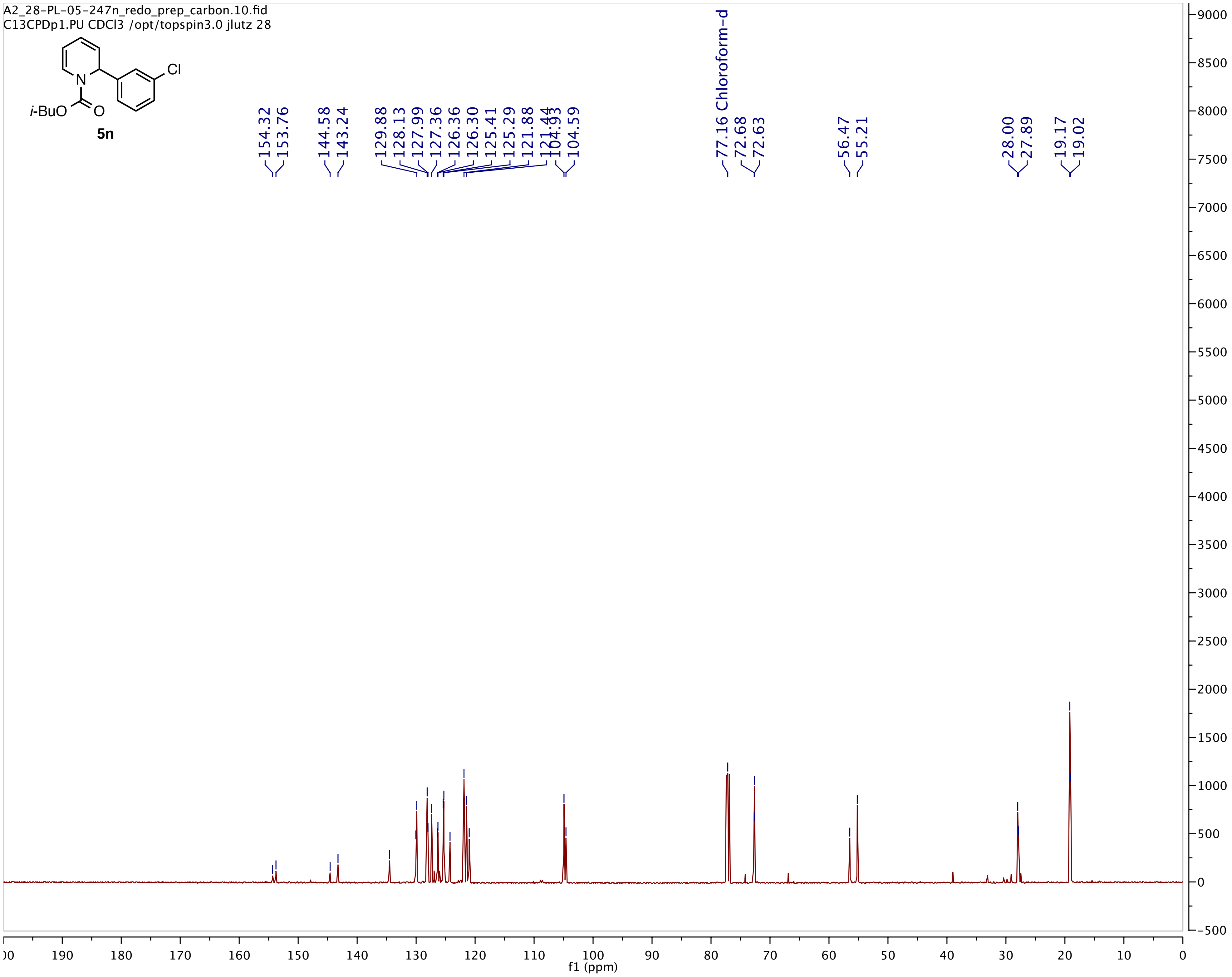

125 MHz ^{13}C-NMR spectrum of **5n** in $CDCl_3$

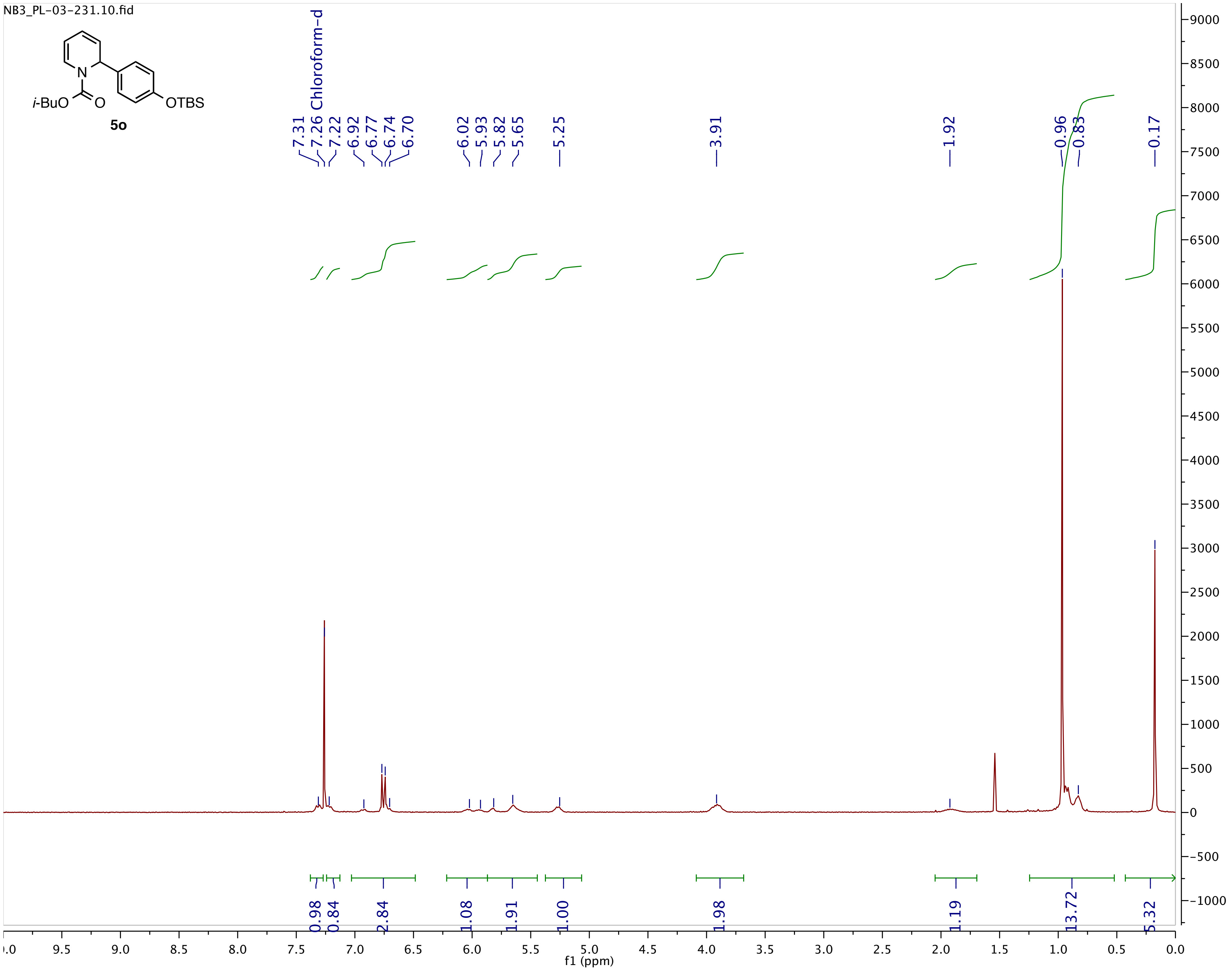

300 MHz ¹H-NMR spectrum of **5o** in $CDCl_3$

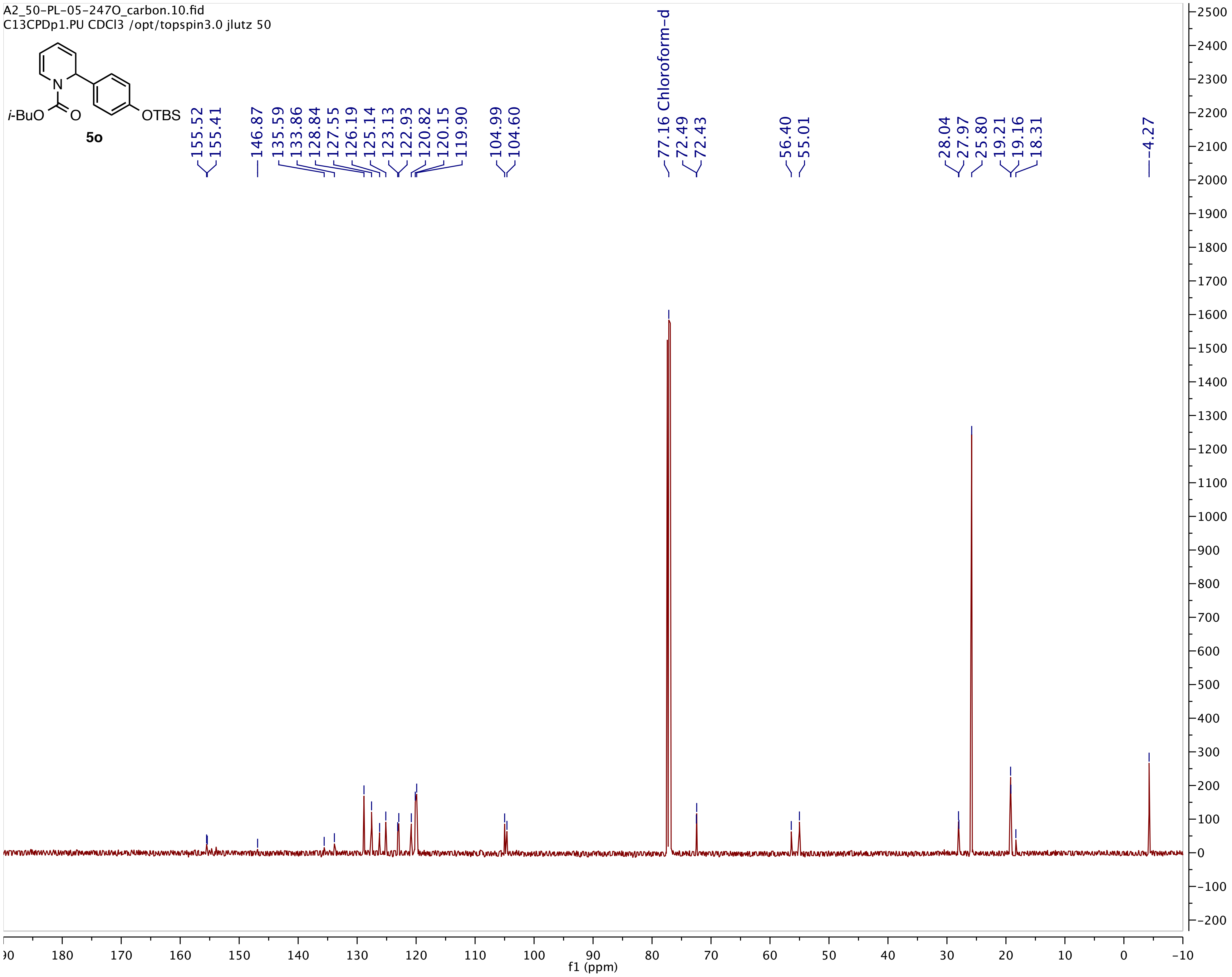

125 MHz ^{13}C-NMR spectrum of **5o** in $CDCl_3$

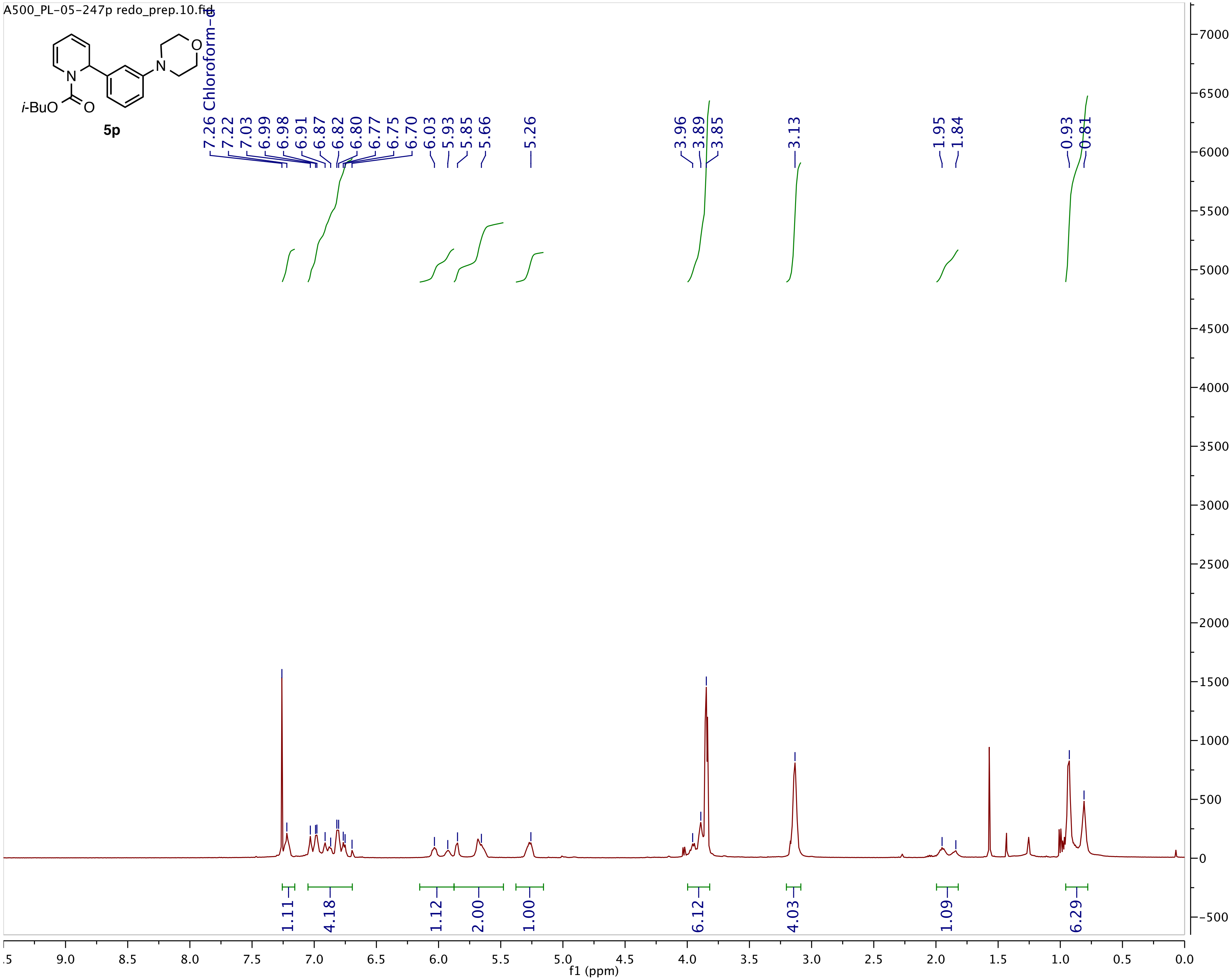

500 MHz ^{1}H-NMR spectrum of **5p** in $CDCl_3$

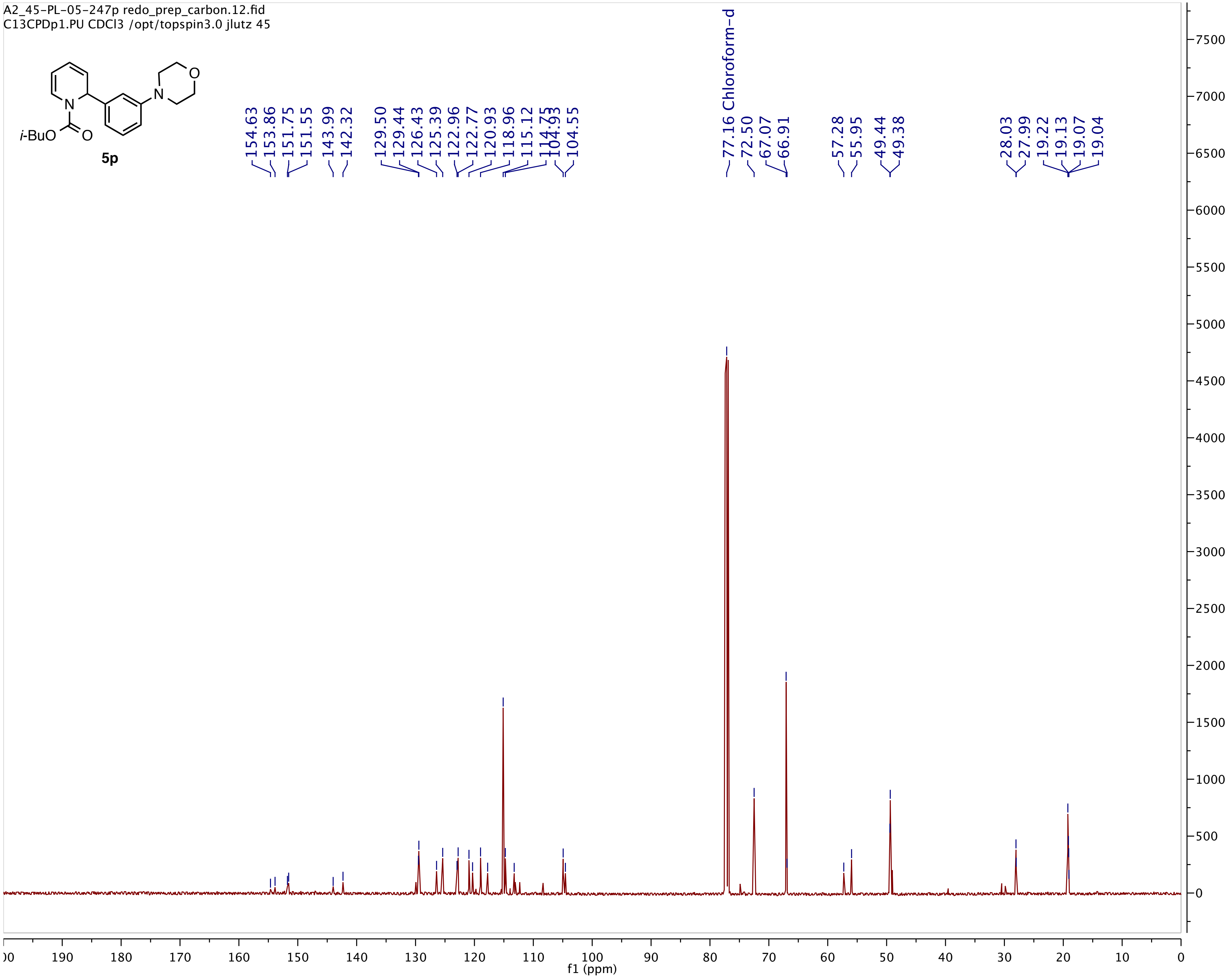

125 MHz ^{13}C-NMR spectrum of **5p** in $CDCl_3$

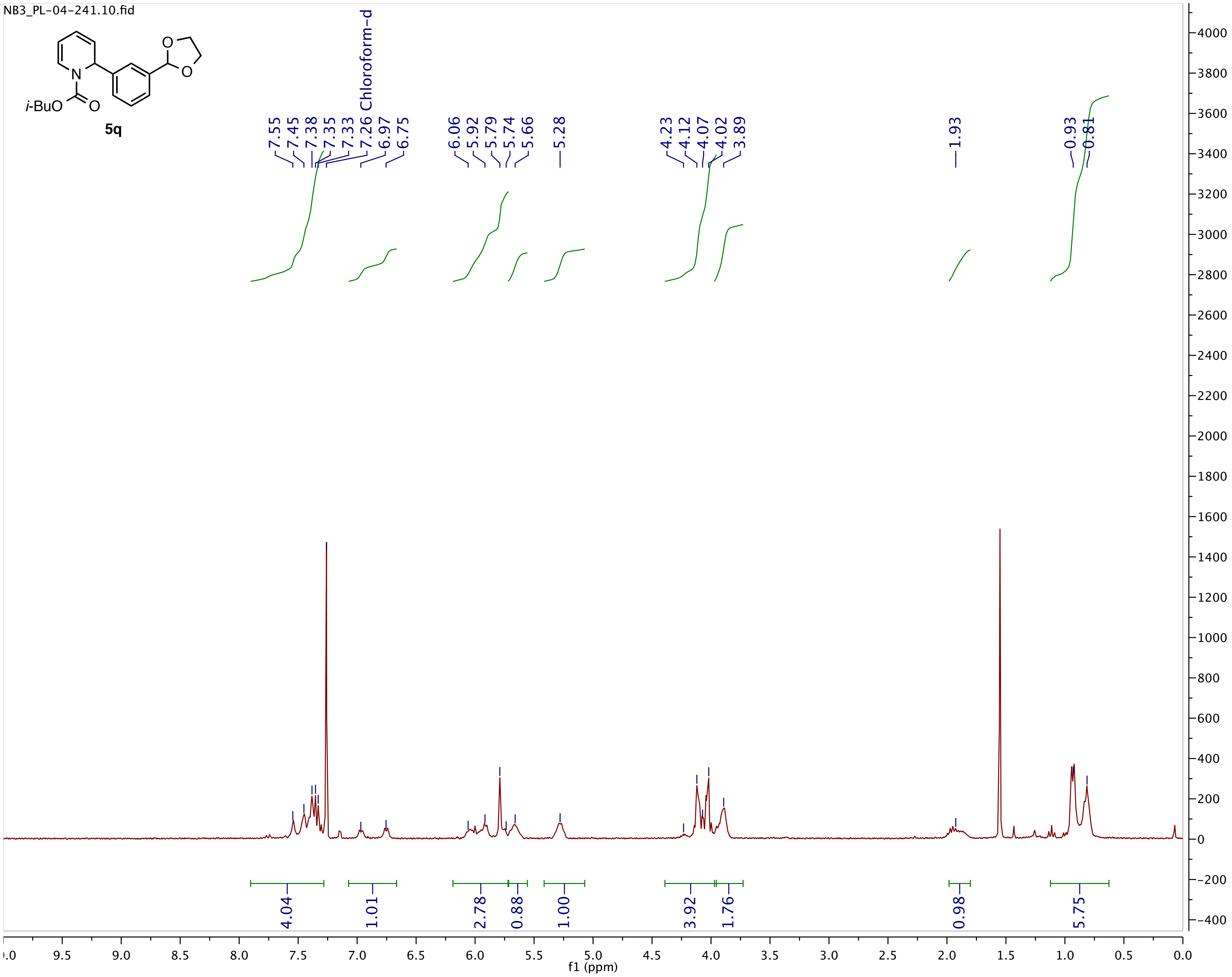

300 MHz ^{1}H-NMR spectrum of **5q** in $CDCl_3$

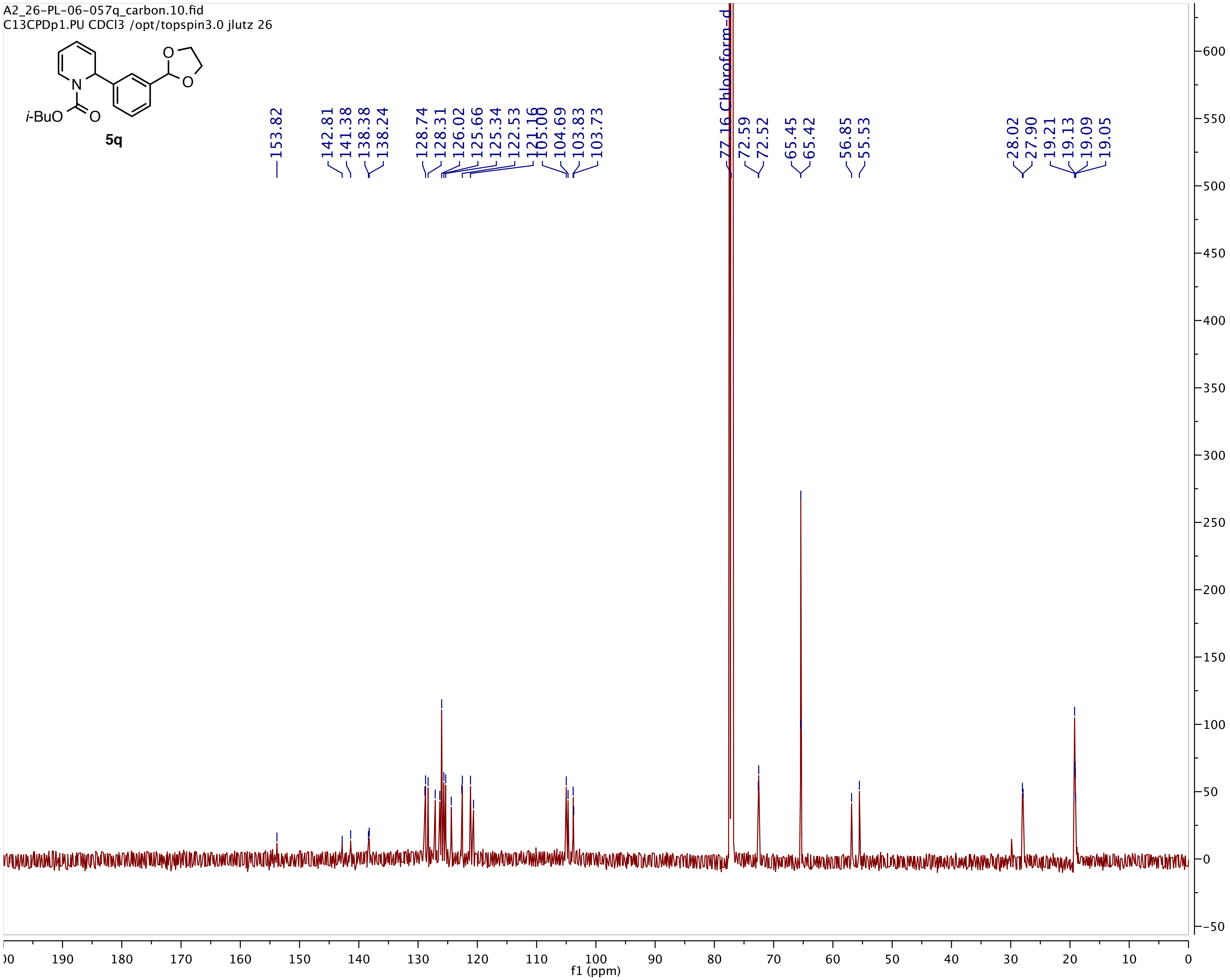

125 MHz ^{13}C-NMR spectrum of **5q** in $CDCl_3$

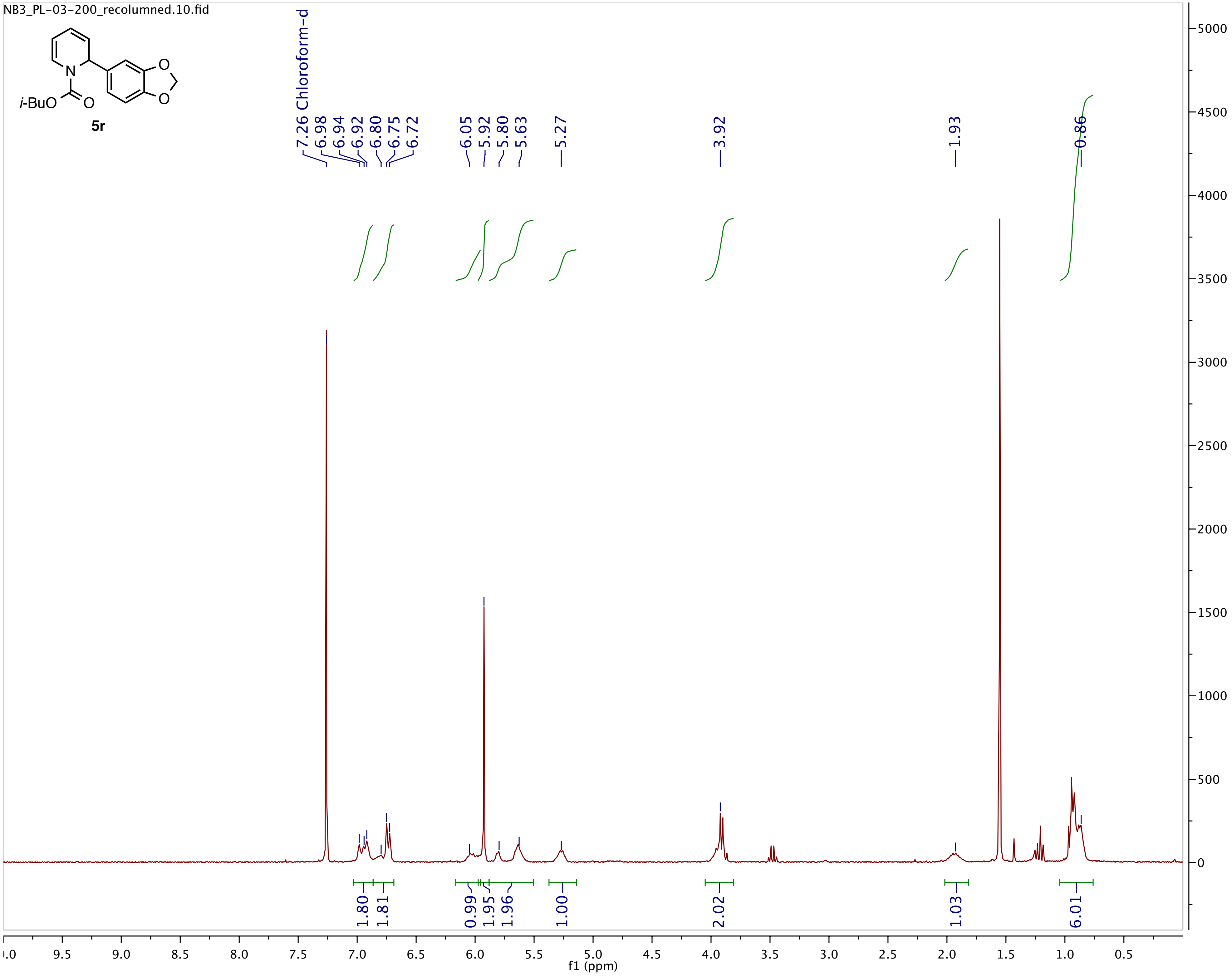

300 MHz ^{1}H-NMR spectrum of **5r** in $CDCl_3$

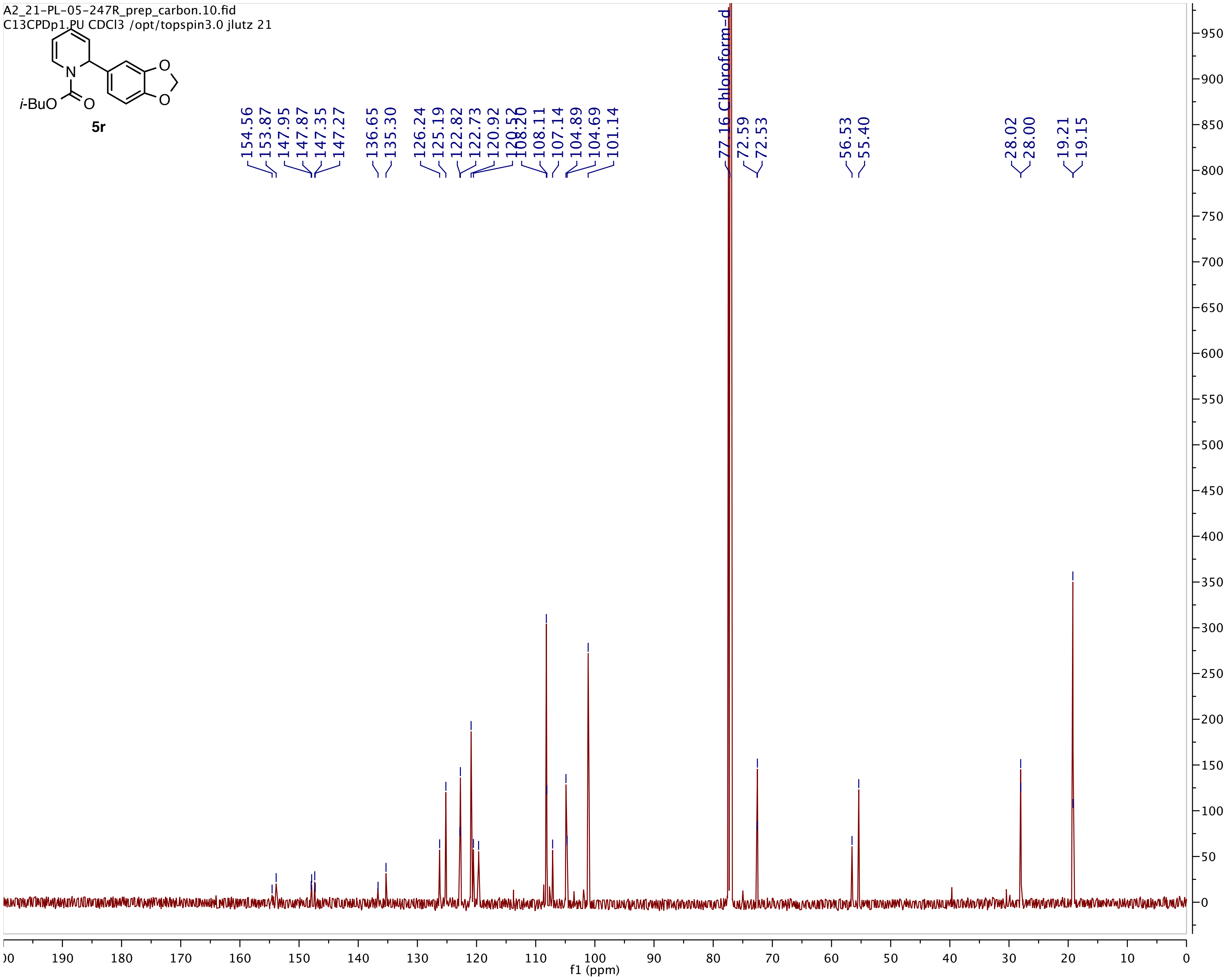

125 MHz ^{13}C-NMR spectrum of **5r** in $CDCl_3$

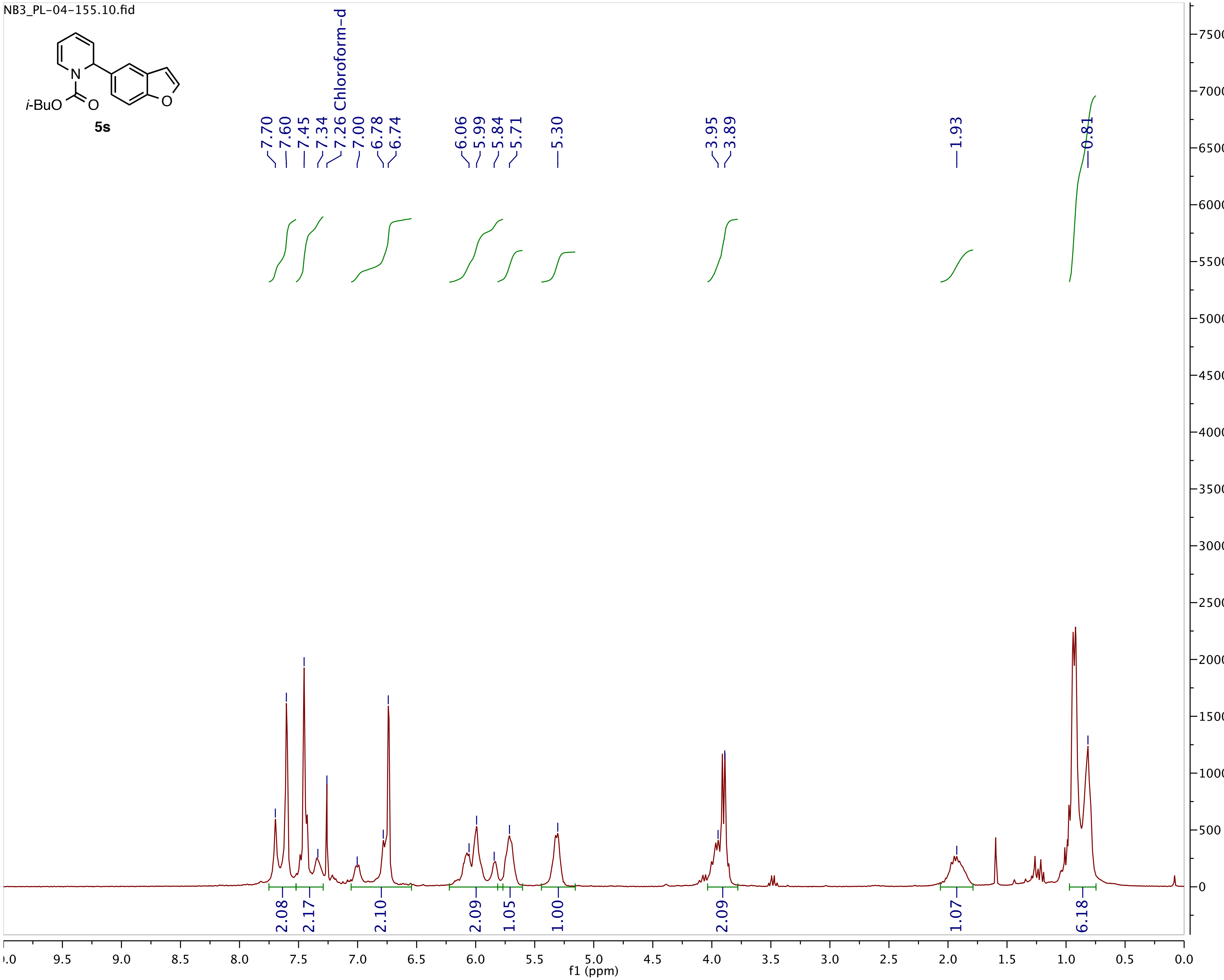

300 MHz ^{1}H-NMR spectrum of **5s** in $CDCl_3$

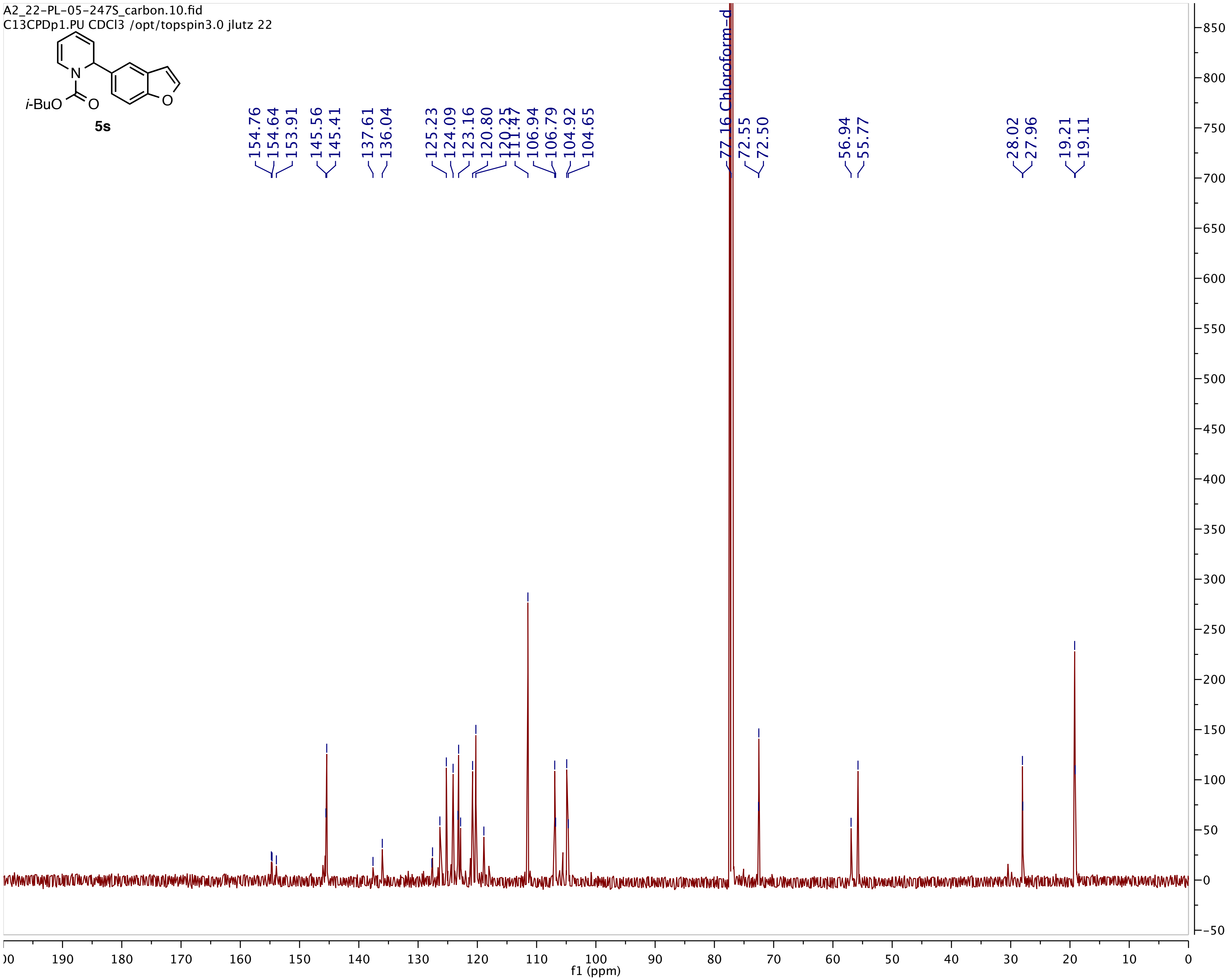

125 MHz ^{13}C-NMR spectrum of **5s** in $CDCl_3$

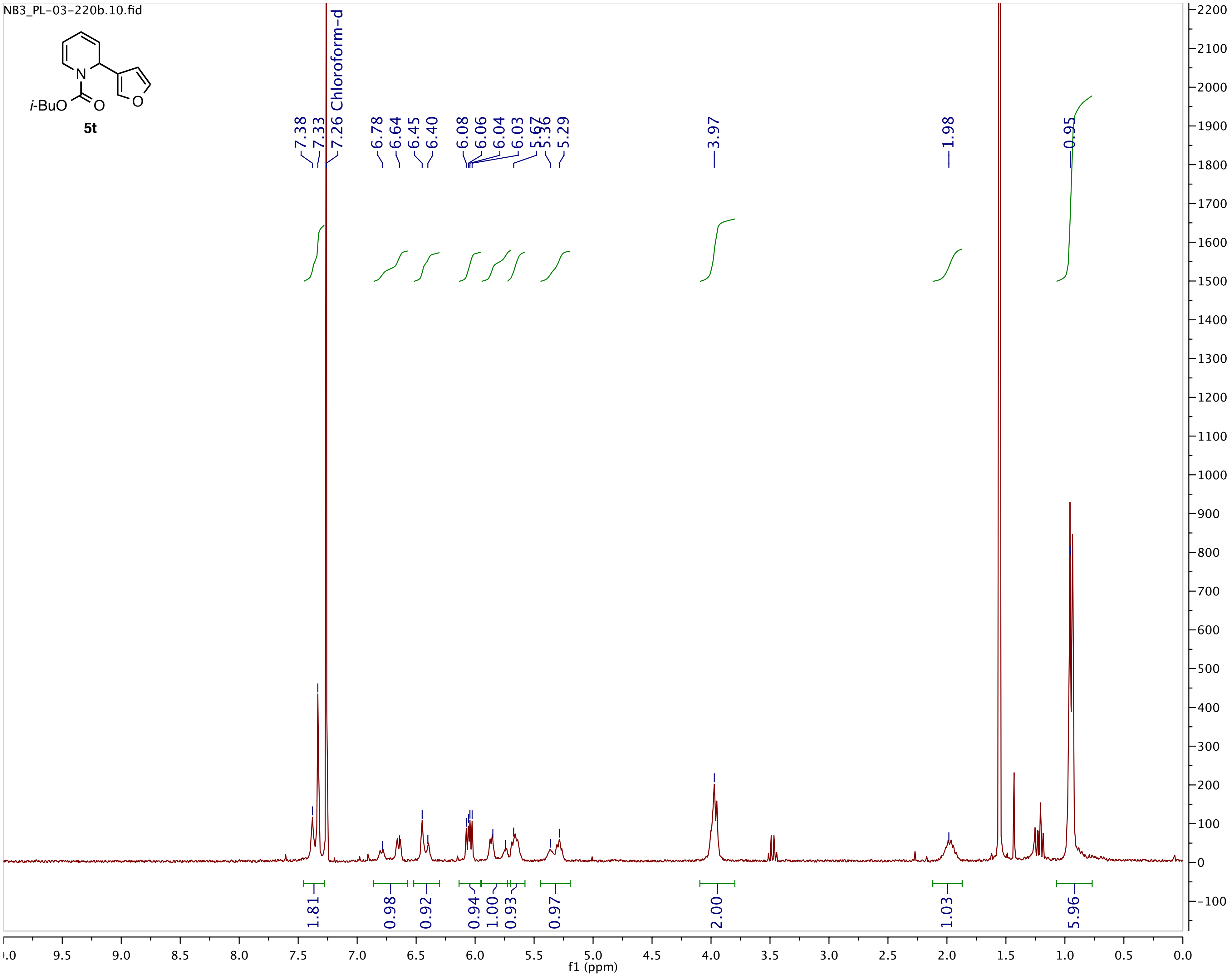

300 MHz ^{1}H-NMR spectrum of **5t** in $CDCl_3$

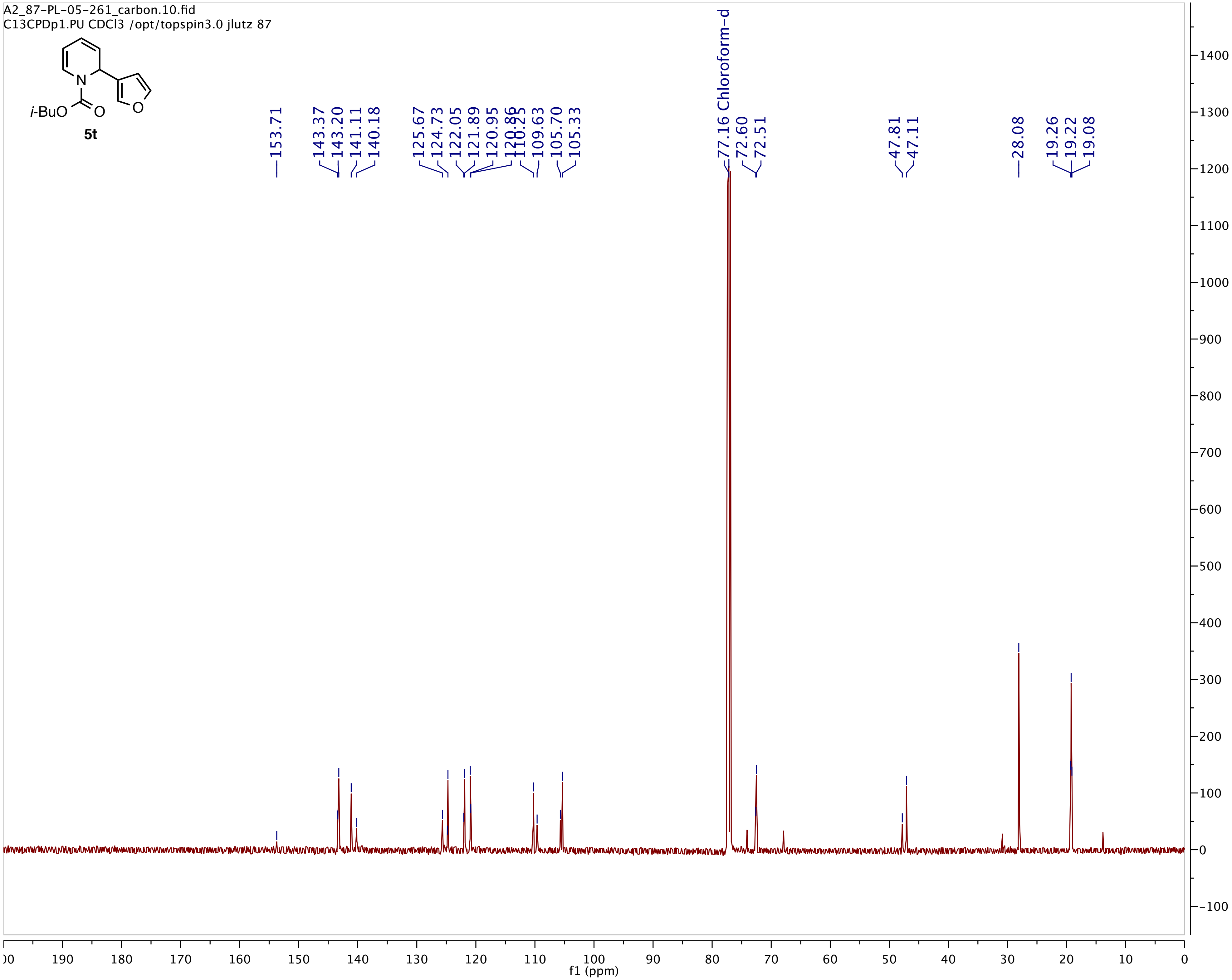

125 MHz ^{13}C-NMR spectrum of **5t** in $CDCl_3$

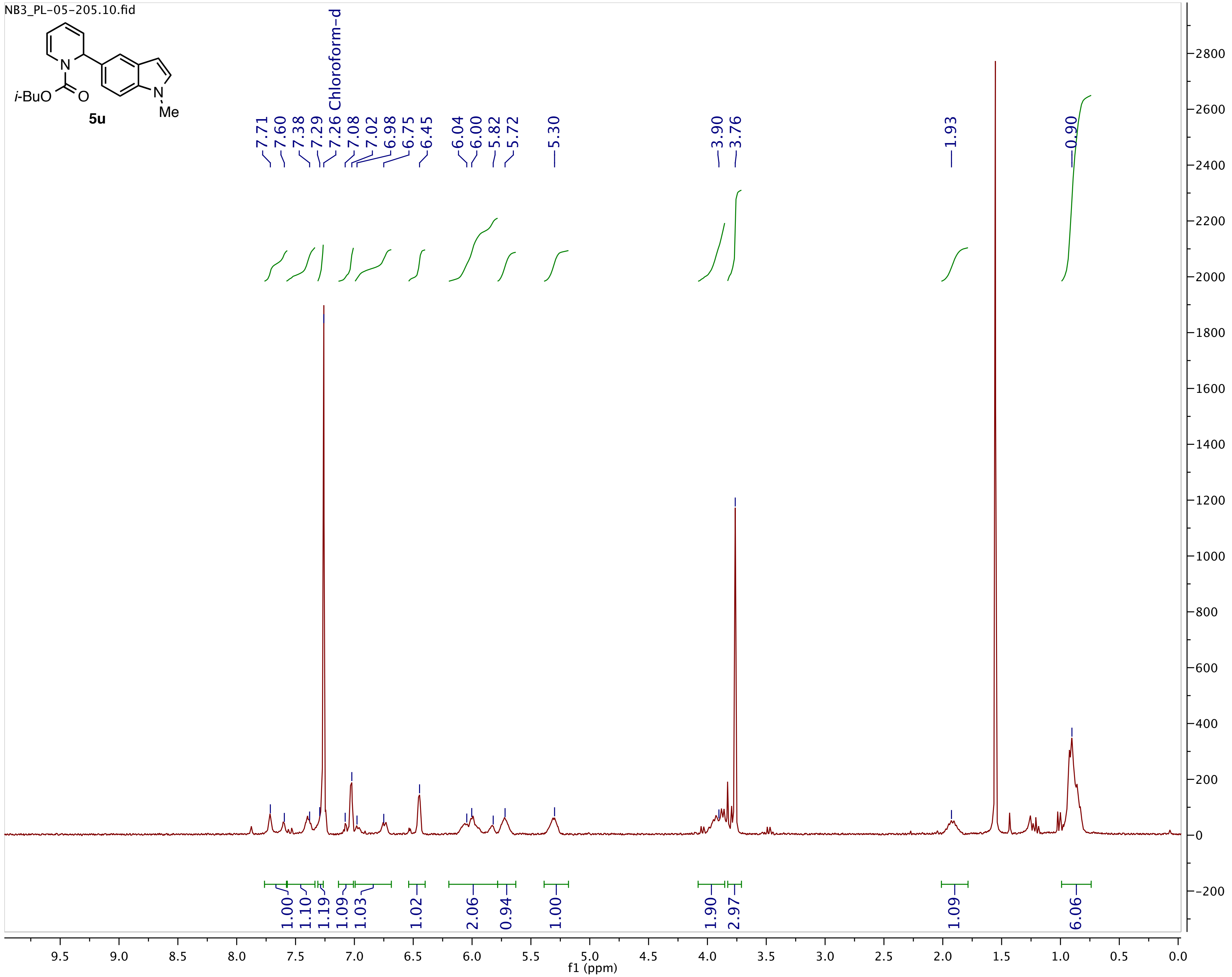

300 MHz ^{1}H-NMR spectrum of **5u** in $CDCl_3$

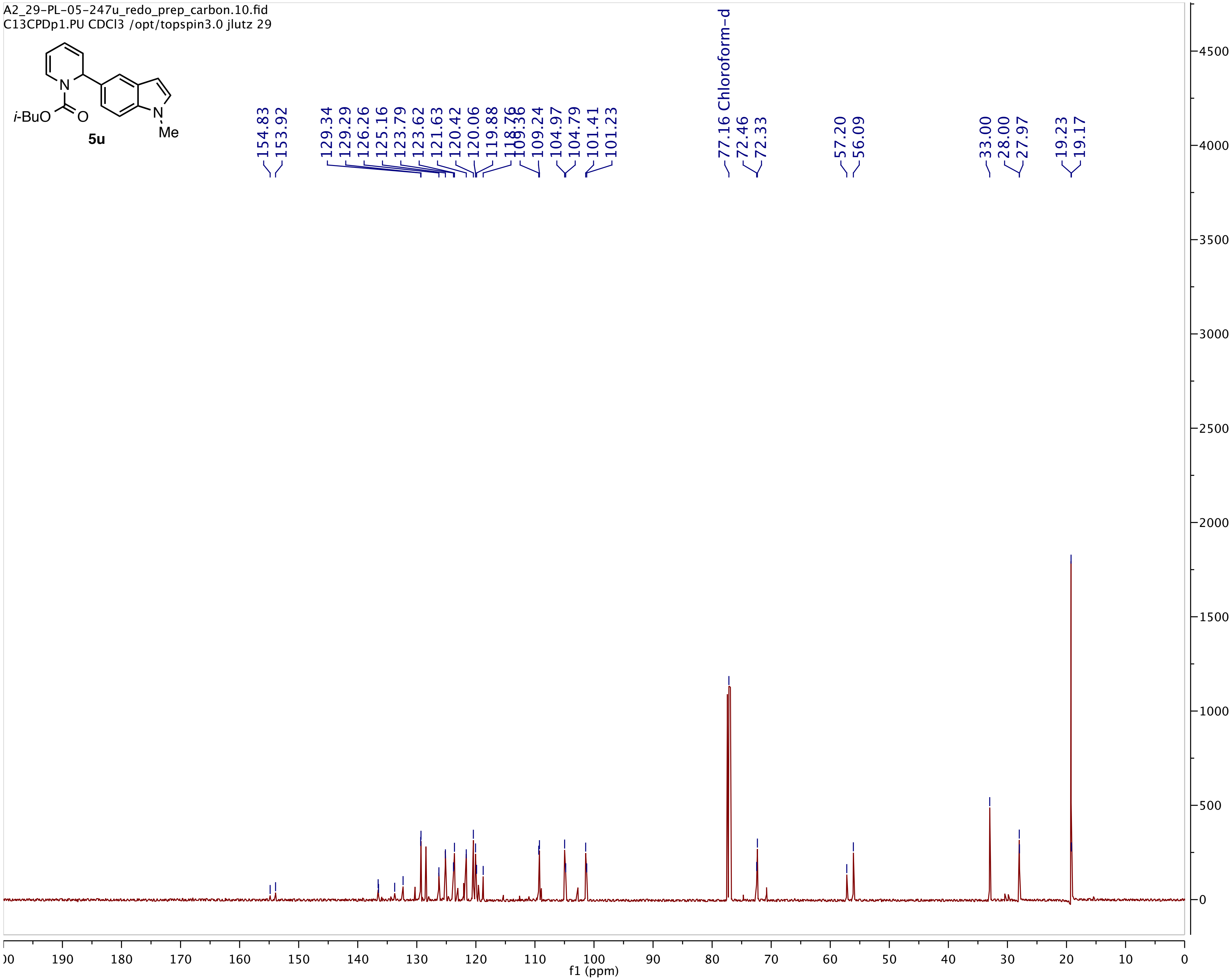

125 MHz ^{13}C-NMR spectrum of **5u** in $CDCl_3$

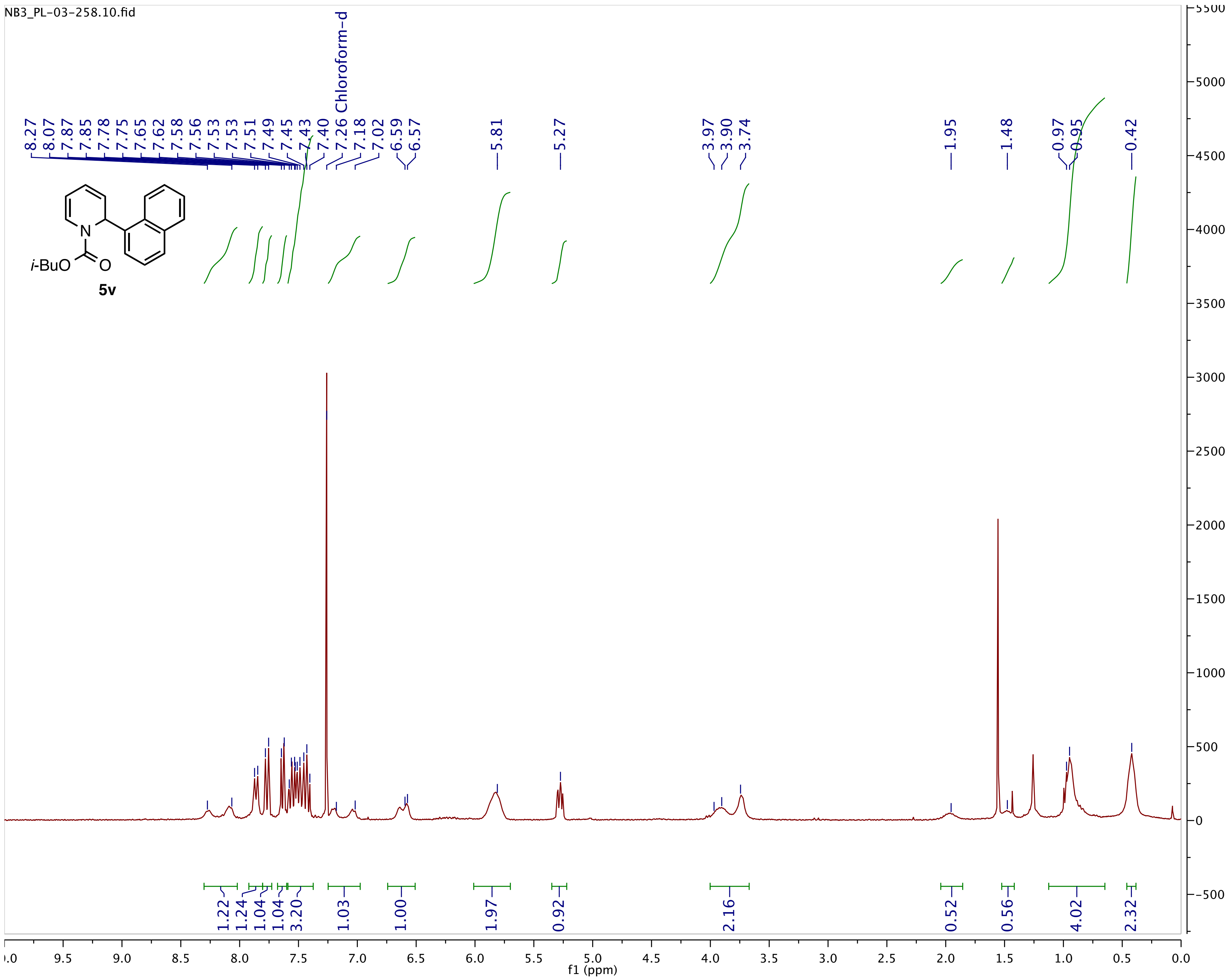

300 MHz ^{1}H-NMR spectrum of **5v** in $CDCl_3$

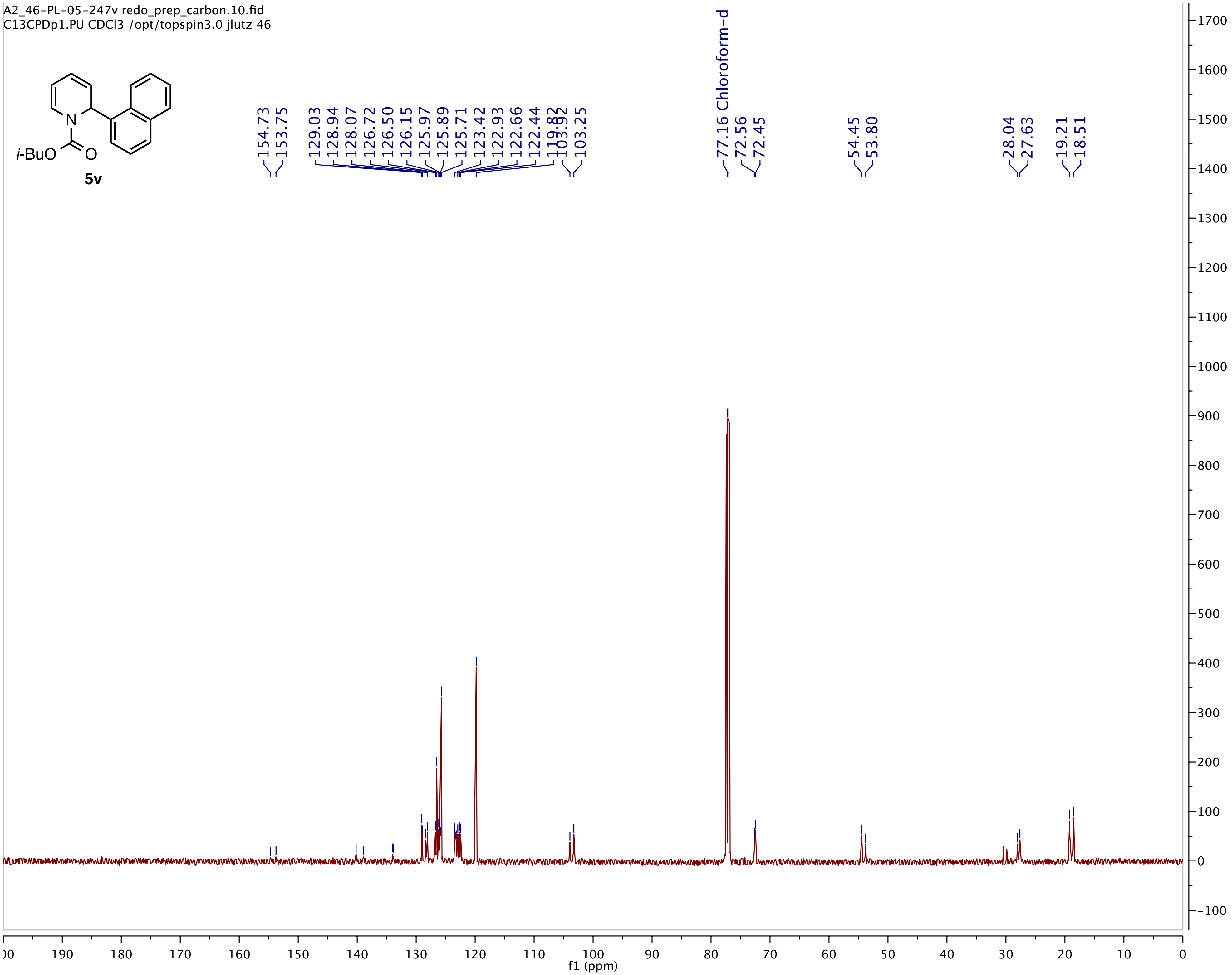

125 MHz ^{13}C-NMR spectrum of **5v** in $CDCl_3$

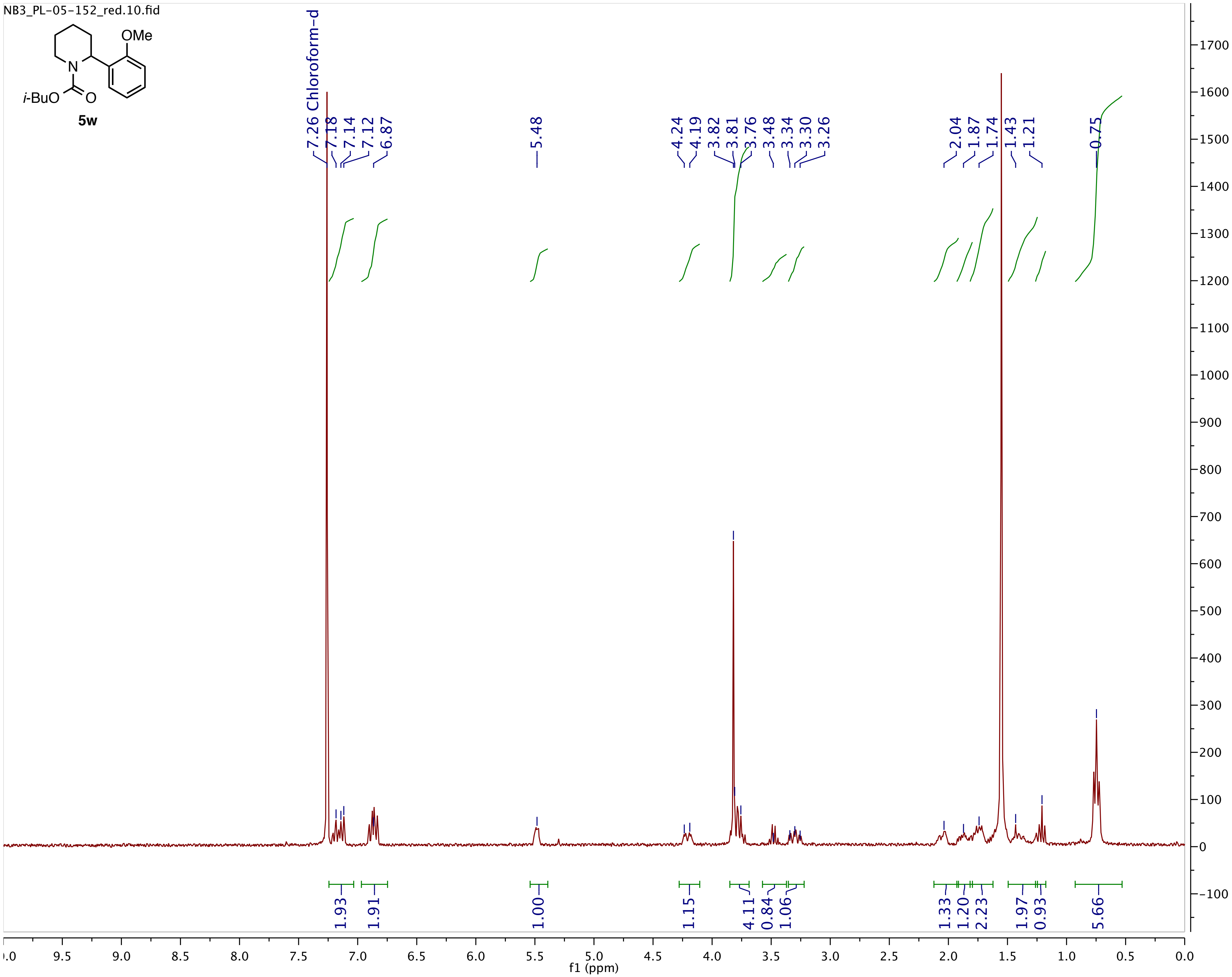

300 MHz ^{1}H-NMR spectrum of **5w** in $CDCl_3$

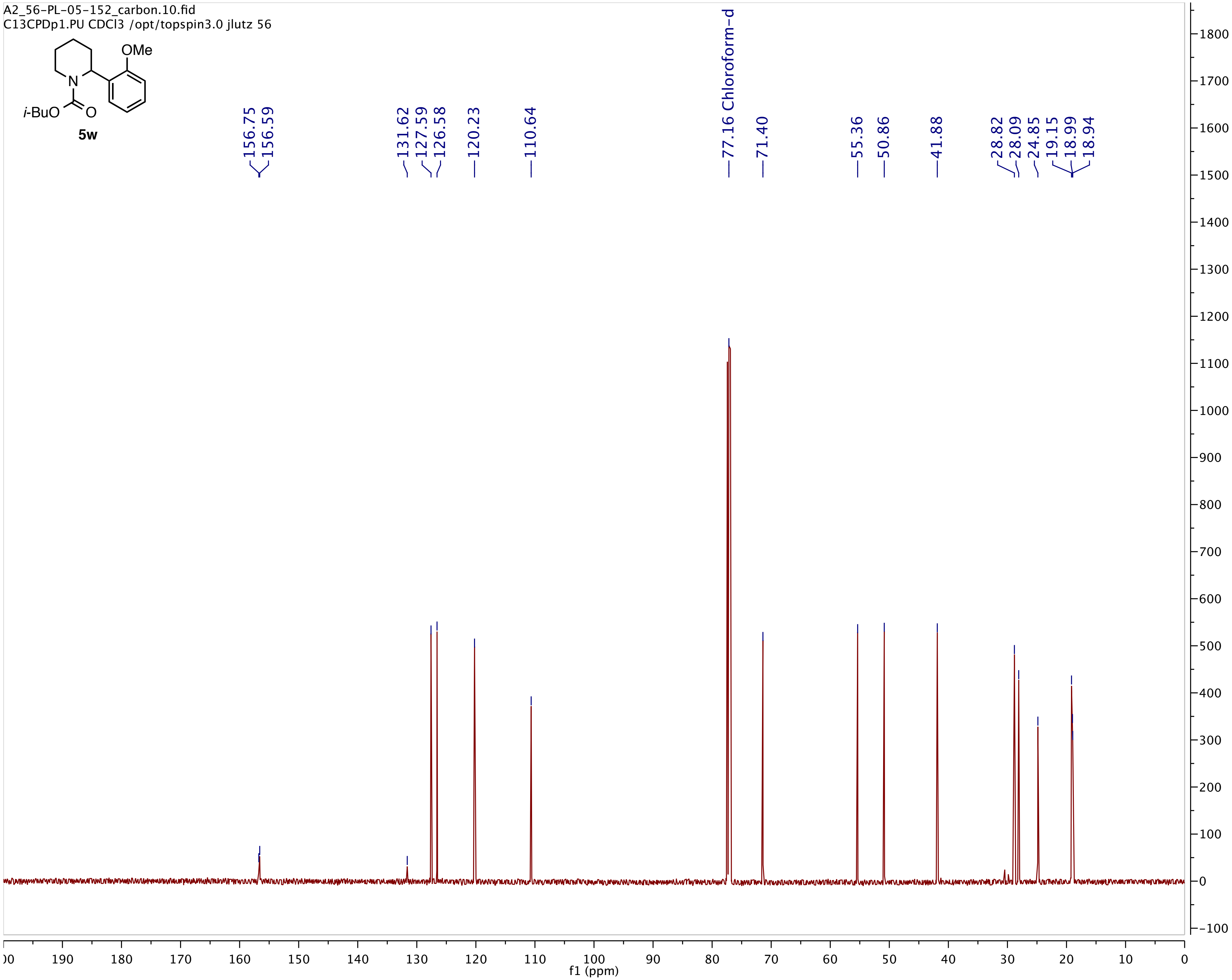

125 MHz ^{13}C-NMR spectrum of **5w** in $CDCl_3$

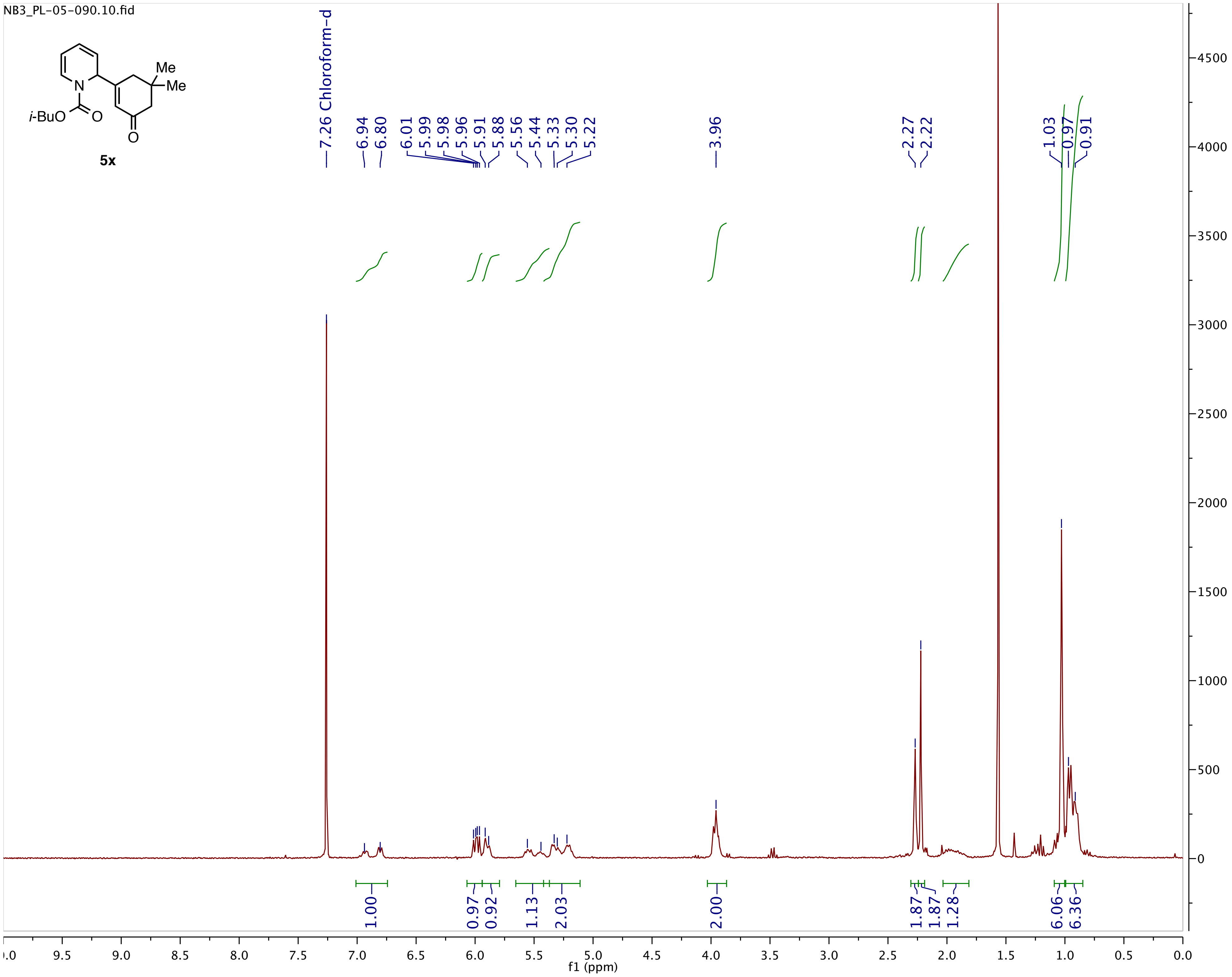

300 MHz ^{1}H-NMR spectrum of **5x** in $CDCl_3$

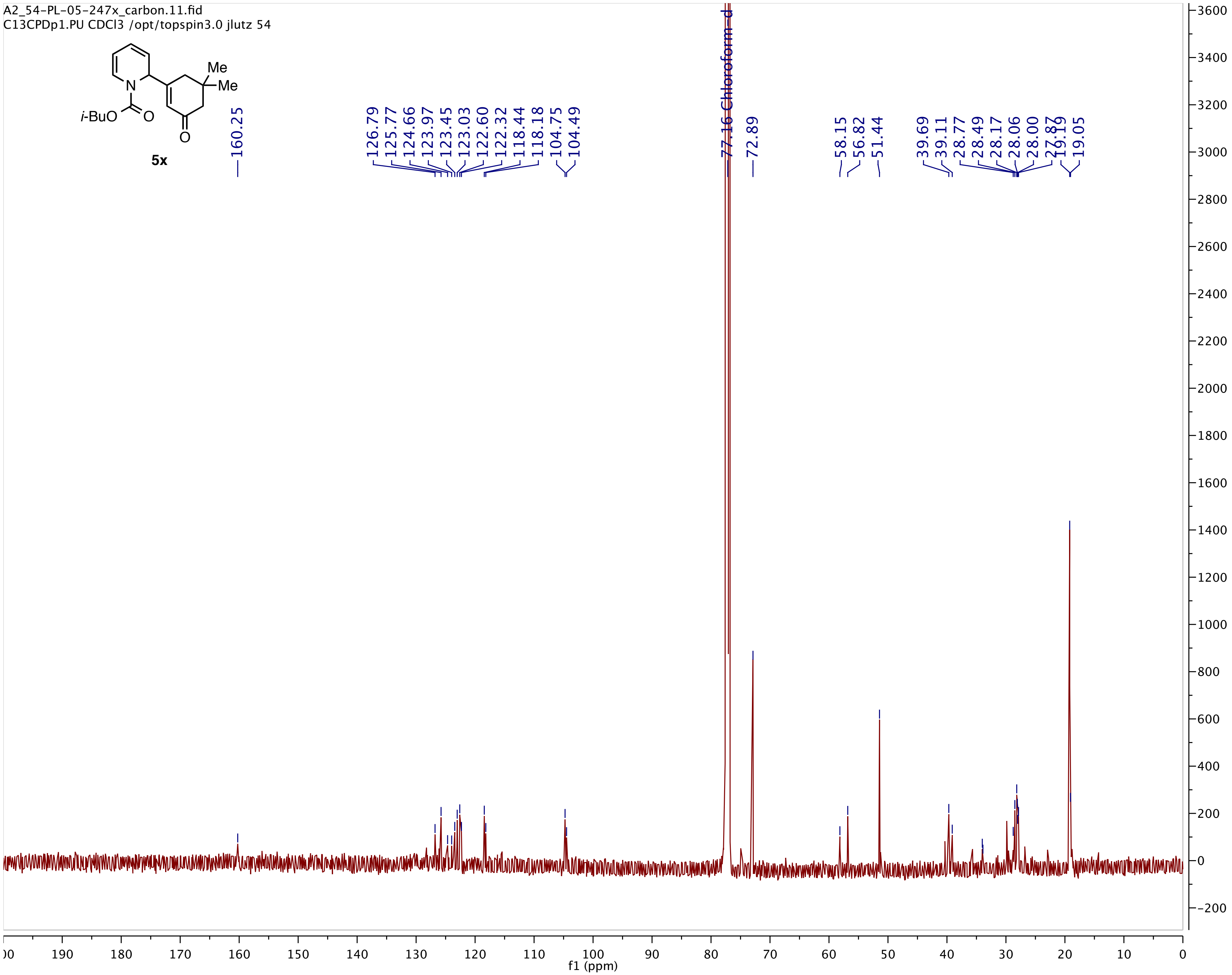

125 MHz ^{13}C-NMR spectrum of **5x** in $CDCl_3$

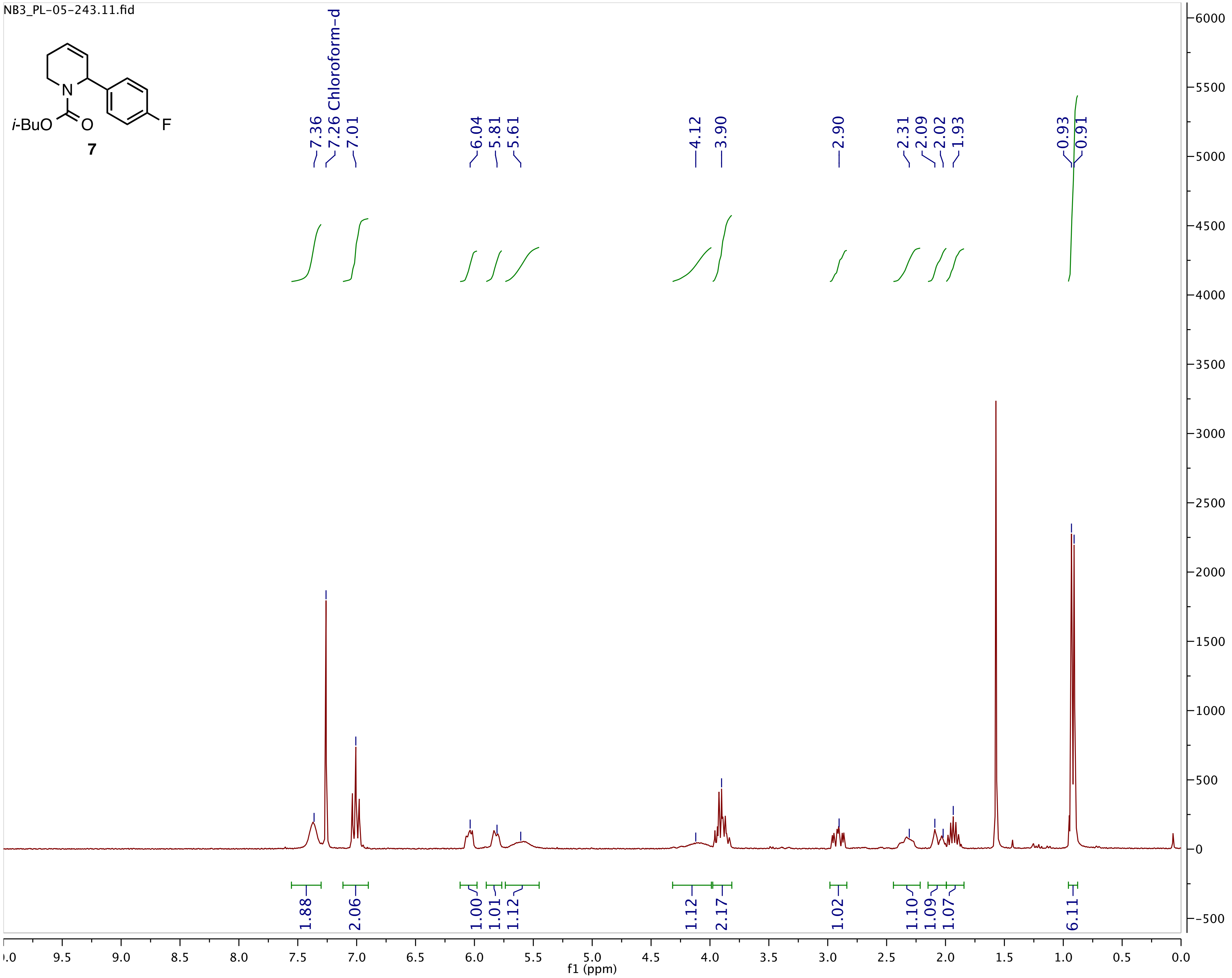

300 MHz ^{1}H-NMR spectrum of **7** in $CDCl_3$

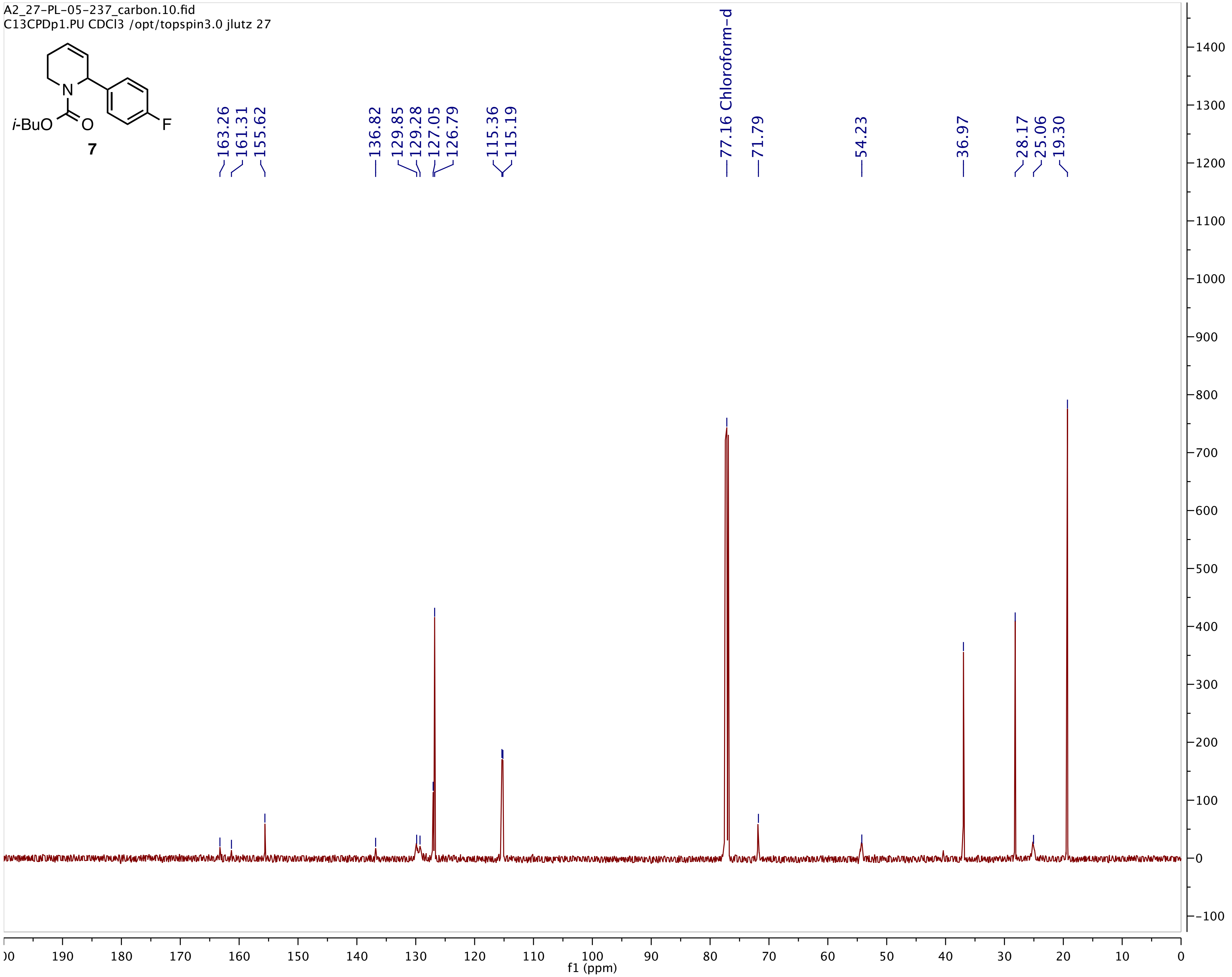

125 MHz ^{13}C-NMR spectrum of **7** in $CDCl_3$

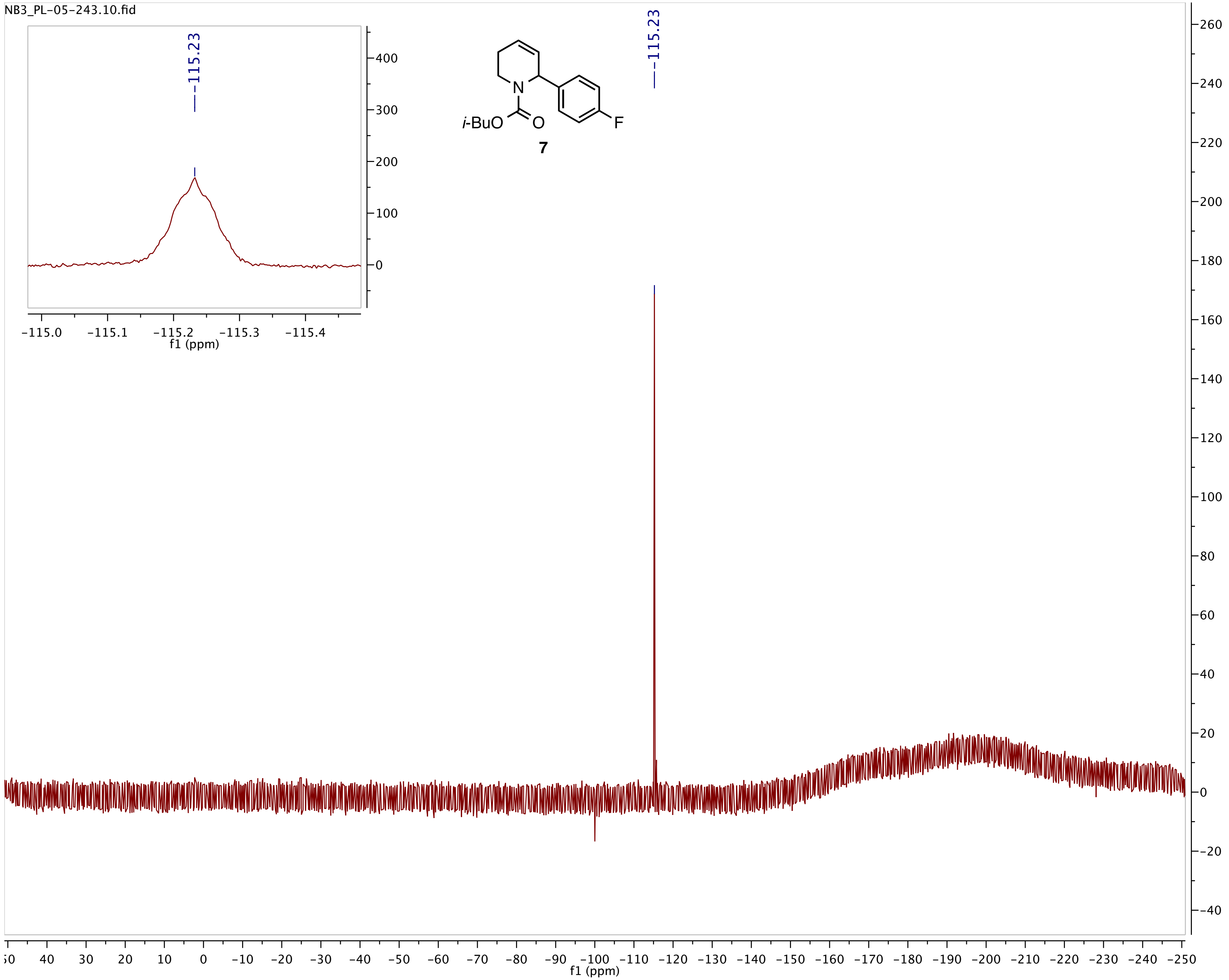

282 MHz ^{19}F-NMR spectrum of **7** in $CDCl_3$

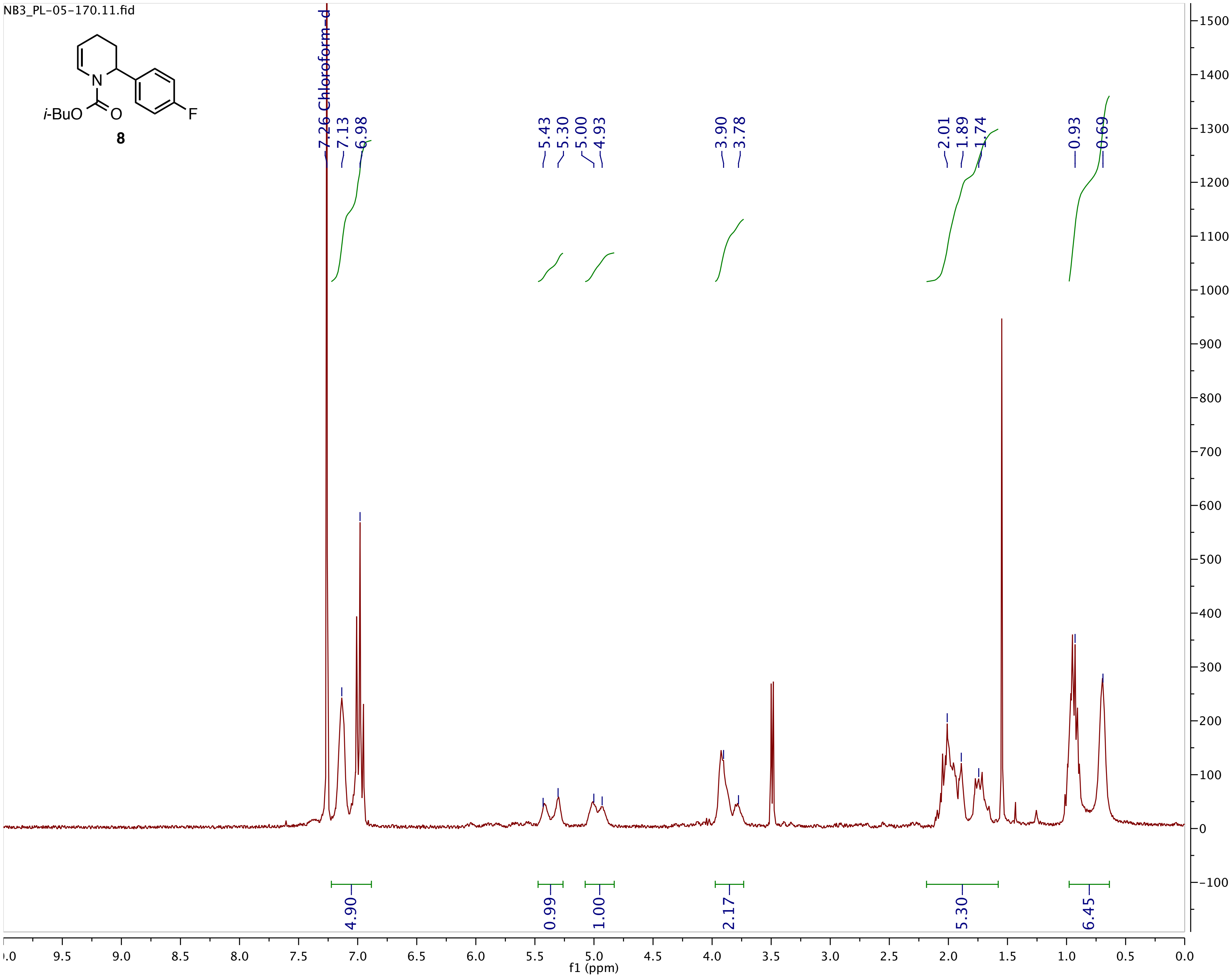

300 MHz ^{1}H-NMR spectrum of **8** in $CDCl_3$

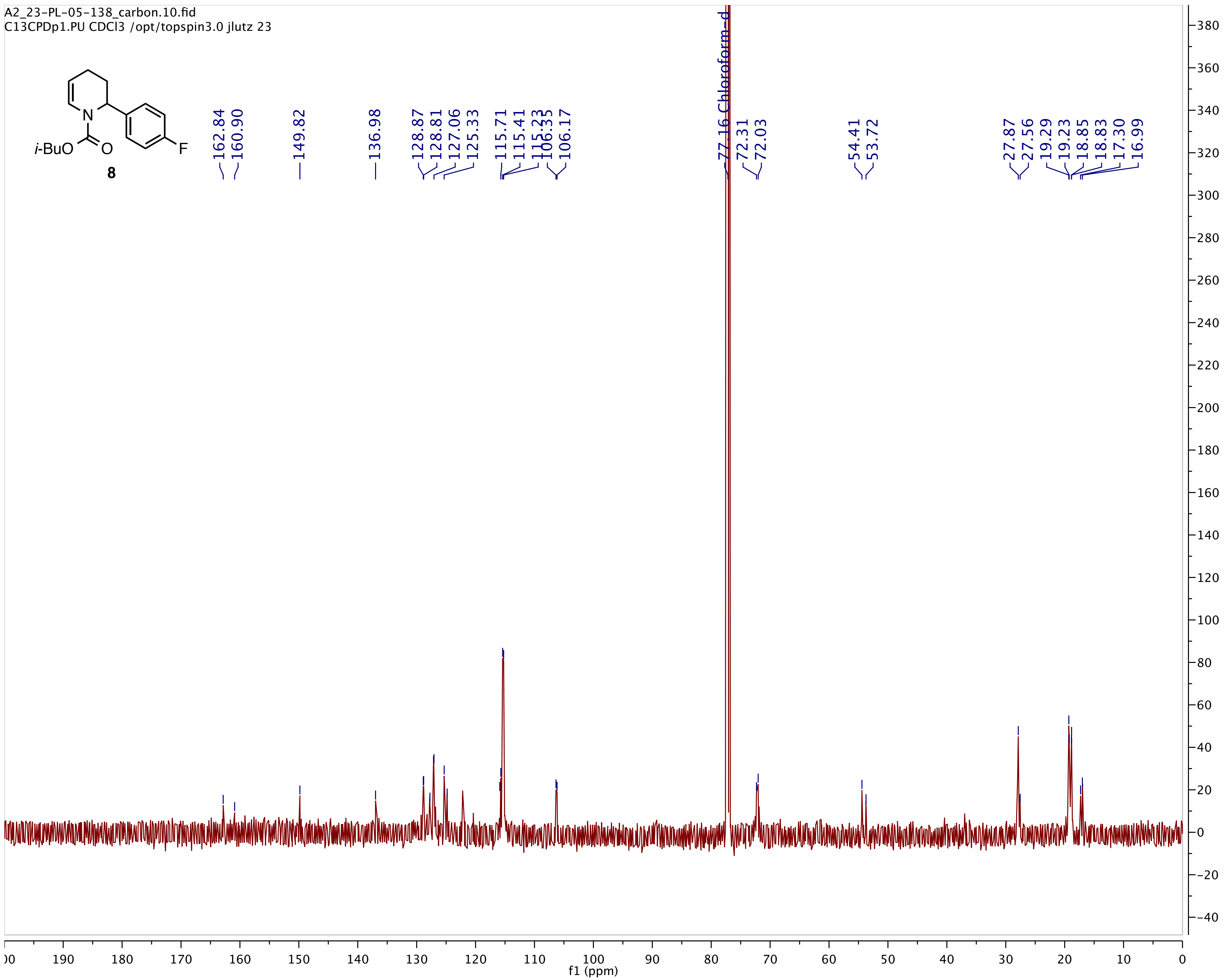

125 MHz ^{13}C-NMR spectrum of **8** in $CDCl_3$

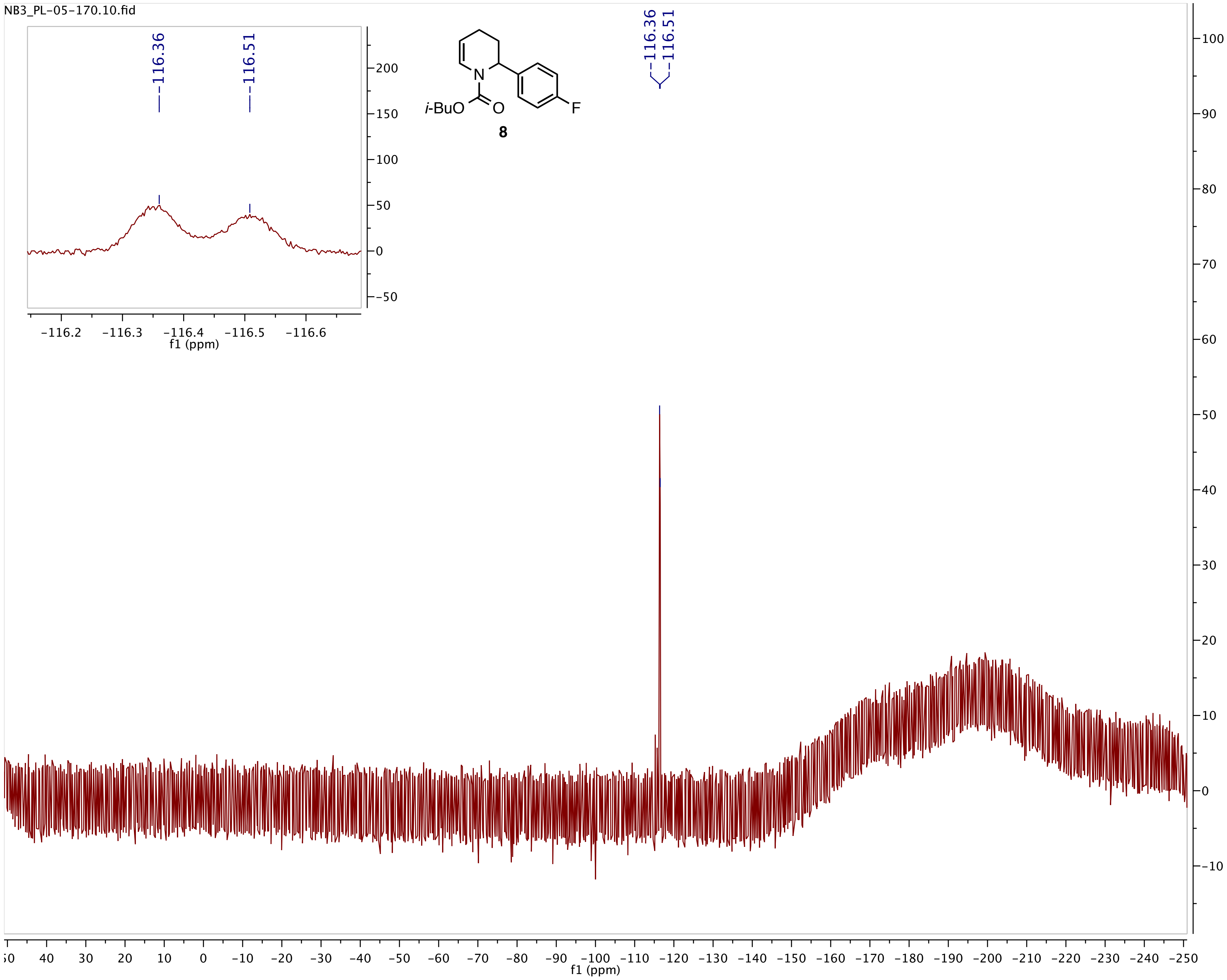

282 MHz ^{19}F-NMR spectrum of **8** in $CDCl_3$

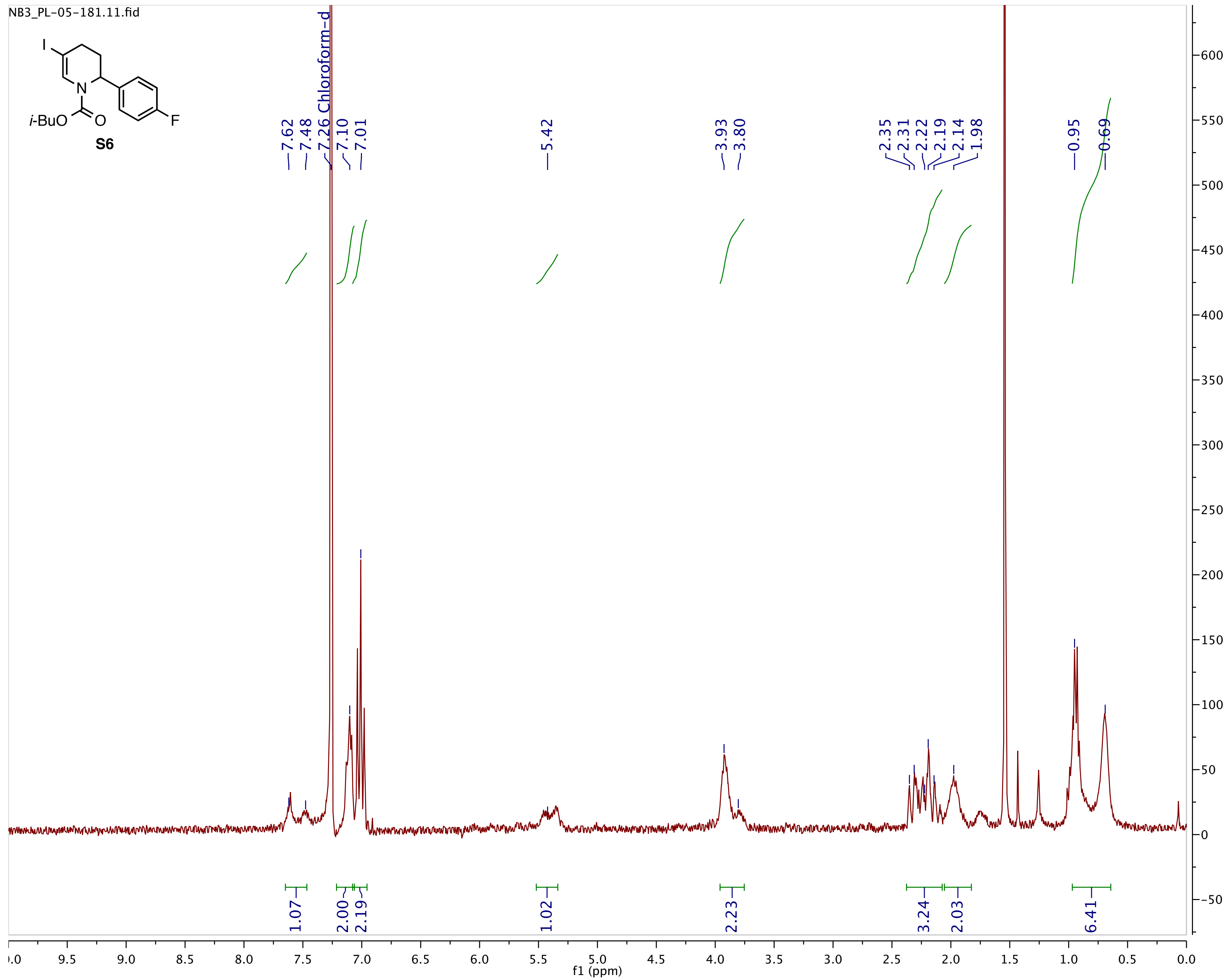

300 MHz ^{1}H-NMR spectrum of **S6** in $CDCl_3$

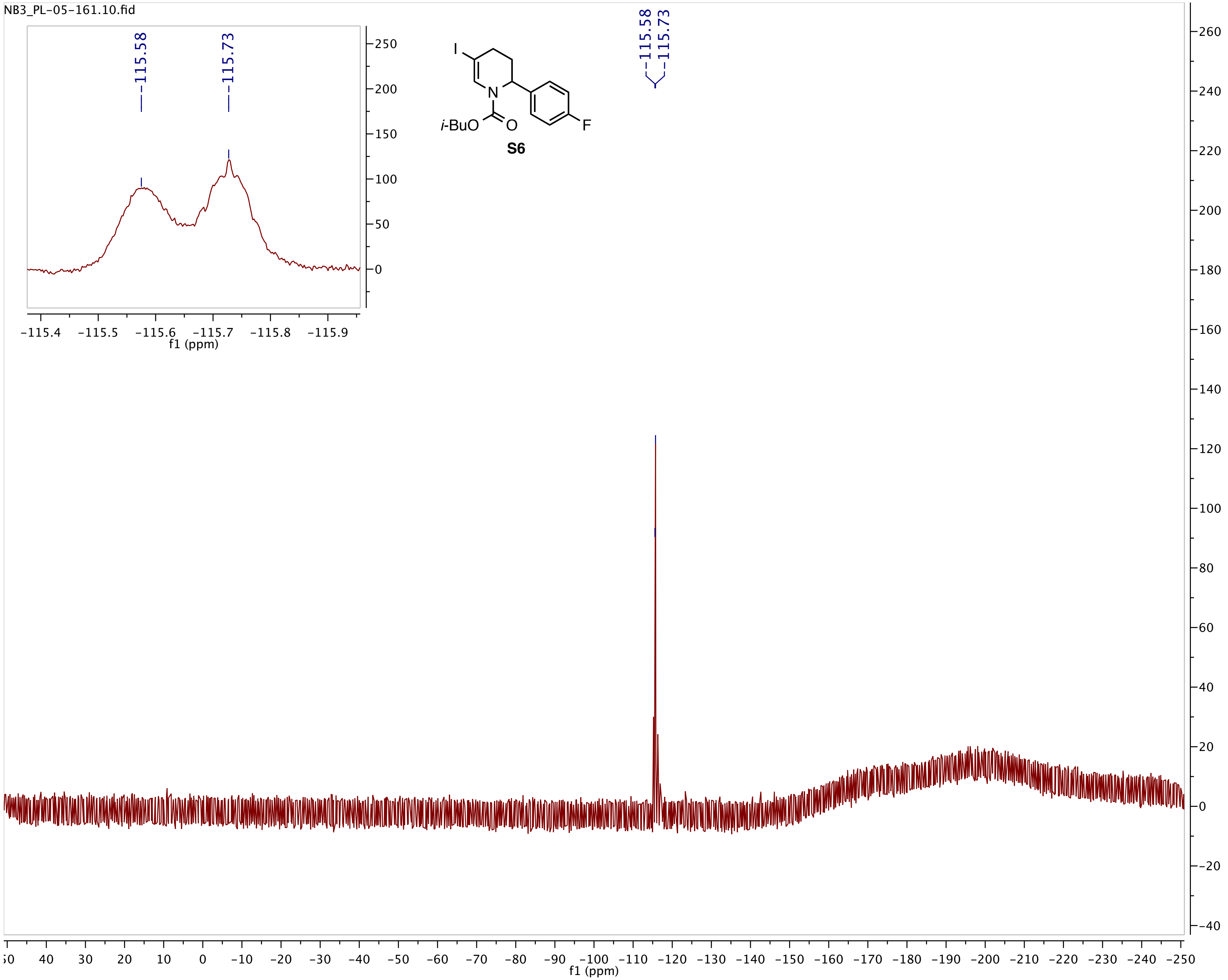

282 MHz ^{19}F-NMR spectrum of **S6** in $CDCl_3$

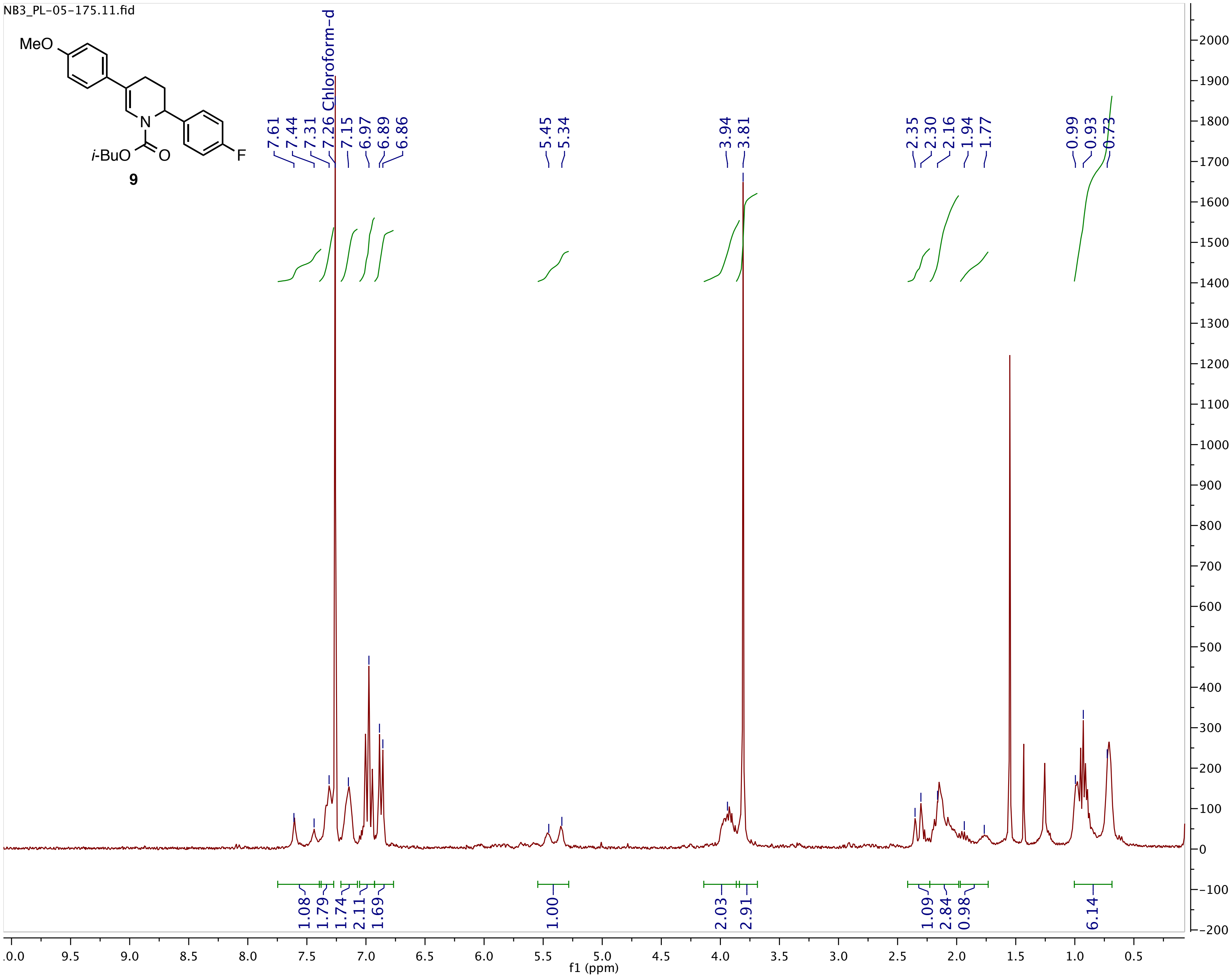

300 MHz ¹H-NMR spectrum of **9** in $CDCl_3$

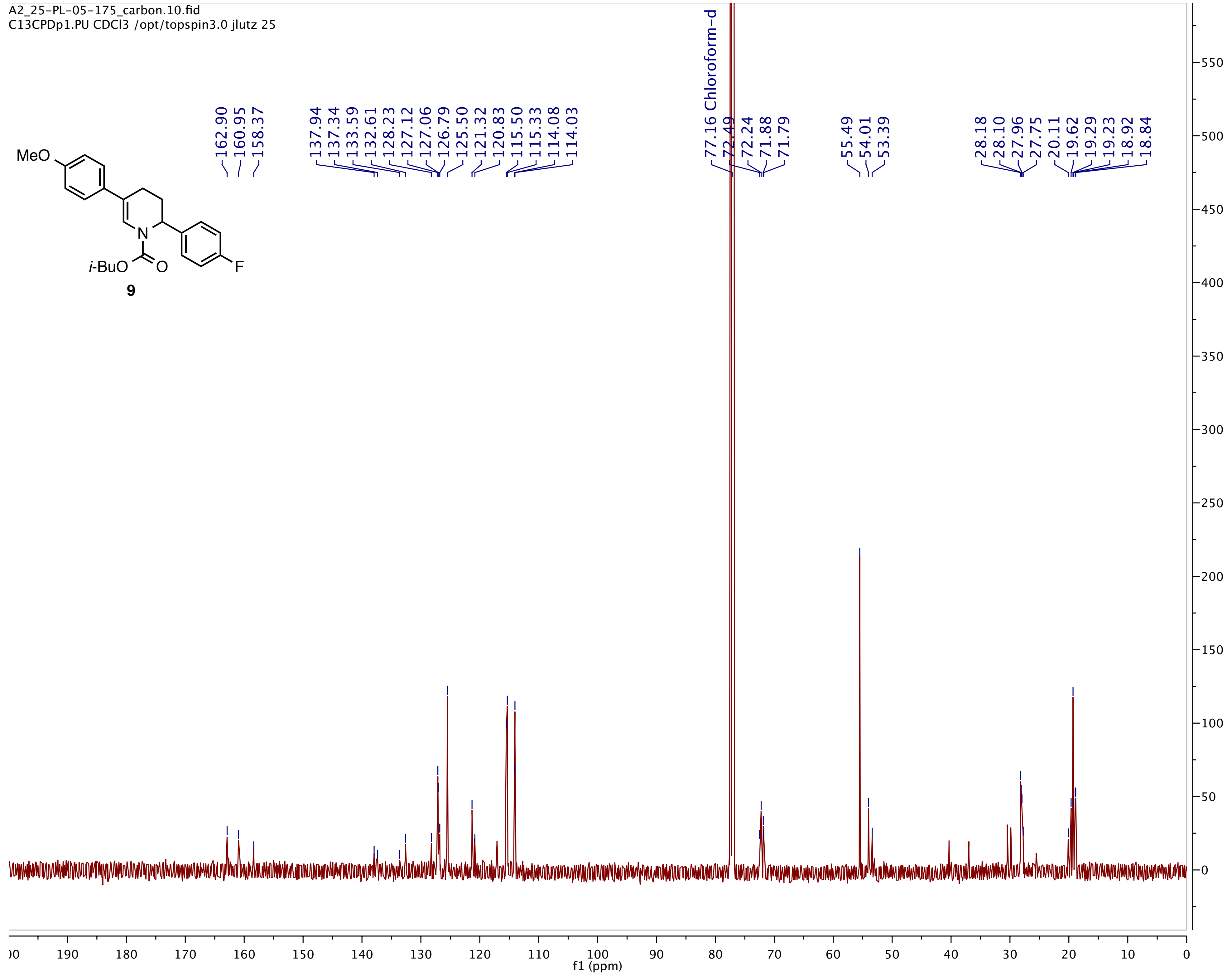

125 MHz ^{13}C-NMR spectrum of **9** in $CDCl_3$

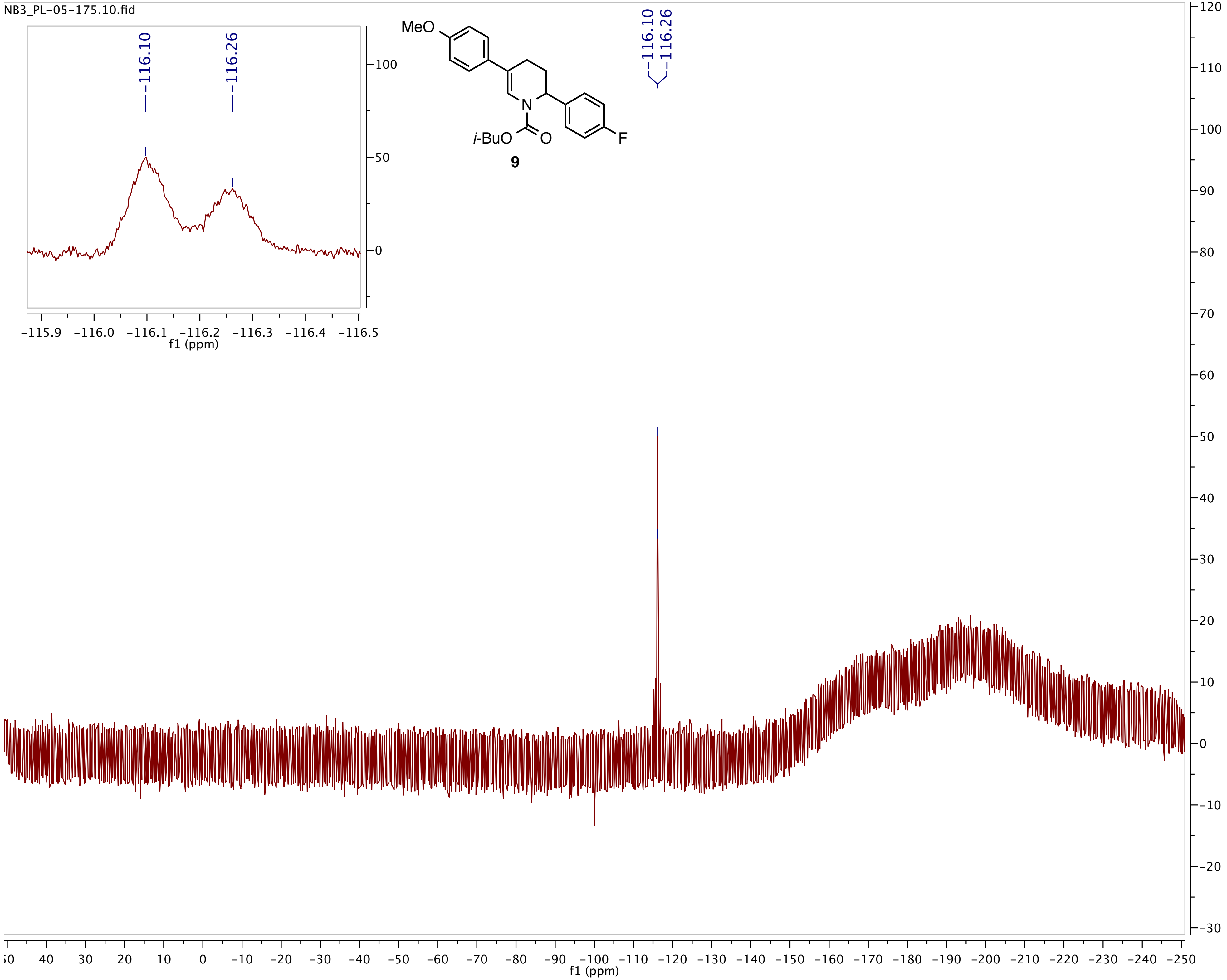

282 MHz ^{19}F-NMR spectrum of **9** in $CDCl_3$

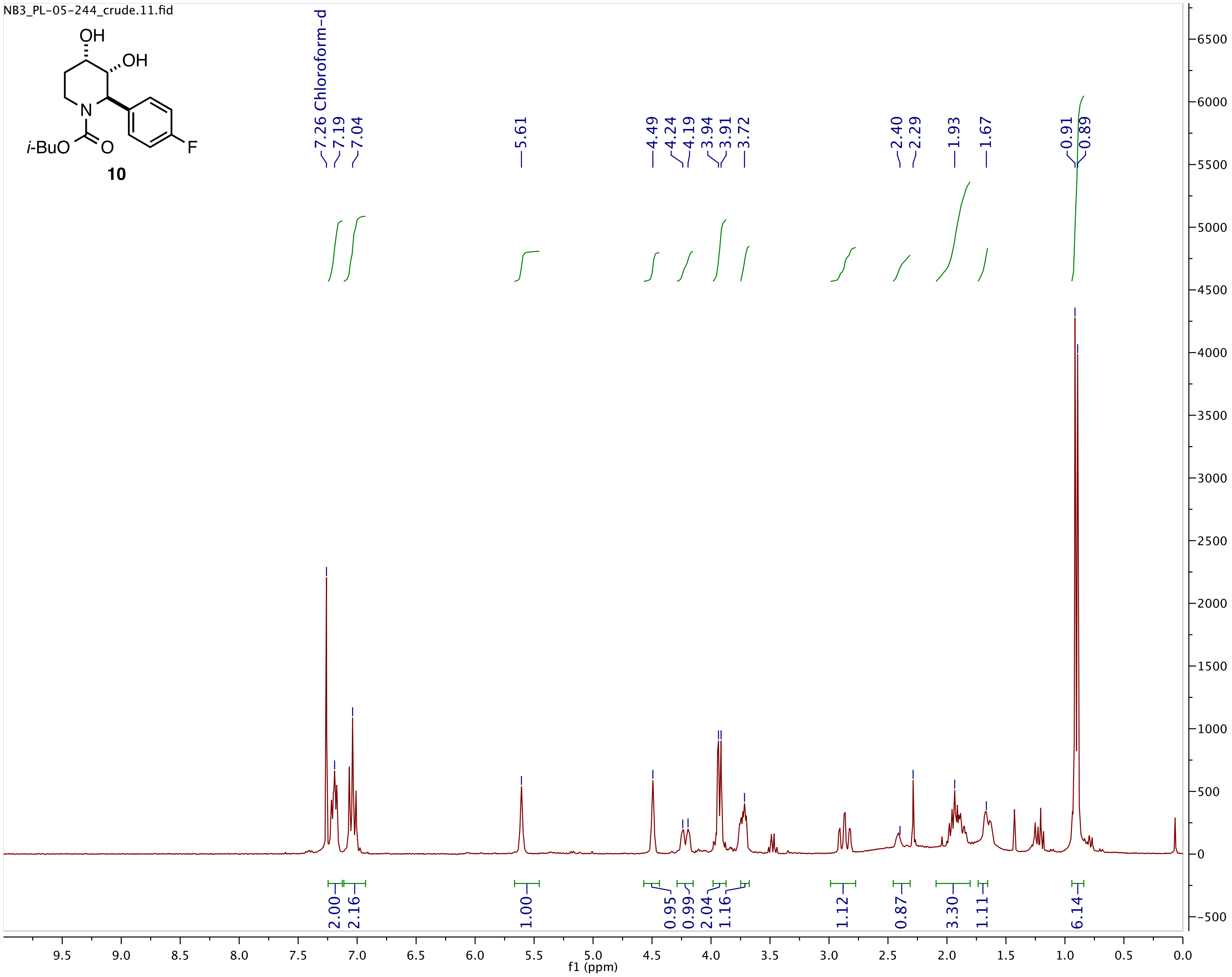

300 MHz ^{1}H-NMR spectrum of **10** in $CDCl_3$

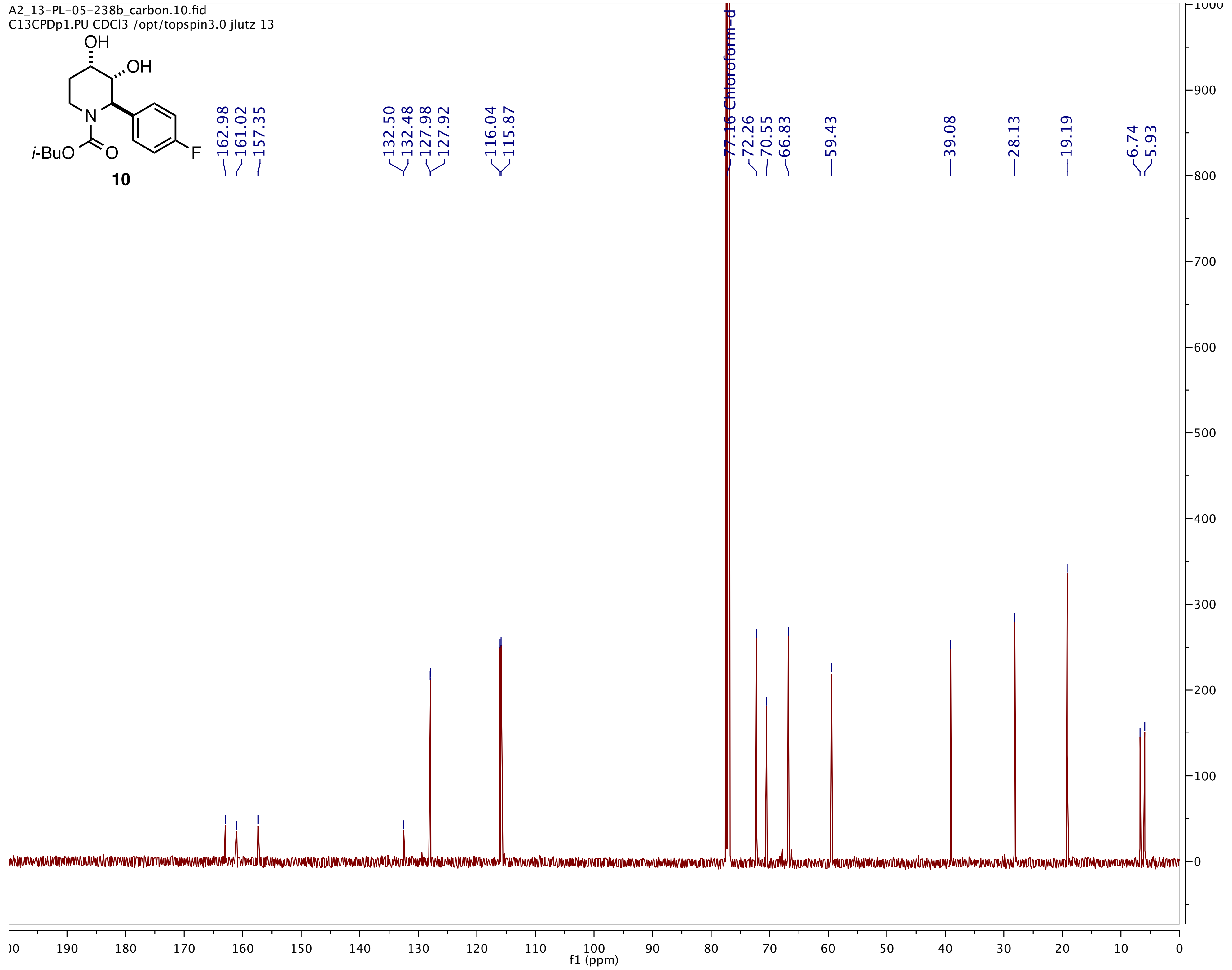

125 MHz ^{13}C-NMR spectrum of **10** in $CDCl_3$

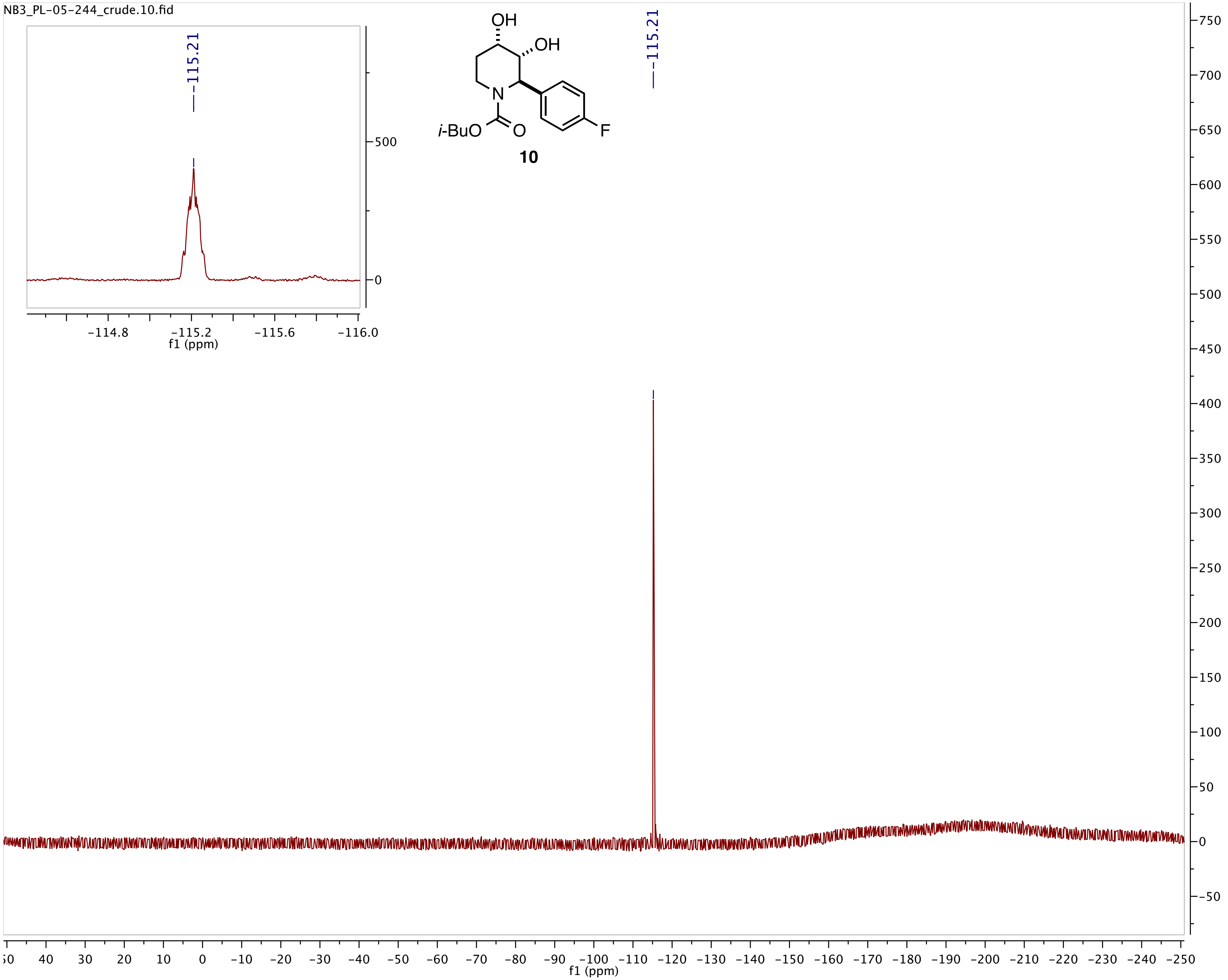

282 MHz ^{19}F-NMR spectrum of **10** in $CDCl_3$

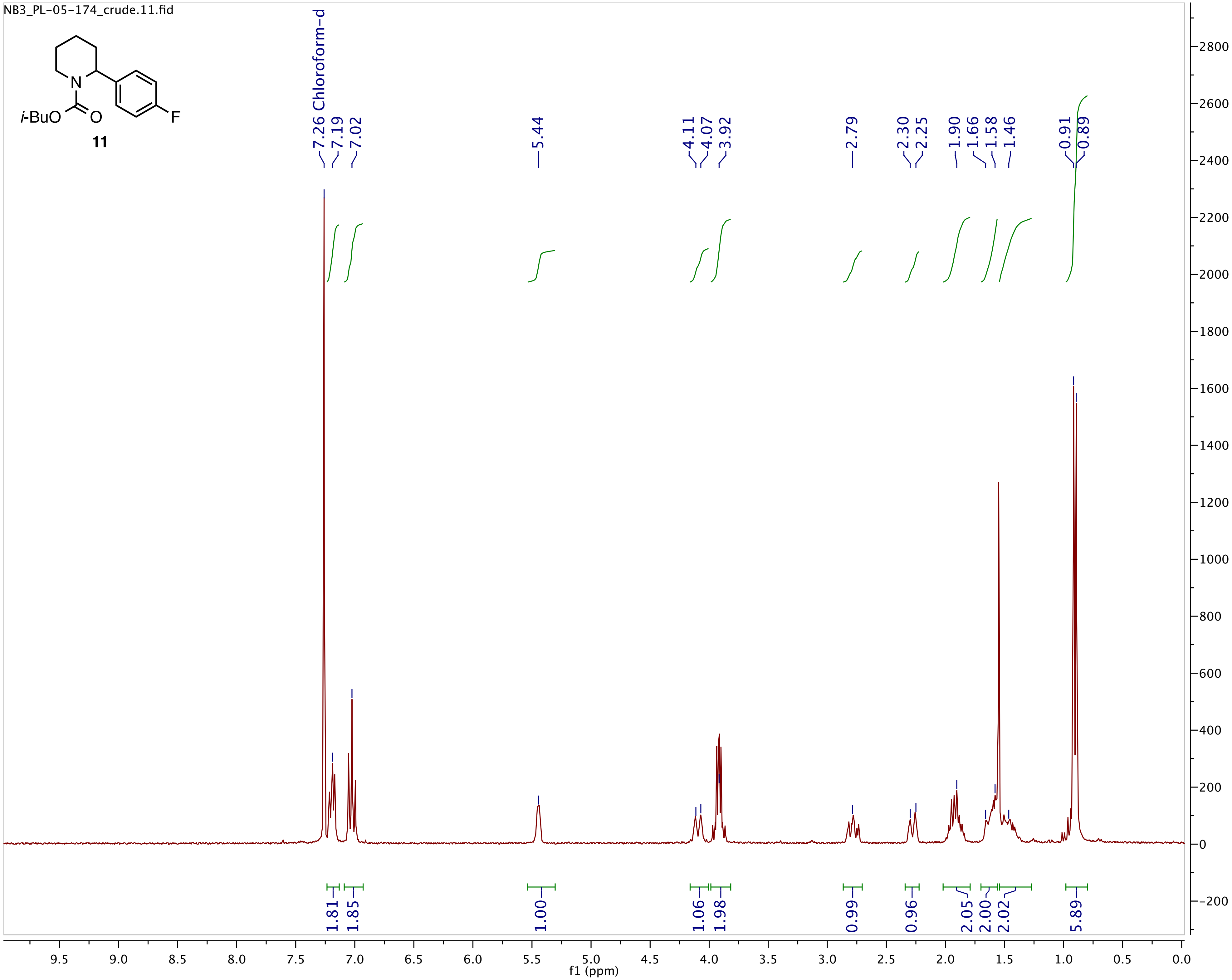

300 MHz ^{1}H-NMR spectrum of **11** in $CDCl_3$

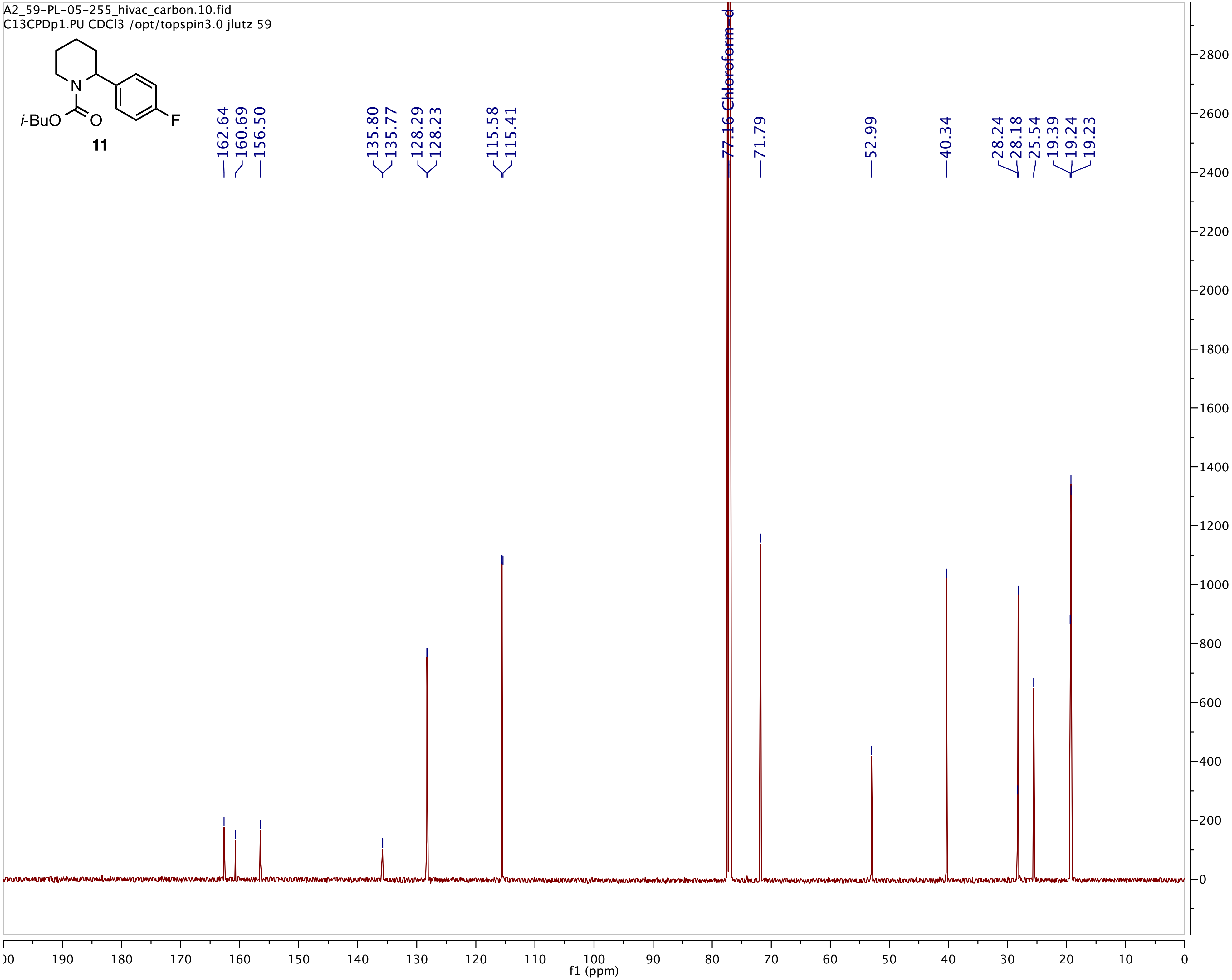

125 MHz ^{13}C-NMR spectrum of **11** in $CDCl_3$

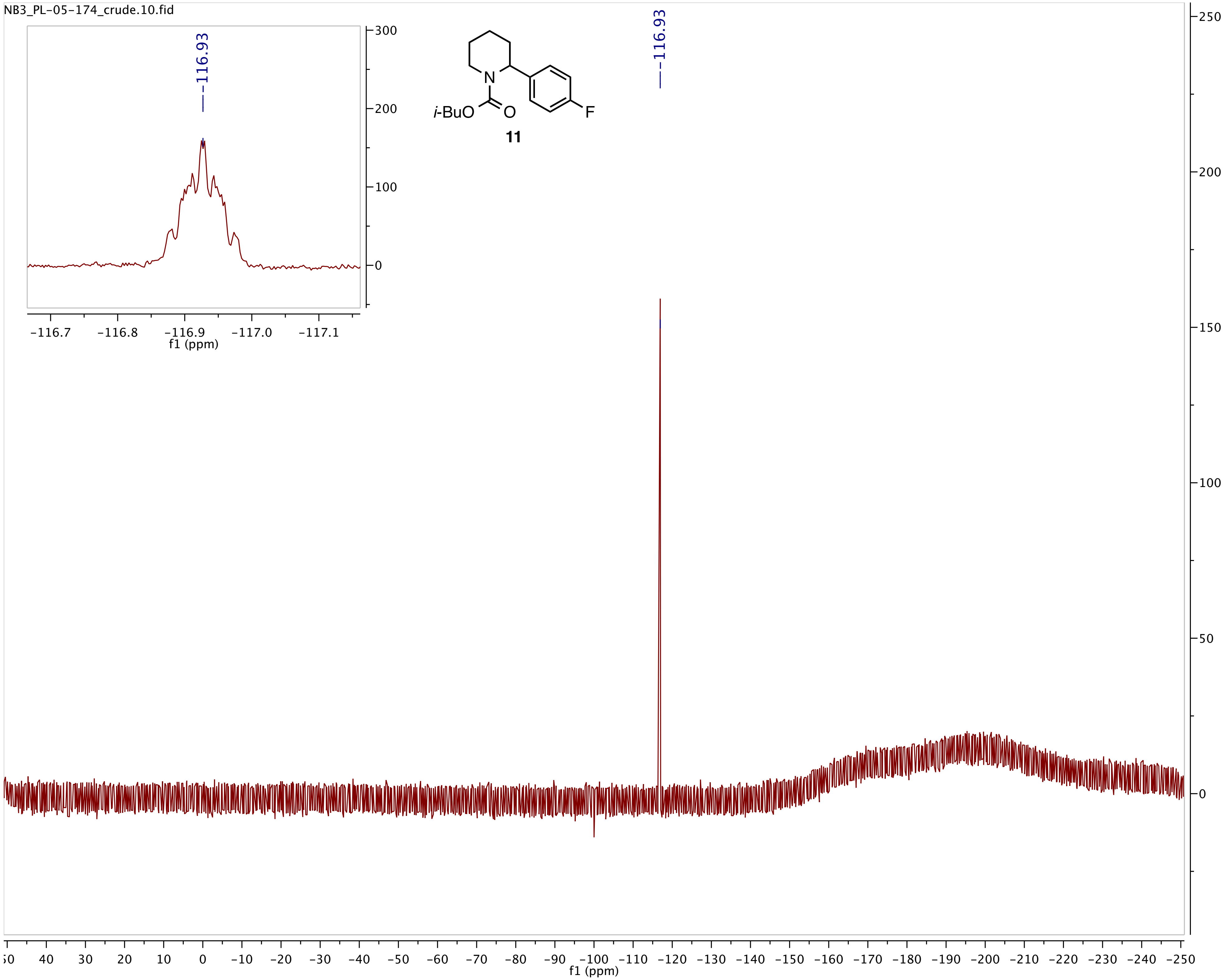

282 MHz ^{19}F-NMR spectrum of **11** in $CDCl_3$

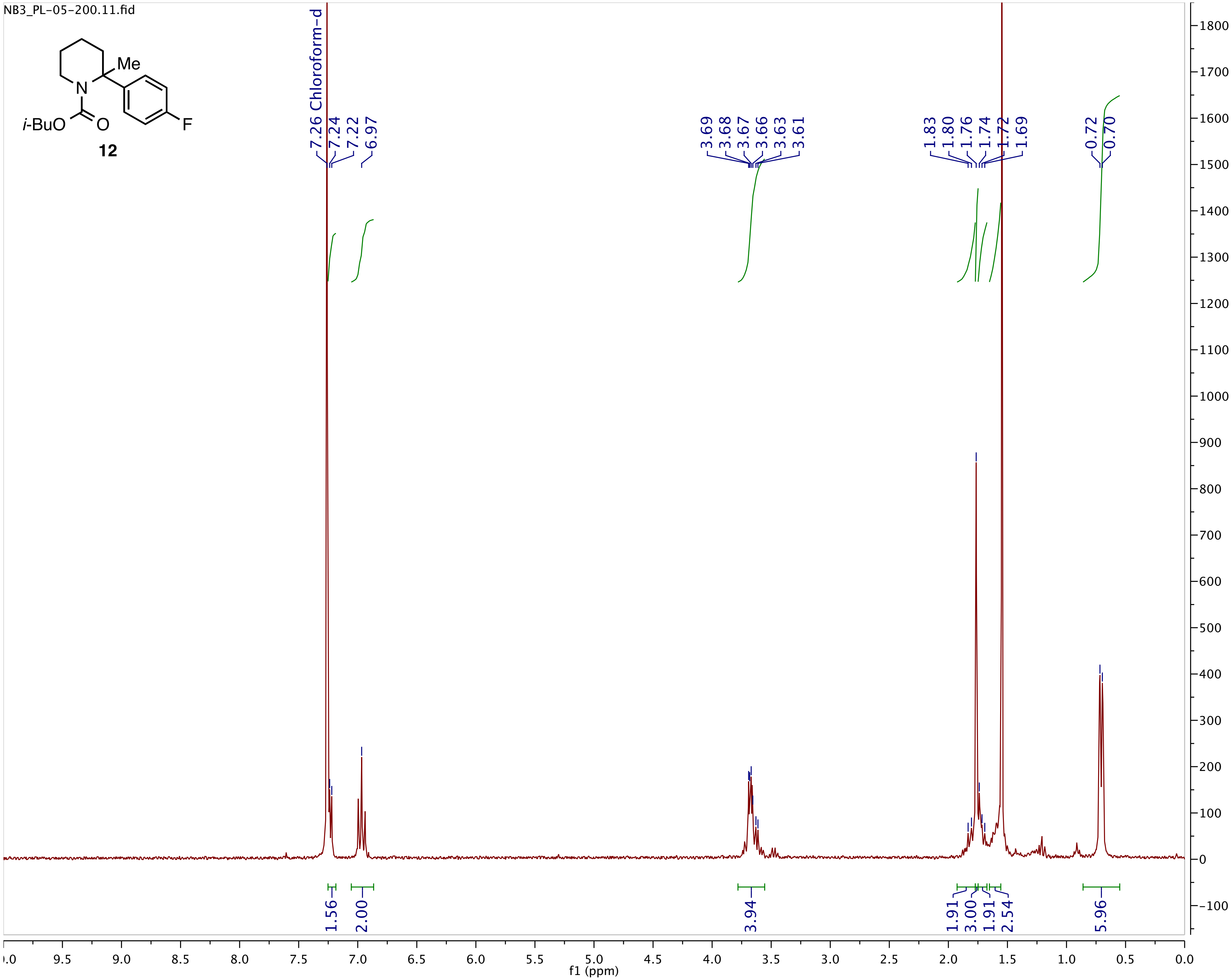

300 MHz ^{1}H-NMR spectrum of **12** in $CDCl_3$

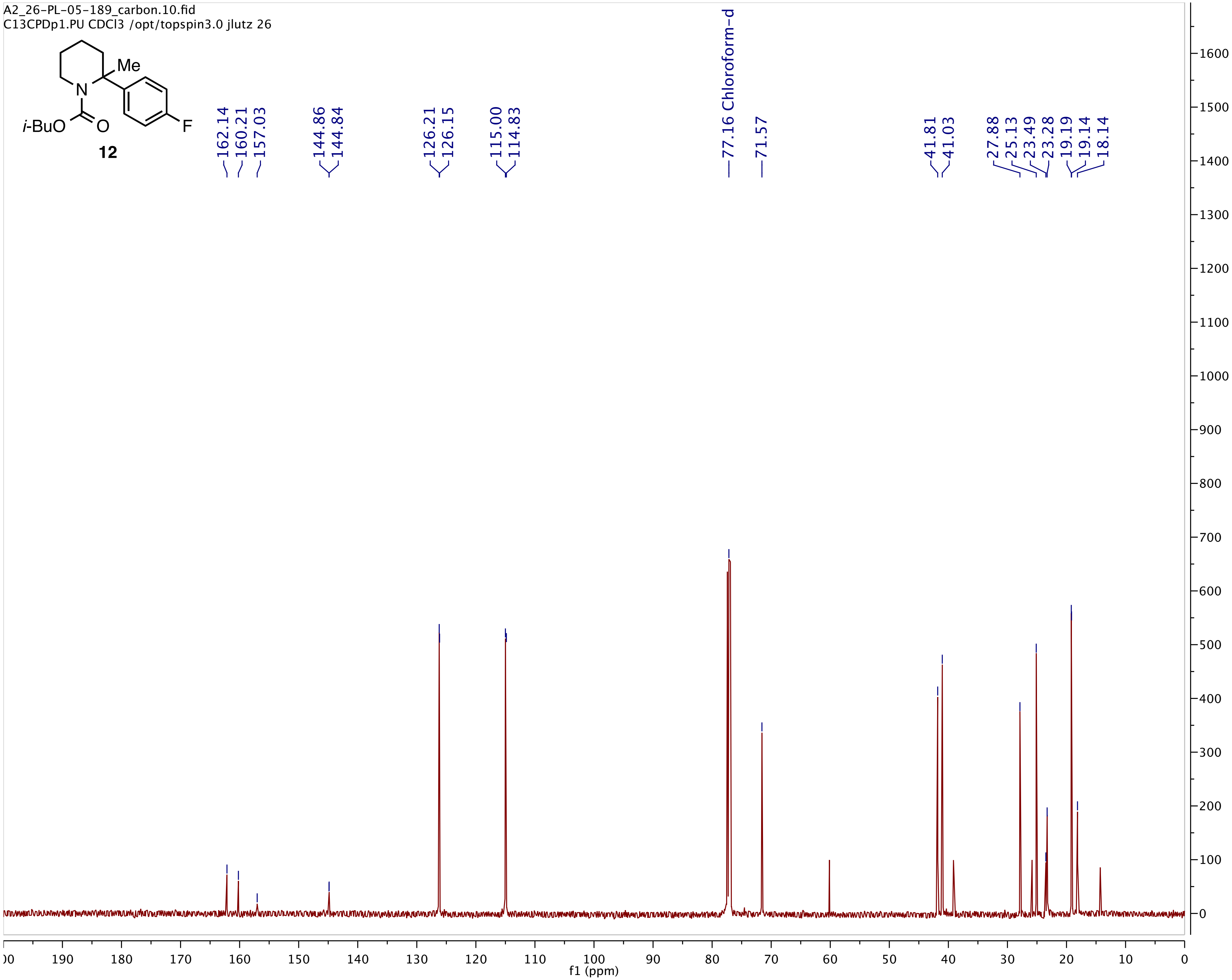

300 MHz ^{13}C-NMR spectrum of **12** in $CDCl_3$

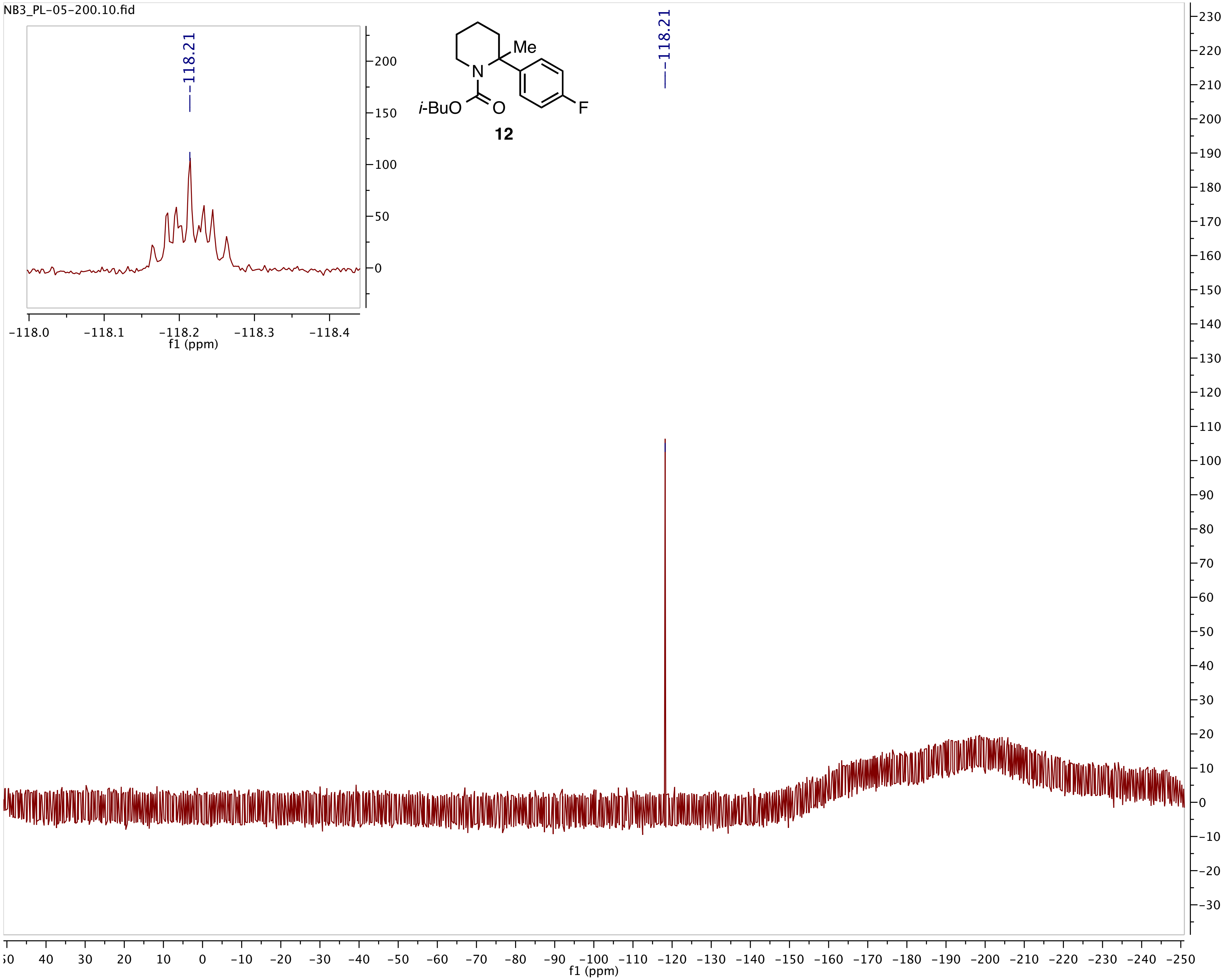

470 MHz ^{19}F-NMR spectrum of **12** in $CDCl_3$

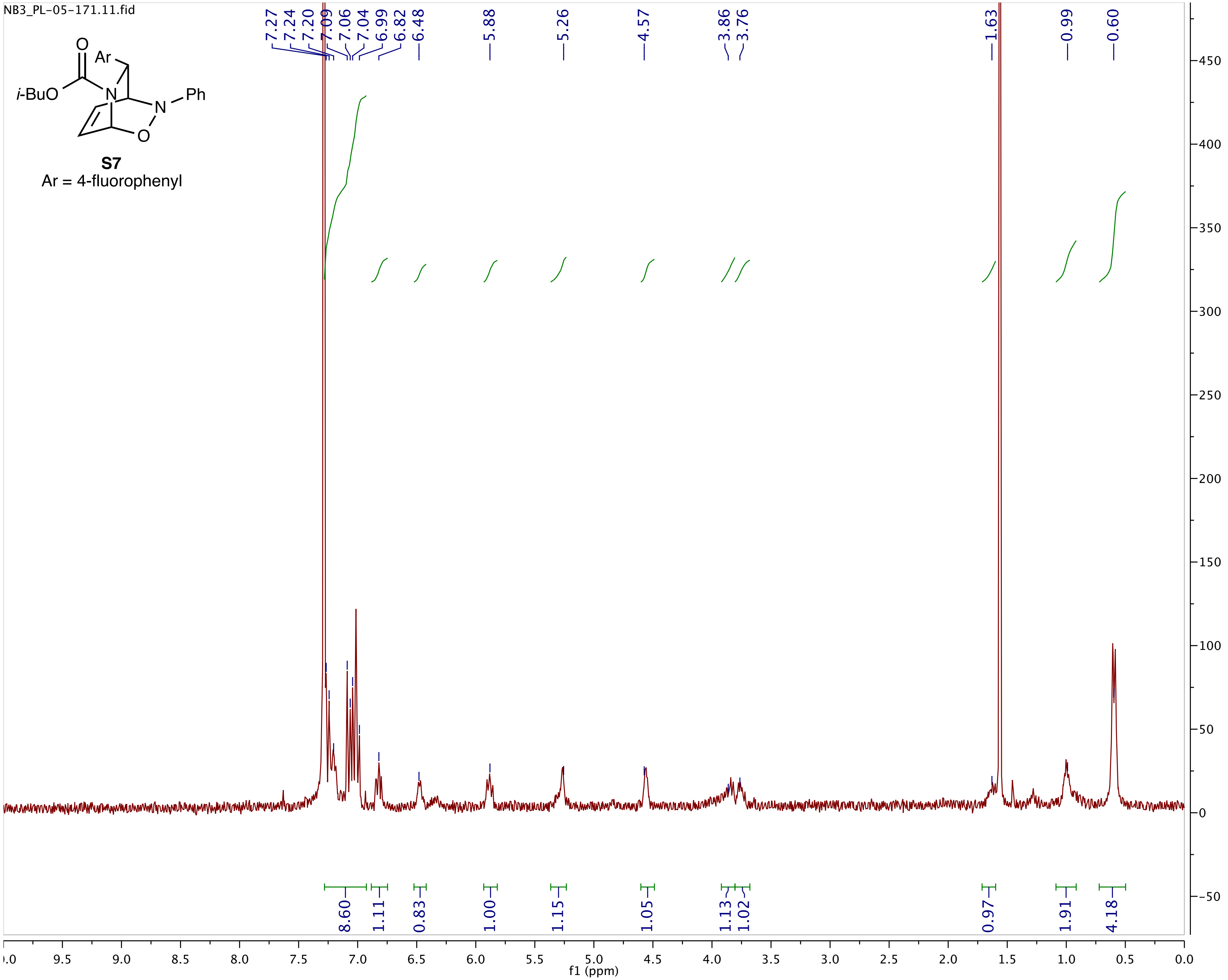

300 MHz ¹H-NMR spectrum of **S7** in $CDCl_3$

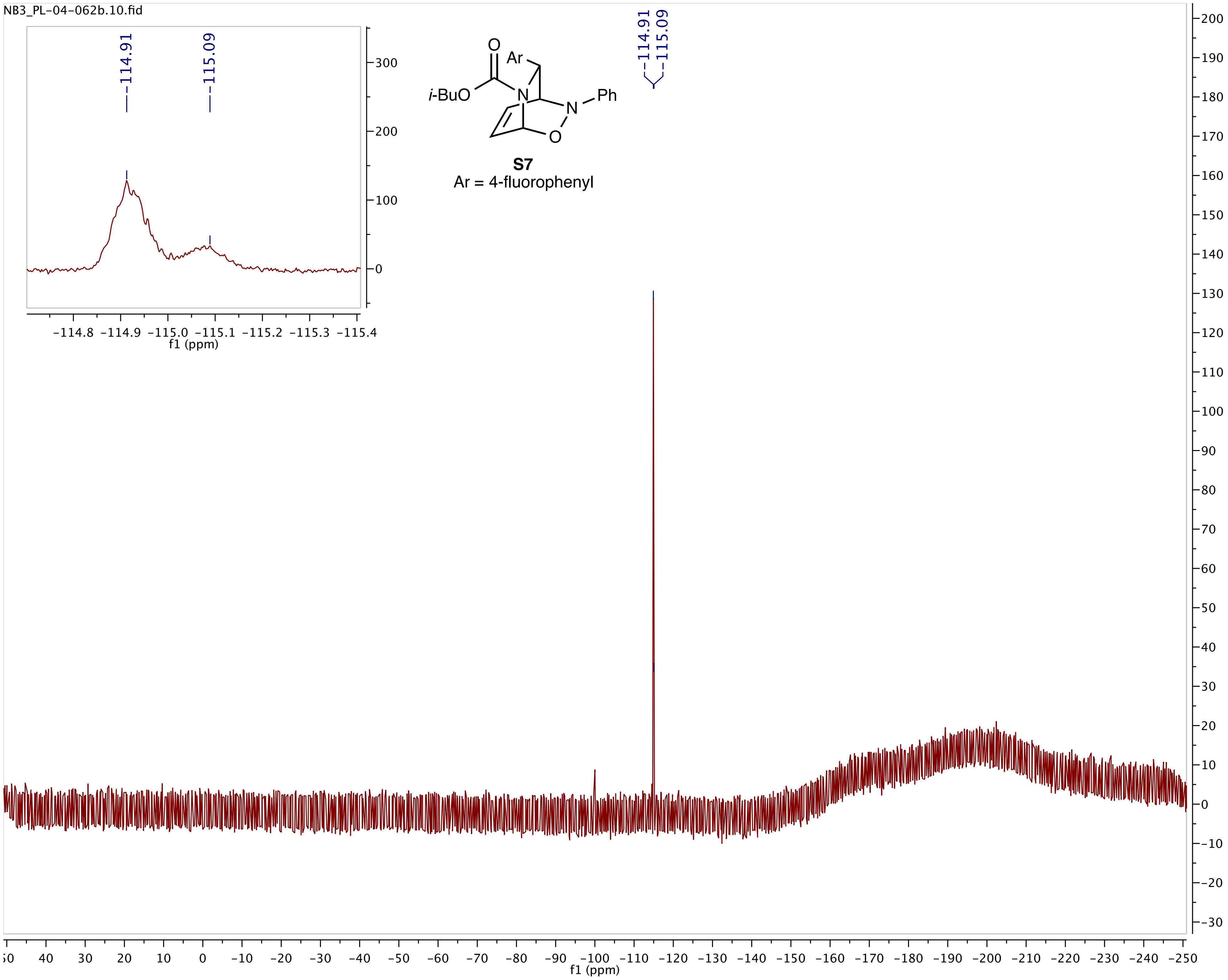

282 MHz ^{19}F-NMR spectrum of **S7** in $CDCl_3$

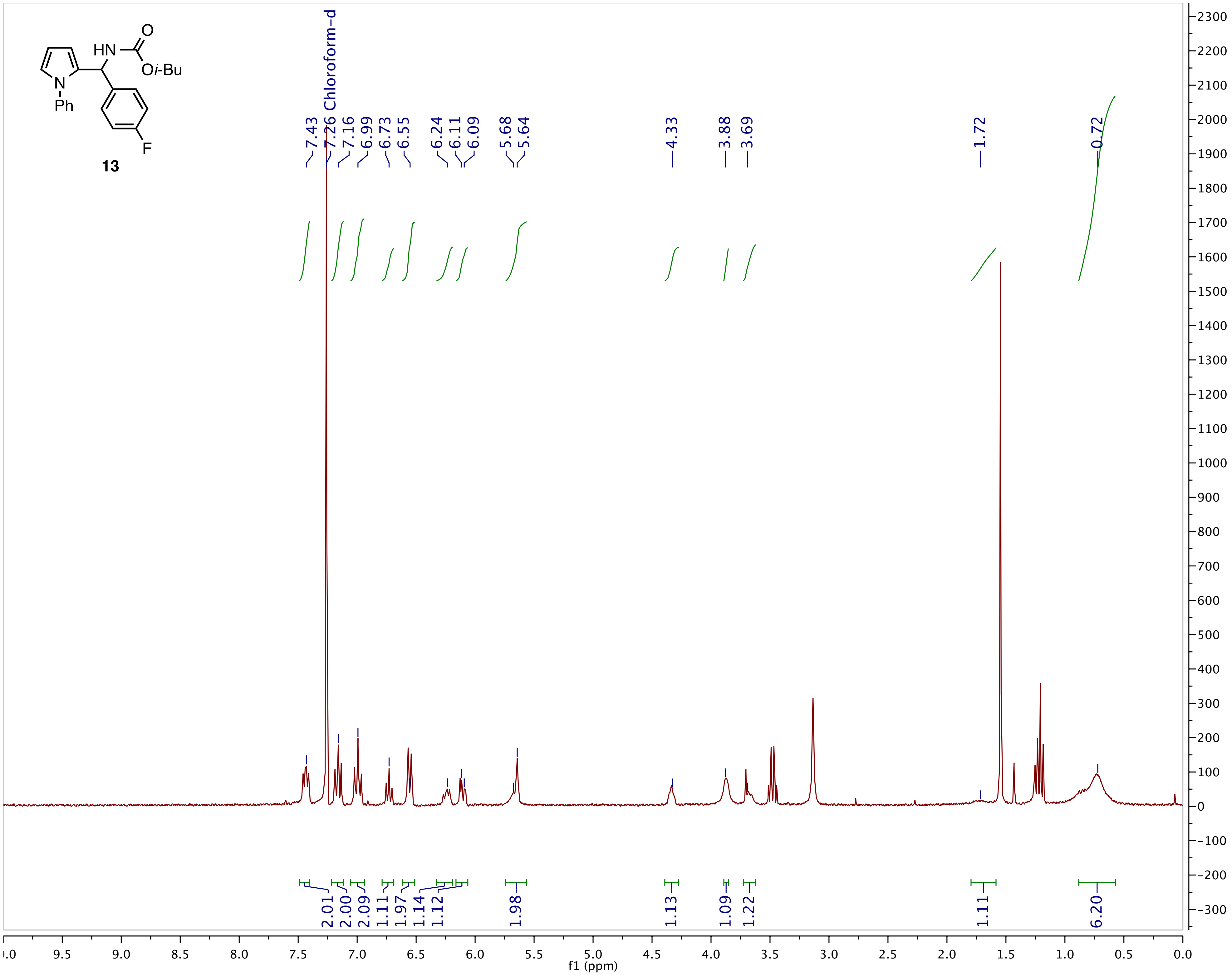

300 MHz ^{1}H-NMR spectrum of **13** in $CDCl_3$

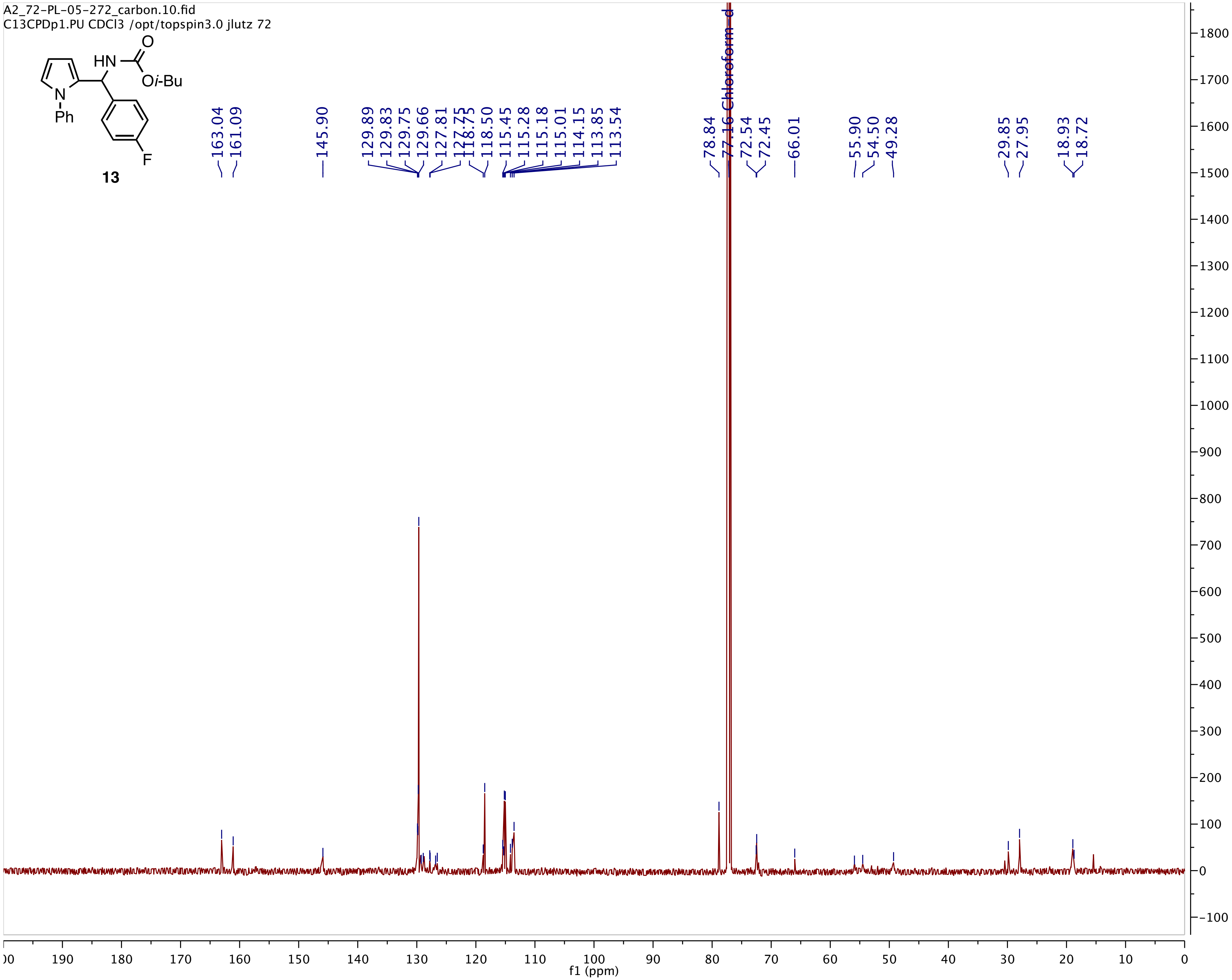

125 MHz ^{13}C-NMR spectrum of **13** in $CDCl_3$

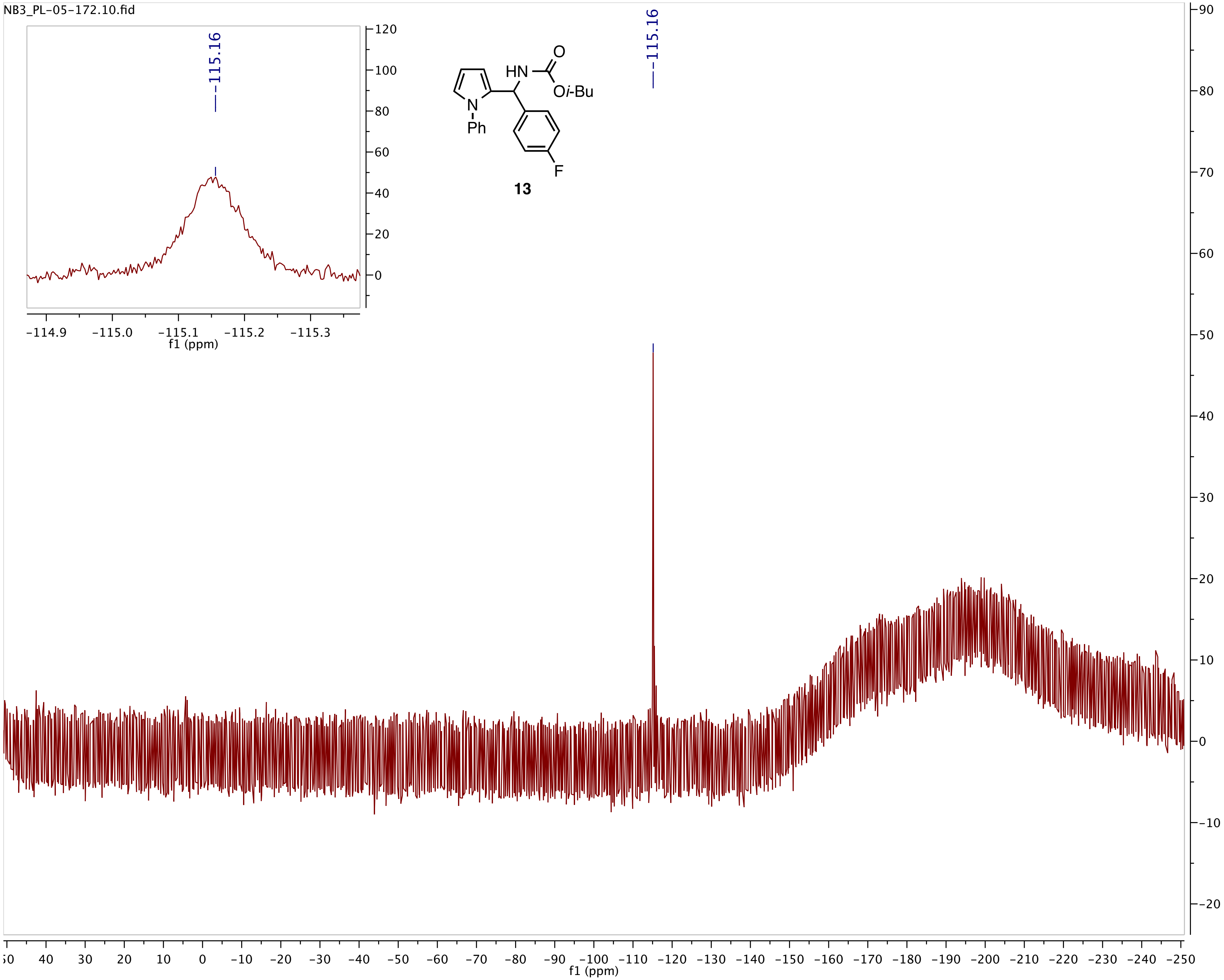

282 MHz ^{19}F-NMR spectrum of **13** in $CDCl_3$

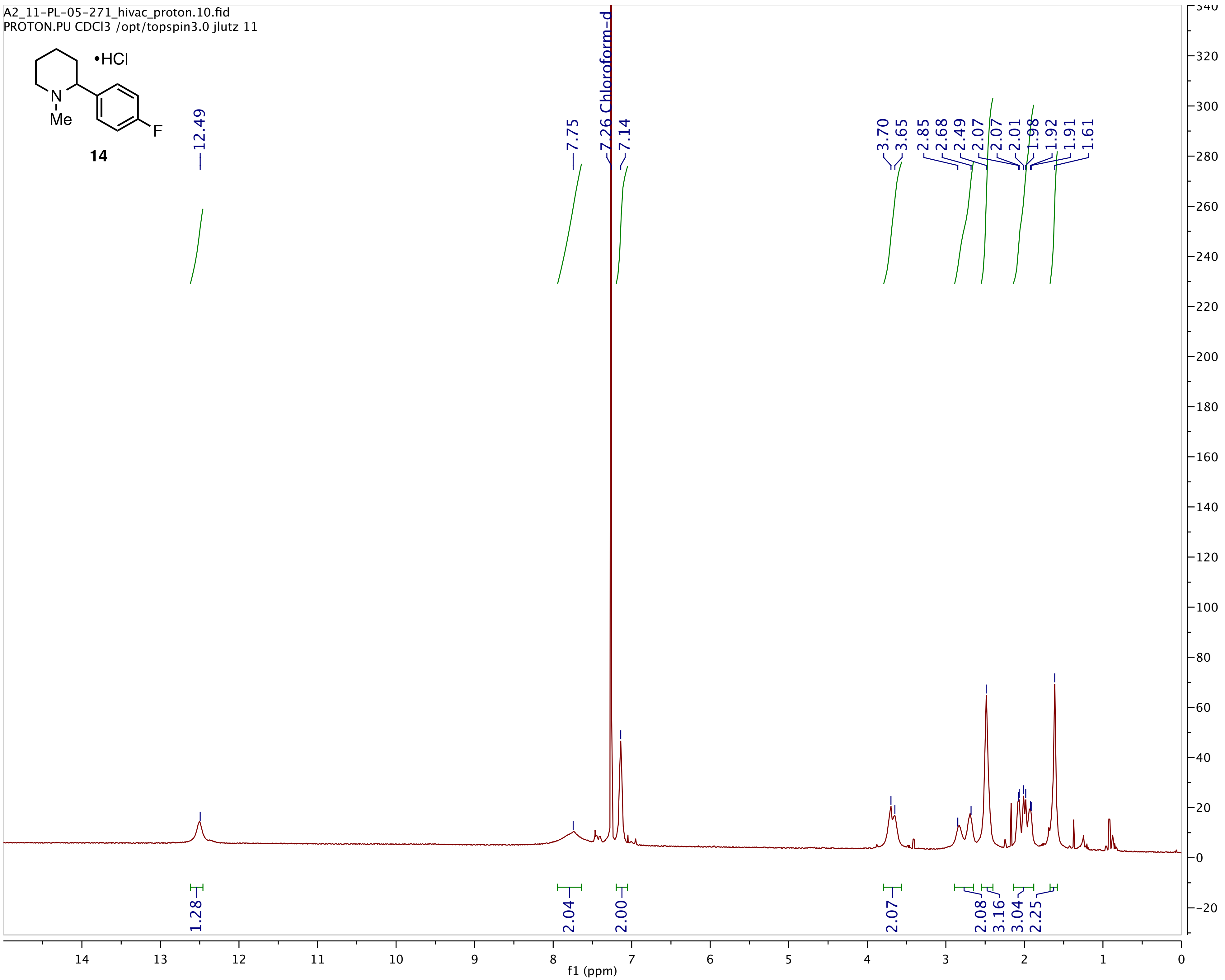

500 MHz ^{1}H-NMR spectrum of **14** in $CDCl_3$

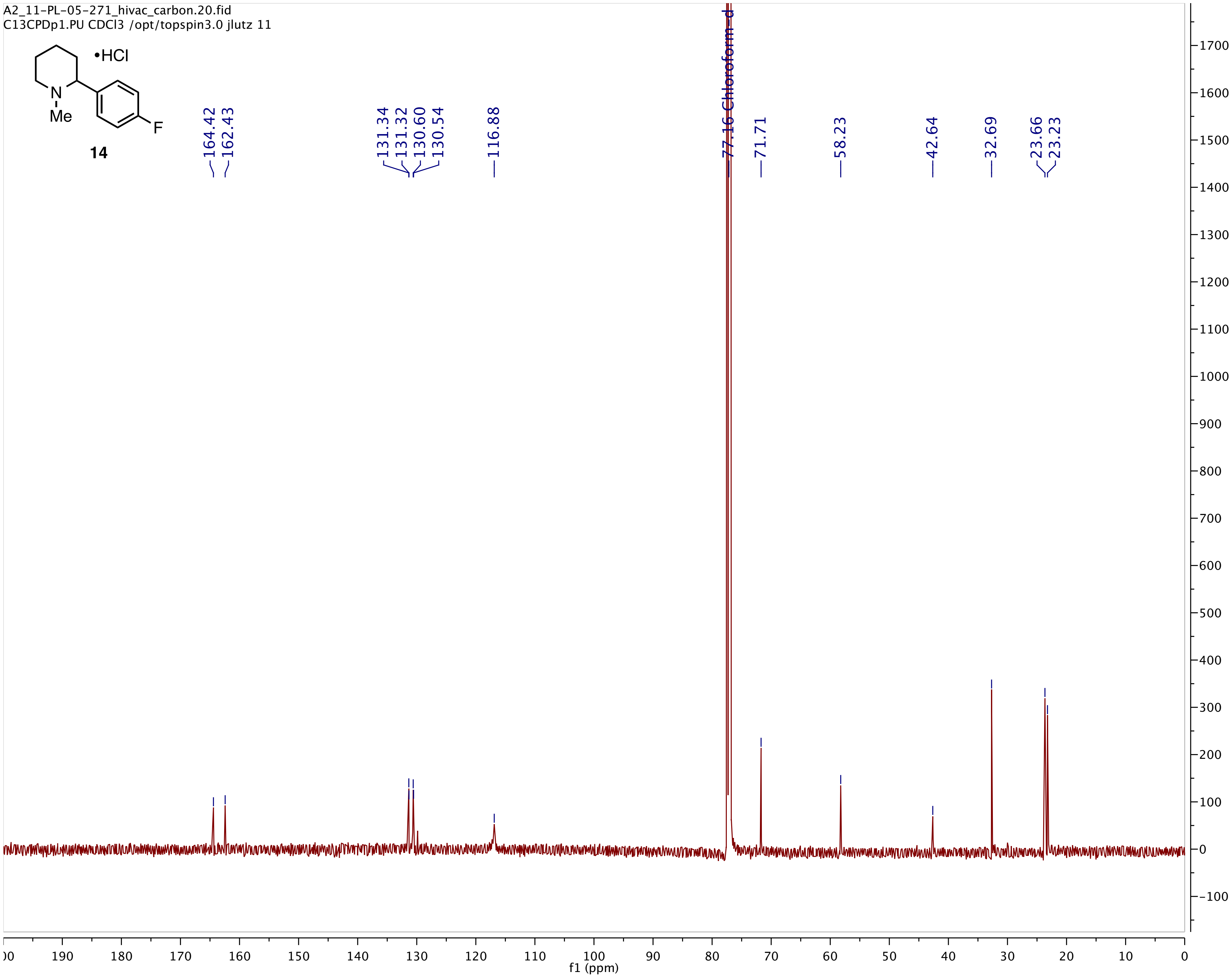

125 MHz ^{13}C-NMR spectrum of **14** in $CDCl_3$

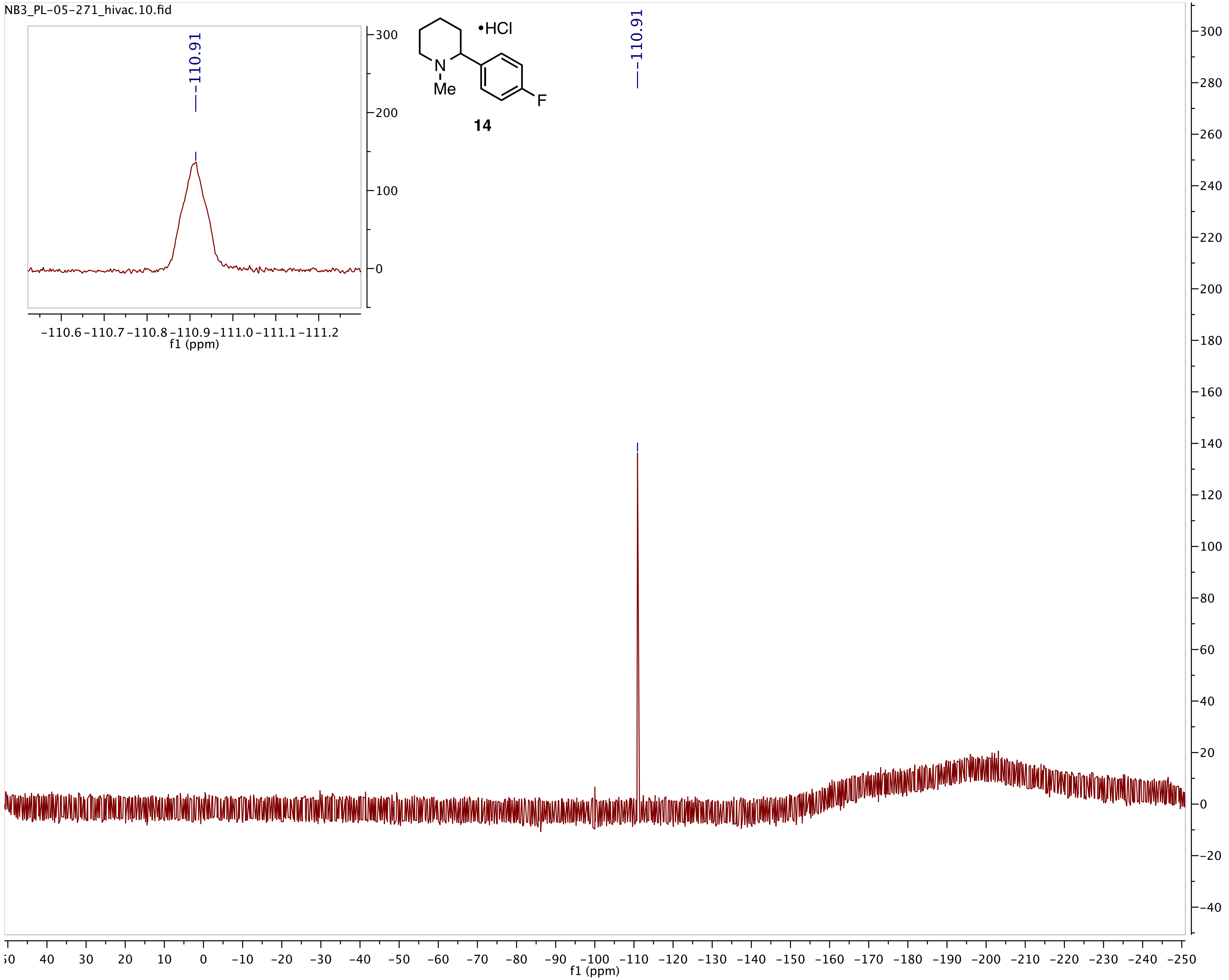

282 MHz ^{19}F-NMR spectrum of **14** in $CDCl_3$

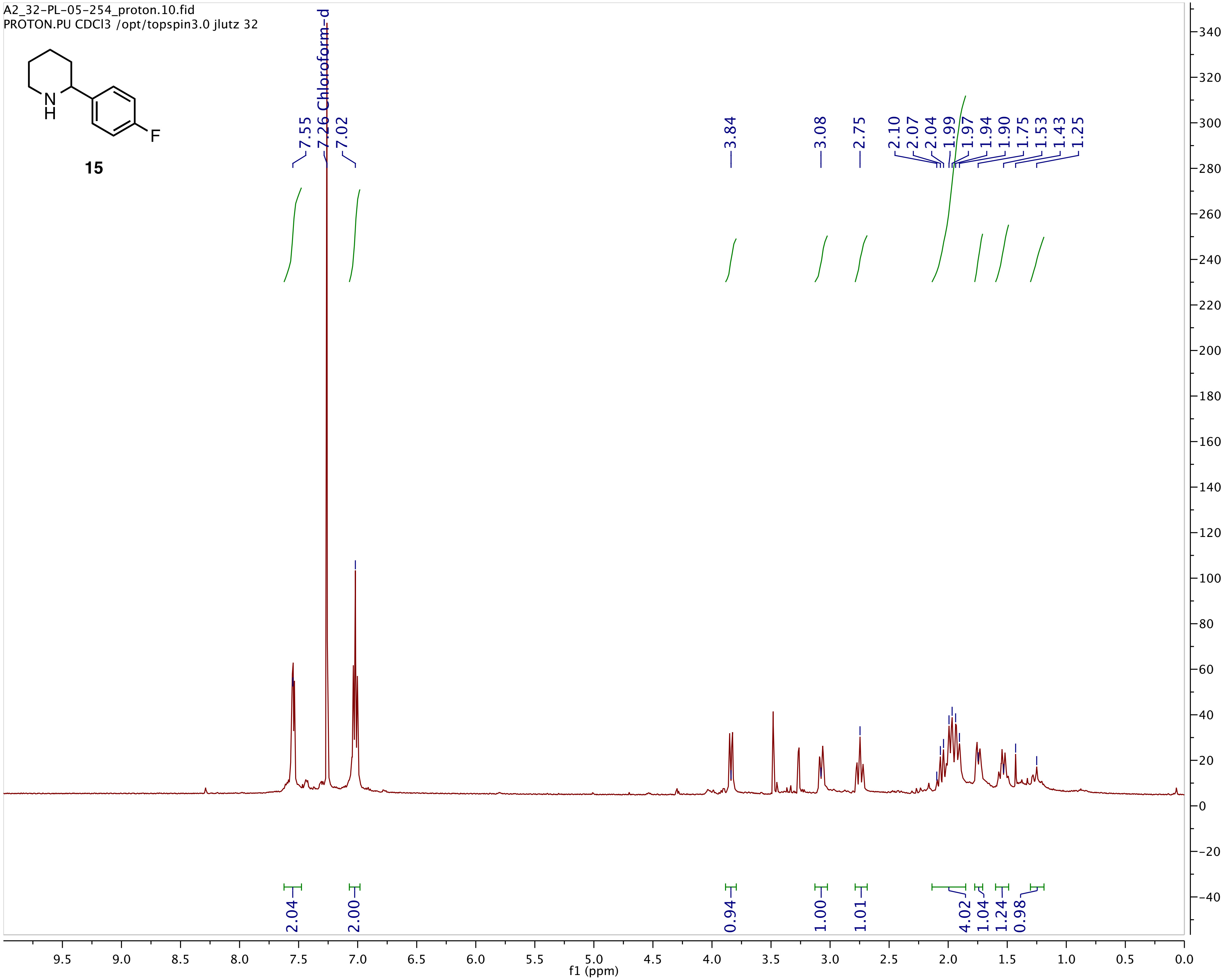

500 MHz ^{1}H-NMR spectrum of **15** in $CDCl_3$

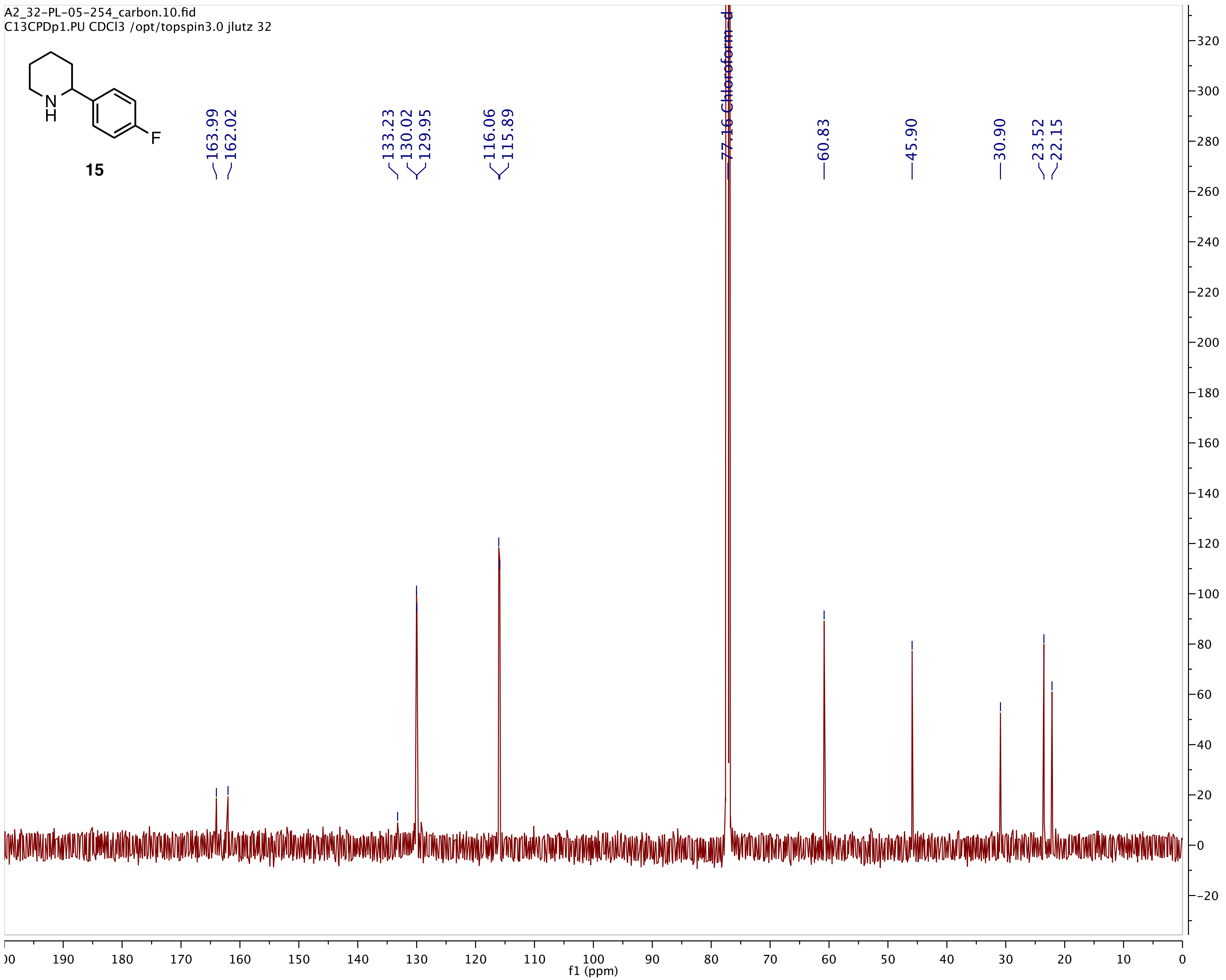

125 MHz ^{13}C-NMR spectrum of **15** in $CDCl_3$

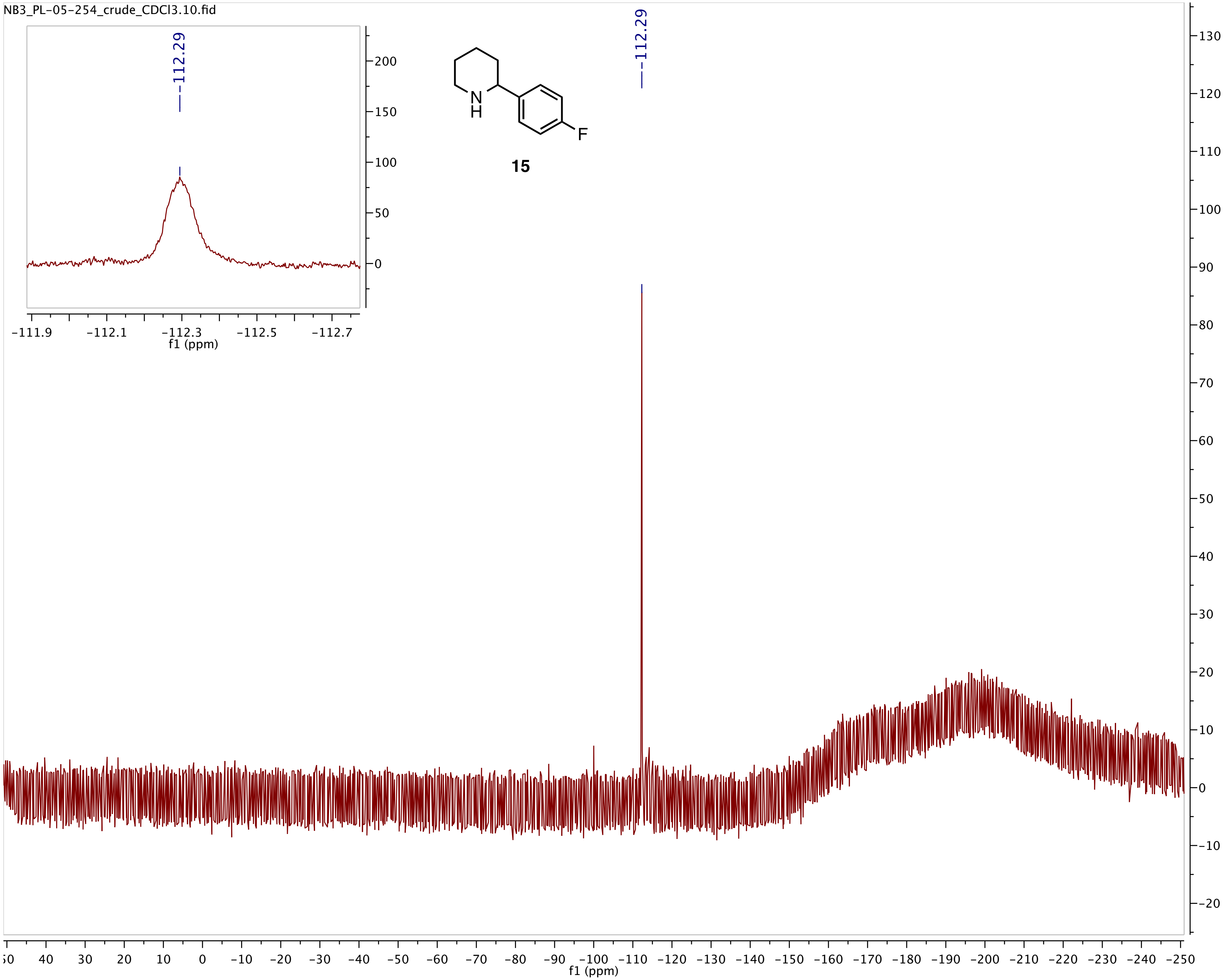

282 MHz ^{19}F-NMR spectrum of **15** in $CDCl_3$
